# Selective Reduction of Barbituric Acids Using SmI_2_/H_2_O: Synthesis, Reactivity, and Structural Analysis of Tetrahedral Adducts**

**DOI:** 10.1002/anie.201306484

**Published:** 2013-10-09

**Authors:** Michal Szostak, Brice Sautier, Malcolm Spain, Maike Behlendorf, David J Procter

**Affiliations:** School of Chemistry, University of ManchesterOxford Road, Manchester M13 9PL (UK)

**Keywords:** electron transfer, heterocycles, reductive coupling, samarium iodide, synthetic methods

Since the 1864 landmark discovery by Adolf von Baeyer,^1^ barbituric acids have played a prominent role in medicine and organic synthesis. The barbituric acid scaffold occurs in more than 5000 pharmacologically active compounds, including commonly used anticonvulsant, hypnotic, and anticancer agents (Figure 1 a).^2^ Moreover, as an easily accessible feedstock material, it is an extremely useful building block for organic synthesis.^3^ However, despite the fact that barbiturates have been extensively studied for over a century, the general monoreduction of barbituric acids remains unknown,^4^ even though it would have considerable potential for the production and discovery of pharmaceuticals, materials, and polymers. Interestingly, the barbiturate monoreduction products would formally constitute a new class of tetrahedral intermediates of amide bond addition reactions, only few of which have been successfully isolated to date because of their transient nature.^5^

**Figure 1:**
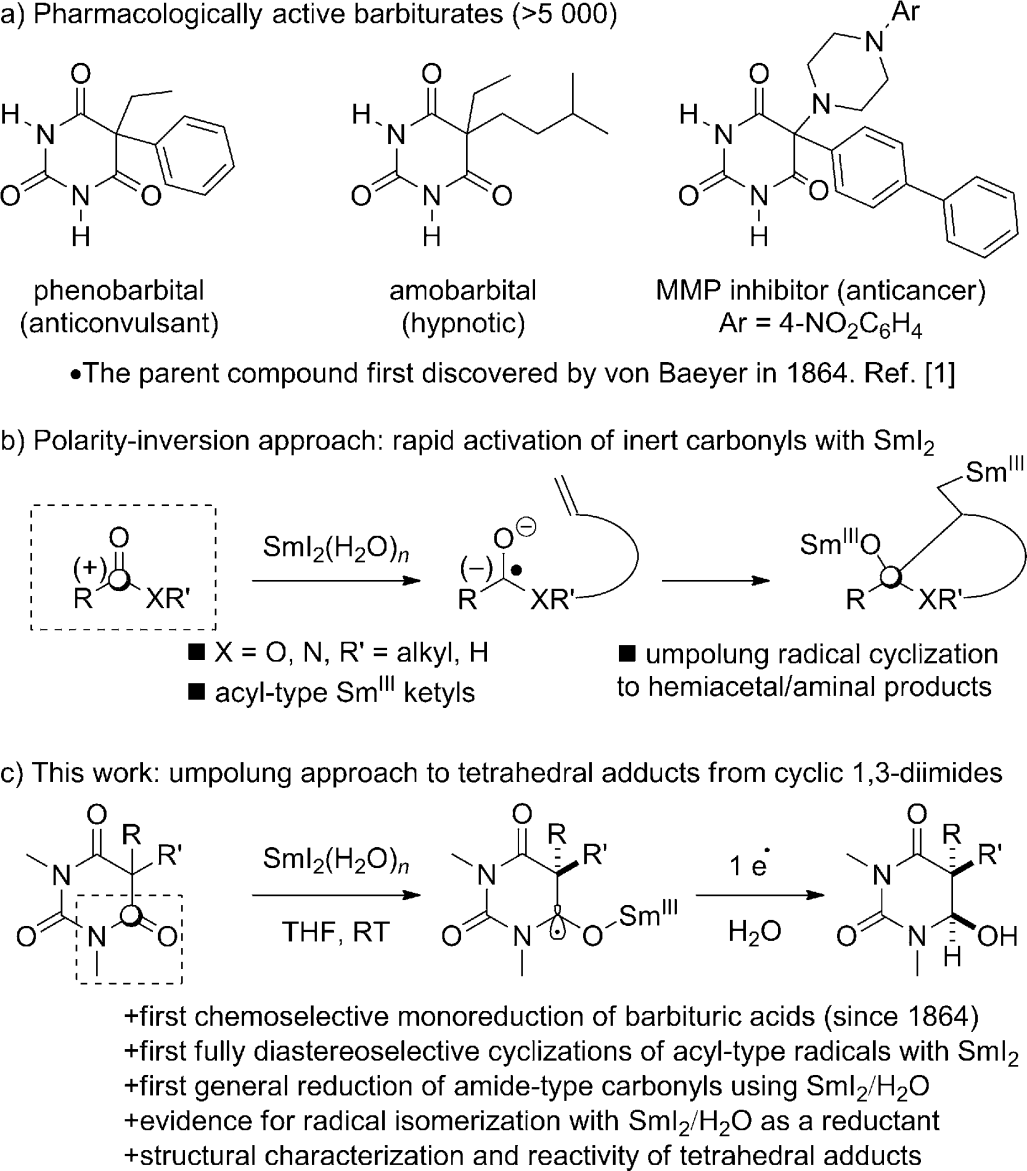
a) Examples of pharmacologically active barbiturates. b) Polarity inversion strategy using SET approach. c) This study.

Single-electron-transfer reactions open up unexplored reaction space charted with chemoselectivity and reactivity levels difficult to access by ionic reaction mechanisms.^6^ The generation of ketyl-type radicals with SmI_2_ is particularly valuable in this regard because of the excellent chemoselectivity imparted by the reagent and the potential to effect polarity reversal of the carbonyl group through a single-electron-reduction event (Figure 1 b).^7^, ^8^ However, the selective reduction of amide carbonyls with SmI_2_ is challenging and no general method to achieve this highly desirable transformation is currently available.^9^

Herein, we demonstrate that the SmI_2_/H_2_O reagent^10^ can perform the selective monoreduction of barbituric acids to the corresponding hemiaminals (Figure 1 c). To our knowledge these are the first general examples of monoreduction of such systems^4^ as well as the reduction of amide-type carbonyls with SmI_2_.^7^, ^8^ The hemiaminal products are analogous to tetrahedral intermediates derived from amide addition reactions.^5^ Moreover, the radical intermediates formed by the one-electron reduction have been utilized in intramolecular additions to alkenes. For the first time in any SmI_2_-mediated cross-couplings of acyl-type radicals,^11^ these additions proceed with full control of diastereoselectivity.^12^ Furthermore, experimental evidence is provided for the isomerization of vinyl radical intermediates under SmI_2_/H_2_O reaction conditions. This discovery opens the door for the use of SmI_2_/H_2_O in cascade reductive processes employing C-centered radicals.^13^ Overall, these studies provide a basis for multiple methodologies to form versatile hemiaminal products (cf. hemiacetals) by a formal amide polarity reversal event.^6^

We hypothesized that single-electron reduction of barbituric acids (cyclic 1,3-diimides) to their respective radical anions could provide a benchmark for the development of a general system for the reduction of a wide range of amide functional groups. We considered that 1) in the barbituric acid system the reduction of one of the imide carbonyls would be enhanced because of its lower energy π*_CO_ orbital, 2) the reduction would be favored by anomeric stabilization of the radical anion intermediate, and 3) the n_N_→π*_CO_ delocalization into the remaining carbonyl in a conformationally locked system would provide access to stable, and unusual, hemiaminal products.

After extensive optimization of the reaction conditions, we determined that barbituric acids are reduced with SmI_2_/H_2_O to the corresponding hemiaminals in good yields and diastereoselectivities (Table 1). Typically, a twofold excess of reagent was used to ensure that the reactions were complete. A wide range of substrates exhibited excellent reactivity, including those with sensitive α protons (entries 1–8), as well as those with sterically hindered quaternary centers (entries 9–11). Importantly, the method tolerates functional groups that are typically reduced under single-electron-transfer conditions, including aromatic rings (entries 4 and 5), ethers (entry 6), trifluoromethyl groups (entry 7), and halides (entry 8). The potential of the reaction to streamline synthetic routes by sequential reductive processes has also been demonstrated (entries 12 and 13). Several products bear close analogy to the pharmacologically active barbiturates (entry 3: amobarbital, entry 10: butalbital).

**Table 1:** Reduction of barbituric acids using SmI_2_.^[a]^

Entry	1,3-Diimide	Product	Yield [%]	d.r. [%]
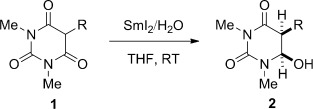				
1	**1 a**, R=*i*Bu	**2 a**	83	88:12
2	**1 b**, R=C_10_H_21_	**2 b**	56	86:14
3	**1 c**, R=(CH_2_)_2_*i*Pr	**2 c**	80	91:9
4	**1 d**, R=(CH_2_)_2_Ph	**2 d**	75	88:12
5	**1 e**, R=(CH_2_)_2_CHMePh	**2 e**	78	85:15
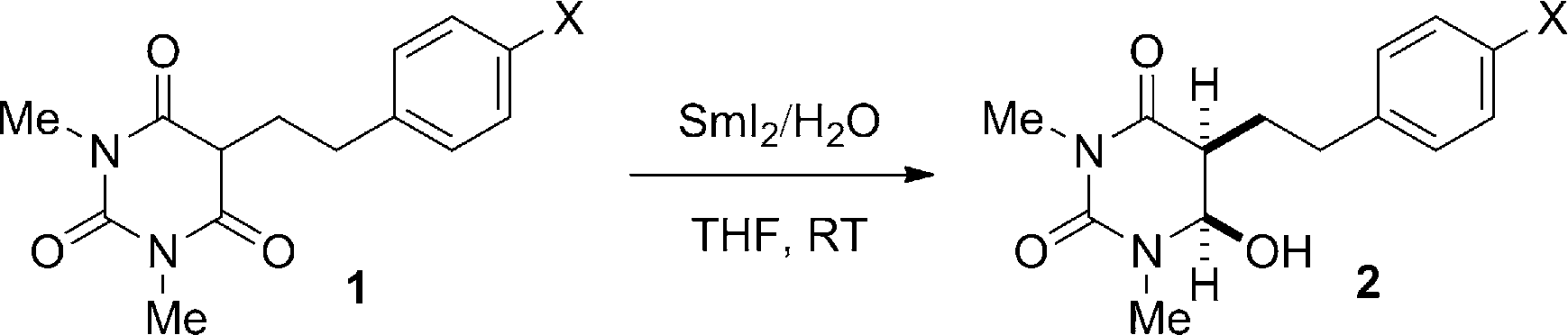				
6	**1 f**, X=MeO	**2 f**	80	88:12
7	**1 g**, X=CF_3_	**2 g**	76	85:15
8	**1 h**, X=Br	**2 h**	67	87:13
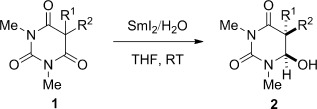				
9	**1 i**, R^1^=Me, R^2^=C_10_H_21_	**2 i**	71	77:23
10	**1 j**, R^1^=Me, R^2^=*i*Bu	**2 j**	50	87:13
11	**1 k**, R^1^,R^2^=-(CH_2_)_2_CH=CH(CH_2_)_2_-	**2 k**	55	–
12^[b]^	**1 l**, R^1^,R^2^= =C(OH)Bn	**2 l**	76	87:13
13^[b]^	**1 m**, R^1^,R^2^= =CH*i*Pr	**2 m**	58	88:12

[a] Reaction conditions: SmI_2_ (4 equiv), THF, H_2_O, 10–60 s. [b] Reaction conditions: SmI_2_ (6–8 equiv), THF, H_2_O, 10–60 s. See the Supporting Information for full experimental details. THF=tetrahydrofuran.

We determined that the use of H_2_O is critical for the observed reactivity, which is in line with the formation of a more thermodynamically powerful reductant required to activate amide-type carbonyls.^14^ No over-reduction is seen, even in the presence of excess reagent. Other SmI_2_-based systems,^15^ including reductants with a higher redox potential than SmI_2_/H_2_O, such as those with alcohols (MeOH, *t*BuOH, EG), Lewis bases (HMPA, Et_3_N), or salts (LiCl) did not provide the desired products.^15^ Competition experiments (see the Supporting Information) illustrate that SmI_2_/H_2_O is selective for cyclic 1,3-diimides over reactive six-membered lactones, thus suggesting that significant levels of selectivity are possible with this reagent system.

To further evaluate the potential of our method, we examined several substrates bearing an unactivated π system tethered to the barbituric acid scaffold (Table 2). A broad range of cyclic 1,3-diimides bearing alkene (entries 1–5) and alkyne (entries 6–9) substitutents underwent efficient radical cyclizations, thus resulting in the formation of bicyclic hemiaminals in good to excellent yields. For the first time in any radical cyclization mediated by SmI_2_/H_2_O, all products were formed with a high degree of stereoisomeric control around the five-membered ring.^11^ We hypothesize that the increased half-life of the acyl-type radical, stabilized by the n_N_→SOMO conjugation (cf. esters),^16^ permits the alkene tether to adopt the lowest energy conformation before the cyclization. This finding bodes well for the development of other SmI_2_-promoted radical cyclizations based on amide bond umpolung.

**Table 2:** Reductive coupling of barbituric acids using SmI_2_.^[a]^

Entry	3	R^1^	R^2^	4	Yield [%]	d.r. [%]
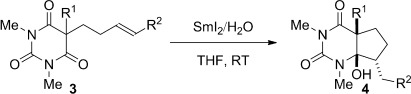						
1	**3 a**	*i*Bu	H	**4 a**	74	>95:5
2	**3 b**	*i*Bu	Ph	**4 b**	58	>95:5
3	**3 c**	*i*Bu	4-MeOC_6_H_4_	**4 c**	59	>95:5
4	**3 d**	C_7_H_13_	Ph	**4 d**	64	>95:5
5	**3 e**	C_4_H_7_	H	**4 e**	55	>95:5
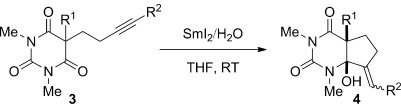						
6	**3 f**	*i*Bu	H	**4 f**	63	>95:5
7	**3 g**	*i*Bu	TMS	**4 g**	66	>95:5^[b]^
8	**3 h**	*i*Bu	Ph	**4 h**	90	63:37^[c]^
9	**3 i**	C_4_H_5_	H	**4 i**	82	>95:5

[a] Reaction conditions: SmI_2_ (6 equiv), THF, H_2_O, 1–15 min. See the Supporting Information for full experimental details. [b] *E* isomer; >95:5 d.r. [c] *Z*/*E* geometry. TMS=trimethylsilyl.

We have carried out preliminary studies to elucidate the mechanism of the reaction (see the Supporting Information for details): 1) The reduction of **1 i** with SmI_2_/D_2_O (>98 % D_1_; *k*_H_/*k*_D_=1.5±0.1)11a suggests that anions are generated and protonated by H_2_O in a series of electron-transfer steps and that proton transfer is not involved in the rate-determining step of the reaction.^17^ 2) Control experiments with a cyclic 1,3-malonamide and DMPU demonstrate that activation of the amide carbonyl facilitates the reaction. 3) Intermolecular competition experiments show that the rate of the reduction can be modified by steric and electronic substitution at the α-carbon atom. 4) Deuterium incorporation and KIE studies on the reductive cyclizations suggest that proton transfer is not involved in the rate-determining step (Figure 2). 5) The reaction of **3 d** to give [D_1_]-**4 d** (1:1 d.r.) demonstrates that the benzylsamarium(III) intermediate is not coordinated to the hydroxy group (Figure 2).11a 6) The reactions of **3 f** and **3 g** indicate inversion of the vinyl radical^18^ under the reduction conditions (Figure 2). 7) A gradual change in diastereoselectivity is observed in the cyclizations of **3 h** at varied concentrations of H_2_O,^10^ additionally suggesting that the carbon-centered radicals do not undergo instantaneous reduction/protonation.^14^ 8) Finally, intermolecular competition experiments indicate that the rate of the cyclization is governed by electronic and steric properties of the π acceptor,^19^ suggesting significant levels of chemoselectivity in these cyclizations.8i

**Figure 2:**
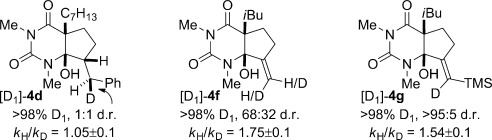
Reductive coupling of barbituric acids **3 d**, **3 f**, and **3 g** using SmI_2_/D_2_O (only products are shown).

A proposed mechanism is shown in Scheme 1. We hypothesize that the kinetic diastereoselectivity in the reduction results from the formation of an organosamarium(III) on the less hindered face of the molecule. This is analogous to the classic reduction of cyclic ketones to equatorial alcohols by related SmI_2_/H_2_O systems.^10^ In the reductive cyclization, the radical anion undergoes an *anti* addition^20^ to give the vinyl radical intermediate, which isomerizes, depending on the steric and electronic preferences of the π acceptor and the reaction conditions. Control experiments (see the Supporting Information) point to the critical role of H_2_O in stabilizing the radical anion^14^ and promoting cyclization (no reaction is observed in the absence or at low concentration of H_2_O as well as with more powerful SmI_2_-based reductants, SmI_2_/LiCl and SmI_2_/HMPA).

**Scheme 1:**
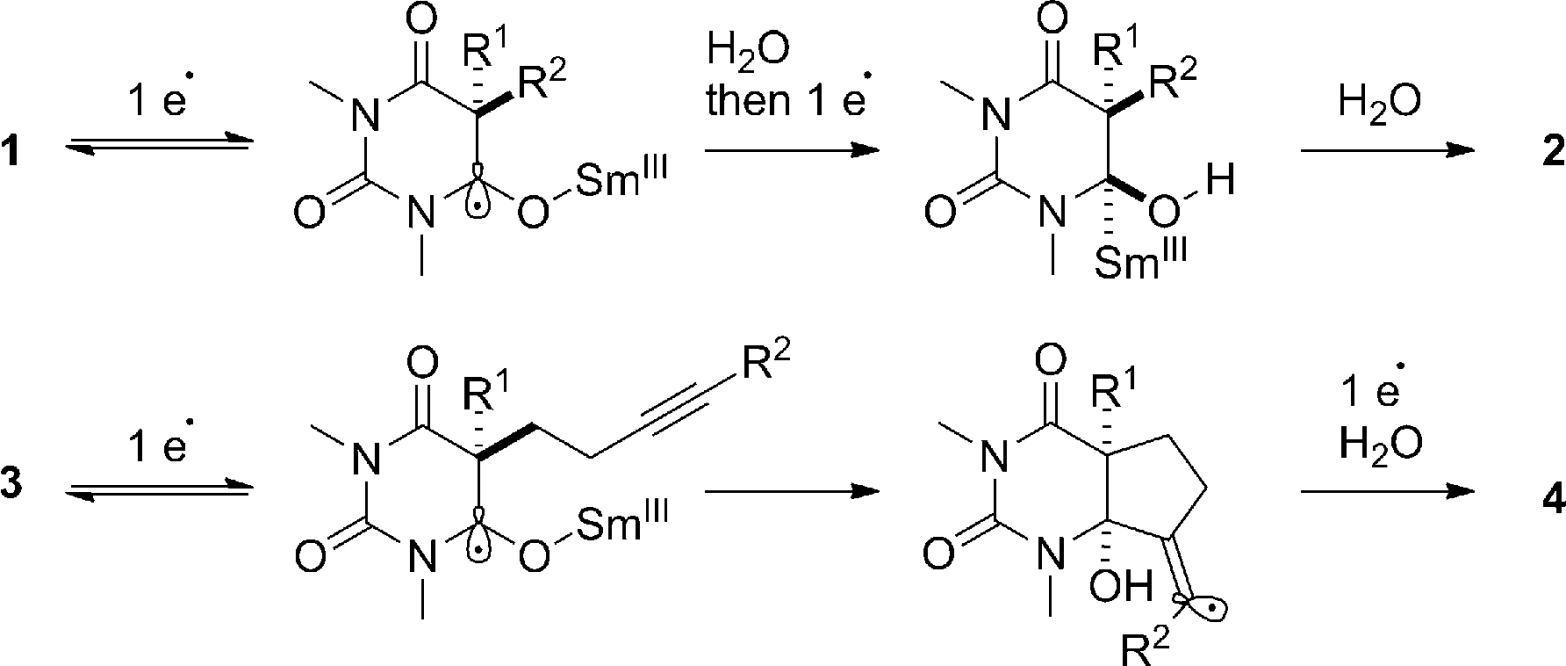
Mechanism of the reduction and cyclization of barbituric acids using SmI_2_/H_2_O.

The α-amino alcohol moiety derived from barbituric acid reduction is stabilized by a nonplanar arrangement of atoms (Figure 3). The X-ray crystal structure of **4 a** reveals that the C1–O1 bond (1.407 Å) is shorter than the average C_sp3_–O bond (1.432 Å),5a whereas the length of N1–C1 bond is 1.466 Å, which corresponds to a typical C_sp3_–N bond (1.469 Å).5a The C1–C4 bond length of 1.552 Å is slightly longer than the average C_sp3_–C_sp3_ bond (1.530 Å).5a The torsion angle between N lp (lp=lone pair) and C1–O1 bond of 57.3° is consistent with the absence of N lp→σ*_C−O_ interactions. However, there is a good overlap between the O1 lp1 and the N1–C1 bond (ca. 172°) and between O1 lp2 and the C1–C4 bonds (ca. 191°). The shortened C1–O1 bond and the elongated C_1_–C_4_ bond are consistent with an anomeric effect resulting from O lp1→σ*_C1–N1_ and O lp2→σ*_C1–C4_ interactions, while the geometry of the N1 atom indicates the beginning of the decomposition of the tetrahedral intermediate by the elimination of N(CO) group. It should be noted that the α-amino alcohol function in this system is stabilized by the reduced N lp→σ*_C1–O1_ conjugation because of the interaction of N lp with the adjacent carbonyl group.

**Figure 3:**
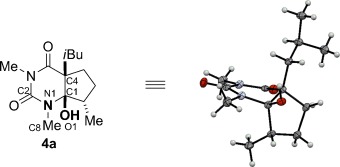
X-ray structure of **4 a**. Selected bond lengths [Å] and angles [°]: N1–C1 1.466, C1–O1 1.407, C1–C4 1.552, C1-H1 0.84, N1–C2 1.354, C2–O2 1.218, N2–C2 1.421; C2-N1-C1-O1 155.1, C8-N1-C1-O1 −40.6, N1-C1-O1-H1 −52.1, C4-C1-O1-H1 70.8, C_1_-N_1_-C_2_-N_2_ −6.9, C2-N2-C3-C4 −14.6, N1-C2-N2-C3 −3.6.^23^

Interestingly, the X-ray structure of the monocyclic analogue **2 f** shows kinetic rather than thermodynamic stability (see the Supporting Information). The torsion angles between N lp and C1–O1 of about 175° and O lp and C1–N1 of about 37° indicate a significant N lp→σ*_C1–O1_ interaction in this system, and the absence of O lp→σ*_C1–N1_ conjugation. The O1-C1-C4-H4 torsion angle of approximately 180° reveals a perfect antiperiplanar arrangement between the α-hydrogen atom and the hydroxy group. These parameters are consistent with the beginning of the decomposition of the α-amino alcohol moiety by the elimination of a hydroxy group to give acyliminium. Overall, these features seem to be characteristic of the α-amino alcohol function stabilized by scaffolding effects in a barbituric acid system and indicate that isolation of a range of analogues can be readily achieved.

Finally, we have preliminary results pertaining to the reactivity of these hemiaminals (Scheme 2). We determined that the alcohol could be directly displaced with a variety of heteroatom and carbon nucleophiles under mild reaction conditions. We also showed that the *N*,*N*-phenobarbital-derived hemiaminal undergoes an unprecedented 1,2-aryl shift (see the Supporting Information). These results bode well for accessing a wide range of biologically active uracil derivatives.^21^

**Scheme 2:**
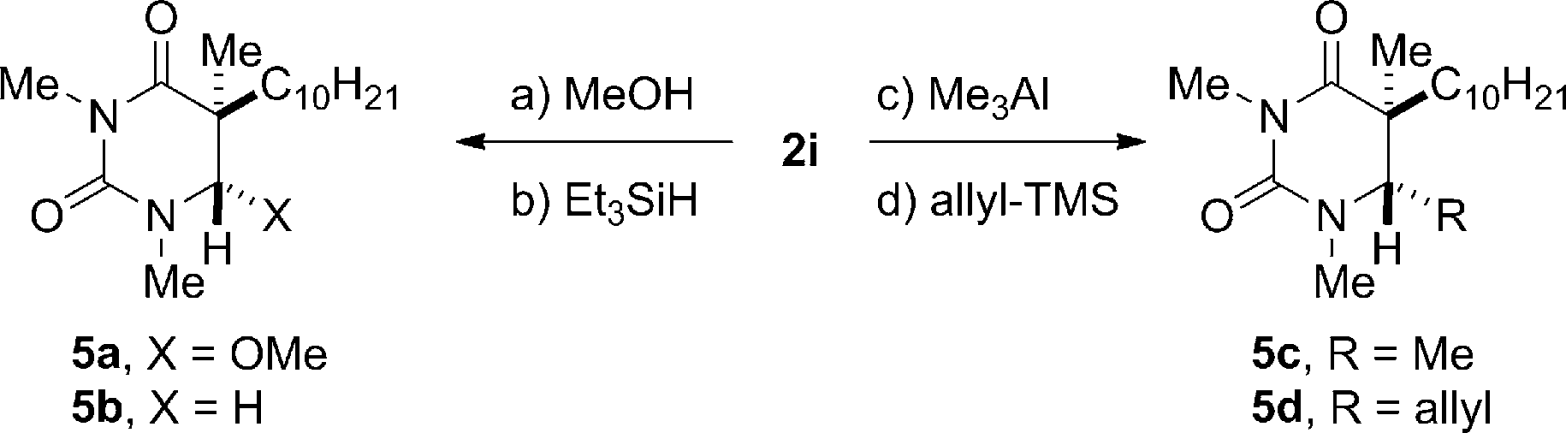
Reactivity of the tetrahedral adducts **2**. Reaction conditions: a) MeOH, HCl, RT, 3 h, 99 %. b) Et_3_SiH, BF_3_⋅Et_2_O, RT, 3 h, 96 %. c) Me_3_Al, RT, 3 h, 78 %. d) allyl-TMS, BF_3_⋅Et_2_O, RT, 2 h, 86 %.

In summary, we have developed the first general method for the monoreduction of barbituric acids since their seminal discovery in 1864 by von Baeyer. This reaction constitutes the first general method for the reduction of amide-type carbonyls using SmI_2_.^22^ The radicals formed by one-electron reduction of the amide bond have been applied in intramolecular additions to alkenes. The cyclic hemiaminal products are analogous to tetrahedral intermediates derived from amide addition reactions and are formed in a formal polarity reversal event. We fully expect that the present work will provide the basis for the synthesis of novel barbituric acid derivatives and will result in the development of an array of modern synthetic methodologies to access reductive amide umpolung by electron transfer events.

## Supplementary material

As a service to our authors and readers, this journal provides supporting information supplied by the authors. Such materials are peer reviewed and may be re-organized for online delivery, but are not copy-edited or typeset. Technical support issues arising from supporting information (other than missing files) should be addressed to the authors.
