# Supplementary material for: Selective Reduction of Barbituric Acids Using SmI2/H2O: Synthesis, Reactivity, and Structural Analysis of Tetrahedral Adducts
Source: Angew Chem Int Ed Engl. 2013 Oct 9;52(48):12559–63. doi: 10.1002/anie.201306484 (PMC4065343; doi:10.1002/anie.201306484)
Supplement: Supplementary file 1 [file anie0052-12559-SD1.pdf]

Angewandte Chemie
Eine Zeitschrift der Gesellschaft Deutscher Chemiker

Supporting Information

© Wiley-VCH 2013

69451 Weinheim, Germany

Selective Reduction of Barbituric Acids Using SmI_2/H_2O: Synthesis, Reactivity, and Structural Analysis of Tetrahedral Adducts**

Michal Szostak, Brice Sautier, Malcolm Spain, Maike Behlendorf, and David J. Procter**

anie_201306484_sm_miscellaneous_information.pdf

Supplementary Information

Table of Contents 1

List of Known Compounds/General Methods 2

Experimental Procedures and Characterization Data 3

- Preparation of Starting Materials 3
- Selective Monoreduction of Cyclic 1,3-Diimides 15
- Reductive Cyclization of Cyclic 1,3-Diimides 24
- Transformations of Cyclic alpha-Hydroxy Carboxamides 33
- Mechanistic Studies 36
  - A) Effect of Additives and Optimization Studies 36
  - B) Studies on Mechanism of Reductive Cyclization 38
  - C) Selectivity of Monoreduction and Cyclization of Cyclic 1,3-Diimides 39
  - D) Deuterium Incorporation and Kinetic Isotope Effect Studies 42
  - E) Evidence for Isomerization of Vinyl Radicals 43
  - F) Additional Selectivity Studies 44

Structural Characterization and Additional Discussion 46

References 48

^{1}H and ^{13}C NMR Spectra 50

Corresponding Author:

Dr. Michal Szostak

Professor David J. Procter

School of Chemistry

University of Manchester

Oxford Road

Manchester, M13 9PL

United Kingdom

General Methods

All experiments involving SmI_2 were performed using standard Schlenk or glovebox techniques under argon or nitrogen atmosphere unless stated otherwise. All solvents were purchased at the highest commercial grade and used as received or after purification by passing through activated alumina columns or distillation from sodium/benzophenone under nitrogen. All solvents were deoxygenated prior to use. All other chemicals were purchased at the highest commercial grade and used as received. Reaction glassware was oven-dried at 140 °C for at least 24 h or flame-dried prior to use, allowed to cool under vacuum and purged with argon (three cycles). Samarium(II) iodide was prepared by standard methods and titrated prior to use.[1-5] ^{1}H NMR and ^{13}C NMR spectra were recorded in $CDCl_3$ on Bruker spectrometers at 300, 400 and 500 MHz (^{1}H NMR) and 75, 100 and 125 MHz (^{13}C NMR). All shifts are reported in parts per million (ppm) relative to residual $CHCl_3$ peak (7.27 and 77.2 ppm, ^{1}H NMR and ^{13}C NMR, respectively). All coupling constants (J) are reported in hertz (Hz). Abbreviations are: s, singlet; d, doublet; t, triplet; q, quartet; br s, broad singlet. All flash chromatography was performed using silica gel, 60 Å, 230−400 mesh. TLC analysis was carried out on aluminium sheets coated with silica gel 60 F254, 0.2 mm thickness. The plates were visualized using a 254 nm ultraviolet lamp or aqueous potassium permanganate solutions.

List of Known Compounds

The following compounds are known: barbituric acids **1a**,[6] **1b**,[7] **1c**,[8] **1d**,[9] **1l**,[10] **1m**,[11] **3e**.[12] The following barbituric acids and derivatives have been prepared according to the known procedures: **1a**,[13] **1b**,[14] **1d**,[15] **1i**,[16] **SI-5**.[17] (*E*)-(4-Bromobut-1-en-1-yl)benzene, (*E*)-1-(4-bromobut-1-en-1-yl)-4-methoxybenzene have been prepared following the procedure by Wong.[18] The intermediate in the synthesis of barbituric acid **3d**, 5-(cyclohexylmethyl)-1,3-dimethylpyrimidine-2,4,6(1*H*,3*H*,5*H*)-trione, have been previously reported.[19] All other substrates have been prepared according to the procedures outlined below. ^{1}H NMR and ^{13}C NMR data are given for all compounds in the Supporting Experimental for characterization purposes. ^{1}H NMR, ^{13}C NMR, IR and HRMS data are reported for all new compounds.

Preparation of Starting Materials

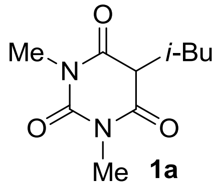

5-Isobutyl-1,3-dimethylpyrimidine-2,4,6(1*H*,3*H*,5*H*)-trione (1a). A 100 mL round-bottomed flask was charged with 1,3-dimethylbarbituric acid (4.68 g, 30 mmol, 1.0 equiv), CH_2Cl_2 (60 mL), isovaleraldehyde (6.48 g, 90 mmol, 3.0 equiv) and BF_3•Et_2O (1.8 mL, 15 mmol, 0.5 equiv), and stirred at room temperature for 18 h. The reaction mixture was diluted with CH_2Cl_2 (200 mL), washed with NaOH (2 *N*, 2 x 50 mL), dried and concentrated to give 1,3-dimethyl-5-(2-methylpropylidene)pyrimidine-2,4,6(1*H*,3*H*,5*H*)-trione which was used in the next step without further purification. To the intermediate α,β-unsaturated barbituric acid (30 mmol) dissolved in absolute EtOH (50 mL), $NaBH_4$ (1.14 g, 30 mmol, 1.0 equiv) was added in portions over 5 minutes at room temperature, and the reaction mixture was stirred at room temperature for 2 h. The reaction mixture was concentrated to dryness under reduced pressure, the residue was taken in H_2O (100 mL) and extracted with CH_2Cl_2 (3 x 100 mL). The aqueous layer was acidified using HCl (conc., 20 mL), extracted with CH_2Cl_2 (3 x 100 mL), the organic layer was dried and concentrated to give the title product as a yellow solid (Mp = 48-50 °C). Yield 84% (5.33 g, 2 steps). ^1H NMR (400 MHz, $CDCl_3$) δ 0.86 (d, J = 6.4 Hz, 6 H), 1.70-1.81 (m, 1 H), 1.87 (t, J = 6.4 Hz, 2 H), 3.23 (s, 6 H), 3.41 (t, J = 6.4 Hz, 1 H); ^{13}C NMR (100 MHz, $CDCl_3$) δ 22.2, 25.7, 28.6, 40.5, 47.8, 151.7, 169.0. Spectroscopic data matched literature values.

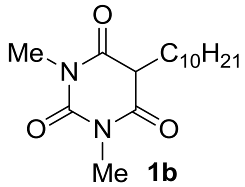

5-Decyl-1,3-dimethylpyrimidine-2,4,6(1*H*,3*H*,5*H*)-trione (1b). A solution of decanoyl chloride (prepared from decanoic acid (1.72 g, 10 mmol, 1.0 equiv) and $SOCl_2$ (15 mL) under reflux for 1 h, followed by removal of $SOCl_2$ under vacuum) was added neat to a solution of 1,3-dimethylbarbituric acid (3.12 g, 20 mmol, 2.0 equiv) in pyridine (25 mL). The resulting mixture was stirred at room temperature for 18 h, acidified with HCl (conc., 25 mL), filtered, washed with water and dried to give 5-decanoyl-1,3-dimethylpyrimidine-2,4,6(1*H*,3*H*,5*H*)-

trione which was used in the next step without further purification. To the intermediate α,β-unsaturated barbituric acid (10 mmol) dissolved in acetic acid (15 mL), $NaCNBH_3$ (1.25 g, 20 mmol, 2.0 equiv) was added in portions over two minutes. The reaction mixture was stirred at room temperature for 2 h, diluted with H_2O (50 mL) and quenched with HCl (conc., 2 mL). After careful removal of HCN by bubbling N_2 through the solution, the reaction mixture was placed at 4 °C overnight, filtered, washed with water and dried in air to give the title compound as a solid (Mp = 53-55 °C). Yield 80% (2.36 g, 2 steps). ^1H NMR (300 MHz, $CDCl_3$) δ 0.86 (t, J = 6.6 Hz, 3 H), 1.10-1.35 (m, 16 H), 2.02-2.16 (m, 2 H), 3.30 (s, 6 H), 3.48 (t, J = 5.1 Hz, 1 H); ^{13}C NMR (75 MHz, $CDCl_3$) δ 14.1, 22.6, 26.0, 28.5, 29.2, 29.2, 29.2, 29.4, 29.5, 31.5, 31.9, 49.1, 151.7, 168.8. Spectroscopic data matched literature values. **Caution! Note that HCN is formed during the reaction and during the work-up. The above procedure should be performed in a well-ventilated fume-cupboard and appropriate precautions should be taken.**

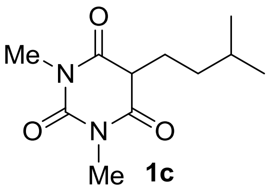

1c

5-Isopentyl-1,3-dimethylpyrimidine-2,4,6(1*H*,3*H*,5*H*)-trione (1c). Prepared according to the procedure described below for **1d** from 1,3-dimethylbarbituric acid (1.56 g, 10 mmol, 1.0 equiv), isovaleric acid (1.8 mL, 15 mmol, 1.5 equiv), DMAP (0.61 g, 5 mmol, 0.5 equiv), DCC (2.27 g, 11 mmol, 1.1 equiv) in CH_2Cl_2 (10 mL) to give the intermediate 5-(1-hydroxy-3-methylbutylidene)-1,3-dimethylpyrimidine-2,4,6(1*H*,3*H*,5*H*)-trione (2.74 g), which was converted directly (2.0 g) into the title compound using $NaCNBH_3$ (1.57 g, 19.7 mmol, 3 equiv) in AcOH (10 mL) for 2 h at room temperature. Colorless oil. Yield 92% (1.7 g, 2 steps). ^1H NMR (400 MHz, $CDCl_3$) δ 0.86 (d, J = 6.6 Hz, 6 H), 1.08 - 1.17 (m, 2 H), 1.46 - 1.58 (m, 1 H), 2.07 - 2.16 (m, 2 H), 3.30 (s, 6 H), 3.48 (t, J = 5.3 Hz, 1 H); ^{13}C NMR (100 MHz, $CDCl_3$) δ 22.2, 27.9, 28.5, 29.3, 34.6, 49.0, 151.6, 168.7. IR (neat) 755, 994, 1087, 1149, 1274, 1320, 1375, 1422, 1446, 1673, 2868, 2954. HRMS calcd for $C_{11}H_{19}N_2O_3$ (M^+ + H) 227.1390, found 227.1384.

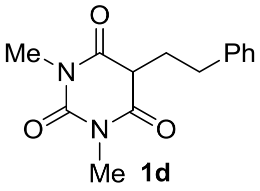

1,3-Dimethyl-5-phenethylpyrimidine-2,4,6(1*H*,3*H*,5*H*)-trione (1d). A 100 mL round-bottomed flask was charged with 1,3-dimethylbarbituric acid (5.0 g, 32 mmol, 1.0 equiv), phenylacetic acid (6.53 g, 48 mmol, 1.5 equiv), DMAP (1.95 g, 16 mmol, 0.5 equiv) and CH_2Cl_2 (60 mL), and cooled to 0 °C. DCC (7.26 g, 35 mmol, 1.1 equiv) was added in portions over 5 minutes and the reaction mixture was stirred for 18 h at room temperature. The reaction mixture was filtered and the precipitated solid was washed with CH_2Cl_2 (1 x 150 mL). The organic layers were combined, washed with HCl (2 *N*, 2 x 40 mL), dried and concentrated. Recrystallization from MeOH gave the intermediate 1,3-dimethyl-5-(2-phenylacetyl)pyrimidine-2,4,6(1*H*,3*H*,5*H*)-trione as a white solid which was used in the next step without further purification. The intermediate α,β-unsaturated barbituric acid (2.74 g, 10 mmol, 1.0 equiv) was reduced according to the procedure described above using $NaCNBH_3$ (1.89 g, 30 mmol, 3.0 equiv) in acetic acid (15 mL) to give the title product as a white solid (Mp = 87-89 °C). Yield 92% (2.29 g, 2 steps). ^{1}H NMR (300 MHz, $CDCl_3$) δ 2.49-2.55 (m, 2 H), 2.72 (t, J = 7.2 Hz, 2 H), 3.24 (s, 6 H), 3.49 (t, J = 5.4 Hz, 1 H), 7.13-7.32 (m, 5 H); ^{13}C NMR (75 MHz, $CDCl_3$) δ 28.5, 31.5, 32.1, 47.9, 126.6, 128.5, 128.7, 139.4, 151.4, 168.4. Spectroscopic data matched literature values.

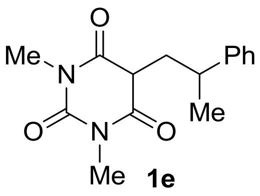

1,3-Dimethyl-5-(2-phenylpropyl)pyrimidine-2,4,6(1*H*,3*H*,5*H*)-trione (1e). Prepared according to the procedure described for **1a** from 1,3-dimethylbarbituric acid (2.34 g, 15 mmol, 1.0 equiv), 2-phenylpropionaldehyde (2.12 g, 15 mmol, 1.05 equiv) and BF_3•Et_2O (0.90 mL, 7.5 mmol, 0.5 equiv) in CH_2Cl_2 (30 mL) to give the intermediate 1,3-dimethyl-5-(2-phenylpropylidene)pyrimidine-2,4,6(1*H*,3*H*,5*H*)-trione which was converted without further purification into the title compound using $NaBH_4$ (1.14 g, 30 mmol, 2.0 equiv) in EtOH (50 mL). Yellow solid (Mp = 45-46 °C). Yield 77% (3.15 g, 2 steps). ^{1}H NMR (300 MHz, $CDCl_3$) δ 1.27 (d, J = 6.6 Hz, 3 H), 2.38-2.60 (m, 2 H), 2.88 (s, 3 H), 2.89-2.98 (m, 1

H), 3.29 (s, 3 H), 3.35 (dd, *J* = 3.0, 7.2 Hz, 1 H), 7.08-7.30 (m, 5 H); ^{13}C NMR (75 MHz, $CDCl_3$) δ 23.3, 28.3, 28.4, 37.4, 37.9, 47.1, 127.0, 127.6, 128.6, 143.8, 151.2, 168.5. IR (neat) 3028, 2960, 1667, 1435, 1418, 1373, 1278, 1127, 1083, 1024, 963, 913, 754 cm^{-1}. HRMS calcd for $C_{15}H_{19}N_2O_3$ (M^+ + H) 275.1391, found 275.1389.

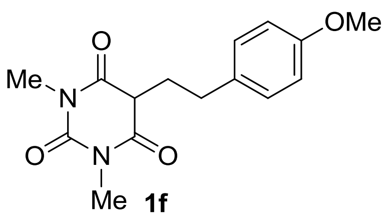

1f

5-(4-Methoxyphenethyl)-1,3-dimethylpyrimidine-2,4,6(1*H*,3*H*,5*H*)-trione (1f). Prepared according to the procedure described above from 1,3-dimethylbarbituric acid (1.56 g, 10 mmol, 1.0 equiv), 4-methoxyphenylacetic acid (2.49 g, 15 mmol, 1.5 equiv), DMAP (0.61 g, 5 mmol, 0.5 equiv), DCC (2.27 g, 11 mmol, 1.1 equiv) in CH_2Cl_2 (10 mL) to give 5-(1-hydroxy-2-(4-methoxyphenyl)ethylidene)-1,3-dimethylpyrimidine-2,4,6(1*H*,3*H*,5*H*)-trione (2.79 g), which was converted directly (2.0 g) into the title compound using $NaCNBH_3$ (1.24 g, 19.7 mmol, 3 equiv) in AcOH (10 mL). Colorless oil. Yield 92% (1.9 g, 2 steps).^{1}H NMR (500 MHz, $CDCl_3$) δ 2.47 (dt, *J* = 6.1, 8.2 Hz, 2 H), 2.61 - 2.68 (m, 2 H), 3.22 (s, 6 H), 3.46 (t, *J* = 5.4 Hz, 1 H), 3.77 (s, 3 H), 6.80 (d, *J* = 8.7 Hz, 2 H), 7.05 (d, *J* = 8.7 Hz, 2 H); ^{13}C NMR (125 MHz, $CDCl_3$) δ 28.4, 31.1, 31.7, 47.7, 55.2, 113.8, 129.7, 131.2, 151.3, 158.1, 168.3. IR (neat) 754, 815, 1034, 1098, 1181, 1245, 1286, 1378, 1421, 1446, 1511, 1676, 2837, 2950, 3206. HRMS calcd for $C_{15}H_{19}N_2O_3$ (M^+ + H) 291.1340, found 291.1348.

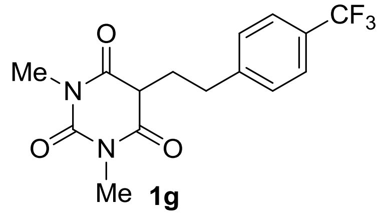

1g

1,3-Dimethyl-5-(4-(trifluoromethyl)phenethyl)pyrimidine-2,4,6(1*H*,3*H*,5*H*)-trione (1g). Prepared according to the procedure described above from 1,3-dimethylbarbituric acid (1.56 g, 10 mmol, 1.0 equiv), 4-(trifluoromethyl)phenylacetic acid (3.06 g, 15 mmol, 1.5 equiv), DMAP (0.61 g, 5 mmol, 0.5 equiv), DCC (2.27 g, 11 mmol, 1.1 equiv) in CH_2Cl_2 (10 mL) to give the intermediate 5-(1-hydroxy-2-(4-(trifluoromethyl)phenyl)ethylidene)-1,3-dimethyl pyrimidine-2,4,6(1*H*,3*H*,5*H*)-trione (2.48 g), which was converted directly (2.0 g) into the title compound using $NaCNBH_3$ (1.10 g, 17.5 mmol, 3 equiv) in AcOH (10 mL). White solid

(Mp = 63-65 °C). Yield 68% (1.8 g, 2 steps).^{1}H NMR (400 MHz, $CDCl_3$) δ 2.47 - 2.54 (m, 2 H), 2.76 - 2.82 (m, 2 H), 3.28 (s, 6 H), 3.49 (t, *J* = 5.4 Hz, 1 H), 7.31 (d, *J* = 8.1 Hz, 2 H), 7.55 (d, *J* = 8.1 Hz, 2 H); ^{13}C NMR (100 MHz, $CDCl_3$) δ 28.6, 31.0, 32.0, 47.8, 124.2 (q, J^1= 272.3 Hz), 125.5 (q, J^3= 3.7 Hz), 128.9 (q, J^2= 32.3 Hz), 129.0, 143.8, 151.3, 168.1. ^{19}F NMR (470 MHz, $CDCl_3$) δ -62.5. IR (neat) 636, 756, 833, 1019, 1065, 1113, 1161, 1284, 1322, 1378, 1422, 1445, 1674, 2864, 2953. HRMS calcd for $C_{15}H_{16}N_2O_3F_3$ (M^+ + H) 329.1108, found 329.1110.

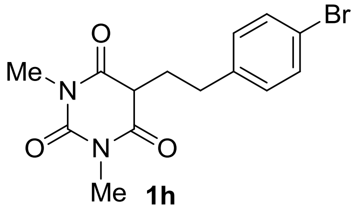

1h

5-(4-Bromophenethyl)-1,3-dimethylpyrimidine-2,4,6(1*H*,3*H*,5*H*)-trione (1h). Prepared according to the procedure described above from 1,3-dimethylbarbituric acid (1.56 g, 10 mmol, 1.0 equiv), 4-bromophenylacetic acid (3.23 g, 15 mmol, 1.5 equiv), DMAP (0.61 g, 5 mmol, 0.5 equiv), DCC (2.27 g, 11 mmol, 1.1 equiv) in CH_2Cl_2 (10 mL) to give 5-(1-hydroxy-2-(4-bromophenyl)ethylidene)-1,3-dimethylpyrimidine-2,4,6(1*H*,3*H*,5*H*)-trione (3.31 g), which was converted directly (3.0 g) into the title compound using $NaCNBH_3$ (1.60 g, 25.5 mmol, 3 equiv) in AcOH (10 mL). White solid (Mp = 52-54 °C). Yield 87% (2.7 g, 2 steps). ^{1}H NMR (500 MHz, $CDCl_3$) δ 2.42 - 2.49 (m, 2 H), 2.63 - 2.70 (m, 2 H), 3.25 (s, 6 H), 3.46 (t, *J* = 5.4 Hz, 1 H), 7.05 (d, *J* = 8.4 Hz, 2 H), 7.39 (d, *J* = 8.4 Hz, 2 H); ^{13}C NMR (125 MHz, $CDCl_3$) δ 28.5, 31.2, 31.6, 47.7, 120.3, 130.4, 131.5, 138.5, 151.3, 168.1. IR (neat) 635, 709, 755, 817, 1011, 1101, 1379, 1425, 1445, 1674, 2948, 3226, 3402. HRMS calcd for $C_{14}H_{15}N_2O_3BrNa$ (M^+ + Na) 361.0158, found 361.0143.

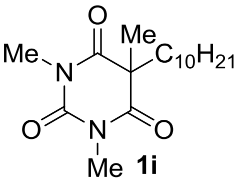

1i

5-Decyl-1,3,5-trimethylpyrimidine-2,4,6(1H,3H,5H)-trione (1i). A 25 mL round-bottomed flask was charged with 5-decyl-1,3-dimethylpyrimidine-2,4,6(1*H*,3*H*,5*H*)-trione (1.0 g, 3.3 mmol, 1.0 equiv), acetone (5 mL), iodomethane (2 mL, 30 mmol, 10 equiv) and K_2CO_3 (2.0 g, 15 mmol, 4.5 equiv), and refluxed at 50 °C for 18 h. The reaction mixture was cooled to

room temperature, diluted with H_2O (50 mL) and extracted with Et_2O (3 x 50 mL). The organic layers were combined, washed with NaOH (2 *N*, 2 x 50 mL), dried and concentrated to give the title compound as a yellow solid (Mp = 51-51 °C). Yield 60% (0.61 g). ^1H NMR (300 MHz, $CDCl_3$) δ 0.79 (t, *J* = 6.6 Hz, 3 H), 0.93-1.06 (m, 2 H), 1.06-1.28 (m, 14 H), 1.44 (s, 3 H), 1.85-1.91 (m, 2 H), 3.24 (s, 6 H); ^{13}C NMR (75 MHz, $CDCl_3$) δ 14.0, 22.6, 24.4, 25.2, 28.5, 29.1, 29.2, 29.3, 29.4, 29.4, 31.8, 40.3, 51.6, 151.2, 172.3. IR (neat) 2924, 2854, 1674, 1444, 1380, 1278, 1068, 913, 755, 731 cm^{-1}. HRMS calcd for $C_{17}H_{30}N_2O_3Na$ (M^+ + Na) 333.2149, found 333.2153.

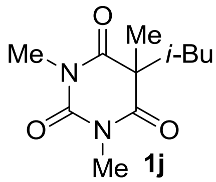

1j

5-Isobutyl-1,3,5-trimethylpyrimidine-2,4,6(1*H*,3*H*,5*H*)-trione (1j). Prepared according to the procedure described above for **1i** using 5-isobutyl-1,3-dimethylpyrimidine-2,4,6(1*H*,3*H*,5*H*)-trione (0.70 g, 3.30 mmol, 1.0 equiv), iodomethane (2 mL, 30 mmol, 10 equiv) and K_2CO_3 (2.0 g, 15 mmol, 4.5 equiv) in acetone (5 mL) at 50 °C for 18 h to give the title compound as a low melting solid (mp = 38-39 °C). Yield 85% (0.63 g). ^1H NMR (300 MHz, $CDCl_3$) δ 0.81 (d, *J* = 6.9 Hz, 6 H), 1.54 (s, 3 H), 1.45-1.56 (m, 1 H), 2.02 (d, *J* = 6.9 Hz, 2 H), 3.34 (s, 6 H); ^{13}C NMR (75 MHz, $CDCl_3$) δ 23.0, 25.5, 26.8, 28.5, 48.1, 50.5, 151.1, 172.3. IR (neat) 1681, 1448, 1381, 1357, 1287, 1199, 1096, 1067, 978, 754 cm^{-1}. HRMS calcd for $C_{11}H_{19}N_2O_3$ (M^+ + H) 227.1390, found 227.1387.

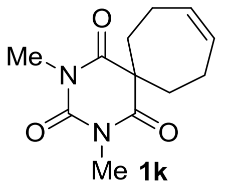

1k

2,4-Dimethyl-2,4-diazaspiro[5.6]dodec-9-ene-1,3,5-trione (1k). A 100 mL flask was charged with 5,5-di(but-3-en-1-yl)-1,3-dimethylpyrimidine-2,4,6(1*H*,3*H*,5*H*)-trione (0.5 g, 1.9 mmol, 1 equiv), Grubbs I catalyst (156 mg, 0.2 mmol, 0.1 equiv) and toluene (60 mL) and stirred for 24 h at 90 °C to give after purification by chromatography using EtOAc/hexanes (10/90) the title compound as a white solid (Mp = 118-120 °C). Yield 70% (0.31 g). ^1H NMR (400 MHz, $CDCl_3$) δ 2.22 - 2.31 (m, 4 H), 2.41 - 2.51 (m, 4 H), 3.29 (s, 6 H), 5.65 (t, *J* = 2.6 Hz, 2 H); ^{13}C NMR (100 MHz, $CDCl_3$) δ 24.9, 28.9, 34.6, 53.8, 129.9, 151.3, 172.6. IR (neat)

631, 707, 756, 931, 1023, 1065, 1109, 1236, 1271, 1371, 1420, 1455, 2856, 2926, 3017. HRMS calcd for $C_{12}H_{16}N_2O_3$ (M^+) 236.1155, found 236.1157.

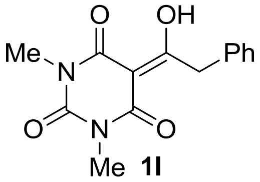

1l

5-(1-Hydroxy-2-phenylethylidene)-1,3-dimethylpyrimidine-2,4,6(1*H*,3*H*,5*H*)-trione (1l). Prepared according to the procedure described above from 1,3-dimethylbarbituric acid (5.0 g, 32 mmol, 1.0 equiv), phenylacetic acid (6.53 g, 48 mmol, 1.5 equiv), DMAP (1.95 g, 16 mmol, 0.5 equiv) and DCC (7.26 g, 35 mmol, 1.1 equiv) in CH_2Cl_2 (60 mL) for 18 h at room temperature to give the title compound as a white solid (Mp = 82-84 °C). Yield 75% (6.58 g). ^{1}H NMR (300 MHz, $CDCl_3$) δ 3.39 (s, 6 H), 4.50 (s, 2 H), 7.22-7.35 (m, 3 H), 7.37-7.42 (m, 2 H); ^{13}C NMR (75 MHz, $CDCl_3$) δ 27.9, 28.1, 41.8, 95.3, 127.3, 128.6, 129.7, 134.5, 150.2, 160.8, 169.9, 196.2. Spectroscopic data matched literature values.

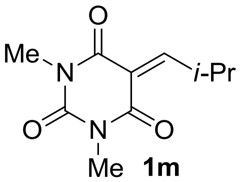

1m

1,3-Dimethyl-5-(2-methylpropylidene)pyrimidine-2,4,6(1H,3H,5H)-trione (1m). Prepared according to the procedure described above from 1,3-dimethylbarbituric acid (4.68 g, 30 mmol, 1.0 equiv), isovaleraldehyde (6.48 g, 90 mmol, 3.0 equiv) and BF_3•Et_2O (1.8 mL, 15 mmol, 0.5 equiv) in CH_2Cl_2 (60 mL) at room temperature for 18 h to give the title compound as white solid (mp = 39-40 °C). Yield 99% (6.2 g). ^{1}H NMR (400 MHz, $CDCl_3$) δ 1.08 (d, *J* = 6.4 Hz, 6 H), 3.28 (s, 3 H), 3.29 (s, 3 H), 3.85-3.97 (m, 1 H), 7.69 (d, J = 10.0 Hz, 1 H); ^{13}C NMR (75 MHz, $CDCl_3$) δ 21.4, 28.1, 28.7, 29.1, 118.4, 151.3, 160.9, 161.7, 173.7. Spectroscopic data matched literature values.

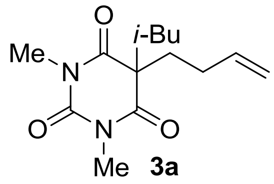

5-(But-3-en-1-yl)-5-isobutyl-1,3-dimethylpyrimidine-2,4,6(1*H*,3*H*,5*H*)-trione (3a). Prepared according to the procedure described above for **1i** from 5-isobutyl-1,3-dimethylpyrimidine-2,4,6(1*H*,3*H*,5*H*)-trione (0.77 g, 3.6 mmol, 1.0 equiv), 4-bromobut-1-ene (0.73 g, 5.4 mmol, 1.5 equiv) and K_2CO_3 (1.0 g, 7.2 mmol, 2.0 equiv) in acetone (20 mL) for 24 h at 60 °C to give after purification by chromatography using EtOAc/hexanes (1/99-10/90) the title compound as a colorless oil. Yield 51% (0.49 g). R_f (20% EtOAc/hexanes) = 0.73. ^{1}H NMR (400 MHz, $CDCl_3$) δ 0.71 (d, J = 6.8 Hz, 6 H), 1.32-1.44 (m, 1 H), 1.82 (q, J = 6.8 Hz, 2 H), 1.92 (d, J = 6.8 Hz, 2 H), 2.02 (t, J = 7.6 Hz, 2 H), 3.24 (s, 6 H), 4.80-4.87 (m, 2 H), 5.49-5.61 (m, 1 H); ^{13}C NMR (100 MHz, $CDCl_3$) δ 23.1, 25.5, 28.4, 29.6, 40.8, 48.8, 55.0, 115.6, 136.5, 151.1, 171.9. IR (neat) 2960, 2932, 1674, 1444, 1417, 1379, 1349, 1318, 1083, 917, 787 cm^{-1}. HRMS calcd for $C_{14}H_{22}N_2O_3Na$ (M^+ + Na) 289.1523, found 289.1516.

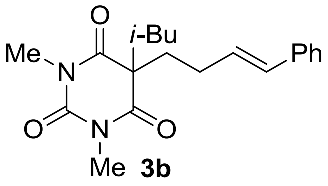

(*E*)-5-Isobutyl-1,3-dimethyl-5-(4-phenylbut-3-en-1-yl)pyrimidine-2,4,6(1*H*,3*H*,5*H*)-trione (3b). Prepared according to the procedure described above for **1i** using 5-isobutyl-1,3-dimethylpyrimidine-2,4,6(1*H*,3*H*,5*H*)-trione (0.50 g, 2.4 mmol, 1.0 equiv), (*E*)-(4-bromobut-1-en-1-yl)benzene (0.60 g, 2.8 mmol, 1.2 equiv, prepared according to the procedure described by Wong[18]) and K_2CO_3 (0.65 g, 4.7 mmol, 2.0 equiv) in acetone (5 mL) for 18 h at 60 °C to give after purification by chromatography using EtOAc/hexanes (2/98-10/90) the title compound as a colorless oil. Yield 53% (0.43 g). R_f (10% EtOAc/hexanes) = 0.43. ^{1}H NMR (300 MHz, $CDCl_3$) δ 0.79 (d, J = 6.9 Hz, 6 H), 1.39-1.53 (m, 1 H), 2.00 (d, J = 6.6 Hz, 2 H), 2.10 (q, J = 6.6 Hz, 2 H), 2.18-2.25 (m, 2 H), 3.24 (s, 6 H), 6.02 (dt, J = 6.9, 15.6 Hz, 1 H), 6.25 (d, J = 15.9 Hz, 1 H), 7.18-7.34 (m, 5 H); ^{13}C NMR (75 MHz, $CDCl_3$) δ 23.2, 25.5, 28.5, 29.2, 40.6, 49.5, 55.1, 126.1, 127.4, 128.2, 128.6, 131.3, 136.8, 151.1, 172.1. IR (neat) 2960, 2932, 2873, 1672, 1495, 1438, 1417, 1378, 1352, 1316, 1276, 1119, 1078, 967, 745, 692 cm^{-1}. HRMS calcd for $C_{20}H_{27}N_2O_3$ (M^+ + H) 343.2016, found 343.2018.

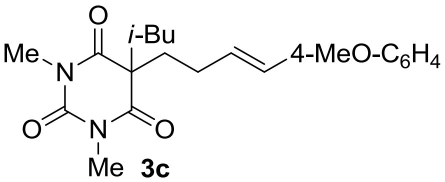

3c

(*E*)-5-Isobutyl-5-(4-(4-methoxyphenyl)but-3-en-1-yl)-1,3-dimethylpyrimidine-2,4,6 (1*H*,3*H*,5*H*)-trione (3c). Prepared according to the procedure described above for **1i** using 5-isobutyl-1,3-dimethylpyrimidine-2,4,6(1*H*,3*H*,5*H*)-trione (0.30 g, 1.4 mmol, 1.0 equiv), (*E*)-1-(4-bromobut-1-en-1-yl)-4-methoxybenzene (0.41 g, 1.7 mmol, 1.2 equiv, prepared according to the procedure described by Wong[18]) and K_2CO_3 (0.39 g, 2.8 mmol, 2.0 equiv) in acetone (5 mL) for 18 h at 60 °C to give after purification by chromatography using EtOAc/hexanes (2/98-10/90) the title compound as a colorless oil. Yield 71% (0.37 g). R_f (10% EtOAc/hexanes) = 0.26. ^{1}H NMR (300 MHz, $CDCl_3$) δ 0.78 (d, *J* = 6.9 Hz, 6 H), 1.38-1.52 (m, 1 H), 2.00 (d, J = 6.6 Hz, 2 H), 2.08 (q, *J* = 6.9 Hz, 2 H), 2.20 (t, *J* = 6.6 Hz, 2 H), 3.23 (s, 6 H), 3.81 (s, 3 H), 5.86 (dt, *J* = 6.9, 15.6 Hz, 1 H), 6.17 (d, *J* = 15.9 Hz, 1 H), 6.82 (d, *J* = 8.7 Hz, 2 H), 7.21 (d, *J* = 8.7 Hz, 2 H); ^{13}C NMR (75 MHz, $CDCl_3$) δ 23.2, 25.5, 28.5, 29.3, 40.8, 49.5, 55.1, 55.3, 114.0, 126.0, 127.2, 129.6, 130.6, 151.1, 159.1, 172.1. IR (neat) 2959, 2932, 1672, 1510, 1439, 1379, 1351, 1244, 1174, 1033, 967, 837, 755 cm^{-1}. HRMS calcd for $C_{21}H_{29}N_2O_4$ (M^+ + H) 373.2122, found 373.2129.

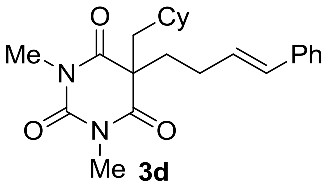

3d

(*E*)-5-(Cyclohexylmethyl)-1,3-dimethyl-5-(4-phenylbut-3-en-1-yl)pyrimidine-2,4,6 (1*H*,3*H*,5*H*)-trione (3d). Prepared according to the procedure described above for **1i** using 5-(cyclohexylmethyl)-1,3-dimethylpyrimidine-2,4,6(1*H*,3*H*,5*H*)-trione (0.50 g, 2.0 mmol, 1.0 equiv (prepared from 1,3-dimethylbarbituric acid and cyclohexanecarboxaldehyde as described above in 73% yield, 5.48 g, 2 steps, solid, mp = 85-87 °C.[19] ^{1}H NMR (300 MHz, $CDCl_3$) δ 0.88 (qd, *J* = 3.0, 11.7 Hz, 2 H), 1.03-1.26 (m, 3 H), 1.35-1.51 (m, 1 H), 1.53-1.72 (m, 5 H), 1.89 (t, *J* = 6.9 Hz, 2 H), 3.24 (s, 6 H), 3.46 (t, *J* = 6.3 Hz, 1 H); ^{13}C NMR (75 MHz, $CDCl_3$) δ 25.9, 26.2, 28.5, 32.8, 34.8, 38.9, 47.0, 151.7, 169.0. Spectroscopic data matched literature values), (*E*)-(4-bromobut-1-en-1-yl)benzene (0.63 g, 3.0 mmol, 1.5 equiv, prepared according to the procedure described by Wong[18]) in acetone (5 mL) for 18 h at 60 °C to give after purification by chromatography using EtOAc/hexanes (15/75) the title

compound as a white solid (mp = 98-99 °C). Yield 66% (0.48 g). R_f (15% EtOAc/hexanes) = 0.48. ^1H NMR (500 MHz, $CDCl_3$) δ 0.81 (qd, J = 3.0, 12.0 Hz, 2 H), 0.93-1.09 (m, 4 H), 1.32 (d, J = 12.5 Hz, 2 H), 1.45-1.56 (m, 3 H), 1.90 (d, J = 6.5 Hz, 2 H), 2.00 (q, J = 7.0 Hz, 2 H), 2.11 (t, J = 7.5 Hz, 2 H), 3.16 (s, 6 H), 5.93 (dt, J = 7.0, 15.5 Hz, 1 H), 6.15 (d, J = 15.5 Hz, 1 H), 7.10-7.14 (m, 1 H), 7.17-7.23 (m, 4 H); ^{13}C NMR (125 MHz, $CDCl_3$) δ 25.9, 26.0, 28.5, 29.2, 33.7, 34.8, 40.7, 48.0, 54.8, 126.1, 127.4, 128.2, 128.6, 131.2, 136.8, 151.4, 172.1. IR (neat) 2997, 2945, 2850, 1669, 1492, 1411, 1261, 1240, 1200, 1054, 987, 954, 753, 744, 693 cm^{-1}. HRMS calcd for $C_{23}H_{31}N_2O_3$ (M^+ + H) 383.2330, found 383.2321.

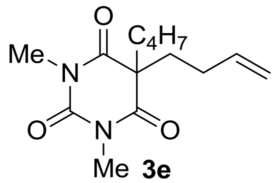

3e

5,5-Di(but-3-en-1-yl)-1,3-dimethylpyrimidine-2,4,6(1*H*,3*H*,5*H*)-trione (3e). A 50 mL flask was charged with 5,5-di(but-3-en-1-yl)-1,3-dimethylpyrimidine-2,4,6(1*H*,3*H*,5*H*)-trione (1.6 g, 10 mmol, 1 equiv), 4-bromo-1-butene (1.2 mL, 12 mmol, 0.6 equiv), tetrabutylammonium bisulfate (34 mg, 0.1 mmol, 0.01 equiv), K_2CO_3 (1.8 g, 20 mmol, 2 equiv) and DMF (25 mL) and stirred at 80 °C for 18 h. The organic layer was separated by filtration, concentrated and purified by chromatography to give the title compound as a white solid (Mp 43-45 °C). Yield 78% (1.2 g). ^1H NMR (400 MHz, $CDCl_3$) δ 1.85 - 1.95 (m, 4 H), 2.07 - 2.15 (m, 4 H), 3.28 (s, 6 H), 4.85 - 4.93 (m, 4 H), 5.55 - 5.68 (m, 2 H); ^{13}C NMR (100 MHz, $CDCl_3$) δ 28.3, 29.8, 39.3, 55.4, 115.7, 136.5, 151.0, 171.7. IR (neat) 639, 753, 921, 999, 1036, 1083, 1171, 1258, 1281, 1334, 1382, 1416, 1445, 2928, 2955. HRMS calcd for $C_{14}H_{20}N_2O_3$ (M^+) 264.1468, found 264.1461.

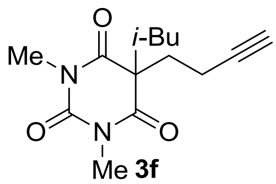

3f

5-(But-3-yn-1-yl)-5-isobutyl-1,3-dimethylpyrimidine-2,4,6(1*H*,3*H*,5*H*)-trione (3f). Prepared according to the procedure described above for **1i** using 5-isobutyl-1,3-dimethylpyrimidine-2,4,6(1*H*,3*H*,5*H*)-trione (0.64 g, 3.0 mmol, 1.0 equiv), 4-bromo-1-butyne (0.42 mL, 4.5 mmol, 1.5 equiv) and K_2CO_3 (0.83 g, 6.0 mmol, 2.0 equiv) in acetone (15 mL) for 21 h at 60 °C to give after purification by chromatography using EtOAc/hexanes (10/90)

the title compound as a colorless oil. Yield 46% (0.31 g). ^{1}H NMR (400 MHz, $CDCl_3$) δ 0.77 (d, *J* = 6.8 Hz, 6 H), 1.40 - 1.52 (m, 1 H), 1.91 (t, *J* = 2.5 Hz, 1 H), 1.95 (d, *J* = 6.8 Hz, 2 H), 2.11 (td, *J* = 2.5, 6.3 Hz, 2 H), 2.17 - 2.24 (m, 2 H), 3.30 (s, 6 H); ^{13}C NMR (100 MHz, $CDCl_3$) δ 14.5, 23.2, 25.3, 28.5, 38.8, 49.3, 54.6, 69.8, 81.4, 151.1, 171.5. IR (neat) 645, 755, 1052, 1089, 1119, 1159, 1219, 1291, 1352, 1380, 1442, 1676, 2872, 2960, 3275. HRMS calcd for $C_{14}H_{21}N_2O_3$ (M^+ + H) 265.1547, found 265.1543.

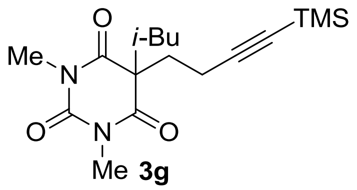

3g

5-Isobutyl-1,3-dimethyl-5-(4-(trimethylsilyl)but-3-yn-1-yl)pyrimidine-2,4,6(1*H*,3*H*,5*H*)-trione (3g). Prepared according to the procedure described above for **1i** using 5-isobutyl-1,3-dimethylpyrimidine-2,4,6(1*H*,3*H*,5*H*)-trione (0.64 g, 3.0 mmol, 1.0 equiv), (4-bromobut-1-yn-1-yl)trimethylsilane (1.0 g, 4.5 mmol, 1.5 equiv) and K_2CO_3 (0.83 g, 6.0 mmol, 2.0 equiv) in acetone (15 mL) for 21 h at 60 °C to give after purification by chromatography using EtOAc/hexanes (5/95) the title compound as a white solid (Mp 58-60 °C). Yield 19% (0.17 g). ^{1}H NMR (500 MHz, $CDCl_3$) δ 0.11 (s, 9 H), 0.78 (d, *J* = 6.9 Hz, 6 H), 1.42 - 1.51 (m, 1 H), 1.96 (d, *J* = 6.9 Hz, 2 H), 2.11 - 2.16 (m, 2 H), 2.18 - 2.24 (m, 2 H), 3.33 (s, 6 H); ^{13}C NMR (125 MHz, $CDCl_3$) δ 0.0, 15.9, 23.2, 25.4, 28.6, 39.0, 49.0, 54.7, 86.2, 103.9, 151.1, 171.4. IR (neat) 641, 700, 757, 842, 1048, 1088, 1159, 1252, 1277, 1351, 1379, 1443, 2959. HRMS calcd for $C_{17}H_{29}N_2O_3Si$ (M^+ + H) 337.1942, found 337.1953.

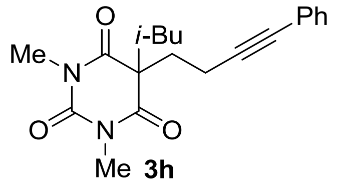

3h

5-Isobutyl-1,3-dimethyl-5-(4-phenylbut-3-yn-1-yl)pyrimidine-2,4,6(1*H*,3*H*,5*H*)-trione (3h). A 10 mL vial was charged with **3f** (106 mg, 0.4 mmol, 1 equiv), iodobenzene (0.1 mL, 0.8 mmol, 2 equiv), tetrakis(triphenylphosphine)palladium(0) (9 mg, 8 μmol, 0.02 equiv), copper(I) iodide (1 mg, 4 μmol, 0.01 equiv), diisopropylamine (0.4 mL) and THF (1 mL), and stirred at 60 °C for 8 h. The reaction mixture was cooled down to room temperature, filtered through celite and purified by chromatography to give the title compound as a colorless oil. Yield 91% (123 mg). ^{1}H NMR (400 MHz, $CDCl_3$) δ 0.83 (d, *J* = 6.6 Hz, 6 H),

1.45 - 1.57 (m, 1 H), 2.03 (d, *J* = 6.6 Hz, 2 H), 2.28 - 2.44 (m, 4 H), 3.28 (s, 6 H), 7.29 - 7.33 (m, 3 H), 7.34 - 7.38 (m, 2 H); ^{13}C NMR (100 MHz, $CDCl_3$) δ 15.6, 23.3, 25.4, 28.6, 38.9, 49.7, 54.8, 82.4, 86.8, 122.9, 128.1, 128.3, 131.7, 151.2, 171.7. IR (neat) 692, 755, 916, 1054, 1088, 1157, 1295, 1351, 1379, 1440, 1676, 2959. HRMS calcd for $C_{20}H_{25}N_2O_3$ (M^+ + H) 341.1860, found 341.1871.

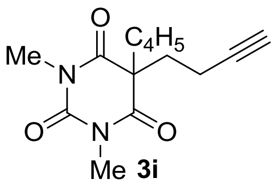

3i

5,5-Di(but-3-yn-1-yl)-1,3-dimethylpyrimidine-2,4,6(1*H*,3*H*,5*H*)-trione (3i). Prepared according to the procedure described above for **3e** using 5,5-di(but-3-en-1-yl)-1,3-dimethylpyrimidine-2,4,6(1*H*,3*H*,5*H*)-trione (1.6 g, 10 mmol, 1 equiv), 4-bromo-1-butyne (1.1 mL, 12 mmol, 0.6 equiv), tetrabutylammonium bisulfate (34 mg, 0.1 mmol, 0.01 equiv) and K_2CO_3 (1.8 g, 20 mmol, 2 equiv) in DMF (25mL) to give after purification by chromatography the title compound as a white solid (Mp 67-69 °C). Yield 42% (0.7 g). ^{1}H NMR (400 MHz, $CDCl_3$) δ 1.93 (t, *J* = 2.8 Hz, 2 H), 2.11 - 2.20 (m, 4 H), 2.21 - 2.30 (m, 4 H), 3.31 (s, 6 H); ^{13}C NMR (100 MHz, $CDCl_3$) δ 14.5, 28.5, 37.9, 54.4, 70.0, 81.3, 151.0, 170.9. IR (neat) 642, 753, 913, 1062, 1127, 1284, 1348, 1382, 1440, 1671, 2947, 3278. HRMS calcd for $C_{14}H_{17}O_3N_2$ (M^+ + H) 261.1234, found 261.1229.

Selective Monoreduction of Cyclic 1,3-Diimides using SmI_2–H_2O

General procedure for monoreduction of cyclic 1,3-diimides using SmI_2–H_2O. An oven-dried vial containing a stir bar was charged with a cyclic 1,3-diimide (1 equiv), placed under a positive pressure of argon, and subjected to three evacuation/backfilling cycles under high vacuum. THF (typically, 2.0 mL) and water (typically, 1000 equiv, ca. 1:1 THF/H_2O v/v) were added, followed by a rapid injection of SmI_2 (in THF, typically 4 equiv) with vigorous stirring. After the specified time (typically, 10-60 s), the reaction was quenched by bubbling air through the reaction mixture, diluted with CH_2Cl_2 (30 mL) and HCl (0.1 *N*, 20 mL), H_2O (20 mL) or $NaHCO_3$ (2% in H_2O, 20 mL). The aqueous layer was extracted with CH_2Cl_2 (3 x 20 mL), organic layers were combined, dried over Na_2SO_4, filtered and concentrated. The sample was analyzed by ^{1}H NMR ($CDCl_3$, C_6D_6 or $CD_3C(O)CD_3$) and/or GC-MS (neat) to determine the product distribution and diastereoselectivity from the crude reaction mixture. The crude product was purified by chromatography on silica gel, concentrated under reduced pressure and stored neat or as a solution in acetone. All compounds have been prepared as racemates.

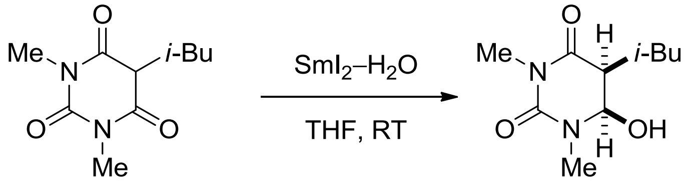

1a **2a**

(5*S*,6*R*)-6-Hydroxy-5-isobutyl-1,3-dimethyldihydropyrimidine-2,4(1*H*,3*H*)-dione (2a). According to the general procedure, the reaction of **1a** (0.10 mmol), SmI_2 (0.30 mmol, 3 equiv, 5.5 mL, 0.055 M) and H_2O (1.8 mL, 1000 equiv) for 10 s afforded after purification by chromatography (1/1 EtOAc/hexanes) the title compound as a colorless oil. Yield 83%. Dr = 88:12 (crude), 88:12 (purified). Stereochemistry of the major diastereoisomer was determined by 2 D NMR experiments and confirmed by an X-ray analysis of a derivative. Rf (50% EtOAc/hexanes) = 0.53. ^{1}H NMR (500 MHz, C_6D_6) δ (major diastereoisomer) 0.88 (d, *J* = 6.5 Hz, 3 H), 0.96 (d, *J* = 6.5 Hz, 3 H), 1.54-1.60 (m, 1 H), 1.69-1.78 (m, 1 H), 2.85-2.14 (m, 1 H), 2.23-2.27 (m, 1 H), 2.87 (s, 3 H), 3.20 (d, *J* = 6.0 Hz, 1 H), 3.32 (s, 3 H), 4.25 (dd, *J* = 4.5, 5.0 Hz, 1 H); (minor, diagnostic peaks only) 0.82 (d, *J* = 6.5 Hz, 3 H), 0.92 (d, *J* = 6.5 Hz, 3 H), 2.93 (s, 3 H), 3.31 (s, 3 H), 3.53 (d, *J* = 4.5 Hz, 1 H), 4.19 (d, *J* = 2.0 Hz, 1 H); ^{13}C NMR (75 MHz, C_6D_6) δ (major diastereoisomer) 21.9, 23.3, 25.3, 27.7, 34.3, 34.8, 44.3,

80.5, 153.6, 170.4; (minor, diagnostic peaks only) 22.2, 22.4, 25.7, 27.5, 34.6, 39.0, 47.6, 82.6, 171.6. IR (neat) 3392, 2956, 2871, 1710, 1650, 1468, 1421, 1295, 1143, 1094, 1055, 1031, 1000, 911, 793 cm^{-1}. HRMS calcd for $C_{10}H_{18}N_2O_3Na$ (M^+ + Na) 237.1210, found 237.1217.

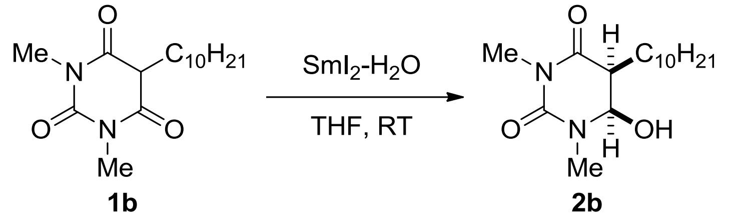

(5*S*,6*R*)-5-Decyl-6-hydroxy-1,3-dimethyldihydropyrimidine-2,4(1*H*,3*H*)-dione (2b). According to the general procedure, the reaction of **1b** (0.10 mmol), SmI_2 (0.30 mmol, 3 equiv, 6.7 mL, 0.045 M) and H_2O (1.8 mL, 1000 equiv) for 10 s afforded after purification by chromatography (1/1 EtOAc/hexanes) the title compound as a colorless oil. Yield 56%. Dr = 86:14 (crude), 92:8 (after purification). Stereochemistry of the major diastereoisomer was determined by 2 D NMR experiments and confirmed by an X-ray analysis of a derivative. Rf (50% EtOAc/hexanes) = 0.47. ^1H NMR (500 MHz, $CD_3C(O)CD_3$) δ (major diastereoisomer) 0.82 (t, J = 7.0 Hz, 3 H), 1.18-1.31 (m, 14 H), 1.36-1.43 (m, 2 H), 1.45-1.53 (m, 1 H), 1.89-1.97 (m, 1 H), 2.67-2.71 (m, 1 H), 2.99 (s, 3 H), 3.00 (s, 3 H), 4.94 (dd, J = 3.5, 5.0 Hz, 1 H), 5.44 (dd, J = 1.0, 5.5 Hz, 1 H); (minor, diagnostic peaks only) 2.59 (td, J = 2.0, 7.5 Hz, 1 H), 3.00 (s, 3 H), 3.00 (s, 3 H), 4.82 (dd, J = 1.5, 4.5 Hz, 1 H), 5.51 (d, J = 5.0 Hz, 1 H); ^{13}C NMR (75 MHz, $CD_3C(O)CD_3$) δ (major diastereoisomer) 14.4, 23.3, 26.4, 27.5, 27.5, 30.1, 30.3, 30.4, 30.5, 32.6, 34.4, 47.0, 80.5, 154.2, 171.5; (minor, diagnostic peaks only) 34.7, 50.2, 80.4, 172.1. IR (neat) 3310, 1920, 2851, 1709, 1652, 1494, 1469, 1418, 1301, 1219, 1156, 1090, 1003, 914, 763 cm^{-1}. HRMS calcd for $C_{16}H_{29}N_2O_2$ (M^+ + H – H_2O) 281.2224, found 281.2222.

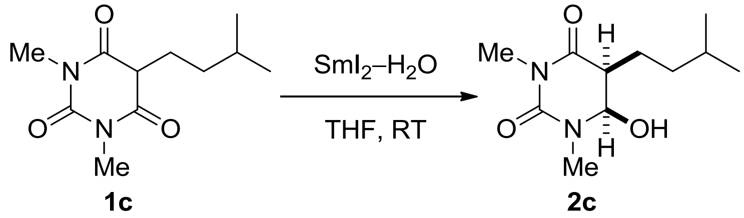

(5*S*,6*R*)-6-Hydroxy-5-isopentyl-1,3-dimethyldihydropyrimidine-2,4(1*H*,3*H*)-dione (2c). According to the general procedure, the reaction of **1c** (0.10 mmol), SmI_2 (0.30 mmol, 3

equiv, 5.5 mL, 0.055 M) and H_2O (1.8 mL, 1000 equiv) for 10 s afforded after purification by chromatography (1/1 EtOAc/hexanes) the title compound as a white solid (Mp 117-119 °C). Yield 80% (^{1}H NMR analysis vs. internal standard). Analytical sample was purified for characterization purposes. Dr = 91:9 (crude), 94:6 (purified). Stereochemistry of the major diastereoisomer was determined by 2 D NMR experiments and confirmed by an X-ray analysis of a derivative. ^{1}H NMR (500 MHz, $CD_3C(O)CD_3$) δ (major diastereoisomer) 0.90 (d, *J* = 6.6 Hz, 3 H), 0.92 (d, *J* = 6.6 Hz, 3 H), 1.27 - 1.38 (m, 2 H), 1.49 - 1.62 (m, 2 H), 1.97 - 2.04 (m, 1 H), 2.71 (td, *J* = 3.6, 8.8 Hz, 1 H), 3.05 (s, 3 H), 3.06 (s, 3 H), 4.99 (dd, *J* = 3.6, 5.5 Hz, 1 H), 5.45 (dd, *J* = 0.8, 5.5 Hz, 1 H); (minor, diagnostic peaks only) δ 0.86 (d, *J* = 6.5 Hz, 3 H), 0.88 (d, *J* = 6.5 Hz, 3 H), 2.61 (td, *J* = 1.9, 7.4 Hz, 1 H), 3.05 (s, 3 H), 3.06 (s, 3 H), 4.88 (dd, *J* = 1.9, 4.7 Hz, 1 H), 5.51 (d, *J* = 4.7 Hz, 1 H); ^{13}C NMR (125 MHz, $CD_3C(O)CD_3$) δ (major diastereoisomer) 22.8, 23.1, 24.4, 27.6, 29.0, 34.4, 36.7, 47.3, 80.5, 154.2, 171.6; (minor, diagnostic peaks only) δ 22.7, 27.4, 28.6, 34.7, 36.8, 50.4, 82.7, 155.1, 172.2. IR (neat) 762, 903, 969, 1004, 1055, 1095, 1146, 1222, 1296, 1379, 1423, 1483, 1653, 1710, 2870, 2954, 3393. HRMS calcd for $C_{11}H_{20}N_2O_3Na$ (M^+ + Na) 251.1366, found 251.1369.

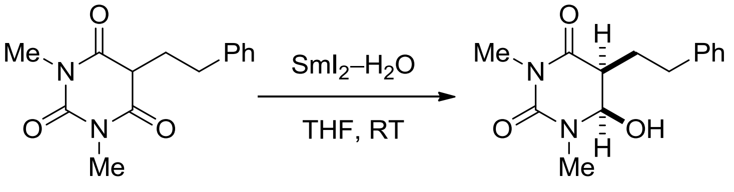

(5*S*,6*R*)-6-Hydroxy-1,3-dimethyl-5-phenethyldihydropyrimidine-2,4(1*H*,3*H*)-dione (2d). According to the general procedure, the reaction of **1d** (0.10 mmol), SmI_2 (0.30 mmol, 3 equiv, 3.5 mL, 0.087 M) and H_2O (1.8 mL, 1000 equiv) for 10 s afforded after purification by chromatography (1/1 EtOAc/hexanes) the title compound as a colorless oil. Yield 75%. Dr = 88:12 (crude), 89:11 (after purification). Rf (60% EtOAc/hexanes) = 0.46. ^{1}H NMR (300 MHz, $CD_3C(O)CD_3$) δ (major diastereoisomer) 1.80-1.98 (m, 1 H), 2.26-2.45 (m, 1 H), 2.68-2.87 (m, 3 H), 3.06 (s, 3 H), 3.08 (s, 3 H), 5.09 (dd, *J* = 3.6, 4.8 Hz, 1 H), 5.60 (d, *J* = 5.1 Hz, 1 H), 7.16-7.34 (m, 5 H); (minor, diagnostic peaks only) 3.06 (s, 3 H), 3.10 (s, 3 H), 3.92 (dd, *J* = 1.8, 4.2 Hz, 1 H); ^{13}C NMR (75 MHz, $CD_3C(O)CD_3$) δ (major diastereoisomer) 27.5, 28.6, 33.6, 34.4, 46.2, 80.6, 126.7, 129.2, 129.3, 143.0, 154.1, 171.4; (minor, diagnostic peaks only) 46.2, 80.5, 126.9, 129.4, 142.2. IR (neat) 3391, 1943, 1712, 1652, 1486, 1454, 1295, 1117, 1079, 752 cm^{-1}. HRMS calcd for $C_{14}H_{18}N_2O_3Na$ (M^+ + Na) 285.1210, found 285.1204.

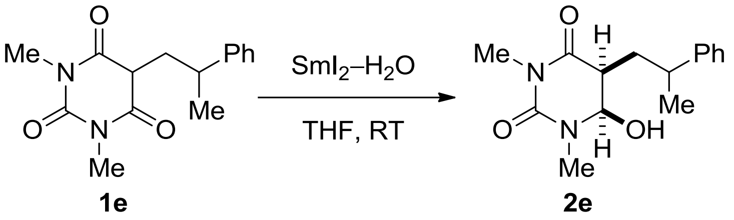

(5*S*,6*R*)-6-Hydroxy-1,3-dimethyl-5-(2-phenylpropyl)dihydropyrimidine-2,4(1*H*,3*H*)-dione (2e). According to the general procedure, the reaction of **1e** (0.10 mmol), SmI_2 (0.40 mmol, 4 equiv, 4.6 mL, 0.087 M) and H_2O (1.8 mL, 1000 equiv) for 60 s afforded after purification by chromatography (EtOAc) the title compound as colorless oil. Yield 78%. Dr = 58:42 (at the Me) (83:17 major, 86:14, minor; total 85:15) (crude); dr = 53:47 (at the Me) (89:11 major, 86:14, minor, total 88:12) (purified). Rf (60% EtOAc/hexanes) = 0.50. ^{1}H NMR (500 MHz, $CD_3C(O)CD_3$) δ (major diastereoisomers) 1.14 (d, *J* = 6.5 Hz, 3 H), 1.15 (d, *J* = 7.0 Hz, 3 H), 1.61-1.68 (m, 1 H), 1.71-1.77 (m, 1 H), 2.10-2.17 (m, 1 H), 2.22-2.26 (m, 1 H), 2.27-2.32 (m, 1 H), 2.50-2.54 (m, 1 H), 2.82 (s, 3 H), 2.82-2.85 (m, 1 H), 2.86 (s, 3 H), 2.89 (s, 3 H), 2.93 (s, 3 H), 2.95-3.00 (m, 1 H), 4.64 (dd, *J* = 3.5, 5.0 Hz, 1 H), 4.89 (dd, *J* = 3.0, 5.0 Hz, 1 H), 5.41 (dd, *J* = 1.0, 5.5 Hz, 1 H), 5.46 (dd, *J* = 1.0, 5.5 Hz, 1 H), 7.03-7.19 (m, 5 H); (minor, diagnostic peaks only) 1.10 (d, *J* = 6.5 Hz, 3 H), 2.83 (s, 3 H), 2.90 (s, 3 H), 2.92 (s, 3 H), 4.52 (dd, *J* = 2.0, 4.5 Hz, 1 H), 4.82 (dd, *J* = 2.0, 5.0 Hz, 1 H), 5.31 (d, *J* = 4.0 Hz, 1 H); ^{13}C NMR (75 MHz, $CD_3C(O)CD_3$) δ (major diastereoisomers) 22.5, 23.7, 27.5, 27.6, 34.1, 34.3, 34.5, 35.6, 37.1, 38.2, 45.1, 45.3, 80.1, 81.2, 127.0, 127.2, 127.8, 127.9, 129.4, 129.5, 147.1, 148.0, 153.9, 154.0, 171.3, 171.6. IR (neat) 3392, 2960, 1715, 1657, 1487, 1423, 1297, 1196, 1053, 992, 763 cm^{-1}. HRMS calcd for $C_{15}H_{20}N_2O_3Na$ (M^+ + Na) 299.1366, found 299.1380.

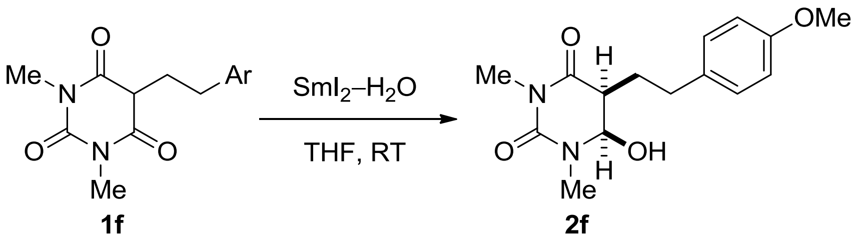

(5*S*,6*R*)-6-Hydroxy-5-(4-methoxyphenethyl)-1,3-dimethyldihydropyrimidine-2,4(1*H*,3*H*)-dione (2f). According to the general procedure, the reaction of **1f** (0.10 mmol), SmI_2 (0.30 mmol, 3 equiv, 5.5 mL, 0.055 M) and H_2O (1.8 mL, 1000 equiv) for 10 s afforded after purification by chromatography (1/1 EtOAc/hexanes) the title compound as a white solid (Mp 123-125 °C). Yield 80% (^{1}H NMR analysis vs. internal standard). Analytical

sample was purified for characterization purposes. Dr = 88:12 (crude), 88:12 (purified). Stereochemistry of the major diastereoisomer determined by an X-ray analysis (re-crystallization from acetone). ^{1}H NMR (500 MHz, $CD_3C(O)CD_3$) δ 1.77 - 1.87 (m, 1 H), 2.24 - 2.33 (m, 1 H), 2.70 - 2.79 (m, 3 H), 3.04 (s, 3 H), 3.06 (s, 3 H), 3.75 (s, 3 H), 5.05 (dd, *J* = 3.8, 5.4 Hz, 1 H), 5.57 (dd, *J* = 0.9, 5.4 Hz, 1 H), 6.85 (d, *J* = 8.6 Hz, 2 H), 7.16 (d, *J* = 8.6 Hz, 2 H); ^{13}C NMR (125 MHz, $CD_3C(O)CD_3$) δ 27.6, 28.8, 32.7, 34.4, 46.3, 55.5, 80.6, 114.7, 130.2, 134.8, 154.1, 159.0, 171.5. IR (neat) 761, 827, 1033, 1120, 1178, 1244, 1296, 1424, 1484, 1510, 1657, 1711, 2850, 2926, 3373. HRMS calcd for $C_{15}H_{21}N_2O_4$ (M^+ + H) 293.1496, found 293.1501.

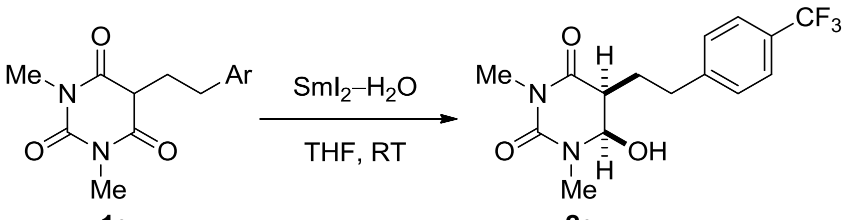

(5*S*,6*R*)-6-Hydroxy-1,3-dimethyl-5-(4-(trifluoromethyl)phenethyl)dihydropyrimidine-2,4(1*H*,3*H*)-dione (2g). According to the general procedure, the reaction of **1g** (0.10 mmol), SmI_2 (0.30 mmol, 3 equiv, 5.5 mL, 0.055 M) and H_2O (1.8 mL, 1000 equiv) for 10 s afforded after purification by chromatography (1/1 EtOAc/hexanes) the title compound as a colorless oil. Yield 76% (^{1}H NMR analysis vs. internal standard). Analytical sample was for purified for characterization purposes. Dr = 85:15 (crude), 76:24 (purified). ^{1}H NMR (500 MHz, $CD_3C(O)CD_3$) δ 1.84 - 1.93 (m, 1 H), 2.29 - 2.39 (m, 1 H), 2.81 - 2.85 (m, 1 H), 2.93 (t, *J* = 8.2 Hz, 2 H), 3.05 (s, 3 H), 3.07 (s, 3 H), 5.10 (dd, *J* = 3.8, 5.4 Hz, 1 H), 5.61 (dd, *J* = 0.6, 5.4 Hz, 1 H), 7.50 (d, *J* = 7.9 Hz, 2 H), 7.64 (d, *J* = 7.9 Hz, 2 H); ^{13}C NMR (125 MHz, $CD_3C(O)CD_3$) δ 27.7, 28.5, 33.5, 34.4, 46.4, 80.6, 125.6 (q, J^1= 272.5 Hz), 126.2 (q, J^3= 3.6 Hz), 128.6 (q, J^2= 32.7 Hz), 130.1, 148.0, 154.0, 171.3. ^{19}F NMR (470 MHz, $CD_3C(O)CD_3$) δ -62.4. IR (neat) 763, 830, 1017, 1066, 1120, 1163, 1325, 1423, 1485, 1660, 1713, 2856, 2927, 3391. HRMS calcd for $C_{15}H_{17}O_3N_2F_3Na$ (M^+ + Na) 353.1083, found 353.1083.

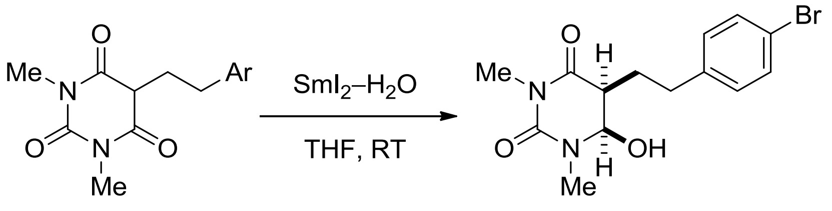

(5*S*,6*R*)-5-(4-Bromophenethyl)-6-hydroxy-1,3-dimethyldihydropyrimidine-2,4(1*H*,3*H*)-dione (2h). According to the general procedure, the reaction of **1h** (0.10 mmol), SmI_2 (0.30 mmol, 3 equiv, 5.5 mL, 0.055 M) and H_2O (1.8 mL, 1000 equiv) for 10 s afforded after purification by chromatography (1/1 EtOAc/hexanes) the title compound as a white solid (Mp 116-118 °C). Yield 67%. Dr = 87:13 (crude), 87:13 (purified). ^{1}H NMR (400 MHz, $CD_3C(O)CD_3$) δ 1.65 - 1.77 (m, 1 H), 2.11 - 2.22 (m, 1 H), 2.64 - 2.71 (m, 3 H), 2.91 (s, 3 H), 2.93 (s, 3 H), 4.94 (dd, *J* = 3.8, 5.3 Hz, 1 H), 5.43 - 5.48 (m, 1 H), 7.10 (d, *J* = 8.5 Hz, 2 H), 7.33 (d, *J* = 8.5 Hz, 2 H);^{13}C NMR (100 MHz, $CD_3C(O)CD_3$) δ 27.6, 28.5, 33.0, 34.4, 46.3, 80.6, 120.0, 131.4, 132.3, 142.4, 153.9, 171.3. IR (neat) 762, 817, 1011, 1067, 1120, 1296, 1425, 1485, 1658, 1710, 2851, 2962, 3363. HRMS calcd for $C_{14}H_{18}N_2O_3Br$ (M^+ + H) 341.0495, found 341.0505.

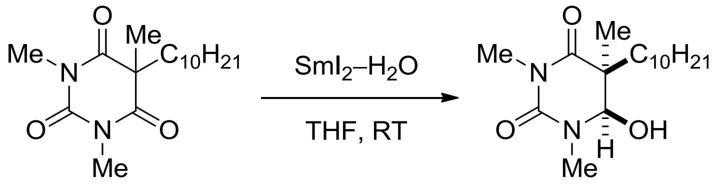

(5*S*,6*R*)-5-Decyl-6-hydroxy-1,3,5-trimethyldihydropyrimidine-2,4(1*H*,3*H*)-dione (2i). According to the general procedure, the reaction of **1i** (0.10 mmol), SmI_2 (0.40 mmol, 4 equiv, 13.3 mL, 0.033 M) and H_2O (1.8 mL, 1000 equiv) for 60 s afforded after purification by chromatography (1/1 EtOAc/hexanes) the title compound as a colorless oil. Yield 71%. Dr = 77:23 (crude), >95:5 (after purification). The major diastereoisomer can be partially separated by chromatography on silica gel. Stereochemistry of the major diastereoisomer was determined by 2 D NMR experiments and subsequently confirmed by an X-ray analysis of a derivative. Rf (50% EtOAc/hexanes) = 0.60. ^{1}H NMR (500 MHz, C_6D_6) δ (major diastereoisomer) 0.97 (s, 3 H), 1.04 (t, *J* = 7.0 Hz, 3 H), 1.27-1.48 (m, 16 H), 2.03 (td, *J* = 5.0, 12.5 Hz, 1 H), 2.13 (td, *J* = 4.0, 12.5 Hz, 1 H), 2.91 (s, 3 H), 3.17 (d, *J* = 5.0 Hz, 1 H), 3.36 (s, 3 H), 4.09 (d, *J* = 5.0 Hz, 1 H); ^{13}C NMR (125 MHz, C_6D_6) δ (major diastereoisomer) 14.4, 20.7, 23.1, 23.1, 27.9, 29.9, 30.1, 30.2, 30.7, 32.3, 32.9, 34.6, 46.2, 85.2, 153.2, 174.5.

IR (neat) 3446, 3014, 1923, 2852, 1739, 1717, 1652, 1365, 1295, 1228, 1216, 1205, 1050, 898, 765 cm^{-1}. HRMS calcd for $C_{17}H_{31}N_2O_2$ (M^+ + H – H_2O) 295.2381, found 295.2380.

(5*S*,6*S*)-5-Decyl-6-hydroxy(6-*D*^{1})-1,3,5-trimethyldihydropyrimidine-2,4(1*H*,3*H*)-dione (2i-*D*^{1}). According to the above procedure reaction of **1i** (0.10 mmol), SmI_2 (0.40 mmol, 3 equiv, 2.7 mL, 0.11 M) and D_2O (1.8 mL, 1000 equiv) for 60 s afforded after purification by chromatography (1/1 EtOAc/hexanes) the title compound with >98% D_1 incorporation as a colorless oil. Yield 57%. Dr = 77:23 (crude), >95:5 (after purification). The major diastereoisomer can be partially separated by chromatography on silica gel. ^{1}H NMR (500 MHz, $CD_3C(O)CD_3$) δ (major diastereoisomer) 0.88 (t, J = 6.9 Hz, 3 H), 1.15 (s, 3 H), 1.22 - 1.36 (m, 15 H), 1.36 - 1.46 (m, 2 H), 1.70 - 1.82 (m, 2 H), 3.05 (s, 3 H), 3.05 (s, 3 H), 5.48 (s, 1 H); (minor, diagnostic peaks only) 0.87 (t, J = 6.9 Hz, 3 H), 1.23 (s, 3 H), 1.42 - 1.50 (m, 1 H), 1.50 - 1.58 (m, 1 H), 5.47 (s, 1 H); ^{13}C NMR (125 MHz, $CD_3C(O)CD_3$) δ (major diastereoisomer) 14.4, 21.1, 23.3, 23.4, 27.7, 30.1, 30.2, 30.2, 30.4, 31.2, 32.7, 33.5, 34.6, 46.6, 84.8 (t, J = 23.6 Hz), 153.7, 175.6; (minor, diagnostic peaks only) 14.4, 17.8, 23.4, 24.7, 27.7, 32.7, 34.6, 37.5, 48.0, 153.7, 174.4. IR (neat) 764, 847, 965, 1064, 1316, 1384, 1417, 1469, 1656, 1711, 2854, 2924, 3398. HRMS calcd for $C_{17}H_{30}DN_2O_2$ (M^+ − OH) 296.2443, found 296.2432. Kinetic isotope effect was determined by reacting **1i** (0.10 mmol), SmI_2 (0.40 mmol, 3 equiv, 2.7 mL, 0.11 M) and D_2O/H_2O (1:1, 1.8 mL, 1000 equiv) for 60 s at rt, followed by standard work-up to give the title compound with 40.1% D^1 incorporation as determined by ^{1}H NMR (500 MHz) analysis (k_H/k_D = 1.49±0.1).

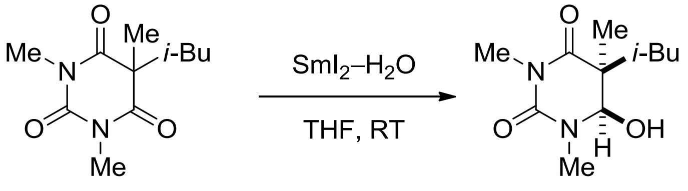

(5*S*,6*R*)-6-Hydroxy-5-isobutyl-1,3,5-trimethyldihydropyrimidine-2,4(1*H*,3*H*)-dione (2j). According to the general procedure, the reaction of **1j** (0.10 mmol), SmI_2 (0.40 mmol, 4 equiv, 13.3 mL, 0.033 M) and H_2O (1.8 mL, 1000 equiv) for 60 s afforded after purification by chromatography (1/1 EtOAc/hexanes) the title compound as a colorless oil. Yield 50%. Dr = 87:13 (crude), >95:5 (after purification). The major diastereoisomer can be partially separated by chromatography on silica gel. Stereochemistry of the major diastereoisomer was determined by 2 D NMR experiments. Rf (50% EtOAc/hexanes) = 0.40. ^{1}H NMR (500 MHz,

C_6D_6) δ (major diastereoisomer) 0.80 (s, 3 H), 0.83 (d, *J* = 6.5 Hz, 3 H), 0.84 (d, *J* = 7.0 Hz, 3 H), 1.46-1.55 (m, 1 H), 1.72 (dd, *J* = 5.5, 14.5 Hz, 1 H), 1.91 (dd, *J* = 6.0, 15.0 Hz, 1 H), 2.71 (s, 3 H), 3.06 (d, *J* = 5.0 Hz, 1 H), 3.13 (s, 3 H), 3.95 (d, *J* = 5.5 Hz, 1 H); (minor, diagnostic peaks only) 0.62 (d, *J* = 6.5 Hz, 3 H), 0.64 (d, *J* = 7.0 Hz, 3 H), 2.73 (s, 3 H), 3.14 (s, 3 H), 3.71 (d, *J* = 4.5 Hz, 1 H); ^{13}C NMR (125 MHz, C_6D_6) δ (major diastereoisomer) 21.0, 23.4, 25.2, 25.5, 27.9, 34.4, 40.8, 46.8, 83.4, 153.0, 174.2; (minor, diagnostic peaks only) 86.4. IR (neat) 3394, 2956, 1711, 1651, 1485, 1419, 1294, 1176, 1049, 975, 913, 765, 747 cm^{-1}. HRMS calcd for $C_{11}H_{20}N_2O_3Na$ (M^+ + Na) 251.1367, found 251.1361. Note that in $CDCl_3$ slow epimerization to the thermodynamic diastereoisomer was observed over time ($t_{1/2}$ = 2 months). ^1H NMR (400 MHz, $CDCl_3$) δ (major diastereoisomer) 0.93 (d, *J* = 6.8 Hz, 3 H), 0.96 (d, *J* = 6.4 Hz, 3 H), 1.17 (s, 3 H), 1.59-1.65 (m, 1 H), 1.66-1.76 (m, 1 H), 1.81 (dd, *J* = 6.0, 14.4 Hz, 1 H), 2.93 (d, *J* = 4.8 Hz, 1 H), 3.07 (s, 3 H), 3.09 (s, 3 H), 4.55 (d, *J* = 4.4 Hz, 1 H); (minor diastereoisomer) 0.72 (d, *J* = 6.4 Hz, 3 H), 0.85 (d, *J* = 6.8 Hz, 3 H), 1.21 (s, 3 H), 1.37 (dd, *J* = 7.2, 14.0 Hz, 1 H), 1.45-1.55 (m, 1 H), 1.65-1.73 (m, 1 H), 3.07 (m, 1 H), 3.10 (s, 3 H), 3.15 (s, 3 H), 3.98 (s, 1 H); ^{13}C NMR (100 MHz, $CDCl_3$) δ (major diastereoisomer) 21.2, 23.3, 25.0, 25.4, 28.0, 34.9, 40.2, 46.7, 85.4, 153.0, 174.6; (75 MHz, $CDCl_3$) (minor diastereoisomer) 17.6, 23.8, 23.9, 25.2, 27.8, 37.4, 45.5, 47.1, 86.5, 153.1, 174.0.

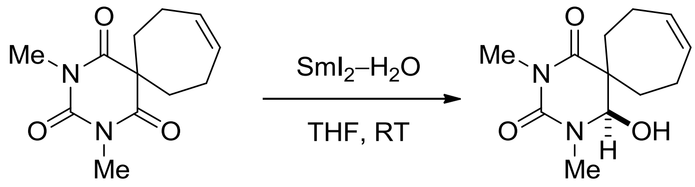

1k **2k**

5-Hydroxy-2,4-dimethyl-2,4-diazaspiro[5.6]dodec-9-ene-1,3-dione (2k). According to the general procedure, the reaction of **1k** (0.10 mmol), SmI_2 (0.30 mmol, 3 equiv, 5.5 mL, 0.055 M) and H_2O (1.8 mL, 1000 equiv) for 10 s afforded after purification by chromatography (1/1 EtOAc/hexanes) the title compound as a white solid (Mp 136-138 °C). Yield 55% (^1H NMR analysis vs. internal standard). Analytical sample was purified for characterization purposes. ^1H NMR (500 MHz, $CD_3C(O)CD_3$) δ 1.75 (br. dd, *J* = 9.0, 14.3 Hz, 1 H), 1.84 - 1.92 (m, 1 H), 1.92 - 1.98 (m, 1 H), 2.05 - 2.13 (m, 1 H), 2.18 - 2.29 (m, 3 H), 2.34 - 2.40 (m, 1 H), 3.05 (s, 3 H), 3.07 (s, 3 H), 4.88 (d, *J* = 5.4 Hz, 1 H), 5.58 (d, *J* = 5.4 Hz, 1 H), 5.59 - 5.63 (m, 1 H), 5.63 - 5.68 (m, 1 H); ^{13}C NMR (125 MHz, $CD_3C(O)CD_3$) δ 14.3, 14.6, 17.9, 19.3, 24.9, 24.9, 40.7, 84.7, 120.9, 122.1, 143.5, 165.1. IR (neat) 736, 763, 859, 928, 1030, 1062, 1109,

1227, 1295, 1376, 1422, 1484, 1658, 1709, 2849, 2927, 3016, 3401. HRMS calcd for $C_{12}H_{19}N_2O_3$ (M^+ + H) 239.1390, found 239.1397.

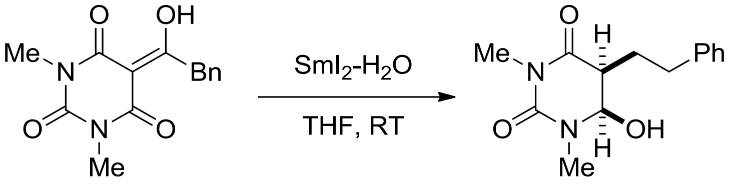

1l **2d**

(5*S*,6*R*)-6-Hydroxy-1,3-dimethyl-5-phenethyldihydropyrimidine-2,4(1*H*,3*H*)-dione (2d). According to the general procedure using preformed SmI_2–H_2O system, the reaction of **1l** (0.10 mmol), SmI_2 (0.80 mmol, 8 equiv, 8.0 mL, 0.10 M) and H_2O (1.8 mL, 1000 equiv) for 10 s afforded after purification by chromatography (1/1 EtOAc/hexanes) the title compound as a colorless oil. Yield 76%. Dr = 87:13 (crude), 88:12 (after purification). Rf (60% EtOAc/hexanes) = 0.46. Spectroscopic properties matched those described above for **2d**.

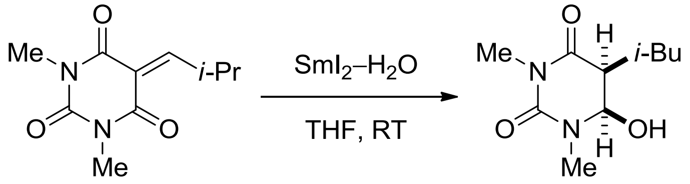

1m **2a**

(5*S*,6*R*)-6-Hydroxy-5-isobutyl-1,3-dimethyldihydropyrimidine-2,4(1*H*,3*H*)-dione (2a). According to the general procedure using preformed SmI_2–H_2O system, the reaction of **1m** (0.10 mmol), SmI_2 (0.60 mmol, 6 equiv, 6.0 mL, 0.10 M) and H_2O (1.8 mL, 1000 equiv) for 10 s afforded after purification by chromatography (1/1 EtOAc/hexanes) the title compound as a colorless oil. Yield 58%. Dr = 88:12 (crude), 88:12 (purified). Rf (50% EtOAc/hexanes) = 0.53. Spectroscopic properties matched those described above for **2a**.

Reductive Cyclization of Cyclic 1,3-Diimides using SmI_2–H_2O

General procedure for reductive cyclization of cyclic 1,3-diimides using SmI_2–H_2O. An oven-dried vial containing a stir bar was charged with a cyclic 1,3-diimide (1 equiv), placed under a positive pressure of argon, and subjected to three evacuation/backfilling cycles under high vacuum. THF (typically, 2.0 mL) and water (typically, 200 equiv) were added, followed by a rapid injection of SmI_2 (in THF, typically 6 equiv) with vigorous stirring. After the specified time (typically, 15 min), the reaction was quenched by bubbling air through the reaction mixture, diluted with CH_2Cl_2 (30 mL) and HCl (0.1 *N*, 20 mL), H_2O (20 mL) or $NaHCO_3$ (2% in H_2O, 20 mL). The aqueous layer was extracted with CH_2Cl_2 (3 x 20 mL), organic layers were combined, dried over Na_2SO_4, filtered and concentrated. The sample was analysed by ^{1}H NMR ($CDCl_3$, C_6D_6 or $CD_3C(O)CD_3$) and/or GC-MS (neat) to determine product distribution and diastereoselectivity from the crude reaction mixture. The crude product was purified by chromatography on silica gel, concentrated under reduced pressure and stored neat or as a solution in acetone. All compounds have been prepared as racemates.

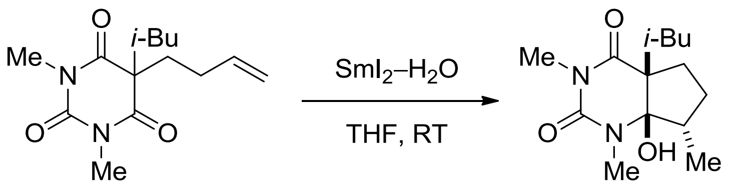

3a **4a**

(4a*S*,7*S*,7a*R*)-7a-Hydroxy-4a-isobutyl-1,3,7-trimethyltetrahydro-1*H*-cyclopenta[d] pyrimidine-2,4(3*H*,4a*H*)-dione (4a). According to the general procedure, the reaction of **3a** (0.10 mmol), SmI_2 (0.60 mmol, 6 equiv, 7.5 mL, 0.080 M) and H_2O (0.36 mL, 200 equiv) for 15 min afforded after purification by chromatography (1/1 EtOAc/hexanes) the title compound as a white solid. Mp = 112-114 °C. Yield 74%. Dr > 95:5 (crude), > 95:5 (purified). Stereochemistry of the major diastereoisomer was determined by X-ray analysis (re-crystallization from EtOAc). Rf (50% EtOAc/hexanes) = 0.41. ^{1}H NMR (400 MHz, $CDCl_3$) δ 0.56 (d, *J* = 7.6 Hz, 3 H), 0.69 (d, *J* = 6.8 Hz, 3 H), 0.77 (d, *J* = 6.4 Hz, 3 H), 1.07-1.16 (m, 1 H), 1.26 (dd, *J* = 8.4, 12.8 Hz, 1 H), 1.30-1.41 (m, 1 H), 1.44-1.53 (m, 2 H), 1.85-1.95 (m, 1 H), 2.09 (s, 1 H), 2.08-2.17 (m, 1 H), 2.58-2.66 (m, 1 H), 2.91 (s, 3 H), 3.02 (s, 3 H); ^{13}C NMR (100 MHz, $CDCl_3$) δ 17.4, 23.4, 24.7, 25.0, 28.4, 28.6, 29.4, 31.5, 42.8, 54.9, 95.3, 152.7, 172.8. IR (neat) 3418, 2957, 1704, 1644, 1455, 1414, 1385, 1326, 1122, 1086, 1044, 913, 754 cm^{-1}. HRMS calcd for $C_{14}H_{25}N_2O_3$ (M^+ + H) 269.1860, found 269.1864.

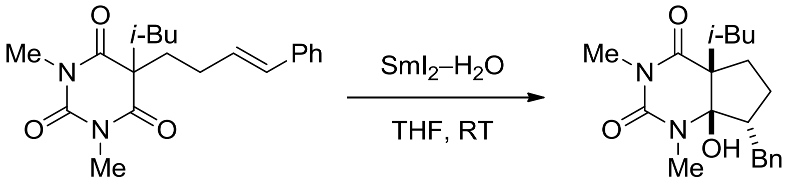

3b **4b**

(4a*S*,7*R*,7a*R*)-7-Benzyl-7a-hydroxy-4a-isobutyl-1,3-dimethyltetrahydro-1*H*-cyclopenta [d]pyrimidine-2,4(3*H*,4a*H*)-dione (4b). According to the general procedure, the reaction of **3b** (0.05 mmol), SmI_2 (0.30 mmol, 6 equiv, 3.75 mL, 0.080 M) and H_2O (2.16 mL, 2400 equiv) for 60 s afforded after purification by chromatography (1/1 EtOAc/hexanes) the title compound as a white solid. Mp = 131-132 °C. Yield 58%. Dr > 95:5 (crude), > 95:5 (purified). Stereochemistry of the major diastereoisomer was determined by analogy to **4a** and further confirmed by 2 D NMR experiments. Rf (50% EtOAc/hexanes) = 0.77. ^{1}H NMR (400 MHz, $CDCl_3$) δ 0.79 (d, *J* = 6.4 Hz, 3 H), 0.87 (d, *J* = 6.4 Hz, 3 H), 1.30-1.41 (m, 2 H), 1.42-1.51 (m, 1 H), 1.56-1.65 (m, 2 H), 1.71-1.80 (m, 1 H), 1.89 (t, *J* = 13.2 Hz, 1 H), 2.36-2.46 (m, 3 H), 2.70-2.78 (m, 1 H), 3.00 (s, 3 H), 3.17 (s, 3 H), 6.99 (d, *J* = 7.2 Hz, 2 H), 7.11 (t, *J* = 7.2 Hz, 1 H), 7.19 (t, *J* = 7.6 Hz, 2 H); ^{13}C NMR (100 MHz, $CDCl_3$) δ 23.4, 24.7, 25.1, 26.0, 28.6, 29.5, 31.3, 37.5, 42.8, 50.1, 55.3, 94.8, 126.4, 128.5, 128.8, 139.1, 152.6, 172.9. IR (neat) 3321, 2952, 2926, 2868, 1699, 1638, 1457, 1413, 1379, 1327, 1080, 1057, 1034, 756 cm^{-1}. HRMS calcd for $C_{20}H_{29}N_2O_3$ (M^+ + H) 345.2173, found 345.2183.

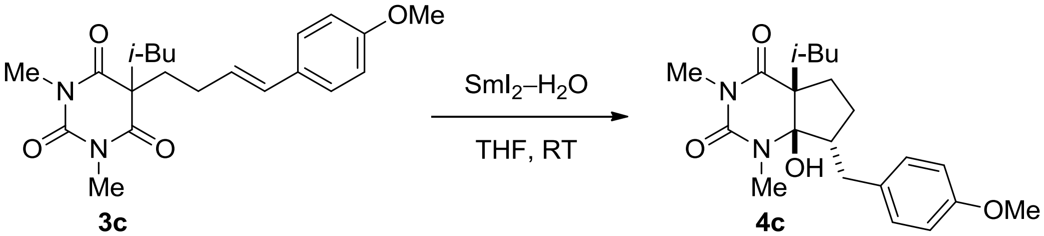

3c **4c**

(4a*S*,7*R*,7a*R*)-7a-Hydroxy-4a-isobutyl-7-(4-methoxybenzyl)-1,3-dimethyltetrahydro-1*H*-cyclopenta[d]pyrimidine-2,4(3*H*,4a*H*)-dione (4c). According to the general procedure, the reaction of **3c** (0.05 mmol), SmI_2 (0.30 mmol, 6 equiv, 3.75 mL, 0.080 M) and H_2O (2.16 mL, 2400 equiv) for 60 s afforded after purification by chromatography (1/1 EtOAc/hexanes) the title compound as a white solid. Mp = 133-134 °C. Yield 59%. Dr > 95:5 (crude), > 95:5 (purified). Stereochemistry of the major diastereoisomer was determined by analogy to **4a** and further confirmed by 2 D NMR experiments. Rf (50% EtOAc/hexanes) = 0.53. ^{1}H NMR (400 MHz, $CDCl_3$) δ 0.79 (d, *J* = 6.8 Hz, 3 H), 0.87 (d, *J* = 6.4 Hz, 3 H), 1.28-1.38 (m, 2 H), 1.41-1.48 (m, 1 H), 1.55-1.64 (m, 2 H), 1.69-1.88 (m, 2 H), 2.08 (s, 1 H), 2.32-2.41 (m, 2 H),

2.70-2.77 (m, 1 H), 3.00 (s, 3 H), 3.17 (s, 3 H), 3.71 (s, 3 H), 6.73 (d, J = 8.8 Hz, 2 H), 6.90 (d, J = 8.8 Hz, 2 H); ^{13}C NMR (100 MHz, $CDCl_3$) δ 23.4, 24.7, 25.1, 26.0, 28.6, 29.5, 31.2, 36.6, 42.8, 50.2, 55.2, 55.3, 94.8, 113.9, 129.7, 131.0, 152.5, 158.1, 172.8. IR (neat) 3439, 2953, 1704, 1651, 1511, 1456, 1418, 1364, 1329, 1246, 1177, 1034, 820, 754 cm^{-1}. HRMS calcd for $C_{21}H_{31}N_2O_4$ (M^+ + H) 375.2279, found 375.2267.

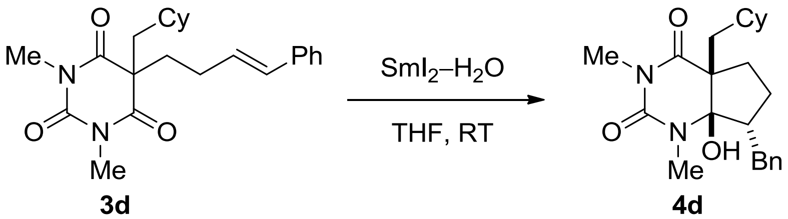

3d **4d**

(4a*S*,7*R*,7a*R*)-7-Benzyl-4a-(cyclohexylmethyl)-7a-hydroxy-1,3-dimethyltetrahydro-1*H*-cyclopenta[d]pyrimidine-2,4(3*H*,4a*H*)-dione (4c). According to the general procedure, the reaction of **3d** (0.05 mmol), SmI_2 (0.30 mmol, 6 equiv, 10.0 mL, 0.030 M) and H_2O (2.16 mL, 2400 equiv) for 5 min afforded after purification by chromatography (1/2 EtOAc/hexanes) the title compound as a white solid. Mp = 144-145 °C. Yield 64%. Dr > 95:5 (crude), > 95:5 (purified). Stereochemistry of the major diastereoisomer was determined by analogy to **4a** and further confirmed by 2 D NMR experiments. Rf (20% EtOAc/hexanes) = 0.31. ^{1}H NMR (500 MHz, $CDCl_3$) δ 0.75-0.85 (m, 2 H), 0.96-1.06 (m, 1 H), 1.10-1.19 (m, 3 H), 1.29-1.36 (m, 1 H), 1.38-1.44 (m, 2 H), 1.47-1.62 (m, 5 H), 1.70-1.79 (m, 2 H), 1.89 (t, J = 13.5 Hz, 1 H), 2.35 (s, 1 H), 2.38-2.45 (m, 2 H), 2.68-2.74 (m, 1 H), 2.99 (s, 3 H), 3.16 (s, 3 H), 6.99 (d, J = 7.0 Hz, 2 H), 7.11 (tt, J = 1.0, 7.0 Hz, 1 H), 7.19 (t, J = 8.0 Hz, 2 H); ^{13}C NMR (125 MHz, $CDCl_3$) δ 26.0, 26.1, 28.6, 29.5, 31.3, 34.1, 34.2, 35.0, 37.5, 41.5, 50.1, 55.0, 94.8, 126.3, 128.5, 128.8, 139.1, 152.6, 172.9. IR (neat) 3371, 2921, 2851, 1717, 1664, 1449, 1414, 1377, 1328, 1282, 1110, 1072, 1030, 912, 754, 731, 699 cm^{-1}. HRMS calcd for $C_{23}H_{33}N_2O_3$ (M^+ + H) 385.2486, found 385.2491.

(4a*S*,7*S*,7a*R*)-4a-(Cyclohexylmethyl)-7a-hydroxy-7-(*D*^{1}-(phenyl)methyl)-1,3-dimethyltetrahydro-1*H*-cyclopenta[d]pyrimidine-2,4(3*H*,4a*H*)-dione (4d-*D*^{1}). According to the general procedure, the reaction of **3d** (0.05 mmol), SmI_2 (0.15 mmol, 3 equiv, 1.7 mL, 0.087 M) and D_2O (0.90 mL, 1000 equiv) for 5 min at rt afforded after purification by chromatography (40/60 EtOAc/hexanes) the title compound with >98% *D*^{1} incorporation. Yield 54%. Dr > 95:5 (crude), > 95:5 (purified). Dr at benzylic position 1:1 (crude), 1:1 (purified). ^{1}H NMR (500 MHz, $CD_3C(O)CD_3$) δ (mixture of *D*^{1} diastereoisomers) 0.66 (qd, J

= 3.0, 11.0 Hz, 2 H), 0.84-1.13 (m, 5 H), 1.26-1.32 (m, 1 H), 1.34-1.47 (m, 6 H), 1.50-1.56 (m, 1 H), 1.58-1.63 (m, 1 H), 1.71 (d, J = 12.0 Hz, 0.5 H), 2.30-2.38 (m, 1.5 H), 2.44-2.50 (m, 1 H), 2.82 (s, 3 H) and 2.83 (s, 3 H), 2.93 (s, 3 H), 4.88 (d, J = 1.0 Hz, 1 H), 6.91 (d, J = 8.0, 2 H), 6.96 (tt, J = 1.5, 7.5 Hz, 1 H), 7.05 (t, J = 8.0 Hz, 2 H); ^{13}C NMR (125 MHz, $CD_3C(O)CD_3$) δ (mixture of D^1 diastereoisomers) 26.6 and 26.7, 26.9, 27.0 and 27.1, 28.5, 29.5, 31.9 and 31.9, 34.9, 35.0, 35.7, 38.3 (t, J^1 = 19.0 Hz) and 38.2 (t, J^1 = 19.0 Hz), 42.3, 51.4 (d, J^2 = 4.5 Hz) and 51.5 (d, J^2 = 4.5 Hz), 56.2, 94.8 and 94.9, 126.9, 129.3, 129.7, 140.7, 152.9, 173.9. Kinetic isotope effect was determined by reacting **3d** (0.032 mmol), SmI_2 (0.19 mmol, 6 equiv, 2.2 mL, 0.085 M) and D_2O/H_2O (1:1, 1.40 mL, 2400 equiv) for 60 s at rt, followed by standard work-up and purification by chromatography (40/60 EtOAc/hexanes) to give the title compound with 48.5% D^1 incorporation as determined by ^{1}H NMR (500 MHz) and GC-MS (k_H/k_D = 1.05±0.1).

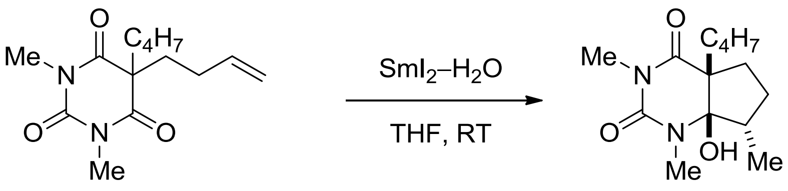

3e **4e**

(4a*R*,7*S*,7a*R*)-4a-(But-3-en-1-yl)-7a-hydroxy-1,3,7-trimethyltetrahydro-1*H*-cyclopenta[*d*]pyrimidine-2,4(3*H*,4a*H*)-dione (4e). According to the general procedure, the reaction of **3e** (0.10 mmol), SmI_2 (0.60 mmol, 6 equiv, 7.1 mL, 0.085 M) and H_2O (0.36 mL, 200 equiv) for 15 min afforded after purification by chromatography (1/1 EtOAc/hexanes) the title compound as a colorless oil. Yield 55%. Dr > 95:5 (crude), > 95:5 (purified). Stereochemistry of the major diastereoisomer determined by 2 D NMR experiments and confirmed by an X-ray analysis of a derivative. ^{1}H NMR (500 MHz, $CD_3C(O)CD_3$) δ 0.70 (d, J = 7.3 Hz, 3 H), 1.16 (dtd, J = 4.7, 8.5, 13.0 Hz, 1 H), 1.64 (ddd, J = 8.5, 9.8, 12.9 Hz, 1 H), 1.70 - 1.83 (m, 2 H), 1.84 - 1.94 (m, 1 H), 1.94 - 2.08 (m, 2 H), 2.38 - 2.46 (m, 1 H), 2.57 (ddd, J = 4.1, 8.5, 12.9 Hz, 1 H), 3.02 (s, 3 H), 3.08 (s, 3 H), 4.88 (ddt, J = 1.5, 2.2, 10.4 Hz, 1 H), 4.96 (dq, J = 1.5, 17.1 Hz, 1 H), 5.12 (s, 1 H), 5.75 (ddt, J = 6.5, 10.4, 17.1 Hz, 1 H); ^{13}C NMR (125 MHz, $CD_3C(O)CD_3$) δ 17.9, 28.3, 29.3, 29.3, 29.9, 32.0, 34.4, 45.3, 56.1, 94.3, 114.9, 139.3, 152.9, 173.7. IR (neat) 648, 755, 913, 1047, 1089, 1121, 1332, 1385, 1411, 1456, 1649, 1702, 2874, 2930, 2960, 3401. HRMS calcd for $C_{14}H_{21}N_2O_2$ ($M^+ - OH$) 249.1598, found 249.1601.

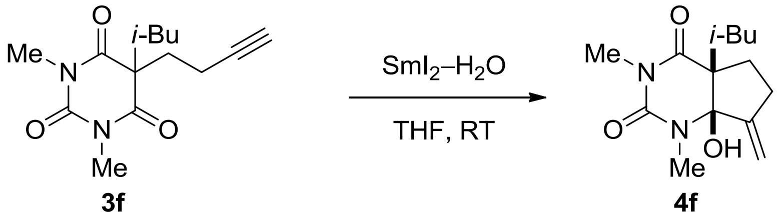

(4a*S*,7a*R*)-7a-Hydroxy-4a-isobutyl-1,3-dimethyl-7-methylenetetrahydro-1*H*-cyclopenta[*d*]pyrimidine-2,4(3*H*,4a*H*)-dione (4f). According to the general procedure, the reaction of **3f** (0.10 mmol), SmI_2 (0.60 mmol, 6 equiv, 7.1 mL, 0.085 M) and H_2O (0.36 mL, 200 equiv) for 15 min afforded after purification by chromatography (1/1 EtOAc/hexanes) the title compound as a colorless oil. Yield 63%. Dr > 95:5 (crude), > 95:5 (purified). Stereochemistry of the major diastereoisomer determined by 2 D NMR experiments and confirmed by an X-ray analysis of a derivative. ^{1}H NMR (400 MHz, $CD_3C(O)CD_3$) δ 0.86 (d, *J* = 6.6 Hz, 3 H), 0.90 (d, *J* = 6.6 Hz, 3 H), 1.53 (dd, *J* = 7.6, 14.3 Hz, 1H), 1.62 (dd, *J* = 4.5, 14.3 Hz, 1 H), 1.66 - 1.75 (m, 1 H), 1.78 - 1.88 (m, 1 H), 2.19 - 2.31 (m, 1 H), 2.36 - 2.49 (m, 2 H), 3.04 (s, 3 H), 3.12 (s, 3 H), 4.99 (t, *J* = 2.3 Hz, 1 H), 5.15 (t, *J* = 2.3 Hz, 1 H), 5.56 (s, 1 H); ^{13}C NMR (100 MHz, $CD_3C(O)CD_3$) δ 24.5, 25.2, 25.7, 26.5, 28.4, 29.7, 30.9, 41.1, 56.5, 91.6, 109.6, 151.9, 152.8, 173.4. IR (neat) 759, 909, 1070, 1110, 1325, 1386, 1416, 1460, 1650, 1706, 2868, 2955, 3376. HRMS calcd for $C_{14}H_{21}N_2O_2$ (M^+–OH) 249.1598, found 249.1605.

(4a*S*,7a*S*)-7a-hydroxy-4a-isobutyl-1,3-dimethyl-7-(D^1-methylene)tetrahydro-1*H*-cyclopenta[*d*]pyrimidine-2,4(3*H*,4a*H*)-dione (4f-D^1). According to the general procedure, reaction of **3f** (0.10 mmol), SmI_2 (0.60 mmol, 6 equiv, 7.1 mL, 0.085 M) and D_2O (0.36 mL, 200 equiv) for 15 min afforded after purification by chromatography (1/1 EtOAc/hexanes) the title compound with >98% deuterium incorporation as a colorless oil. Yield 67%. Dr (at the hemiaminal carbon) > 95:5 (crude), > 95:5 (purified). Dr (at the vinylic position) = 66:34. (major diastereoisomer) ^{1}H NMR (400 MHz, $CD_3C(O)CD_3$) δ (major diastereoisomer) 0.86 (d, *J* = 6.6 Hz, 0 H), 0.89 (d, *J* = 6.6 Hz, 3 H), 1.53 (dd, *J* = 7.6, 14.4 Hz, 1 H), 1.62 (dd, *J* = 4.3, 14.4 Hz, 1 H), 1.66 - 1.74 (m, 1 H), 1.78 - 1.87 (m, 1 H), 2.19 - 2.29 (m, 1 H), 2.36 - 2.49 (m, 2 H), 3.04 (s, 3 H), 3.11 (s, 3 H), 5.13 (t, *J* = 2.5 Hz, 1 H); (minor, diagnostic peaks only) 4.97 (t, *J* = 2.1 Hz, 1 H); ^{13}C NMR (100 MHz, $CD_3C(O)CD_3$) δ (major diastereoisomer) 24.5, 25.2, 25.7, 28.4, 29.7, 30.8, 41.0, 56.4, 91.6, 109.1, 152.9, 154.3, 173.4; IR (neat) 757, 810, 847, 902, 974, 1025, 1067, 1109, 1205, 1324, 1386, 1414, 1454, 1647, 1705, 2865, 2955, 3363; HRMS calcd for $C_{14}H_{20}DN_2O_2$ (M^+ – OH) 250.1660, found 250.1665. Kinetic isotope

effect was determined by reacting **3f** (0.10 mmol), SmI_2 (0.60 mmol, 6 equiv, 7.1 mL, 0.085 M) and D_2O/H_2O (1:1, 0.36 mL, 200 equiv) for 15 min at rt, followed by standard work-up to give the title compound with 36.4% D^1 incorporation as determined by ^{1}H NMR (500 MHz) analysis (k_H/k_D = 1.75±0.1).

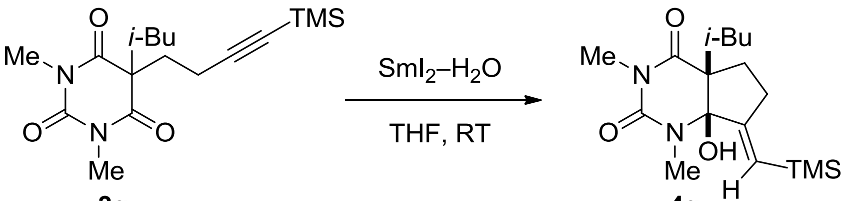

3g **4g**

(4a*S*,7a*S*,*E*)-7a-Hydroxy-4a-isobutyl-1,3-dimethyl-7-((trimethylsilyl)methylene) tetrahydro-1*H*-cyclopenta[*d*]pyrimidine-2,4(3*H*,4a*H*)-dione (4g). According to the general procedure, the reaction of **3e** (0.10 mmol), SmI_2 (0.60 mmol, 6 equiv, 7.1 mL, 0.085 M) and H_2O (0.36 mL, 200 equiv) for 15 min afforded after purification by chromatography (1/1 EtOAc/hexanes) the title compound as a colorless solid (mp 156-158 °C). Yield 66%. Dr > 95:5 (crude), > 95:5 (purified). Stereochemistry of the major diastereoisomer determined by 2 D NMR experiments and confirmed by an X-ray analysis. ^{1}H NMR (400 MHz, $CD_3C(O)CD_3$) δ 0.09 (s, 9 H), 0.88 (d, *J* = 6.6 Hz, 3 H), 0.90 (d, *J* = 6.6 Hz, 3 H), 1.47 (dd, *J* = 7.3, 14.6 Hz, 1 H), 1.60 (dd, *J* = 4.3, 14.6 Hz, 1 H), 1.70 - 1.80 (m, 1 H), 1.85 - 1.92 (m, 1 H), 2.26 - 2.38 (m, 2 H), 2.44 - 2.53 (m, 1 H), 3.03 (s, 3 H), 3.07 (s, 3 H), 5.51 (s, 1 H), 5.72 (t, *J* = 2.3 Hz, 1 H); ^{13}C NMR (100 MHz, $CD_3C(O)CD_3$) δ -0.6, 24.7, 25.3, 25.7, 26.8, 28.3, 29.8, 30.8, 40.8, 55.6, 92.7, 123.0, 153.0, 159.3, 173.6. IR (neat) 760, 846, 1024, 1063, 1103, 1249, 1324, 1385, 1414, 1462, 1652, 1707, 2955, 3390. HRMS calcd for $C_{17}H_{31}N_2O_3Si$ (M^+ + H) 339.2098, found 339.2113.

(4a*S*,7a*S*,*E*)-7a-Hydroxy-4a-isobutyl-1,3-dimethyl-7-(D^1-(trimethylsilyl)methylene) tetrahydro-1*H*-cyclopenta[*d*]pyrimidine-2,4(3*H*,4a*H*)-dione (4g-D^1). According to the general procedure, the reaction of **3e** (0.10 mmol), SmI_2 (0.60 mmol, 6 equiv, 5.5 mL, 0.110 M) and D_2O (0.36 mL, 200 equiv) for 15 min afforded after purification by chromatography (1/1 EtOAc/hexanes) the title compound as a colorless solid (Mp 156-158 °C) with >98% deuterium incorporation. Yield 78%. dr > 95:5 (crude), > 95:5 (purified). Stereochemistry of the major diastereoisomer determined by 2 D NMR experiments and confirmed by an X-ray analysis of a derivative. ^{1}H NMR (400 MHz, $CD_3C(O)CD_3$) δ 0.09 (s, 9 H), 0.87 (d, *J* = 6.7 Hz, 3 H), 0.90 (d, *J* = 6.7 Hz, 3 H), 1.47 (dd, *J* = 7.3, 14.4 Hz, 1 H), 1.60 (dd, *J* = 4.3, 14.4

Hz, 1 H), 1.70 - 1.81 (m, 1 H), 1.85 - 1.92 (m, 1 H), 2.26 - 2.38 (m, 2 H), 2.43 - 2.54 (m, 1 H), 3.03 (s, 3 H), 3.07 (s, 3 H), 5.52 (s, 1 H); ^{13}C NMR (100 MHz, $CD_3C(O)CD_3$) δ −0.6, 24.7, 25.3, 25.7, 26.7, 28.3, 29.8, 30.8, 40.8, 55.6, 92.6, 122.6 (d, J = 20.3 Hz), 153.0, 159.1, 173.6; IR(neat) 649, 690, 759, 837, 999, 1054, 1111, 1207, 1248, 1322, 1386, 1413, 1457, 1648, 1705, 2867, 2954, 3364; HRMS calcd for $C_{17}H_{30}DN_2O_3Si$ (M^+ + H) 340.2161, found 340.2165. Kinetic isotope effect was determined by reacting **3e** (0.10 mmol), SmI_2 (0.60 mmol, 6 equiv, 7.1 mL, 0.085 M) and D_2O/H_2O (1:1, 0.36 mL, 200 equiv) for 15 min at rt, followed by standard work-up to give the title compound with 39.4% D^1 incorporation as determined by ^{1}H NMR (500 MHz) analysis (k_H/k_D = 1.54±0.1).

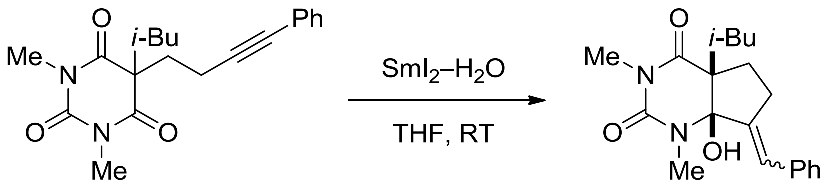

3h **4h**

(4a*S*,7a*R*,*E*)-7-Benzylidene-7a-hydroxy-4a-isobutyl-1,3-dimethyltetrahydro-1*H*-cyclopenta[*d*]pyrimidine-2,4(3*H*,4a*H*)-dione (4h). According to the general procedure, the reaction of **3h** (0.10 mmol), SmI_2 (0.60 mmol, 6 equiv, 5.5 mL, 0.110 M) and H_2O (0.36 mL, 200 equiv) for 15 min afforded after purification by chromatography (1/1 EtOAc/hexanes) the title compound as a colorless oil. Yield 90%. E:Z = 37:63. Stereochemistry of the major diastereoisomer was determined by 2 D NMR experiments. ^{1}H NMR (500 MHz, $CD_3C(O)CD_3$) δ (major diastereoisomer) 0.87 (d, J = 6.6 Hz, 3 H), 0.90 (d, J = 6.6 Hz, 3 H), 1.51 - 1.67 (m, 2 H), 1.69 - 1.76 (m, 1 H), 1.91 - 1.99 (m, 1 H), 2.50 - 2.56 (m, 2 H), 2.69 - 2.76 (m, 1 H), 3.04 (s, 3 H), 3.23 (s, 3 H), 5.72 (s, 1 H), 6.56 (t, J = 2.4 Hz, 1 H), 7.24 - 7.31 (1 H, m, 1 H), 7.34 - 7.39 (m, 4 H); (minor, diagnostic peaks only) 0.80 (d, J = 6.6 Hz, 3 H), 0.89 (d, J = 6.6 Hz, 3 H), 1.47 (dd, J = 4.7, 14.2 Hz, 1 H), 1.80 (ddd, J = 7.9, 10.4, 12.0 Hz, 1 H), 2.29 (s, 3 H), 2.39 (dddd, J = 1.9, 7.9, 9.8, 17.1 Hz, 1 H), 2.60 (ddt, J = 2.5, 10.4, 17.1 Hz, 1 H), 3.07 (s, 3 H), 5.65 (s, 1 H), 6.53 (br. s, 1 H), 7.04 - 7.08 (m, 2 H), 7.18 - 7.23 (m, 1 H); ^{13}C NMR (125 MHz, $CD_3C(O)CD_3$) δ (major diastereoisomer) 24.6, 25.2, 25.7, 26.3, 28.4, 30.0, 31.5, 40.8, 55.9, 93.1, 124.6, 128.2, 129.3, 129.9, 137.3, 144.1, 152.9, 173.3; (minor, diagnostic peaks only) 24.4, 25.0, 25.6, 28.4, 28.8, 29.5, 29.5, 40.3, 58.2, 91.1, 127.1, 127.5, 128.9, 129.1, 129.3, 138.2, 144.0, 152.0, 173.2. IR (neat) 699, 756, 1029, 1069, 1104, 1234, 1383, 1454, 1653, 2869, 2955, 3360. HRMS calcd for $C_{20}H_{27}N_2O_3$ (M^+ + H) 343.2016, found 343.2014.

(4a*S*,7a*R*,*E*)-7a-Hydroxy-4a-isobutyl-1,3-dimethyl-7-(D^1-(phenyl)methylene)tetrahydro-1*H*-cyclopenta[*d*]pyrimidine-2,4(3*H*,4a*H*)-dione (4h-D^1). According to the general procedure, the reaction of **3h** (0.06 mmol), SmI_2 (0.36 mmol, 6 equiv, 3.3 mL, 0.110 M) and D_2O (0.05 mL, 50 equiv) for 15 min afforded after purification by chromatography (1/1 EtOAc/hexanes) the title compound as a colorless oil with >98% deuterium incorporation. Yield 65%. *E*:*Z* = 1.5:1. Stereochemistry of the major diastereoisomer determined by 2 D NMR experiments. ^{1}H NMR (400 MHz, $CD_3C(O)CD_3$) δ (major diastereoisomer) 0.87 (d, *J* = 6.6 Hz, 3 H), 0.89 (d, *J* = 6.6 Hz, 3 H), 1.56 (dd, *J* = 7.3, 14.3 Hz, 1 H), 1.65 (dd, *J* = 4.5, 14.3 Hz, 1 H), 1.68 - 1.77 (m, 1 H), 1.92 - 2.00 (m, 1 H), 2.47 - 2.58 (m, 2 H), 2.65 - 2.79 (m, 1 H), 3.04 (s, 3 H), 3.23 (s, 3 H), 5.72 (s, 1 H), 7.14 - 7.31 (m, 1 H), 7.32 - 7.41 (m, 4 H); (minor, diagnostic peaks only) 0.80 (d, *J* = 6.6 Hz, 3 H), 0.90 (d, *J* = 6.6 Hz, 3 H), 1.44 - 1.52 (m, 1 H), 1.62 - 1.68 (m, 1 H), 1.80 (dt, *J* = 2.2, 10.0 Hz, 1 H), 2.30 (s, 3 H), 2.33 - 2.44 (m, 1 H), 2.59 - 2.65 (m, 1 H), 3.07 (s, 3 H), 5.65 (s, 1 H), 7.04 - 7.10 (m, 2 H); ^{13}C NMR (100 MHz, $CD_3C(O)CD_3$) δ (major diastereoisomer) 24.6, 25.2, 25.7, 26.3, 28.3, 29.9, 31.6, 40.8, 55.9, 93.1, 124.3, 128.2, 129.3, 129.9, 137.3, 144.1, 152.9, 173.3; (minor, diagnostic peaks only) 24.4, 25.0, 25.6, 28.3, 28.7, 29.3, 29.5, 40.3, 58.2, 93.0, 124.0, 127.5, 128.9, 129.1, 137.5, 143.9, 152.0, 173.2. IR (neat) 698, 759, 1000, 1029, 1066, 1110, 1323, 1380, 1414, 1450, 1650, 1704, 2870, 2954, 3363. HRMS calcd for $C_{20}H_{24}DN_2O_2$ (M^+–OH) 326.1973, found 326.1979.

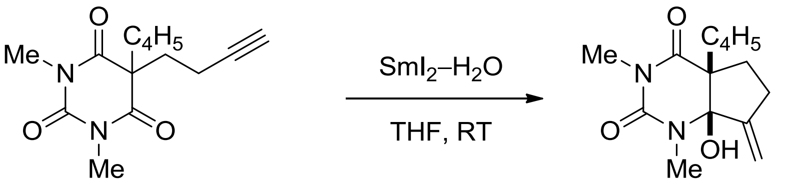

(4a*R*,7a*R*)-4a-(But-3-yn-1-yl)-7a-hydroxy-1,3-dimethyl-7-methylenetetrahydro-1*H*-cyclopenta[*d*]pyrimidine-2,4(3*H*,4a*H*)-dione (4i). According to the general procedure, the reaction of **3i** (0.10 mmol), SmI_2 (0.60 mmol, 6 equiv, 7.1 mL, 0.085 M) and H_2O (0.36 mL, 200 equiv) for 15 min afforded after purification by chromatography (1/1 EtOAc/hexanes) the title compound as a colorless oil. Yield 82%. Dr > 95:5 (crude), > 95:5 (purified). ^{1}H NMR (500 MHz, $CD_3C(O)CD_3$) δ 1.85 - 1.99 (m, 3 H), 2.06 - 2.12 (m, 1 H), 2.25 - 2.32 (m, 1 H), 2.33 (t, *J* = 2.5 Hz, 1 H), 2.34 - 2.43 (m, 1 H), 2.45 - 2.52 (m, 1 H), 2.51 - 2.59 (m, 1 H), 2.99 (s, 3 H), 3.07 (s, 3 H), 5.20 (t, *J* = 2.4 Hz, 1 H), 5.37 (t, *J* = 2.4 Hz, 1 H), 5.62 (s, 1

H);^{13}C NMR (125 MHz, $CD_3C(O)CD_3$) δ 15.2, 26.2, 28.2, 29.6, 31.3, 32.6, 54.4, 69.8, 85.2, 92.4, 111.8, 150.6, 152.9, 173.0. IR (neat) 759, 913, 1054, 1136, 1220, 1334, 1385, 1415, 1459, 1649, 1704, 2855, 2925, 3298. HRMS calcd for $C_{14}H_{19}N_2O_3$ (M^+ + H) 263.1390, found 263.1386.

Transformations of Cyclic alpa-Hydroxy Carboxamides

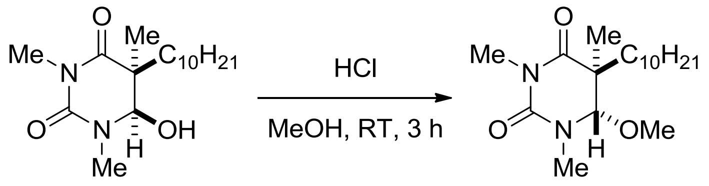

(5*R*,6*R*)-5-Decyl-6-methoxy-1,3,5-trimethyldihydropyrimidine-2,4(1*H*,3*H*)-dione (5a). A 10 mL vial was charged with **2i** (0.032 mmol, 1.0 equiv), MeOH (1 mL) and HCl (0.2 mL, 2.0 *N* in Et_2O), and stirred at room temperature for 3 h. Removal of solvent under reduced pressure afforded the title compound in 99% yield (^{1}H NMR analysis). Dr = 71:39. Note that the title compound is unstable and should be used immediately after preparation. ^{1}H NMR (400 MHz, $CD_3C(O)CD_3$) δ 0.82 (t, J = 6.6 Hz, 3 H), 1.18 (s, 3 H), 1.19 - 1.45 (m, 16 H), 1.45 - 1.55 (m, 1 H), 1.60 - 1.76 (m, 1 H), 3.00 (s, 3 H), 3.00 (s, 3 H), 3.13 - 3.17 (m, 3 H), 4.59 (br. s, 1 H); ^{13}C NMR (100 MHz, $CD_3C(O)CD_3$) δ ppm 14.3, 17.7, 23.3, 24.6, 27.6, 29.9, 30.0, 30.2, 30.6, 31.1, 32.5, 33.3, 34.7, 37.4, 48.0, 86.1, 153.6, 174.3. IR (neat) 722, 764, 930, 1040, 1070, 1184, 1293, 1380, 1421, 1467, 1487, 1657, 1713, 2854, 2924. HRMS calcd for $C_{17}H_{31}N_2O_2$ ($M^+ - OCH_3$) 295.2380, found 295.2392.

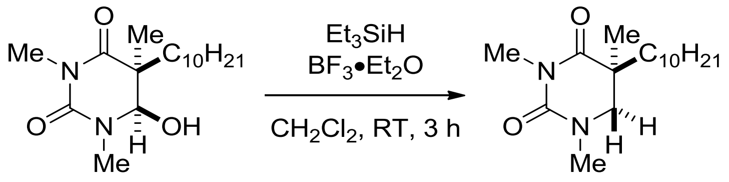

5-Decyl-1,3,5-trimethyldihydropyrimidine-2,4(1*H*,3*H*)-dione (5b). To a 10 mL vial charged with **2i** (0.032 mmol, 1.0 equiv) and CH_2Cl_2 (1 mL), triethylsilane (0.05 mL, 0.32 mmol, 10 equiv) was added at -78 °C followed by BF_3•OEt_2 (0.02 mL, 0.16 mmol, 5 equiv). After stirring for 5 min at -78 °C, the reaction mixture was warmed up to room temperature over 3 h, quenched with NH_4Cl (aq, sat., 1 mL) and extracted with CH_2Cl_2 (3 × 2 mL). Purification by chromatography using EtOAc/hexanes (30%) afforded the title compound as a colorless oil. Yield 96%. ^{1}H NMR (500 MHz, $CD_3C(O)CD_3$) δ 0.87 (t, J = 6.9 Hz, 3 H), 1.14 (s, 3 H), 1.22 - 1.34 (m, 16 H), 1.49 - 1.62 (m, 2 H), 3.00 (s, 3 H), 3.03 (s, 3 H), 3.21 (d, J = 12.5 Hz, 1 H), 3.25 (d, J = 12.5 Hz, 1 H); ^{13}C NMR (125 MHz, $CD_3C(O)CD_3$) δ 14.4, 20.9, 23.4, 24.5, 28.0, 30.0, 30.1, 30.2, 30.3, 30.9, 32.7, 35.9, 36.8, 42.1, 54.0, 154.3, 175.3. IR (neat) 757, 1062, 1184, 1285, 1377, 1416, 1447, 1491, 1673, 1712, 2854, 2924. HRMS calcd for $C_{17}H_{33}N_2O_2$ ($M^+ + H$) 297.2537, found 297.2536.

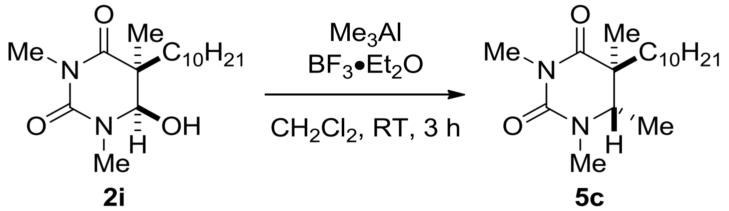

(5*R*,6*R*)-5-Decyl-1,3,5,6-tetramethyldihydropyrimidine-2,4(1*H*,3*H*)-dione (5c). To a 10 mL vial charged with **2i** (0.032 mmol, 1.0 equiv) and CH_2Cl_2 (1 mL), trimethylaluminium (2.0 *M* in hexane, 0.32 mmol, 10 equiv), followed by BF_3•OEt_2 (0.02 mL, 0.16 mmol, 5 equiv) were added, the reaction mixture stirred at room temperature for 3 h, quenched with NH_4Cl (aq, sat., 1 mL) and extracted with CH_2Cl_2 (3 × 2 mL). Purification by chromatography using EtOAc/hexanes (30%) afforded the title compound as a colorless oil. Yield 78%. Dr = 91:9. ^1H NMR (400 MHz, $CD_3C(O)CD_3$) δ (major diastereoisomer) 0.87 (t, *J* = 6.8 Hz, 3 H), 1.07 (d, *J* = 6.6 Hz, 3 H), 1.10 (s, 3 H), 1.11 - 1.17 (m, 1 H), 1.21 - 1.36 (m, 14 H), 1.36 - 1.42 (m, 1 H), 1.46 (ddd, *J* = 4.4, 12.2, 13.4 Hz, 1 H), 1.61 (ddd, *J* = 4.4, 12.2, 13.4 Hz, 1 H), 3.00 (s, 3 H), 3.04 (s, 3 H), 3.27 (q, *J* = 6.6 Hz, 1 H); (minor, diagnostic peaks only) 0.88 (t, *J* = 6.8 Hz, 3 H), 1.06 (d, *J* = 6.6 Hz, 3 H), 1.19 (s, 3 H), 1.42 - 1.51 (m, 1 H), 1.81 (dd, *J* = 10.1, 13.1 Hz, 1 H), 3.01 (s, 3 H), 3.04 (s, 3 H), 3.26 (q, *J* = 6.6 Hz, 1 H); ^{13}C NMR (100 MHz, $CD_3C(O)CD_3$) δ (major diastereoisomer) 14.2, 14.4, 18.3, 23.4, 24.6, 27.8, 30.1, 30.2, 30.4, 30.4, 30.7, 32.7, 34.6, 37.7, 46.4, 59.0, 153.1, 174.7; (minor, diagnostic peaks only) 13.8, 21.7, 23.1, 31.1, 33.7, 58.8, 153.4, 174.9. IR (neat) 722, 758, 1055, 1109, 1185, 1285, 1415, 1459, 1670, 1710, 2855, 2924. HRMS calcd for $C_{18}H_{34}N_2O_2Na$ (M^+ + Na) 333.2512, found 333.2504.

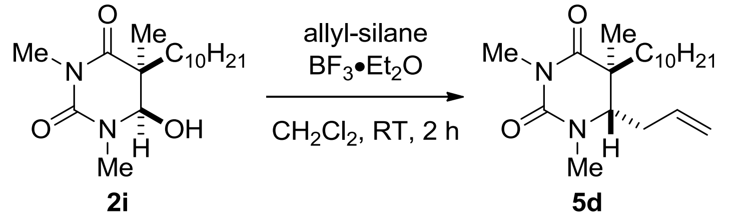

(5*R*,6*R*)-6-Allyl-5-decyl-1,3,5-trimethyldihydropyrimidine-2,4(1*H*,3*H*)-dione (5d). To a solution of **2i** (0.032 mmol) and allyltrimethylsilane (10 equiv) in CH_2Cl_2 (1.0 mL), BF_3•Et_2O (3 equiv) was added dropwise at rt and the reaction was stirred at rt for 2 h. The reaction was diluted with CH_2Cl_2 (20 mL) and HCl (0.1 *N*, 20 mL), extracted with CH_2Cl_2 (2 x 20 mL), dried and concentrated. Purification by chromatography (1/1 EtOAc/hexanes) afforded the title compound as a colorless oil. Yield 86%. Rf (50% EtOAc/hexanes) = 0.76. Dr > 95:5 (crude), > 95:5 (purified). Stereochemistry of the major diastereoisomer was

determined by 2 D NMR experiments. ^{1}H NMR (500 MHz, $CDCl_3$) δ 0.81 (t, *J* = 7.0 Hz, 3 H), 0.99-1.08 (m, 1 H), 1.13 (s, 3 H), 1.14-1.26 (m, 15 H), 1.33 (td, *J* = 4.0, 12.0 Hz, 1 H), 1.53 (td, *J* = 4.5, 13.0 Hz, 1 H), 2.11-2.17 (m, 1 H), 2.30-2.36 (m, 1 H), 3.00 (s, 3 H), 3.02 (dd, *J* = 4.5, 7.5 Hz, 1 H), 3.05 (s, 3 H), 4.98-5.03 (m, 2 H), 5.55-5.65 (m, 1 H); ^{13}C NMR (125 MHz, $CDCl_3$) δ 14.1, 18.1, 22.7, 23.8, 27.7, 29.3, 29.4, 29.5, 29.5, 29.9, 31.9, 34.3, 36.7, 37.7, 44.9, 63.1, 119.1, 132.6, 153.1, 174.5. IR (neat) 2924, 2854, 1669, 1467, 1417, 1381, 1366, 1283, 1217, 1083, 917, 757 cm^{-1}. HRMS calcd for $C_{20}H_{37}N_2O_2$ (M^+ + H) 337.2850, found 337.2852.

Reactivity of α-Hydroxy Carboxamides – Additional Example

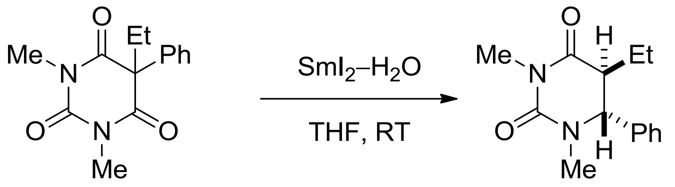

1-SI **2-SI**

5-Ethyl-1,3-dimethyl-6-phenyldihydropyrimidine-2,4(1*H*,3*H*)-dione (2-SI). According to the general procedure **1-SI** (0.10 mmol) was reacted with SmI_2 (0.80 mmol, 8 equiv, 10.0 mL, 0.080 M) and H_2O (1.8 mL, 1000 equiv) for an extended period of 30 min at rt, followed by work-up with CH_2Cl_2/HCl (1.0 *N*) as described above to give after purification by chromatography (1/1 EtOAc/hexanes) the title compound as a colorless oil. Yield 84%. Dr = 72:28 (crude), 71:29 (purified). Rf (50% EtOAc/hexanes) = 0.44. ^{1}H NMR (500 MHz, $CDCl_3$) δ (major diastereoisomer) 0.85 (t, *J* = 8.0 Hz, 3 H), 1.82-1.91 (m, 1 H), 2.11-2.20 (m, 1 H), 2.72 (s, 3 H), 2.85 (s, 3 H), 2.99-3.03 (m, 1 H), 3.92 (d, *J* = 3.5 Hz, 1 H), 7.05 (dd, *J* = 1.5, 7.0 Hz, 2 H), 7.13-7.24 (m, 3 H); (minor) 0.82 (t, *J* = 7.0 Hz, 3 H), 1.82-1.91 (m, 2 H), 2.68 (s, 3 H), 2.96 (s, 3 H), 3.05-3.09 (m, 1 H), 3.93 (d, *J* = 3.0 Hz, 1 H), 7.10 (dd, *J* = 1.0, 7.0 Hz, 2 H), 7.13-7.23 (m, 3 H); ^{13}C NMR (125 MHz, $CDCl_3$) δ (major diastereoisomer) 12.5, 22.6, 24.5, 28.3, 47.4, 66.0, 127.5, 128.2, 128.5, 137.9, 156.6, 172.0; (minor) 12.5, 24.6, 25.0, 30.6, 49.5, 66.5, 127.5, 128.4, 128.6, 137.6, 157.2, 172.2. IR (neat) 2965, 2876, 1767, 1705, 1452, 1421, 1396, 1273, 1216, 1023, 757, 703 cm^{-1}. HRMS calcd for $C_{14}H_{18}N_2O_2Na$ (M^+ + Na) 269.1260, found 269.1255. *Full details of this rearrangement will be reported separately.*

Mechanistic Studies

A) Effect of Additives and Optimization Studies

Table SI-1. *Effect of Additives on the Reduction of Cyclic 1,3-Diimides using SmI_2.*[a]

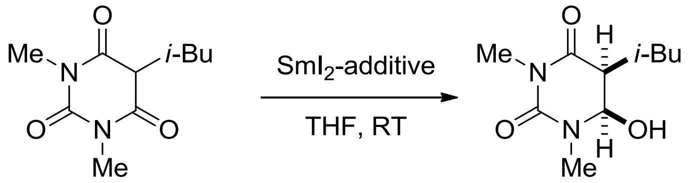

entry	SmI_2 (equiv)	additive	additive (equiv)	time[b]	conv.[c,d] (%)	yield[c] (%)	dr[c]
1	6	-	-	24 h	<5 (83 SM)	-	-
2	6	MeOH	4/1 v/v	2 h	<5 (81 SM)	-	-
3	4	*t*-BuOH	24	2 h	<5 (85 SM)	-	-
4	4	H_2O	1000	10 s	93 (7 SM)	84	86:14
5[e]	4	H_2O	1000	1 h	<5 (95 SM)	-	-
6	4	HMPA	24	2 h	>95 (<5 SM)	-	-
7	4	LiCl	48	2 h	27 (73 SM)	8.6	81:19
8	4	$HO(CH_2)_2OH$	24	2 h	>95 (<5 SM)	11	81:19
9	2	Et_3N-MeOH	12-18	2 h	<5 (69 SM)	<2	-
10	2	Et_3N-H_2O	12-18	<30 s	49 (51 SM)	7.2	86:14

[a]All reactions carried out using standard Schlenk techniques. [b]Quenched with air after the indicated time. [c]Determined by ^1H NMR. [d]Conversion to desired product is shown. The remaining starting material is shown in parentheses. [e]The corresponding cyclic 1,3-malonamide **5-SI** was used. See below for details. In all entries, SmI_2 prepared from Sm metal and ICH_2CH_2I was used.

Table SI-2. *Effect of Addition Sequence and Work-up Conditions on the Reduction of Cyclic 1,3-Diimides using SmI_2.*[a]

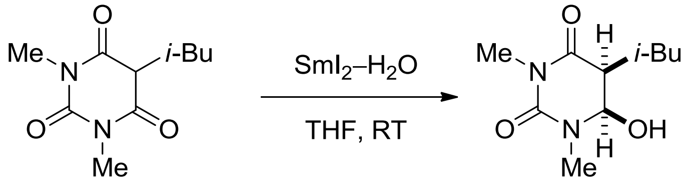

entry	SmI_2 (equiv)	H_2O (equiv)	time[b]	conv.[c] (%)	yield[c] (%)	dr[c]	addition/work-up
1	3	1000	5 min	91	81	88:12	Conditions A
2	3	1000	5 min	92	80	88:12	Conditions B
3	6	1000	3 min	>95	52	77:23	Conditions C

[a]All reactions carried out using standard Schlenk techniques. [b]Quenched with air after the indicated time. [c]Determined by ^{1}H NMR. [d]Addition and work-up conditions: A) Cyclic 1,3-diimide, followed by H_2O and SmI_2; work-up using 0.1 *M* HCl. B) SmI_2, followed by H_2O and cyclic 1,3-diimide; work-up using 0.1 *M* HCl. C) Cyclic 1,3-diimide, followed by SmI_2 and H_2O work-up using 1.0 *M* HCl. In all entries, SmI_2 prepared from Sm metal and ICH_2CH_2I was used.

Table SI-3. *Effect of SmI_2 Stoichiometry on the Reduction of Cyclic 1,3-Diimides using SmI_2.*[a]

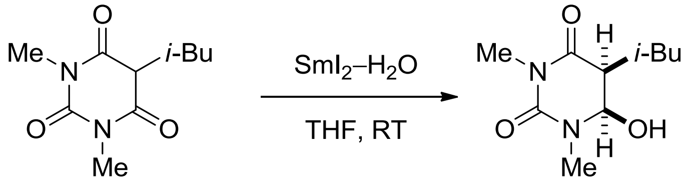

entry	SmI_2 (equiv)	H_2O (equiv)	time	conv. (%)	yield (%)	dr
1	3	1000	60 s	91	76	88:12
2	6	1000	10 s	>95	82	88:12
3	12	1000	15 min	>95	49	86:14

[a]All reactions carried out using standard Schlenk techniques. [b]Quenched with air after the indicated time. [c]Determined by ^{1}H NMR. Conditions: 1,3-Diimide, followed by H_2O and SmI_2; work-up using 0.1 *M* HCl. In all entries, SmI_2 prepared from Sm metal and ICH_2CH_2I was used.

Table SI-4. *Effect of H_2O Stoichiometry on the Reduction of Cyclic 1,3-Diimides using SmI_2.*[a]

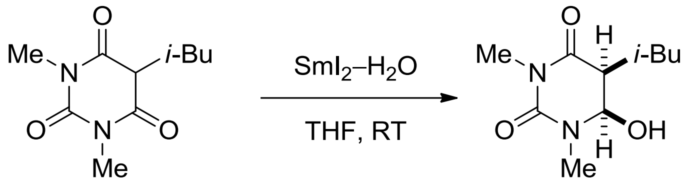

entry	SmI_2 (equiv)	H_2O (equiv)	time[b]	conv.[c] (%)	yield[c] (%)	dr[c]
1	6	-	24 h	<5	<5	-
2	4	10	2 h	24	<5	-
3	4	50	10 s	79	<5	-
4	4	200	10 s	84	57	82:18
5	4	1000	10 s	93	82	86:14
6	4	2500	10 s	66	49	88:12

[a]All reactions carried out using standard Schlenk techniques. [b]Quenched with air after the indicated time. [c]Determined by ^{1}H NMR. Conditions: 1,3-Diimide, followed by H_2O and SmI_2; work-up using 0.1 *M* HCl.

B) Studies on Mechanism of Reductive Cyclizations

Table SI-5. *Effect of Additives on the Reductive Cyclization of Cyclic 1,3-Diimides.*[a]

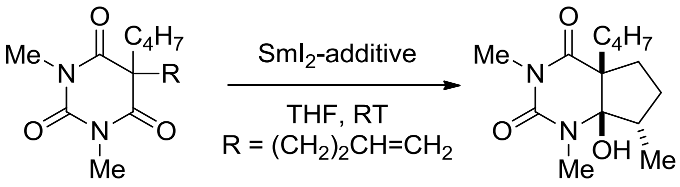

entry	SmI_2 (equiv)	additive	additive (equiv)	time[b]	conv.[c] (%)	yield[c] (%)	dr[c]
1	6	-	-	2 h	<5	<5	-
2	6	H_2O	10	2 h	<5	<5	-
3	6	H_2O	200	15 min	>95	78	-
4	6	MeOH	4/1 v/v	2 h	<5	<5	-
5	6	$HO(CH_2)_2OH$	36	15 min	30	16[d]	>95:5
6	6	LiCl	72	2 h	>95	25	69:31
7	6	HMPA	24	1 h	>95	<5	-

[a]All reactions carried out using standard Schlenk techniques. [b]Quenched with air after the indicated time. [c]Determined by ^{1}H NMR. [d]Reduction product formed in 8% yield corresponding to 67:33 ratio of cyclization to reduction. In other entries reduction was not observed. SmI_2 prepared from Sm metal and ICH_2CH_2I was used.

C) Selectivity of Monoreduction and Cyclization of Cyclic 1,3-Diimides

General Procedure. An oven-dried vial containing a stir bar was placed under a positive pressure of argon, and subjected to three evacuation/backfilling cycles under high vacuum. Samarium(II) iodide (THF solution, 0.10 mmol, 1.0 equiv, 0.10 M) was added followed by H_2O (0.18 mL, 200 equiv) with vigorous stirring, which resulted in the formation of a characteristic burgundy-red color of the $SmI_2(H_2O)_n$ complex (n > 5 with respect to SmI_2). A preformed solution of two substrates (each 0.10 mmol, 1.0 equiv, stock solution in THF, 1.0 mL) was added and the reaction mixture was stirred until decolorization to white had occurred. The reaction mixture was diluted with CH_2Cl_2 (30 mL) and HCl (1 *N*, 30 mL). The aqueous layer was extracted with CH_2Cl_2 (3 x 30 mL), and the organic layers were combined, dried over Na_2SO_4, filtered, and concentrated. The sample was analyzed by ^{1}H NMR ($CDCl_3$) and GC-MS to obtain conversion and yield using internal standard.

Table SI-6. *Selectivity Study in the Reduction of Cyclic 1,3-Diimides using SmI_2–H_2O.*[a]

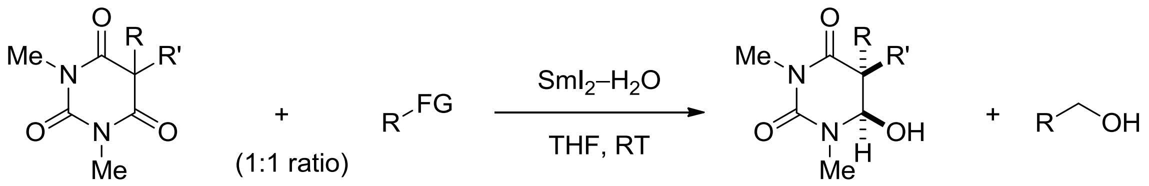

Substrate I **Substrate II** **I-red** **II-red**

entry	Substrate I	Substrate II	conv.[b] (**I-red**, %)	conv.[b] (**II-red**, %)	$k_I/k_{R\text{-}FG}$
1	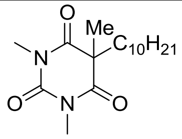	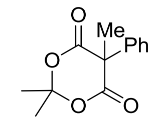	<2	13	<1:20
2	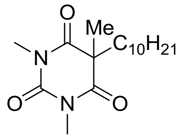	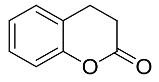	24	2	>20:1
3	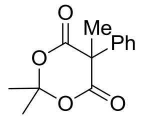	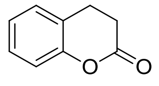	18	<2	>20:1
4	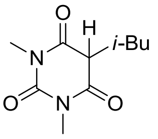	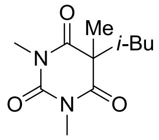	30	6.5	82:18
5	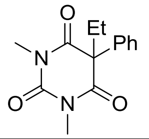	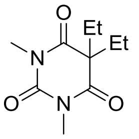	53	<2	>20:1

[a]Conditions: SmI_2 (1 equiv), H_2O (200 equiv), THF, room temperature, 10 s to 1 min. All reactions carried out using standard Schlenk techniques. [b]Determined by ^{1}H NMR (500 MHz) and/or GC-MS. Conversion = (100-SM). In all cases, rapid injection of substrate (THF solution) to the preformed SmI_2–H_2O complex was applied.

Table SI-7. *Selectivity Study in the Coupling of Cyclic 1,3-Diimides using SmI_2–H_2O.*[a]

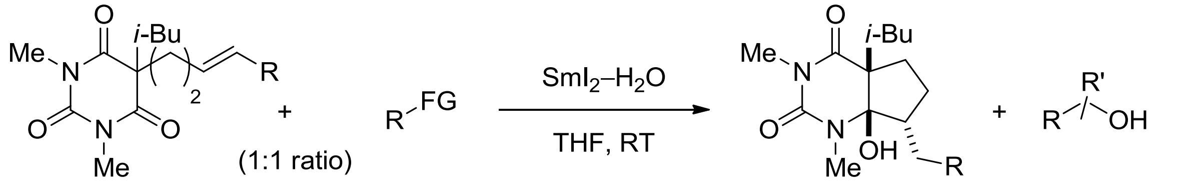

Substrate I | Substrate II | I-cycl | II-red/II-cycl

entry	Substrate I	Substrate II	conv.[b,c] (I-cycl, %)	conv.[b,c] (II-red/cycl, %)	k_I/k_{II}
1	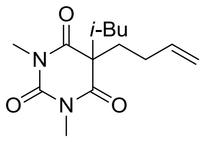	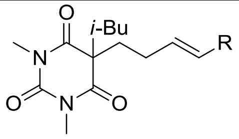 R = 4-MeO-C_6H_4	<2	33	<1:20
2	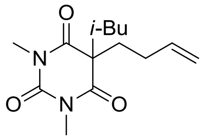	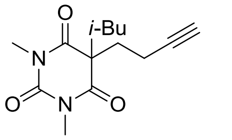	2.7	14	16:84
3	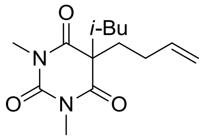	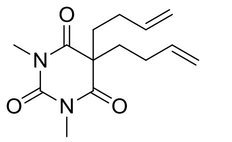	3.5	12	23:77
4	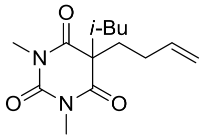	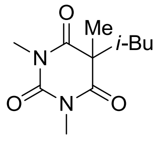	<2	23	<1:20
5	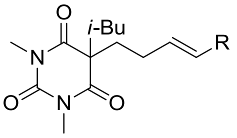 R = 4-MeO-C_6H_4	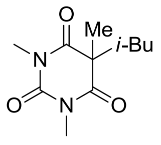	25	3.0	89:11

[a]Conditions: SmI_2 (1 equiv), H_2O (200 equiv), THF, room temperature, 10 s to 1 min. All reactions carried out using standard Schlenk techniques. [b]Determined by ^1H NMR (500 MHz) and/or GC-MS. Conversion = (100-SM). In all cases, rapid injection of substrate (THF solution) to the preformed SmI_2–H_2O complex was applied.

Selectivity studies have been carried out to determine electronic and steric factors that influence the rate of reduction of cyclic 1,3-diimides using the SmI_2–H_2O reagent. The data outlined in Tables SI-6 and SI-7 demonstrate three general trends and indicate that significant levels of selectivity are possible with this reagent system:

1) Chemoselectivity studies in the reduction of cyclic derivatives of carboxylic acids (Table SI-6, entries 1-3) illustrate that the SmI_2–H_2O system is selective for cyclic 1,3-diimides over lactones; however, cyclic 1,3-diesters are reduced preferentially. This outlines the following

reactivity scale for the SmI_2–H_2O system: Meldrum's acids > cyclic 1,3-diimides > lactones, which is in agreement with the stabilization of ketyl-type radicals in these systems. It is worthwhile to note that the SmI_2–H_2O system is fully chemoselective over acyclic carboxylic acid derivatives (esters, carboxylic acids, amides) in that no reduction of these functional groups is observed even if excess of the reagent is used.

2) The reduction of cyclic 1,3-diimides using SmI_2–H_2O is facilitated by electron withdrawing groups and slowed down by steric substitution at the alpha carbon (Table SI-6, entries 4-5). This is consistent with stabilization of the ketyl radical intermediate by electron withdrawing groups and reflects the importance of coordination of Sm(III) to the ketyl-type radical in the transition state of the reaction. Note that this trend allows high levels of chemoselectivity to be achieved in the reduction by careful fine-tuning of both the Sm(II) reagent system and steric/electronic substitution of the substrate.

3) The selectivity studies on reductive cyclizations of cyclic 1,3-diimides using SmI_2–H_2O show that the cyclization rate is governed by electronic and steric properties of the π-acceptor (Table SI-7, entries 1-5). This is in agreement with the proposed mechanism and consistent with the previous findings on related stereoselective radical cyclizations.[26-28] Overall, these general trends further indicate that chemoselectivity levels unattainable via ionic reaction pathways are accessible with Sm(II) reductants.

D) Deuterium Incorporation and Kinetic Isotope Effect Studies[20-24]

Scheme SI-1. *Determination of Deuterium Incorporation and Kinetic Isotope Effect.*

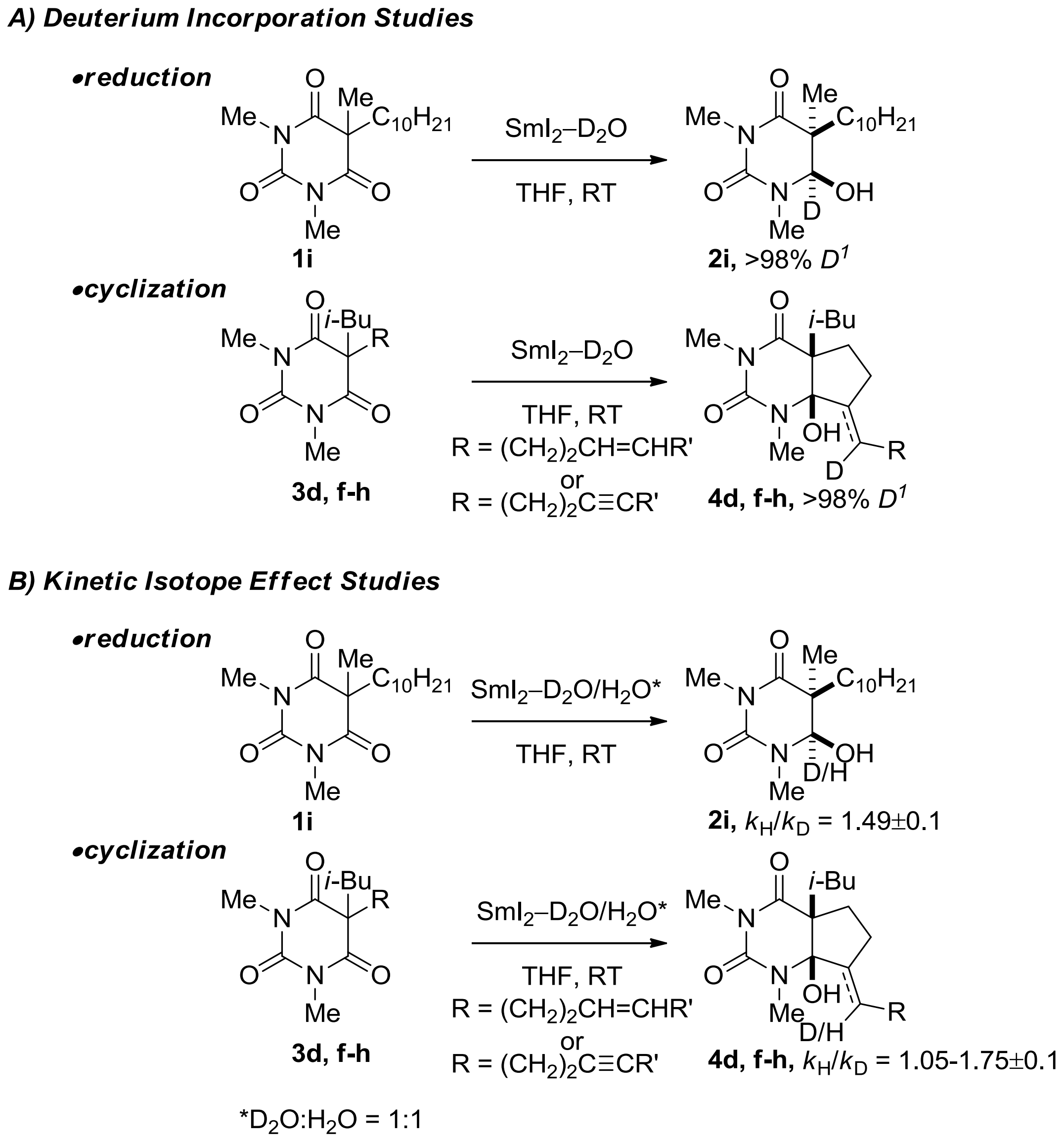

<u>General Procedure.</u> According to the general procedure for monoreduction or reductive cyclization, a cyclic 1,3-diimide (0.05-0.10 mmol) was reacted with SmI_2 (3-6 equiv), and D_2O (200-1000 equiv, deuterium incorporation) or D_2O/H_2O (200-1000 equiv, 1:1, stock solution, KIE) for the indicated time at rt. After the standard work-up, the reaction mixture was diluted with CH_2Cl_2 (30 mL) and HCl (1 *N*, 30 mL). The aqueous layer was extracted with CH_2Cl_2 (3 x 30 mL), and the organic layers were combined, dried over Na_2SO_4, filtered, and concentrated. The sample was analyzed by ^1H NMR ($CDCl_3$) and/or GC-MS to obtain deuterium incorporation. Characterization data for all new compounds is given in the Experimental Part of the Supporting Information.

E) Evidence for Isomerization of Vinyl Radicals[25-27]

Table SI-8. *Evidence for Isomerization of Vinyl Radicals under SmI_2–H_2O Conditions.* [a]

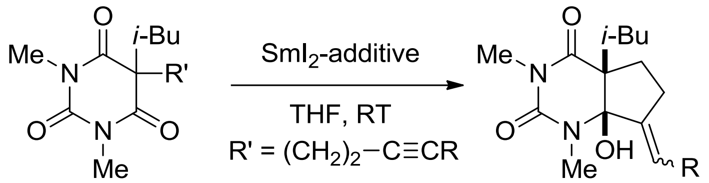

entry	SmI_2 (equiv)	R	H_2O (equiv)	time[b]	conv.[c] (%)	yield[c] (%)	dr[c] (*E*:*Z*)
1	6	Ph	50	15 min	>95	99	58:42
2	6	Ph	200	15 min	>95	99	37:63
3	6	Ph	2400	15 min	>95	99	24:76
4	6	TMS	200	15 min	>95	89	>95:5
5	6	H[d]	200	15 min	>95	85	68:32
6	6	Ph[d]	50	15 min	>95	90	60:40
7	6	TMS[d]	200	15 min	>95	87	>95:5

[a]All reactions carried out using standard Schlenk techniques. [b]Quenched with air after the indicated time. [c]Determined by ^1H NMR. [d]Reaction carried out using D_2O instead of H_2O. SmI_2 prepared from Sm metal and ICH_2CH_2I was used.

Reductive cyclizations of cyclic 1,3-diimides have been carried out at different concentrations of H_2O and with the SmI_2–D_2O system to probe the stability of radical intermediates formed after initial reductive cyclization (Table SI-8). Alkyne tethers have been chosen as mechanistic probes because of the well-established propensity of related radicals to undergo anti radical cyclizations as well as the stability of the resulting vinyl radicals towards isomerization (inversion barrier of ca. 2 kcal/mol).[25-28] In the cyclizations of the phenyl-containing substrate (Table SI-8, entries 1-3), a gradual change in diastereoselectivity is observed at varied concentrations of H_2O. This suggests that the carbon-centered radicals formed in the reductive cyclizations mediated by SmI_2–H_2O do not undergo instantaneous reduction/protonation despite the presence of a thermodynamically powerful reductant (SmI_2–H_2O: -1.9 V vs $Ag/AgNO_3$)[29] and a large excess of the proton source. In the cyclizations of the TMS- and H-containing substrates (Table SI-8, entries 4-5) full and partial inversion of the vinyl radical is observed under the reduction conditions, respectively. The inversion in the first two substrates is also observed in the reactions mediated by SmI_2–D_2O

(Table SI-8, entries 6-7). Overall, the results in Table SI-8 indicate that the extent of isomerization is governed by steric and electronic properties of the π-acceptor (Ph, TMS, H) and the concentration of H_2O co-solvent (a thermodynamically more powerful reductant is formed at higher concentrations of H_2O)[29] (entries 1-3). Application of the above findings to the cascade processes employing C-centered radicals is currently underway in our laboratory and these results will be reported shortly.

F) Additional Selectivity Studies

Scheme SI-2. *Additional Selectivity Studies in the Reduction of Cyclic 1,3-Diimides.*

A) Reduction of cyclic urea

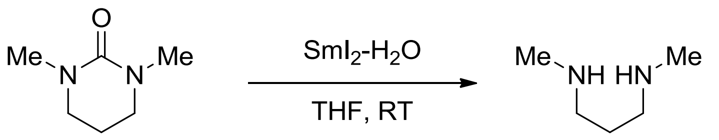

SI-3 **SI-4**, <5% conv. (eq 1)

B) Reduction of cyclic 1,3-malonamide

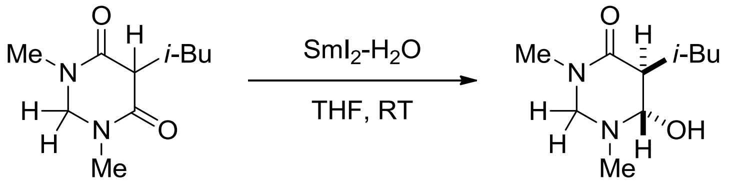

SI-5 **SI-6**, <5% conv. (eq 2)

Eq. 1. According to the general procedure for reduction of cyclic 1,3-diimides with SmI_2-H_2O, 1,3-dimethyltetrahydropyrimidin-2(1*H*)-one (0.10 mmol) was reacted with SmI_2 (0.40 mmol, 4 equiv) and H_2O (200 equiv) for 2 h at room temperature, which resulted in the formation of a characteristic burgundy-red color of the $SmI_2(H_2O)_n$ complex (n > 5 with respect to SmI_2). After the standard work-up as described above, the sample was analyzed by ^{1}H NMR to obtain conversion and yield using internal standard: conversion <5%; yield of recovered starting material: >80%.

Eq. 2. According to the general procedure for reduction of cyclic 1,3-diimides with SmI_2-H_2O, 5-isobutyl-1,3-dimethyldihydropyrimidine-4,6(1*H*,5*H*)-dione (0.10 mmol) was reacted with SmI_2 (0.60 mmol, 6 equiv) and H_2O (1000 equiv) for 1 h at room temperature, which resulted in the formation of a characteristic burgundy-red color of the $SmI_2(H_2O)_n$ complex (n > 5 with respect to SmI_2). After the standard work-up as described above, the sample was analyzed by ^{1}H NMR to obtain conversion and yield using internal standard: conversion <5%; yield of recovered starting material: >95%. In another optimization run, a reaction

using SmI_2 (6 equiv) and H_2O (200 equiv) for 1 h at room temperature resulted in <5% conversion.

5-Isobutyl-1,3-dimethyldihydropyrimidine-4,6(1*H*,5*H*)-dione (SI-5). To a 50 mL round-bottomed flask charged with 1,3-dimethyl-5-(2-methylpropylidene)pyrimidine-2,4,6(1*H*,3*H*,5*H*)-trione prepared as described above (0.80 g, 3.8 mmol, 1.0 equiv) and THF (20 mL), DIBAL-H (1.0 M in hexanes, 20 mL, 4.0 equiv) was added dropwise at -78 °C. The reaction mixture was allowed to slowly warm up to room temperature over 15 h, quenched with an aqueous saturated solution of sodium potassium tartrate, extracted with ethyl acetate, concentrated and dried. Purification by chromatography using EtOAc/hexanes (70/30) afforded the title compound as a white solid (mp = 32-34 °C). Yield 68% (0.51 g). R_f (70% EtOAc/hexanes) = 0.11. ^{1}H NMR (400 MHz, $CDCl_3$) δ 0.87 (d, J = 6.4 Hz, 6 H), 1.68 (t, J = 6.8 Hz, 2 H), 1.70-1.81 (m, 1 H), 2.97 (s, 6 H), 3.05 (t, J = 6.8 Hz, 1 H), 4.49 (d, J = 8.7 Hz, 1 H), 4.53 (d, J = 8.4 Hz, 1 H); ^{13}C NMR (100 MHz, $CDCl_3$) δ 22.4, 26.0, 32.7, 35.8, 48.4, 63.2, 168.7. IR (neat) 2955, 2923, 2866, 1657, 1485, 1439, 1401, 1369, 1264, 1224, 1102, 1064, 787 cm^{-1}. HRMS calcd for $C_{10}H_{19}N_2O_2$ (M^+ + H) 199.1442, found 199.1446.

Structural Characterization and Additional Discussion

Figure A. *ORTEP Structure of* ***4a*** *(CCDC 948382).*

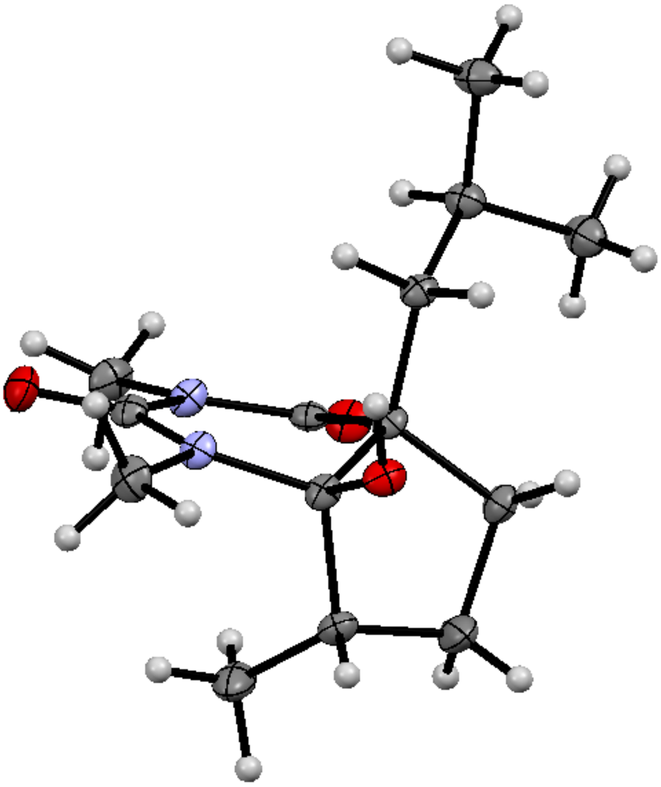

Figure B. *ORTEP Structure of* ***2f*** *(CCDC 948381).*

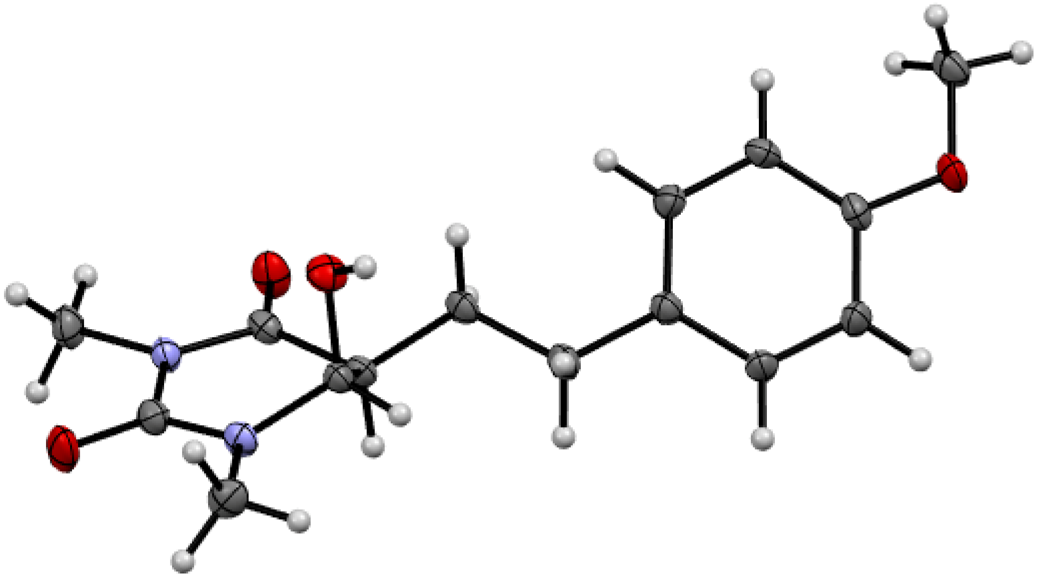

Additional discussion. The alpha-amino alcohol moiety is stabilized by a nonplanar arrangement of atoms. The X-ray crystal structure of **4a** reveals that the C1–O1 bond (1.407 Å) is shorter than the average Cs_{p3}–O bond (1.432 Å), while the N1–C1 bond is 1.466 Å, which corresponds to a typical C_{sp3}–N bond (1.469 Å). The C1–C4 bond length of 1.552 Å is slightly longer than the average C_{sp3}-C_{sp3} bond (1.530 Å). The torsion angle between N_{lp} and C1–O1 of 57.3° is consistent with the absence of $N_{lp}\rightarrow\sigma^*_{C–O}$ interactions in this system. However, there exists a good overlap between $O1_{lp1}$ and the N1–C1 bond (~172°) and between $O1_{lp2}$ and the C1–C4 bonds (~191°). The shortened C1–O1 bond and the elongated C1–C4 bond are consistent with an anomeric effect resulting from $O_{lp}\rightarrow\sigma^*_{C1–N1}$ and $O_{lp}\rightarrow\sigma^*_{C1–C4}$ interactions, while the geometry of N1 atom (N_{lp}, N1–C1 bond length) indicates the beginning of the decomposition of the tetrahedral intermediate by the elimination of N(CO) group to give the open N-acyl urea derivative. Nevertheless, it should be noted that the α-amino alcohol function in this system is stabilized by the reduced $N_{lp}\rightarrow\sigma^*_{C–O}$ conjugation by the interaction with the adjacent carbonyl group. This is further indicated by the co-planarity of all six atoms comprising the uracil ring, with the C1 atom deviated from the plane of the ring by 6.9° and C2 by –14.6°.

The X-ray structure of a mono-cyclic analogue **2i** reveals kinetic rather than thermodynamic stability (*vide supra*). The C1–O1 bond in **2i** of 1.411 Å and the C1–N1 bond of 1.458 Å are in a similar range as for the bicyclic derivative **4a** described above. However, the C1–C2 bond length of 1.519 Å is slightly shorter than the average C_{sp3}–C_{sp3} bond (1.530 Å). The torsion angles between N_{lp} and C1–O1 of ~175° and O_{lp} and C1–N1 of ~137° indicate a significant $N_{lp}\rightarrow\sigma^*_{C1–O1}$ interaction in this system, and the absence of $O_{lp}\rightarrow\sigma^*_{C1–N1}$ conjugation. There exists a reasonably good arrangement between $O_{lp}\rightarrow\sigma^*_{C1–C2}$ (torsion angle of ~163°) and there is no evidence for anomeric effect involving $O_{lp}\rightarrow\sigma^*_{C1–H}$ (torsion angle of ~137°). Finally, the O1–C1–C2–H2 torsion angle of ~180° reveals a perfect antiperiplanar arrangement between the alpha hydrogen atom and the hydroxyl group. These parameters are consistent with the beginning of the decomposition of the alpha amino alcohol moiety by the elimination of hydroxyl group to give the acyliminium. The structural differences between **4a** and **2i** are further emphasized by the bond lengths of the alpha hydroxyl N-acyl urea moiety (**4a**, distal N–C(O) bond length of 1.421 Å, C=O of 1.218 Å; **2i**, distal N-C(O) bond length of 1.396 Å, C=O of 1.237 Å).

References

1. P. Girard, J. L. Namy and H. B. Kagan, *J. Am. Chem. Soc.*, **1980**, *102*, 2693.
2. T. Imamoto and M. Ono, *Chem. Lett.*, **1987**, 501.
3. A. Dáhlen and G. Hilmersson, *Eur. J. Inorg. Chem.*, **2004**, 3020.
4. J. A. Teprovich, Jr., P. K. S. Antharjanam, E. Prasad, E. N. Pesciotta and R. A. Flowers, II, *Eur. J. Inorg. Chem.*, **2008**, 5015.
5. M. Szostak, M. Spain and D. J. Procter, *J. Org. Chem.*, **2012**, *77*, 3049.
6. C. Löfberg, R. Grigg, A. Keep, A. Derrick, V. Sridharan and C. Kilner, *Chem. Commun.*, **2006**, 5000.
7. B. S. Jursic and E. D. Stevens, *Tetrahedron Lett.*, **2003**, *44*, 2203.
8. A. C. Cope, D. Heyl, D. Peck, C. Eide and A. Arroyo, *J. Am. Chem. Soc.*, **1941**, *63*, 356.
9. M. Sekiya and C. Yanaihara, *Chem. Pharm. Bull.*, **1969**, *17*, 747.
10. M. L. Bolte, W. L. Crow and S. Yoshida, *Aust. J. Chem.*, **1982**, *35*, 1411.
11. E. Marom, M. Mizhiritskii and S. Rubnov, WO 2010/109468 A1.
12. S. Kotha, A. C. Deb and R. V. Kumar, *Bioorg. Med. Chem. Lett.*, **2005**, *15*, 1039.
13. (a) Condensation: M. L. Bolte, W. L. Crow and S. Yoshida, *Aust. J. Chem.*, **1982**, *35*, 1411. (b) Reduction: Y. Isobe and K. Hirota, *Chem. Pharm. Bull.*, **2003**, *51*, 1451.
14. C. F. Nutaitis, R. A. Schultz, J. Obaza and F. X. Smith, *J. Org. Chem.*, **1980**, *45*, 4606.
15. (a) Condensation: E. Marom, M. Mizhiritskii and S. Rubnov, WO 2010/109468 A1. (b) Reduction: C. F. Nutaitis, R. A. Schultz, J. Obaza and F. X. Smith, *J. Org. Chem.*, **1980**, *45*, 4606.
16. Y. Frangin, C. Guimbal, F. Wissocq and H. Zamarlik, *Synthesis*, **1986**, 1046.
17. L. F. Tietze, C. Ott, H. Geißler and F. Haunert, *Eur. J. Org. Chem.*, **2001**, 16252.
18. K. T. Wong and Y. Y. Hung, *Tetrahedron Lett.*, **2003**, *44*, 8033.
19. D. B. Ramachary, M. Kishor and Y. V. Reddy, *Eur. J. Org. Chem.*, **2008**, 975.
20. A. Dáhlen and G. Hilmersson, *Tetrahedron Lett.*, **2001**, *42*, 5565.
21. A. Dáhlen and G. Hilmersson, *Chem. Eur. J.*, **2003**, *9*, 1123.
22. D. Parmar, L. A. Duffy, D. V. Sadasivam, H. Matsubara, P. A. Bradley, R. A. Flowers, II and D. J. Procter, *J. Am. Chem. Soc.*, **2009**, *131*, 15467.
23. M. Szostak, M. Spain and D. J. Procter, *Chem. Commun.*, **2011**, *47*, 10254.
24. E. M. Simmons and J. F. Hartwig, *Angew. Chem. Int. Ed.*, **2012**, *51*, 3066.
25. R. W. Fessenden and R. H. Schuler, *J. Chem. Phys.*, **1963**, *39*, 2147.

26. D. P. Curran, N. A. Porter and B. Giese, *Stereochemistry of Radical Reactions*; Wiley-VCH: 1996.
27. B. Giese, *Angew. Chem. Int. Ed.*, **1983**, *22*, 753.
28. C. Leroy, D. Peeters and C. Wilante, *THEOCHEM*, 1982, **5**, 217.
29. E. Prasad and R. A. Flowers, II, *J. Am. Chem. Soc.*, **2005**, *127*, 18093.

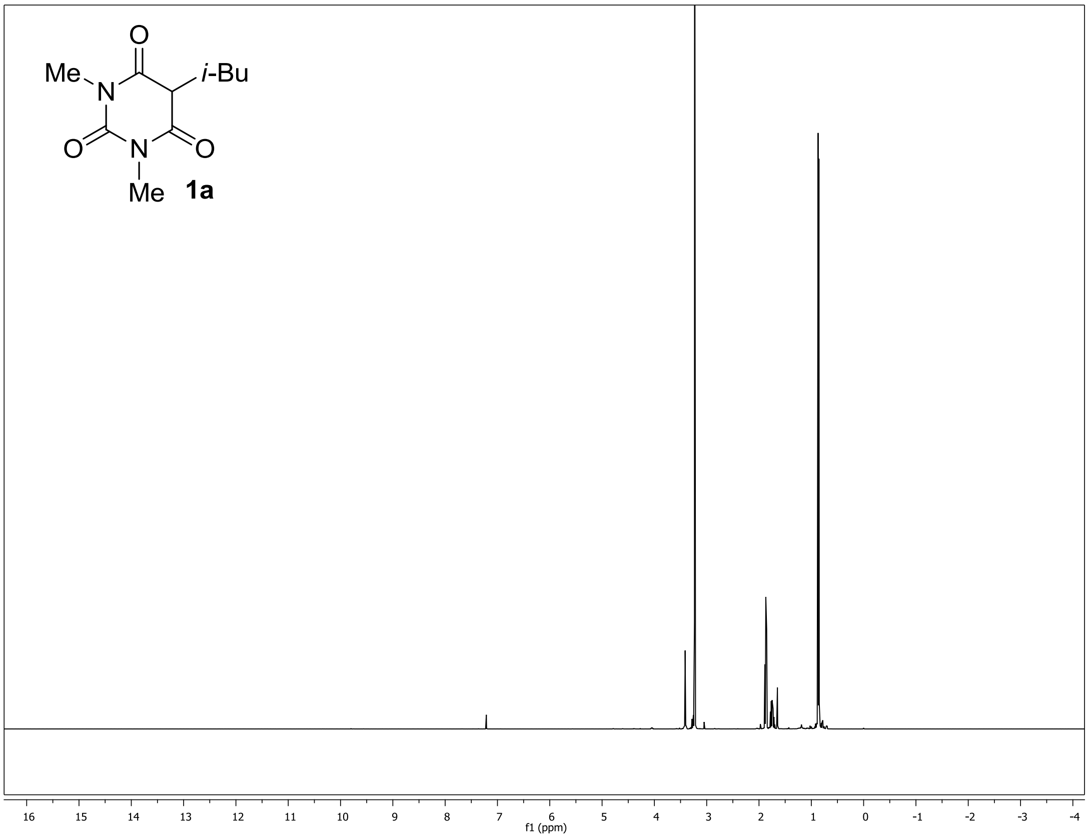
Me
N
i-Bu
O
N
O
O
Me
1a
16 15 14 13 12 11 10 9 8 7 6 5 4 3 2 1 0 -1 -2 -3 -4
f1 (ppm)

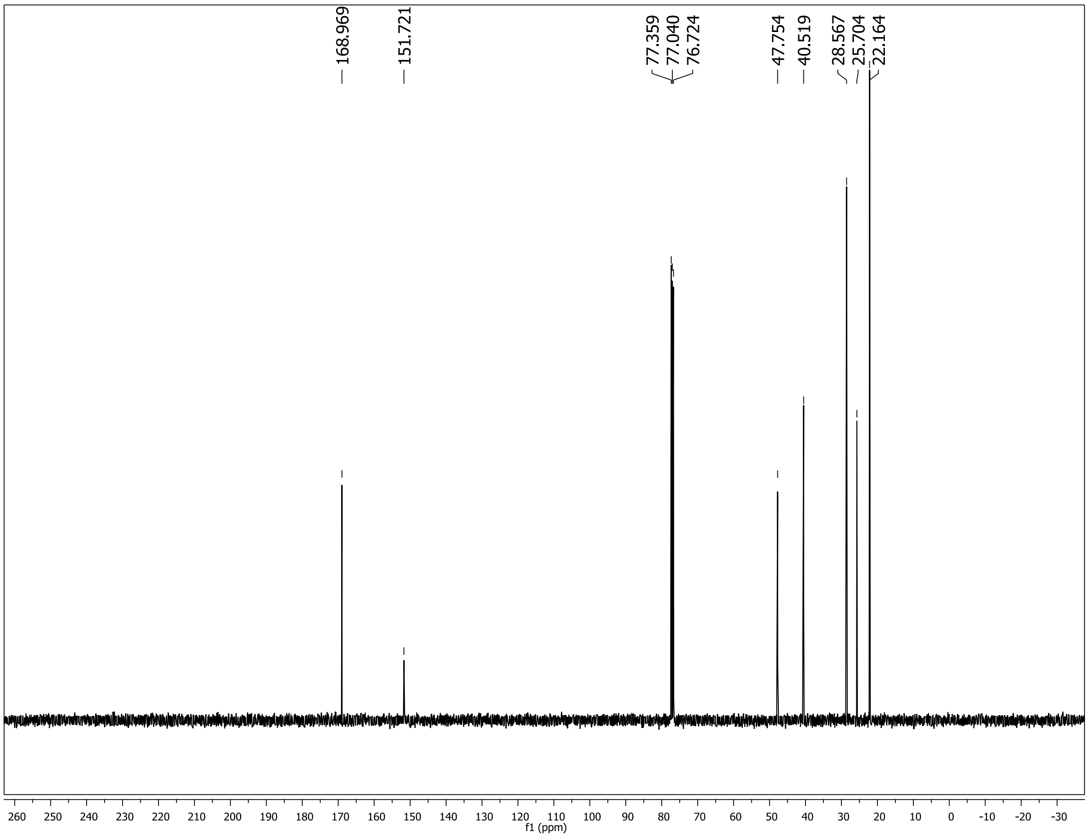
168.969
151.721
77.359
77.040
76.724
47.754
40.519
28.567
25.704
22.164
260 250 240 230 220 210 200 190 180 170 160 150 140 130 120 110 100 90 80 70 60 50 40 30 20 10 0 -10 -20 -30
f1 (ppm)

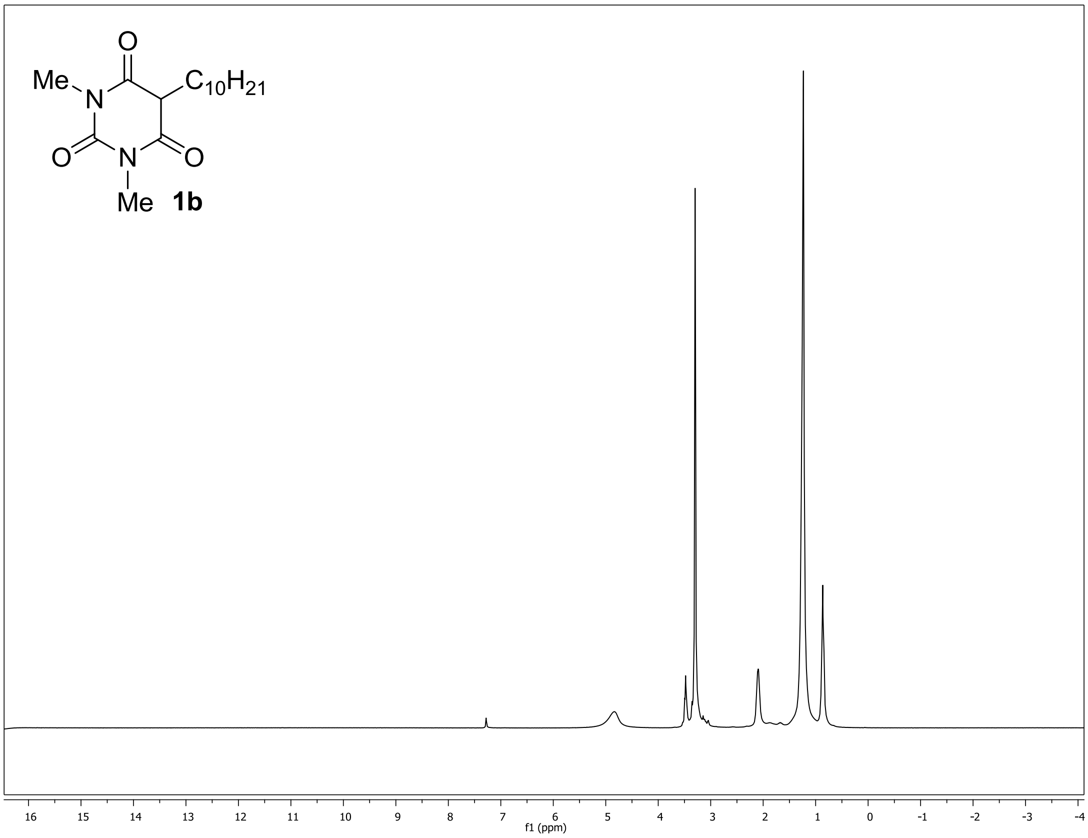
Me
N
O
C10H21
O
N
O
Me
1b
16 15 14 13 12 11 10 9 8 7 6 5 4 3 2 1 0 -1 -2 -3 -4
f1 (ppm)

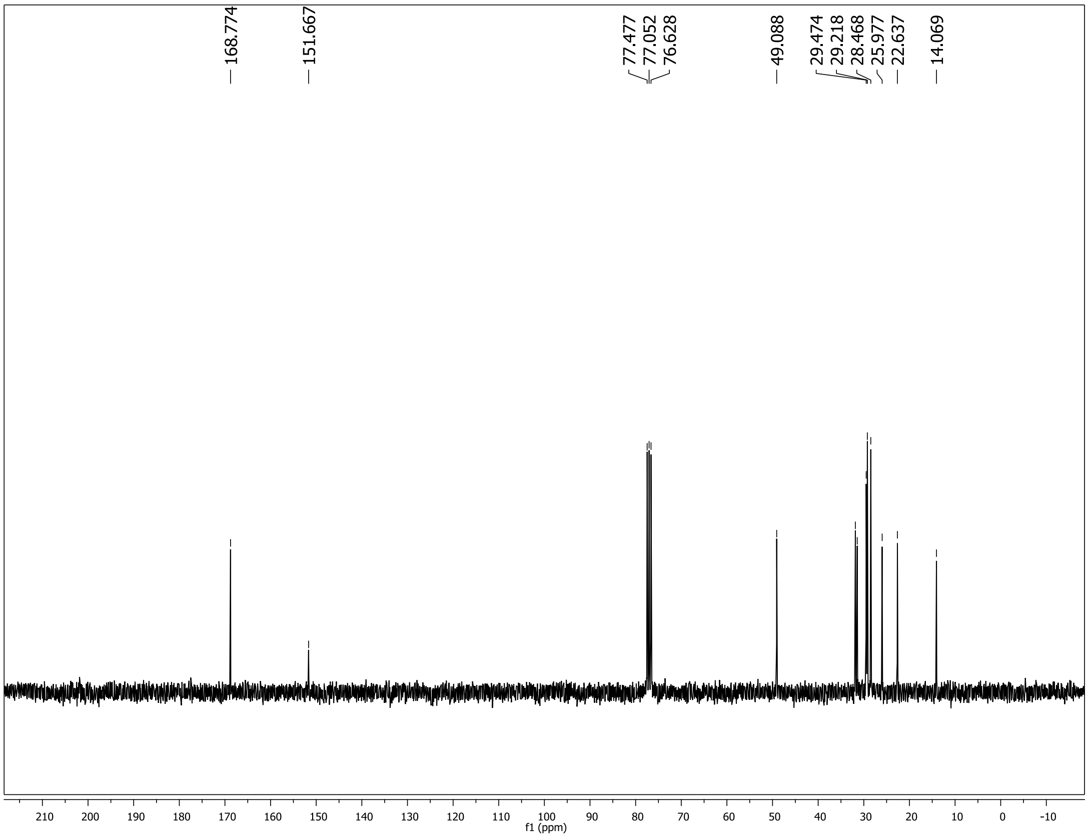
168.774
151.667
77.477
77.052
76.628
49.088
29.474
29.218
28.468
25.977
22.637
14.069
210 200 190 180 170 160 150 140 130 120 110 100 90 80 70 60 50 40 30 20 10 0 -10
f1 (ppm)

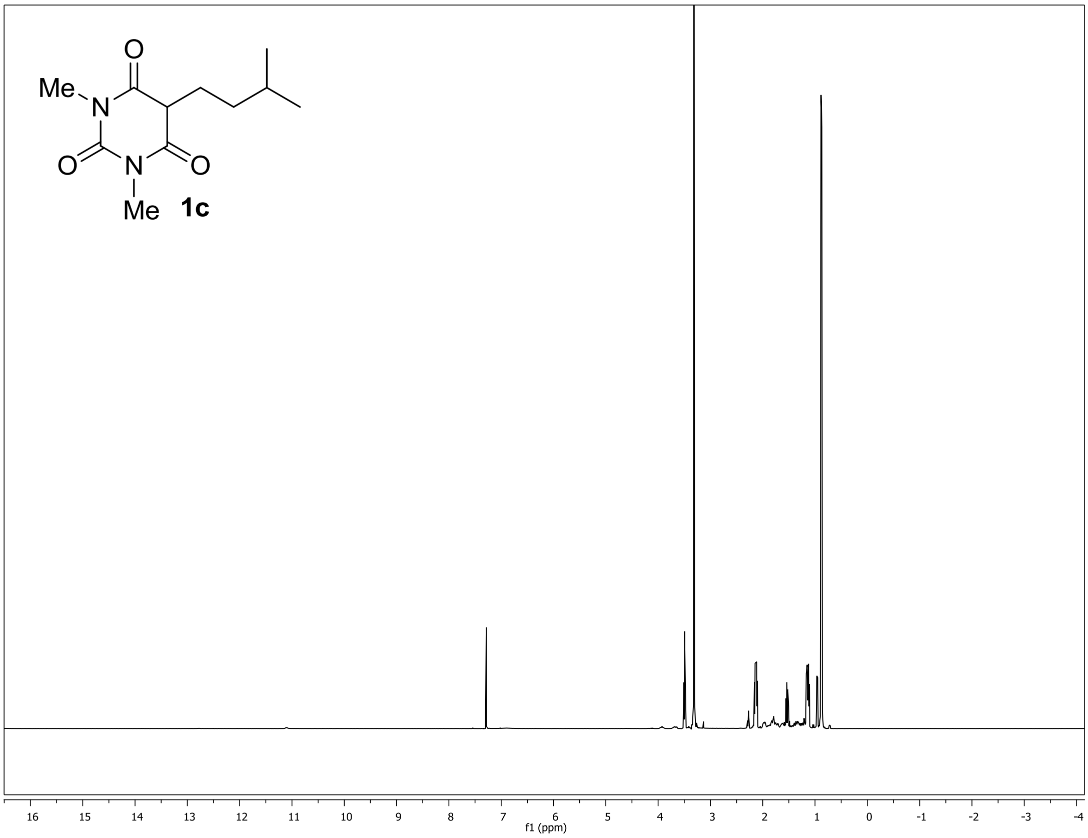
Me
N
O
O
N
O
Me
1c
f1 (ppm)

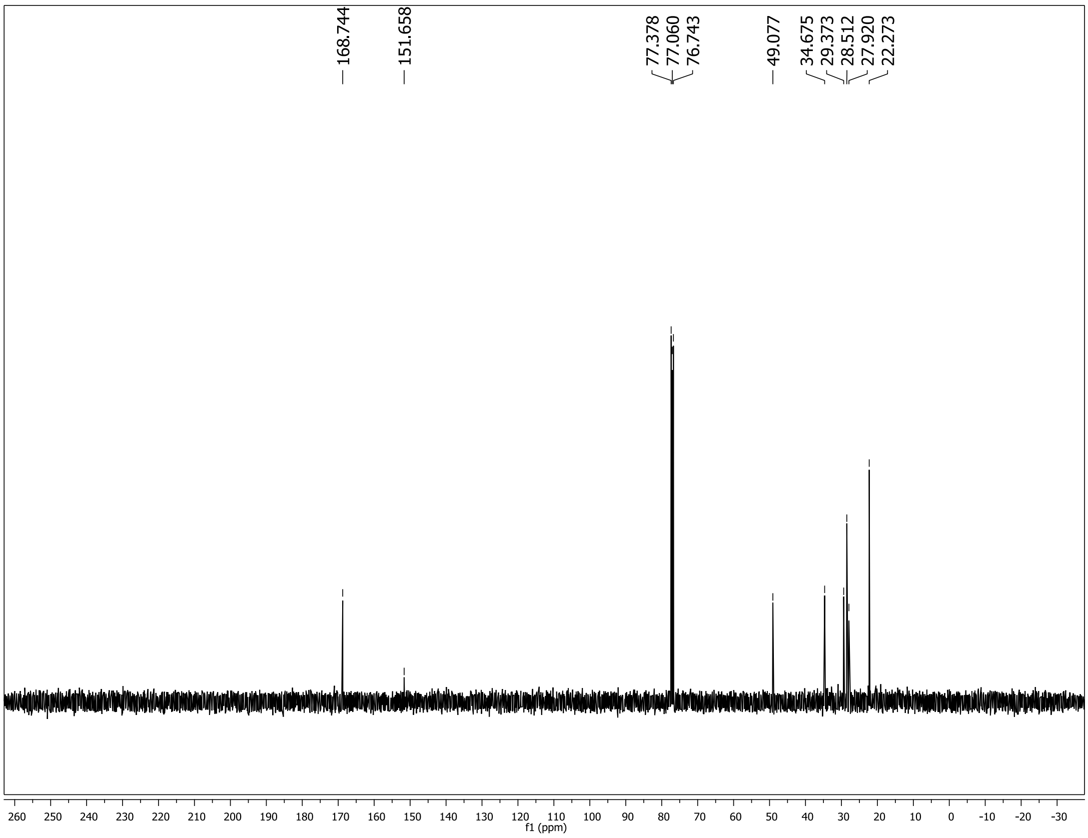
168.744
151.658
77.378
77.060
76.743
49.077
34.675
29.373
28.512
27.920
22.273
f1 (ppm)

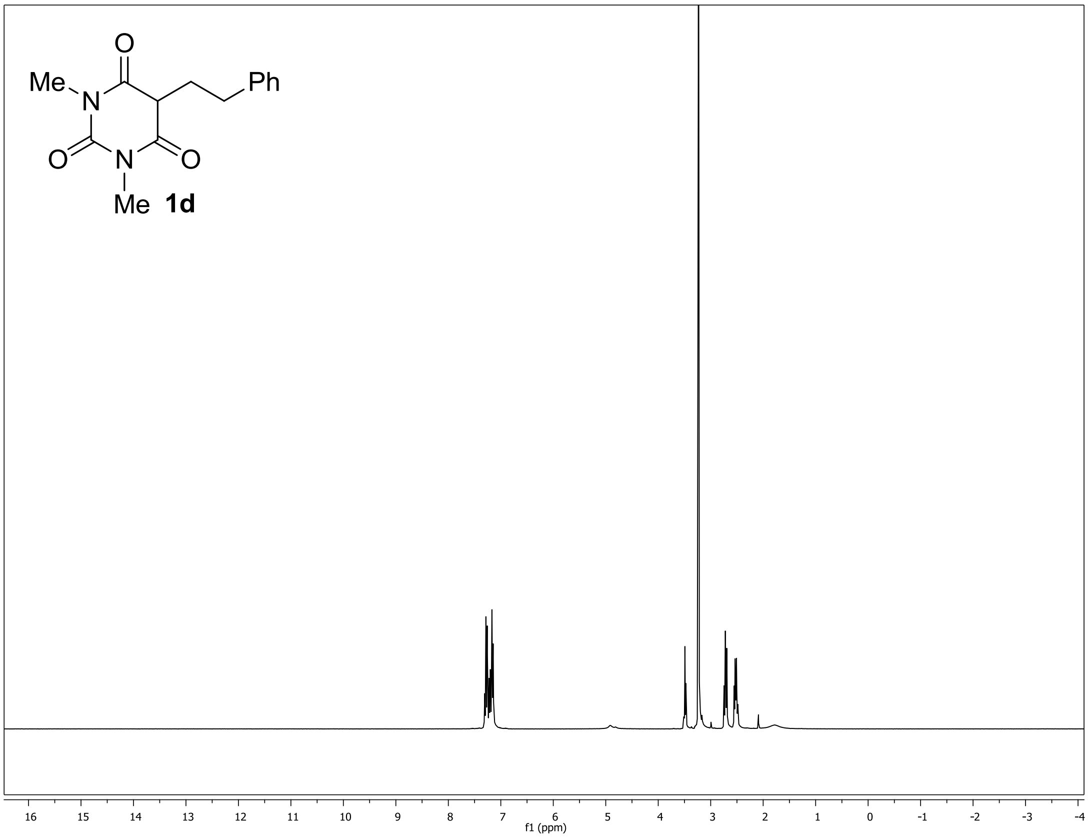
Me
N
O
Ph
N
Me 1d
f1 (ppm)

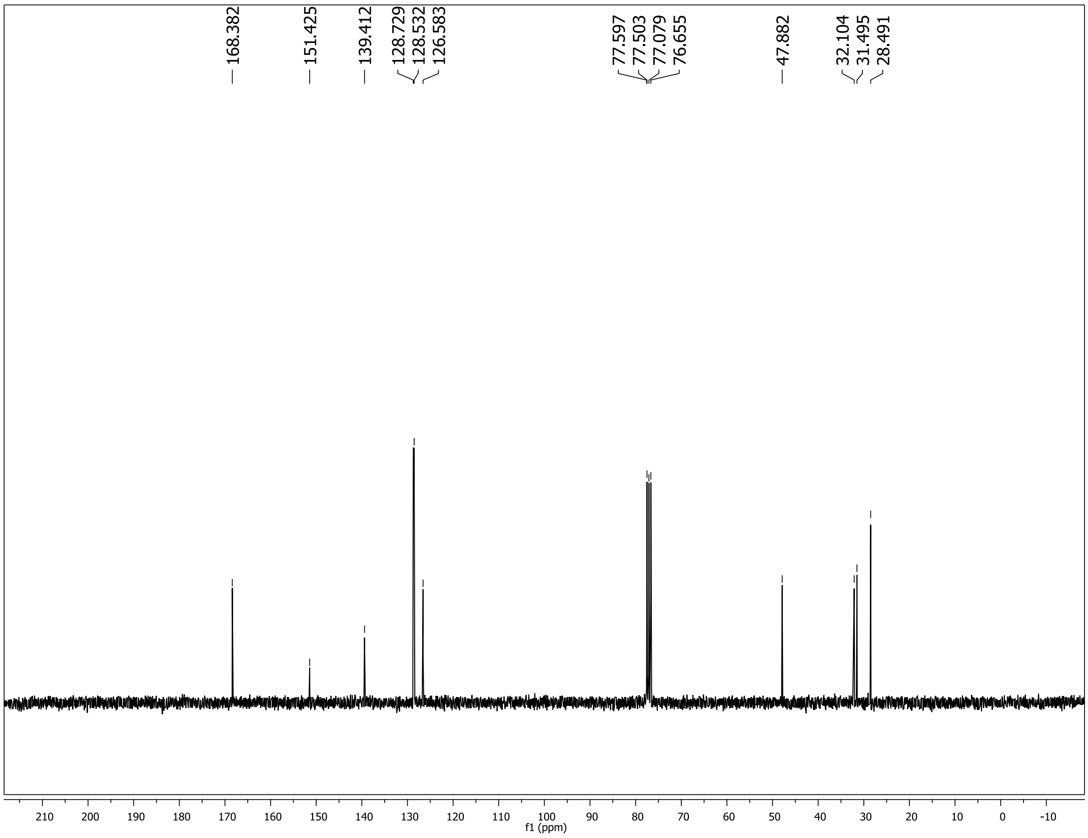
168.382
151.425
139.412
128.729
128.532
126.583
77.597
77.503
77.079
76.655
47.882
32.104
31.495
28.491
f1 (ppm)

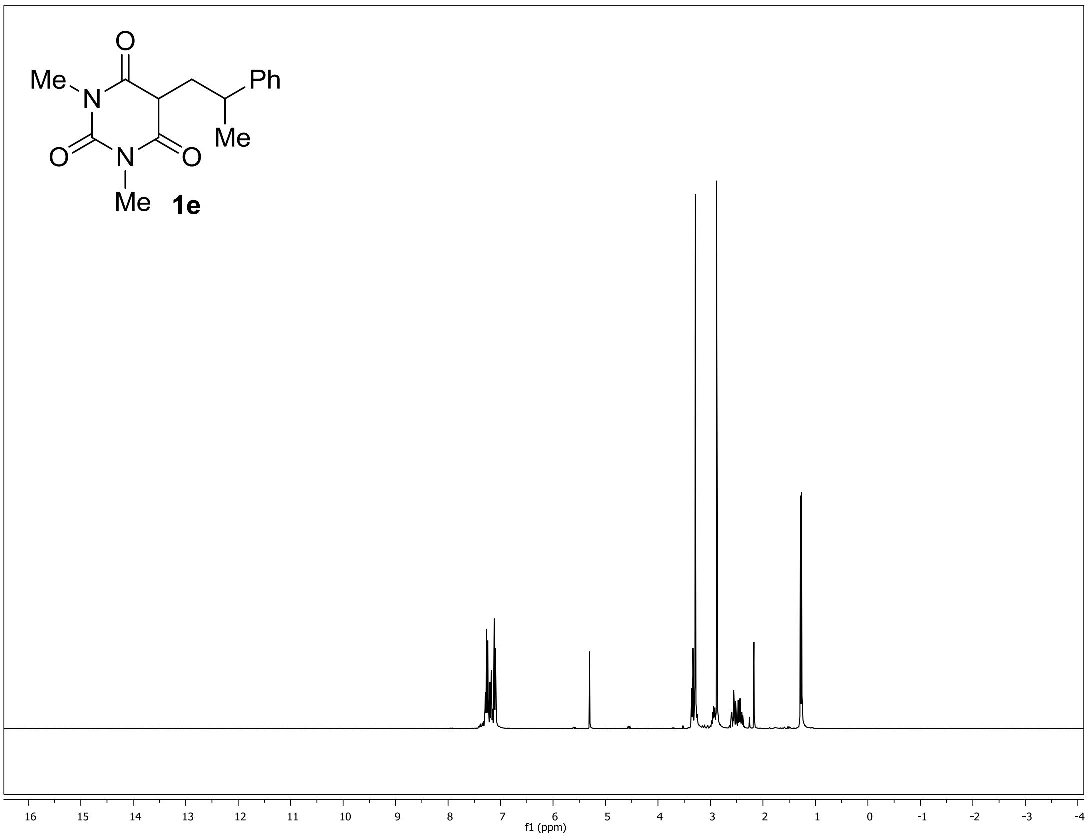
Me
N
O
Ph
Me
1e
16 15 14 13 12 11 10 9 8 7 6 5 4 3 2 1 0 -1 -2 -3 -4
f1 (ppm)

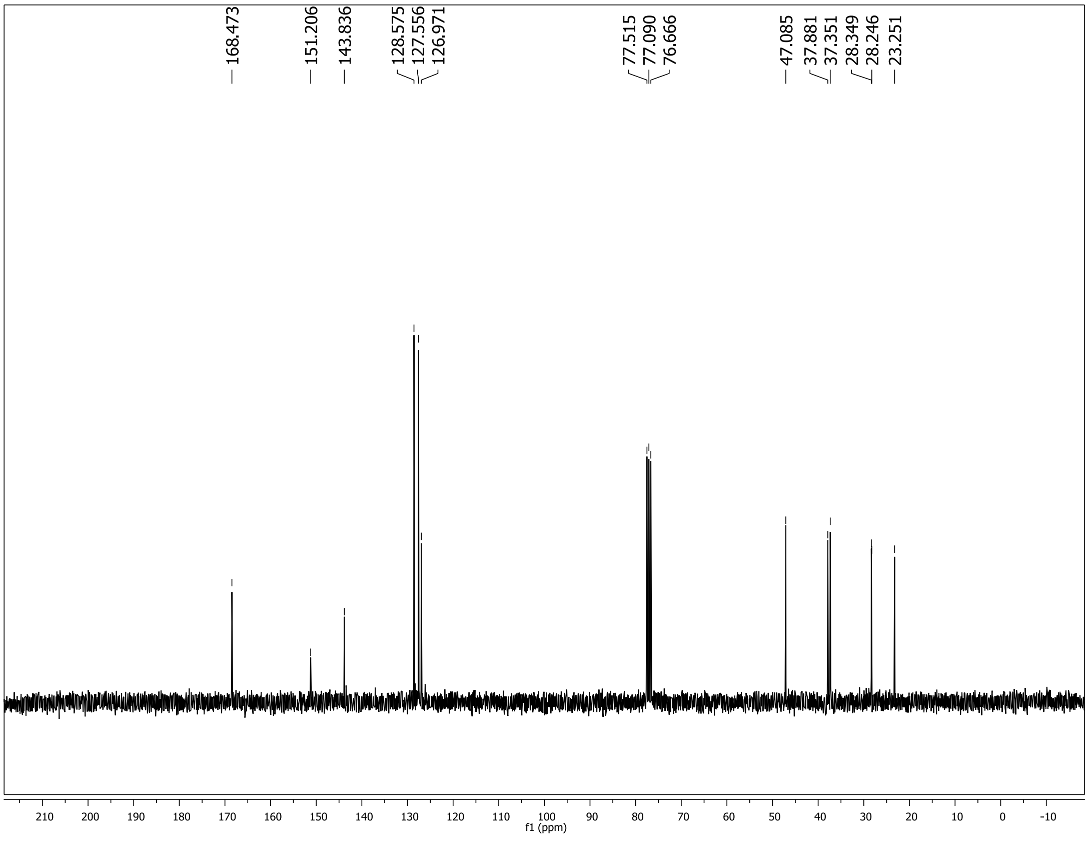
168.473
151.206
143.836
128.575
127.556
126.971
77.515
77.090
76.666
47.085
37.881
37.351
28.349
28.246
23.251
210 200 190 180 170 160 150 140 130 120 110 100 90 80 70 60 50 40 30 20 10 0 -10
f1 (ppm)

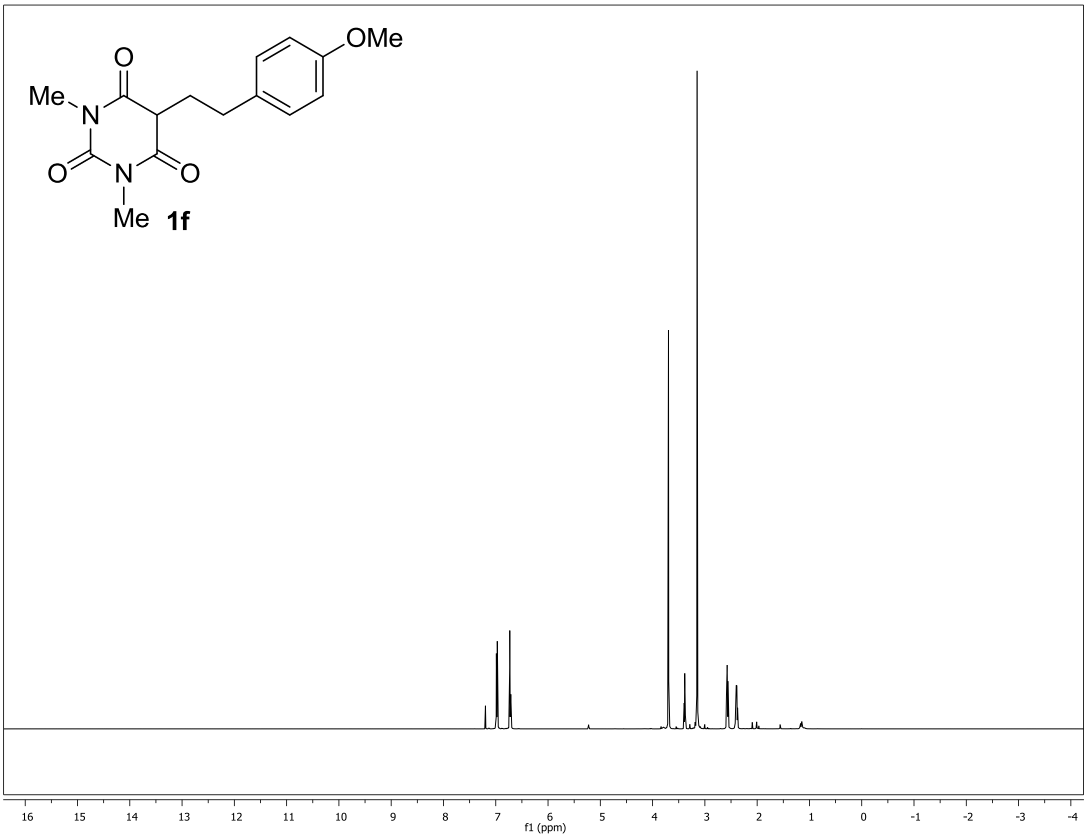
OMe
O
Me
N
O
N
O
Me
1f
16
15
14
13
12
11
10
9
8
7
6
5
4
3
2
1
0
-1
-2
-3
-4
f1 (ppm)

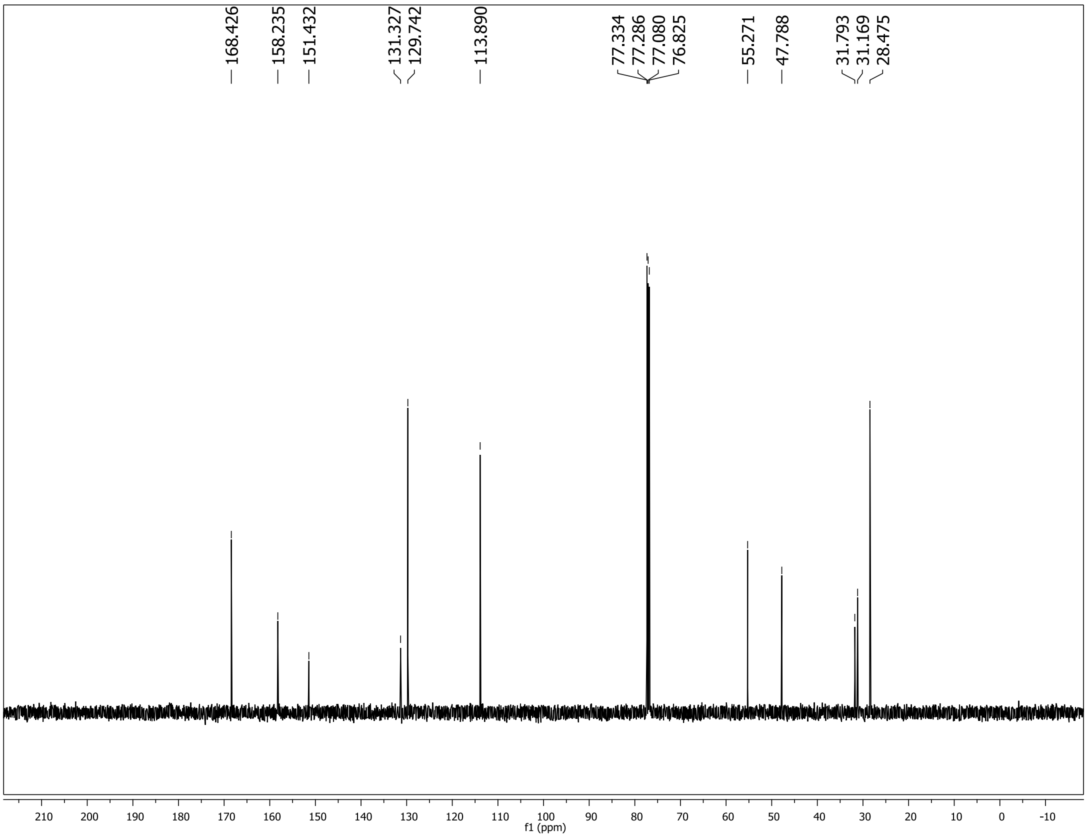
168.426
158.235
151.432
131.327
129.742
113.890
77.334
77.286
77.080
76.825
55.271
47.788
31.793
31.169
28.475
210
200
190
180
170
160
150
140
130
120
110
100
90
80
70
60
50
40
30
20
10
0
-10
f1 (ppm)

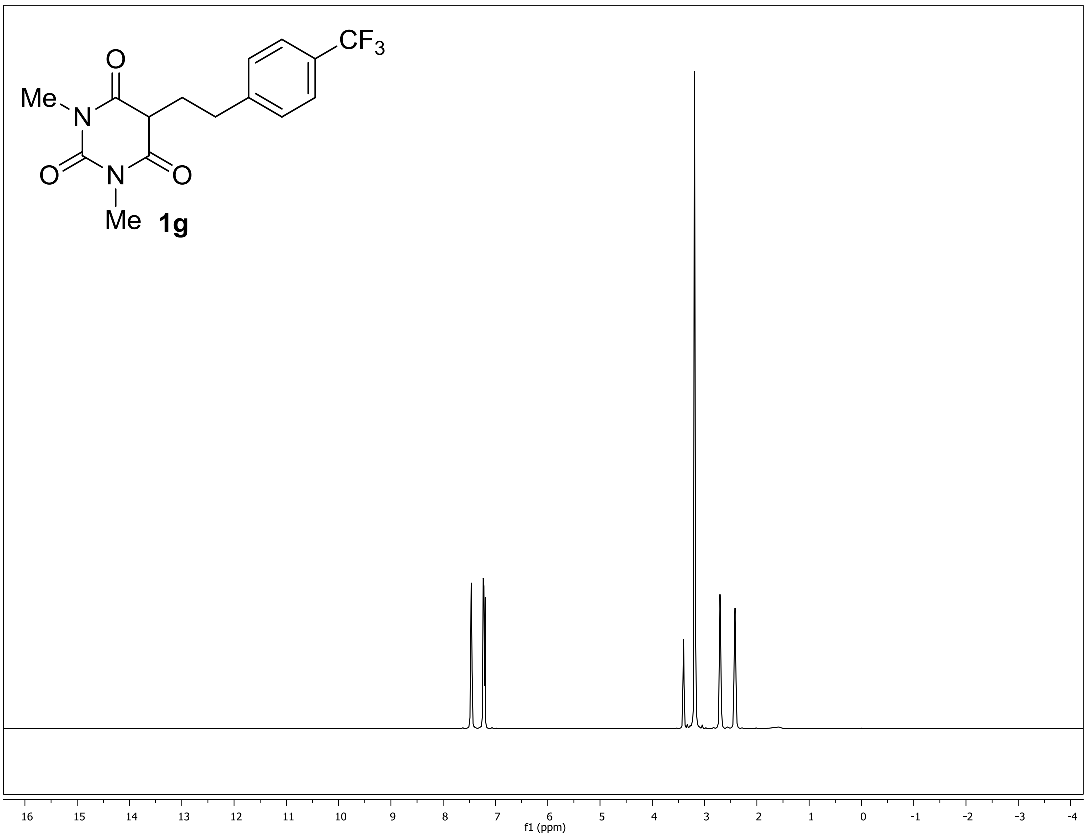
CF3
O
Me
N
O
N
O
Me
1g
16
15
14
13
12
11
10
9
8
7
6
5
4
3
2
1
0
-1
-2
-3
-4
f1 (ppm)

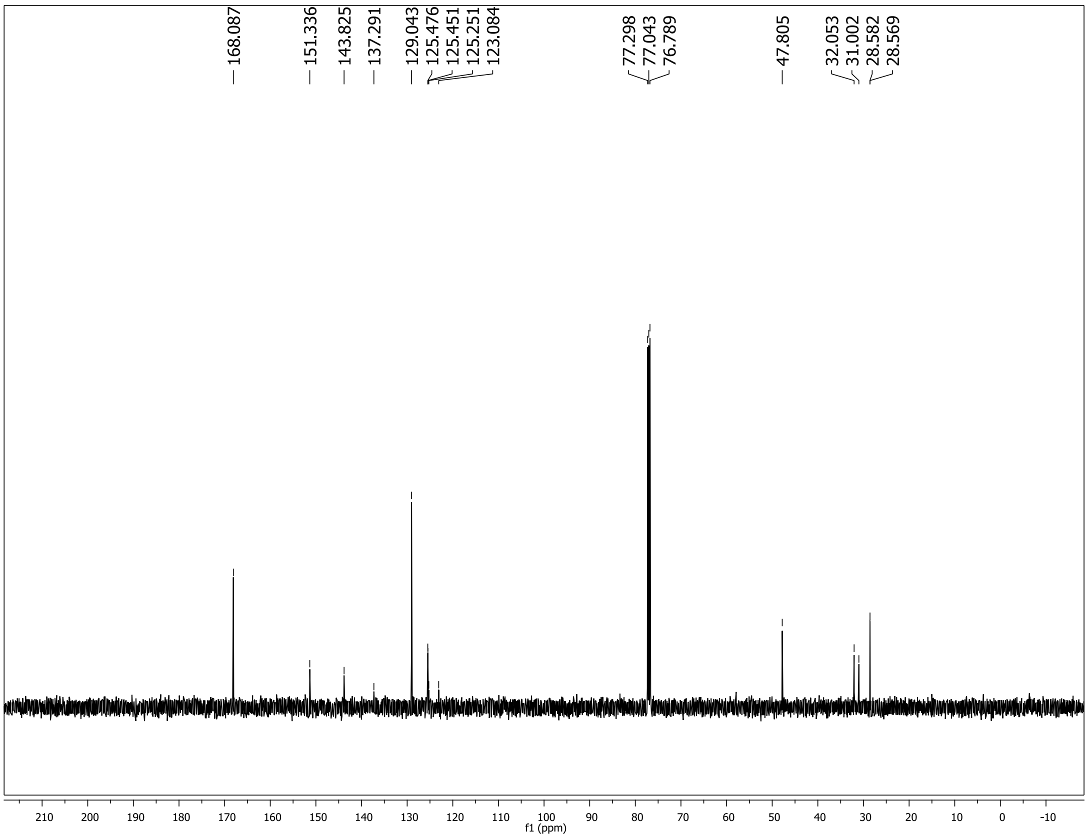
168.087
151.336
143.825
137.291
129.043
125.476
125.451
125.251
123.084
77.298
77.043
76.789
47.805
32.053
31.002
28.582
28.569
210
200
190
180
170
160
150
140
130
120
110
100
90
80
70
60
50
40
30
20
10
0
-10
f1 (ppm)

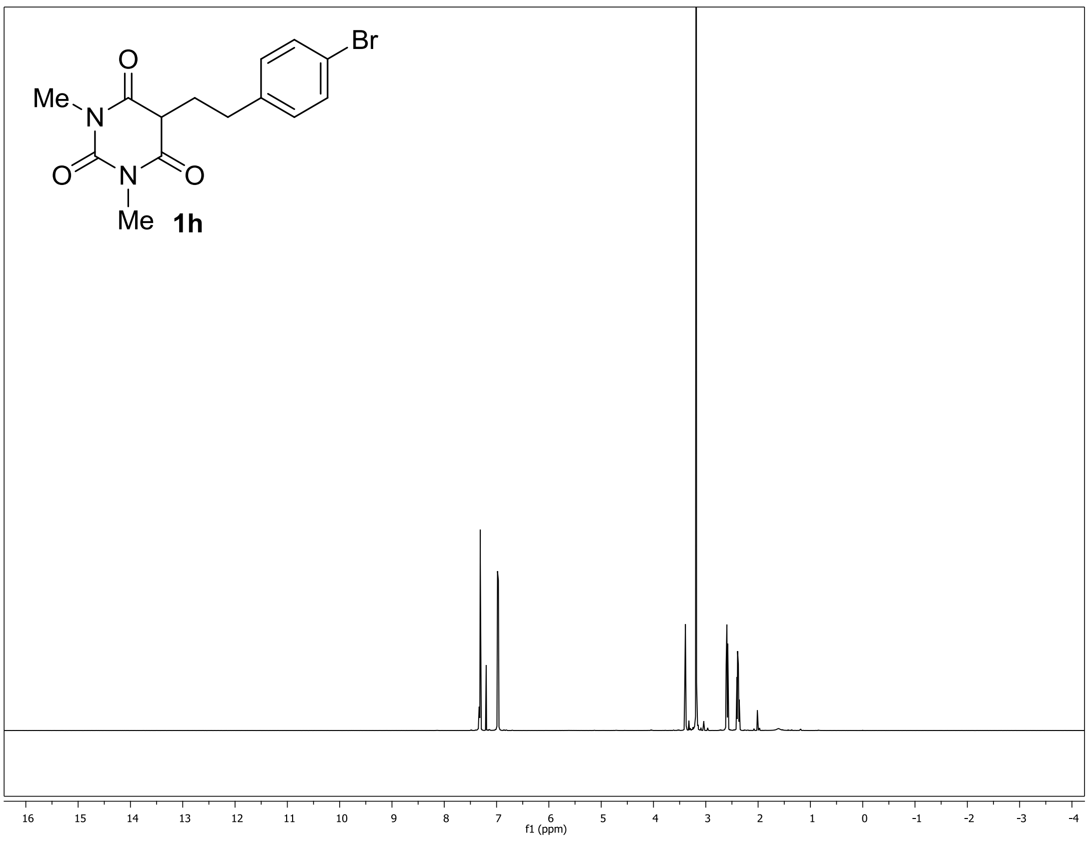
Br
O
Me
N
O
N
O
Me
1h
16 15 14 13 12 11 10 9 8 7 6 5 4 3 2 1 0 -1 -2 -3 -4
f1 (ppm)

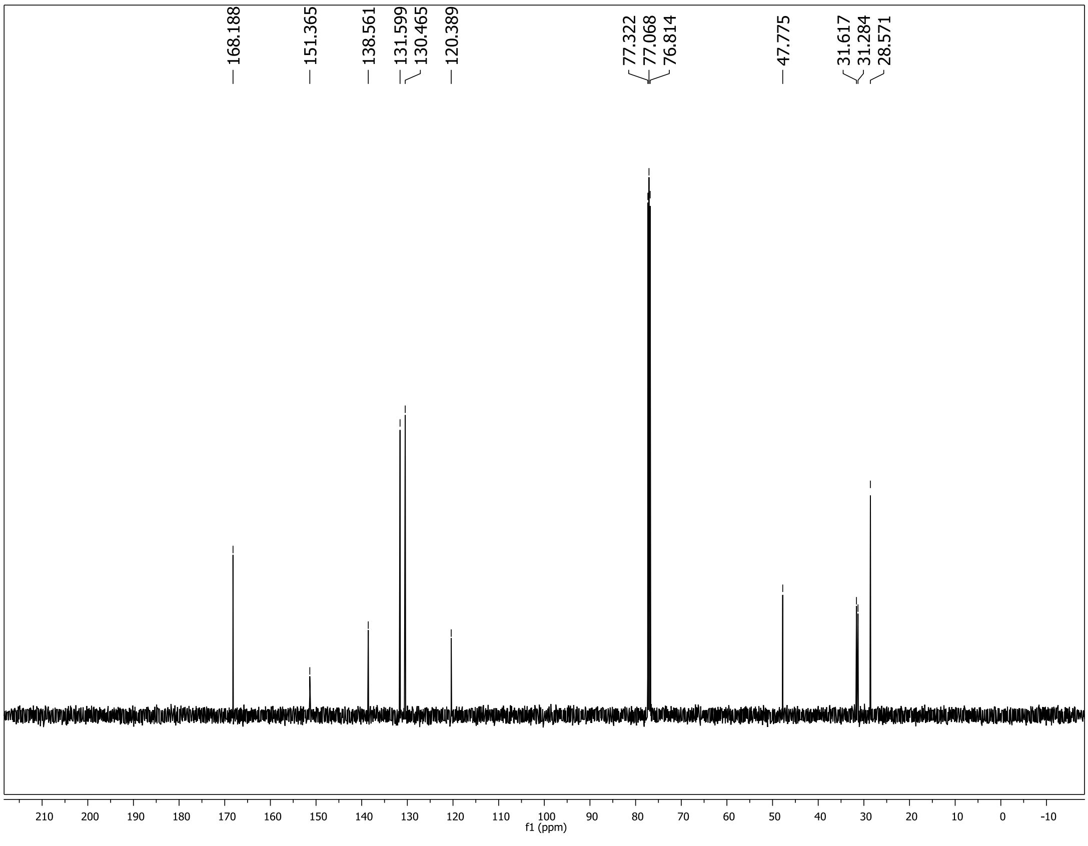
168.188
151.365
138.561
131.599
130.465
120.389
77.322
77.068
76.814
47.775
31.617
31.284
28.571
210 200 190 180 170 160 150 140 130 120 110 100 90 80 70 60 50 40 30 20 10 0 -10
f1 (ppm)

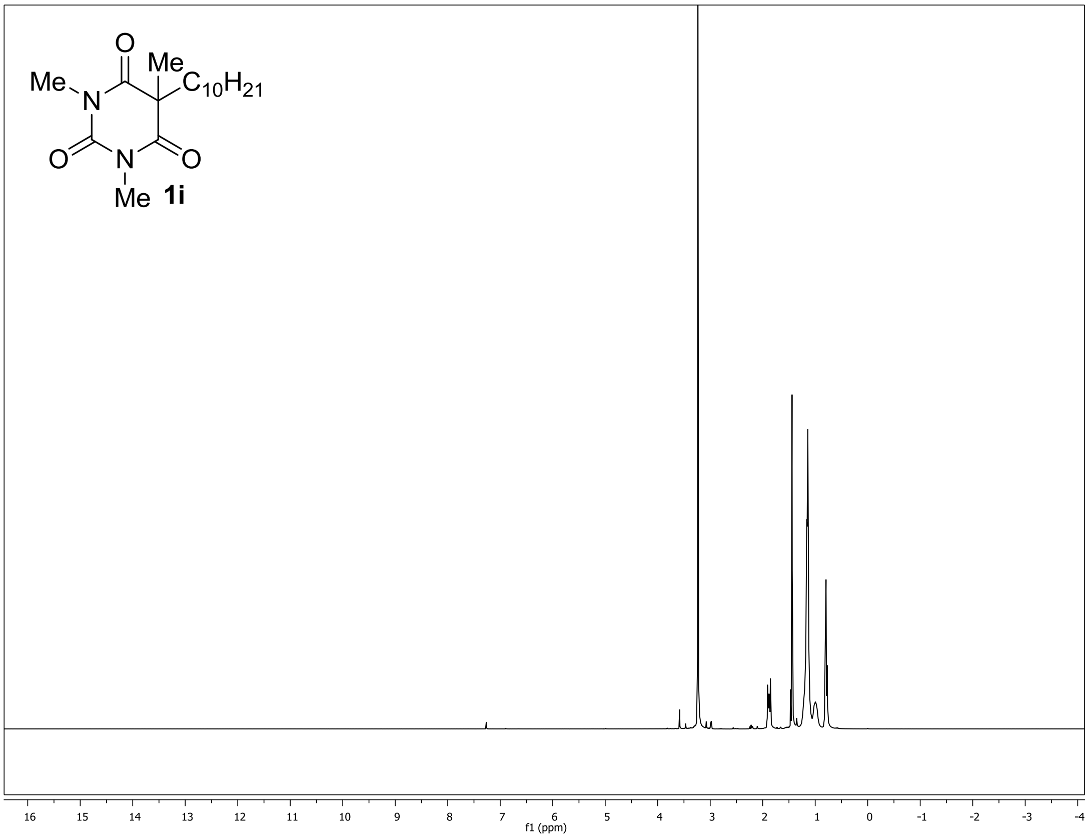
1i
f1 (ppm)

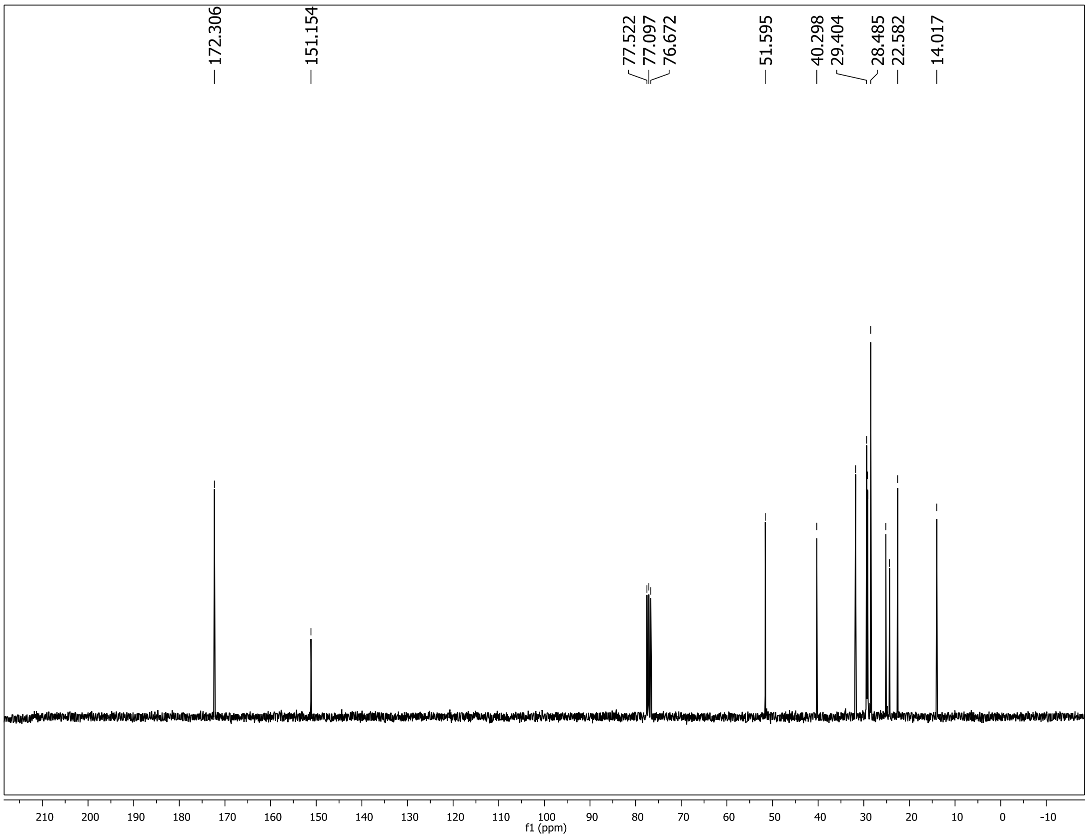
172.306
151.154
77.522
77.097
76.672
51.595
40.298
29.404
28.485
22.582
14.017
f1 (ppm)

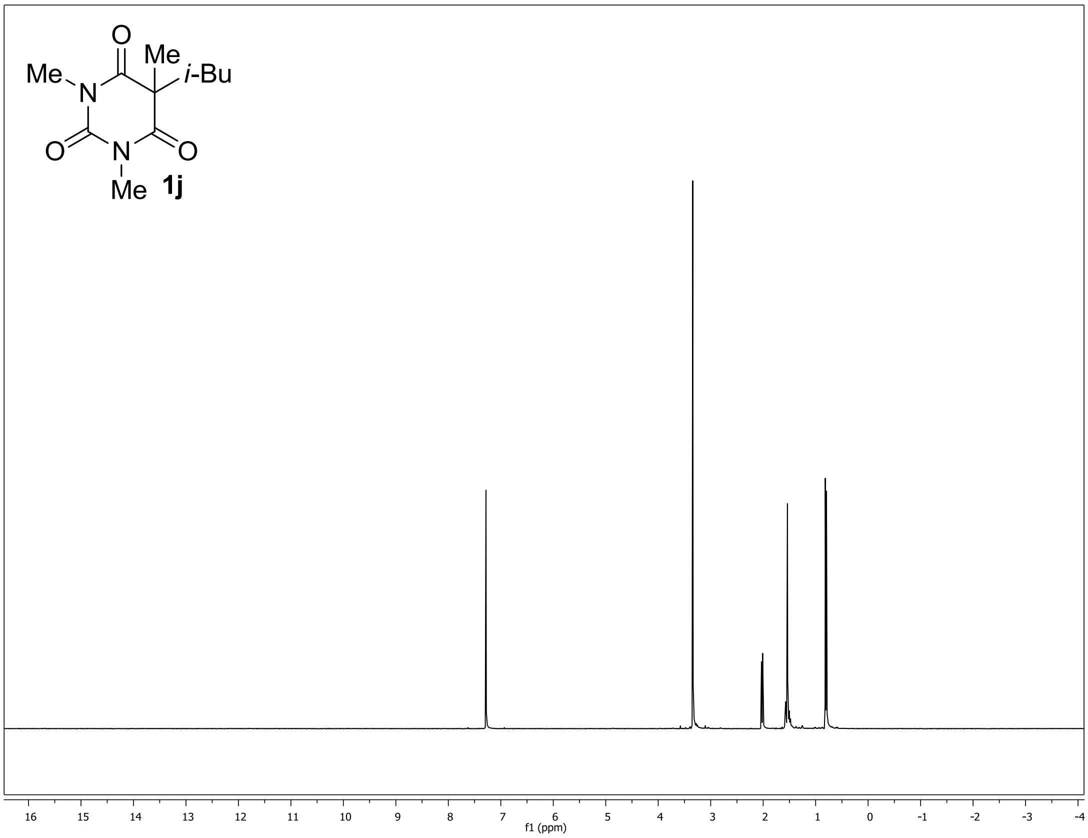
Me
i-Bu
1j
16 15 14 13 12 11 10 9 8 7 6 5 4 3 2 1 0 -1 -2 -3 -4
f1 (ppm)

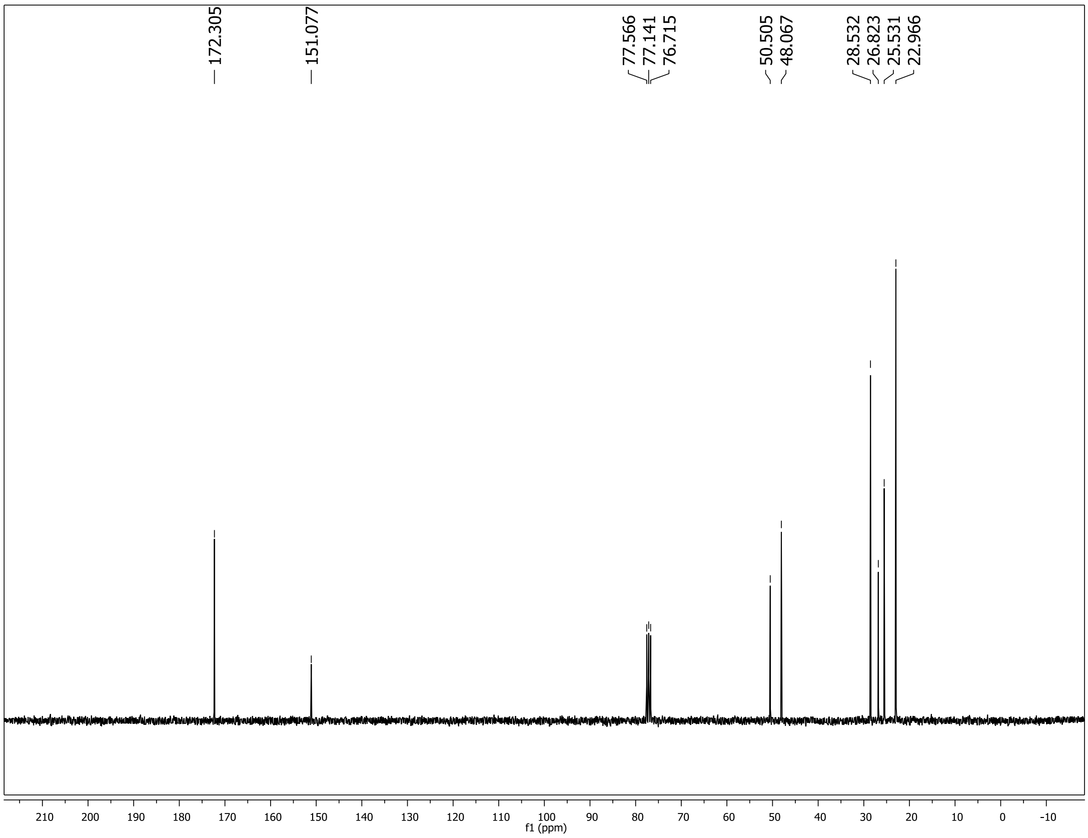
172.305
151.077
77.566
77.141
76.715
50.505
48.067
28.532
26.823
25.531
22.966
210 200 190 180 170 160 150 140 130 120 110 100 90 80 70 60 50 40 30 20 10 0 -10
f1 (ppm)

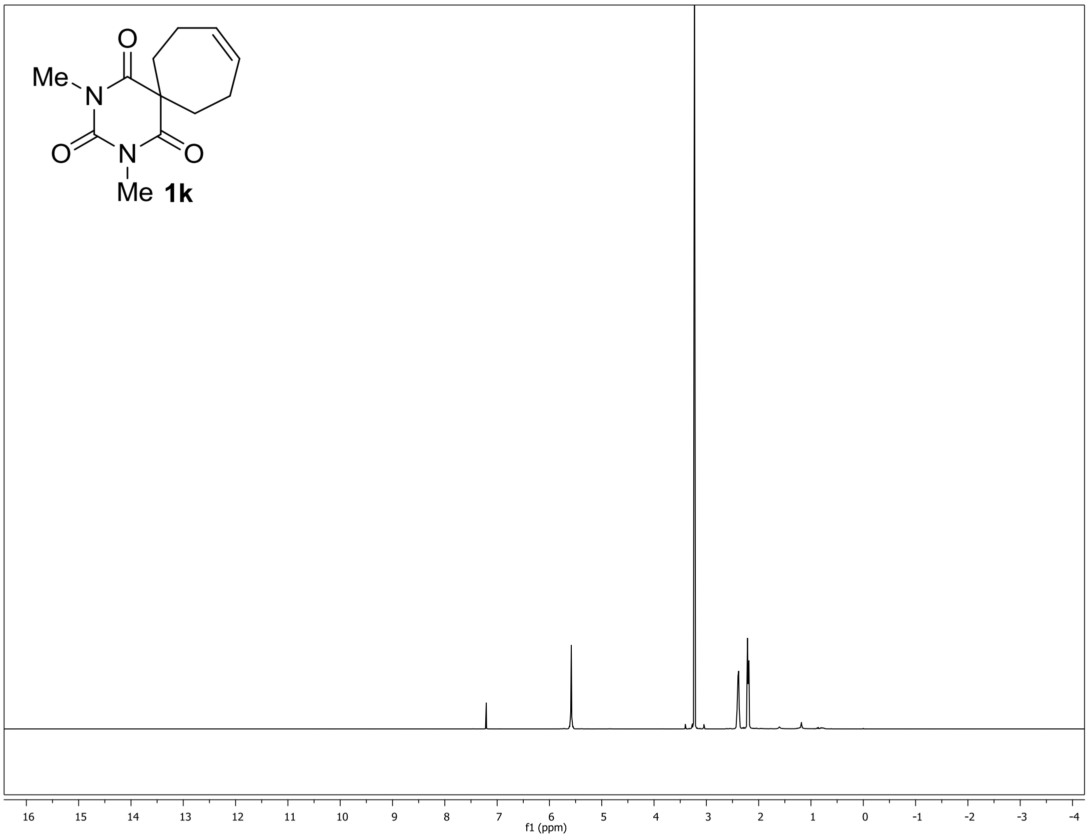
Me
N
O
N
O
O
Me 1k
16 15 14 13 12 11 10 9 8 7 6 5 4 3 2 1 0 -1 -2 -3 -4
f1 (ppm)

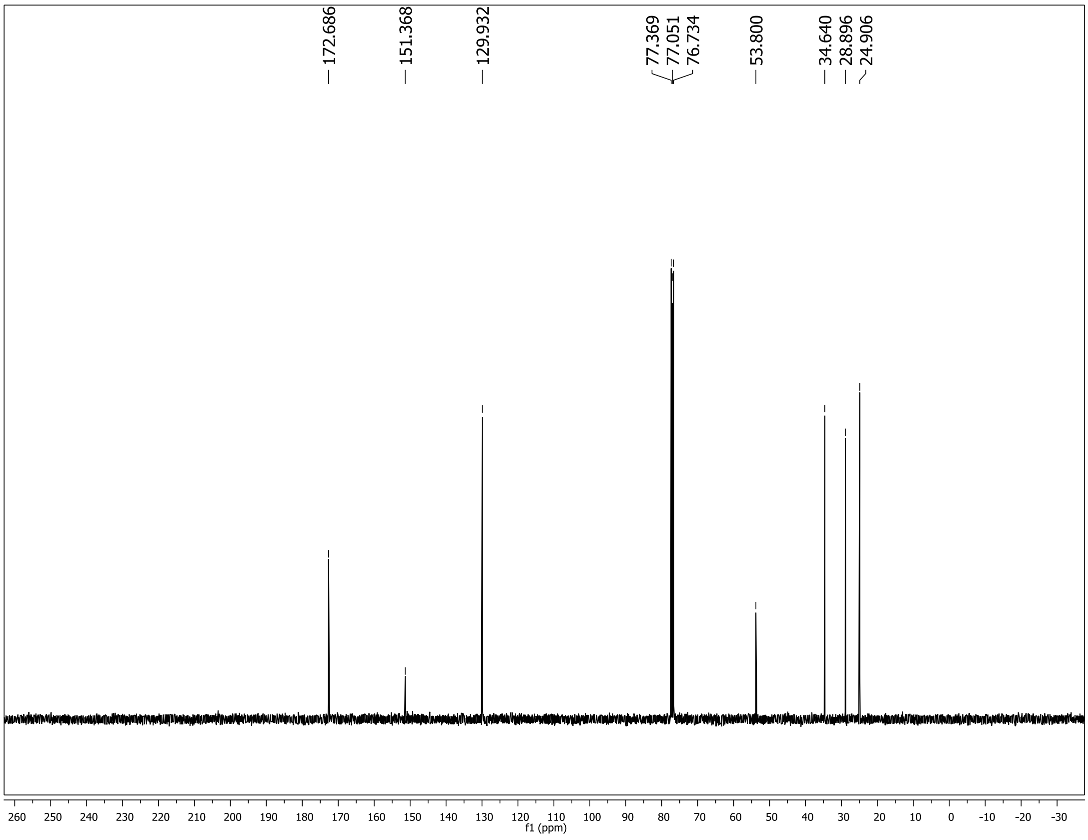
172.686
151.368
129.932
77.369
77.051
76.734
53.800
34.640
28.896
24.906
260 250 240 230 220 210 200 190 180 170 160 150 140 130 120 110 100 90 80 70 60 50 40 30 20 10 0 -10 -20 -30
f1 (ppm)

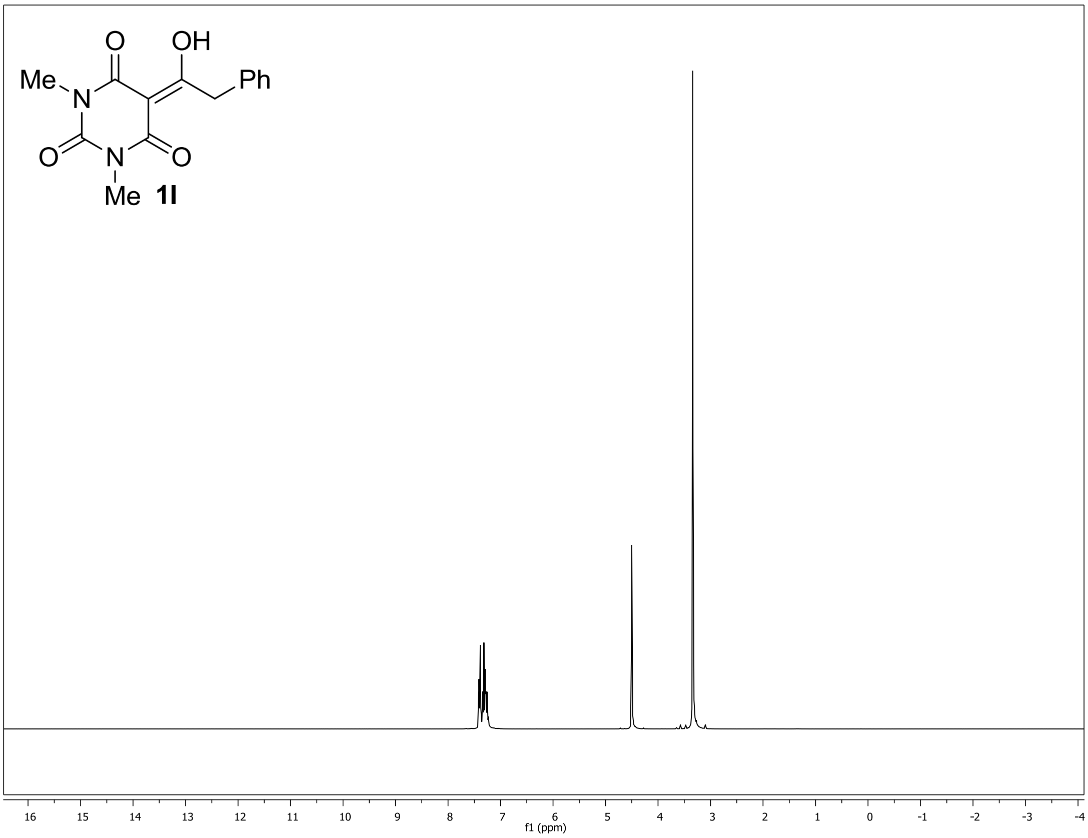
O
OH
Me
N
Ph
O
N
O
Me 1l
16 15 14 13 12 11 10 9 8 7 6 5 4 3 2 1 0 -1 -2 -3 -4
f1 (ppm)

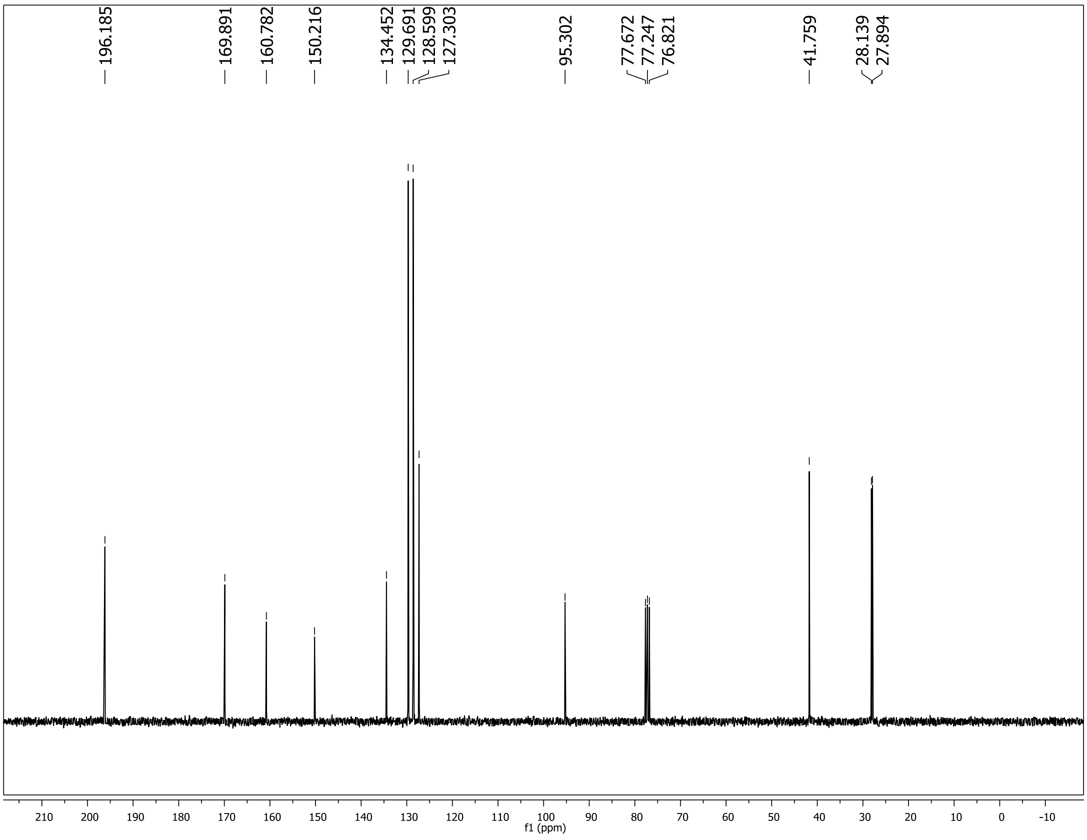
196.185
169.891
160.782
150.216
134.452
129.691
128.599
127.303
95.302
77.672
77.247
76.821
41.759
28.139
27.894
210 200 190 180 170 160 150 140 130 120 110 100 90 80 70 60 50 40 30 20 10 0 -10
f1 (ppm)

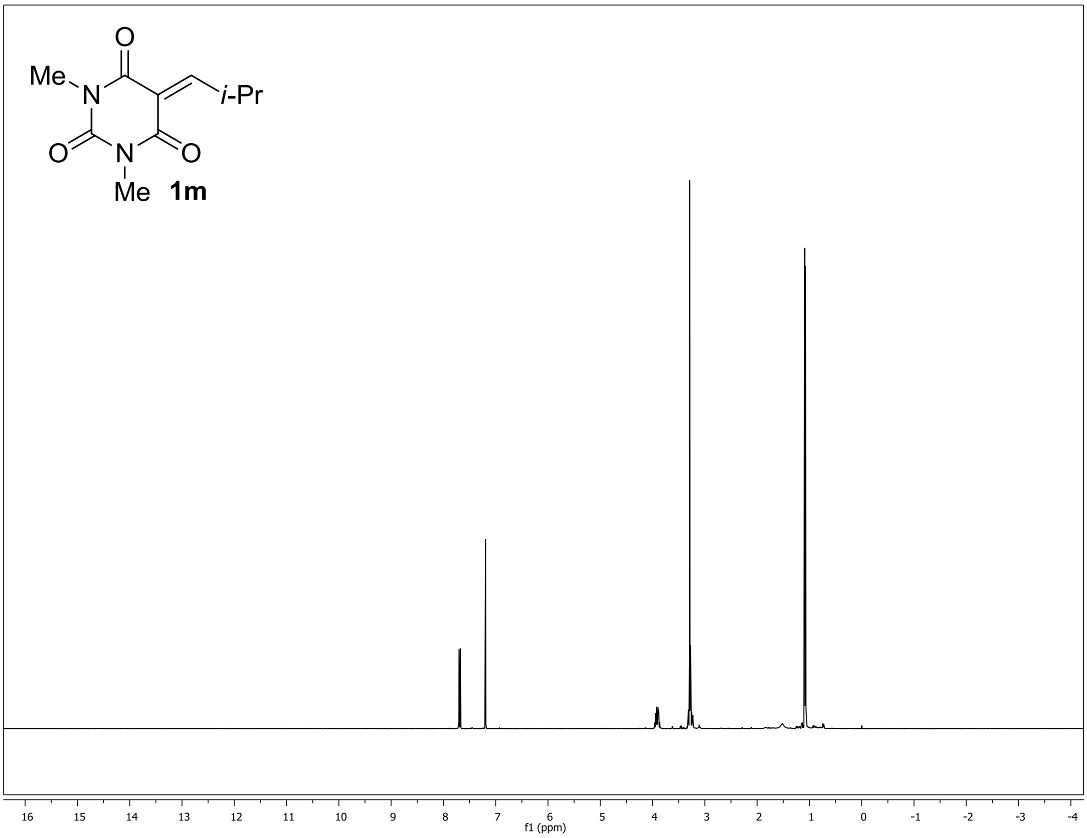
Me
N
O
i-Pr
N
O
O
Me
1m
16 15 14 13 12 11 10 9 8 7 6 5 4 3 2 1 0 -1 -2 -3 -4
f1 (ppm)

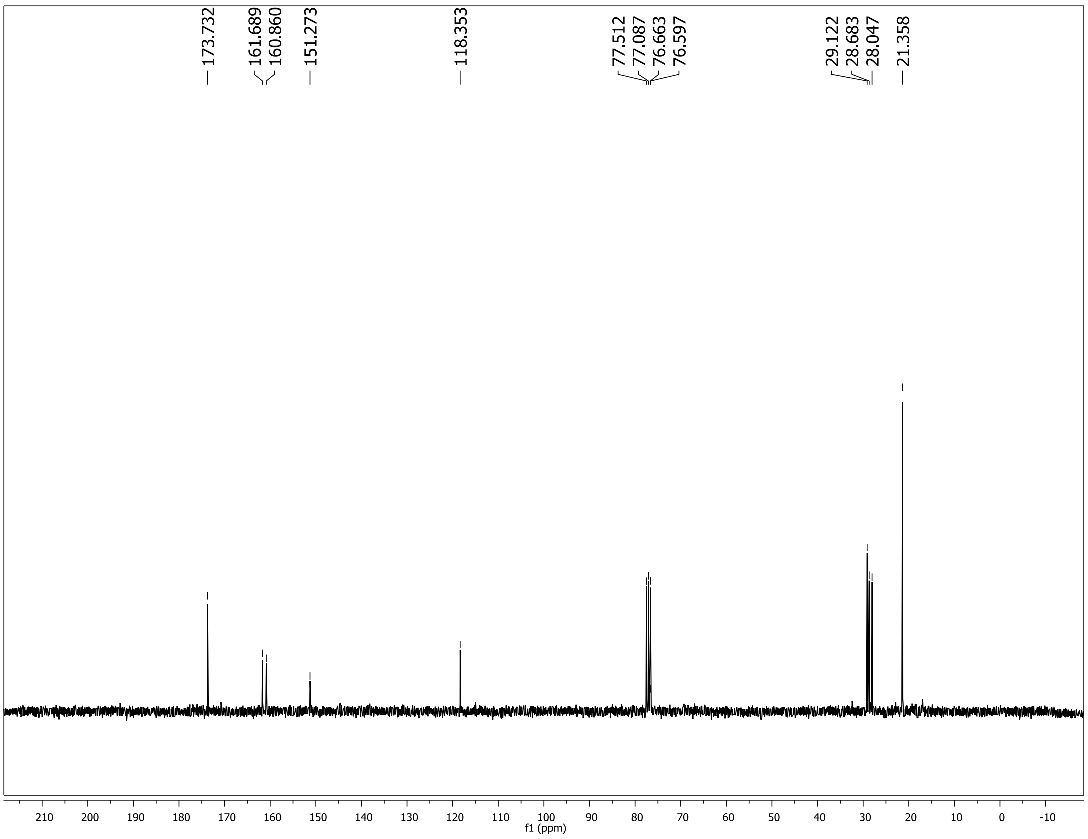
173.732
161.689
160.860
151.273
118.353
77.512
77.087
76.663
76.597
29.122
28.683
28.047
21.358
210 200 190 180 170 160 150 140 130 120 110 100 90 80 70 60 50 40 30 20 10 0 -10
f1 (ppm)

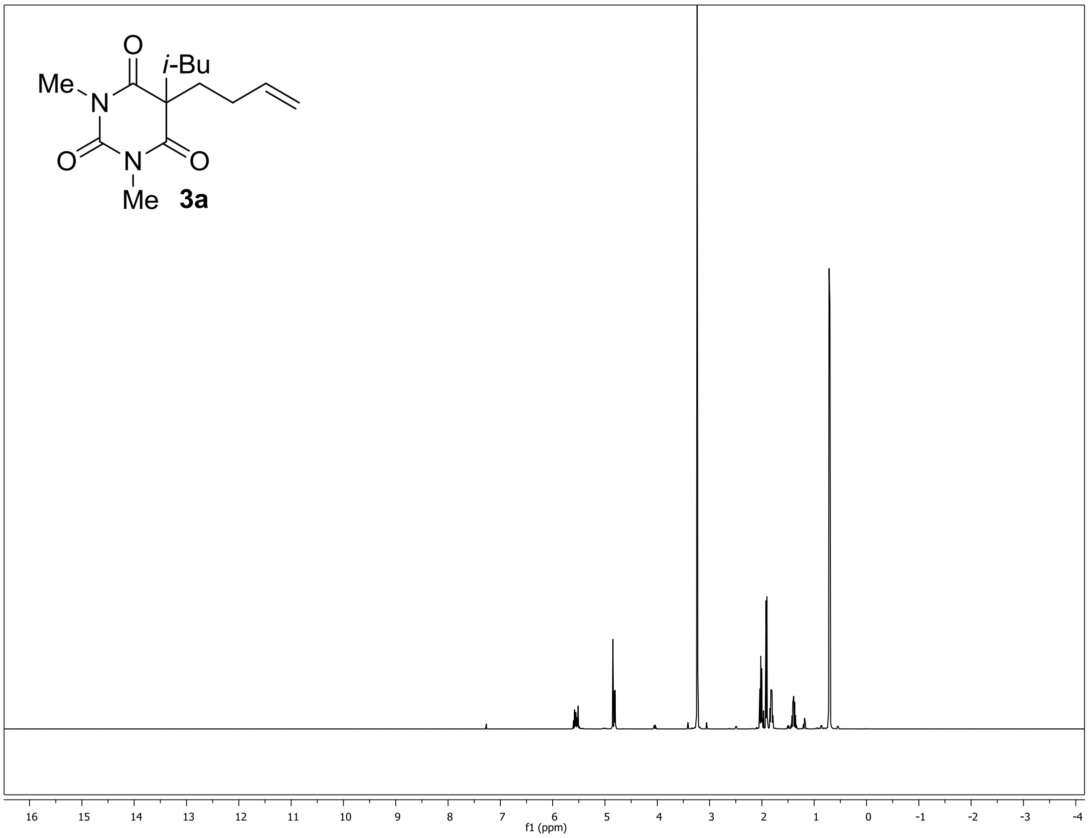
O
i-Bu
Me
N
O
N
O
Me
3a
16 15 14 13 12 11 10 9 8 7 6 5 4 3 2 1 0 -1 -2 -3 -4
f1 (ppm)

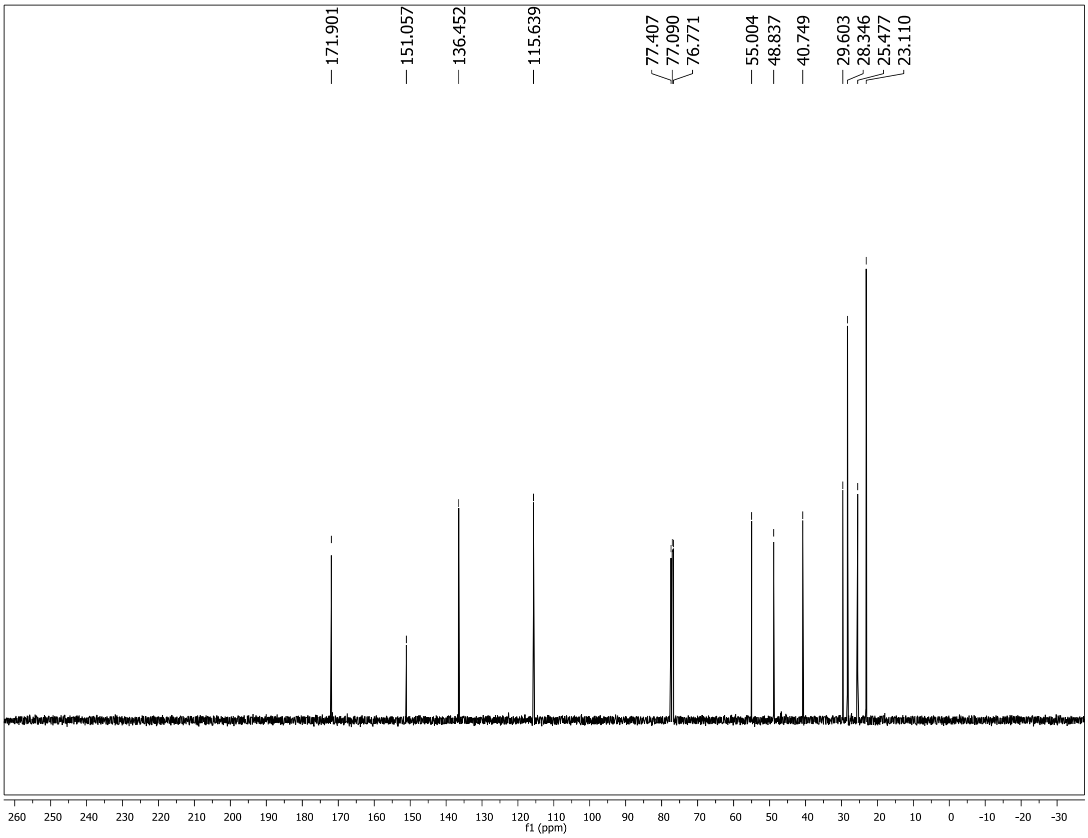
171.901
151.057
136.452
115.639
77.407
77.090
76.771
55.004
48.837
40.749
29.603
28.346
25.477
23.110
260 250 240 230 220 210 200 190 180 170 160 150 140 130 120 110 100 90 80 70 60 50 40 30 20 10 0 -10 -20 -30
f1 (ppm)

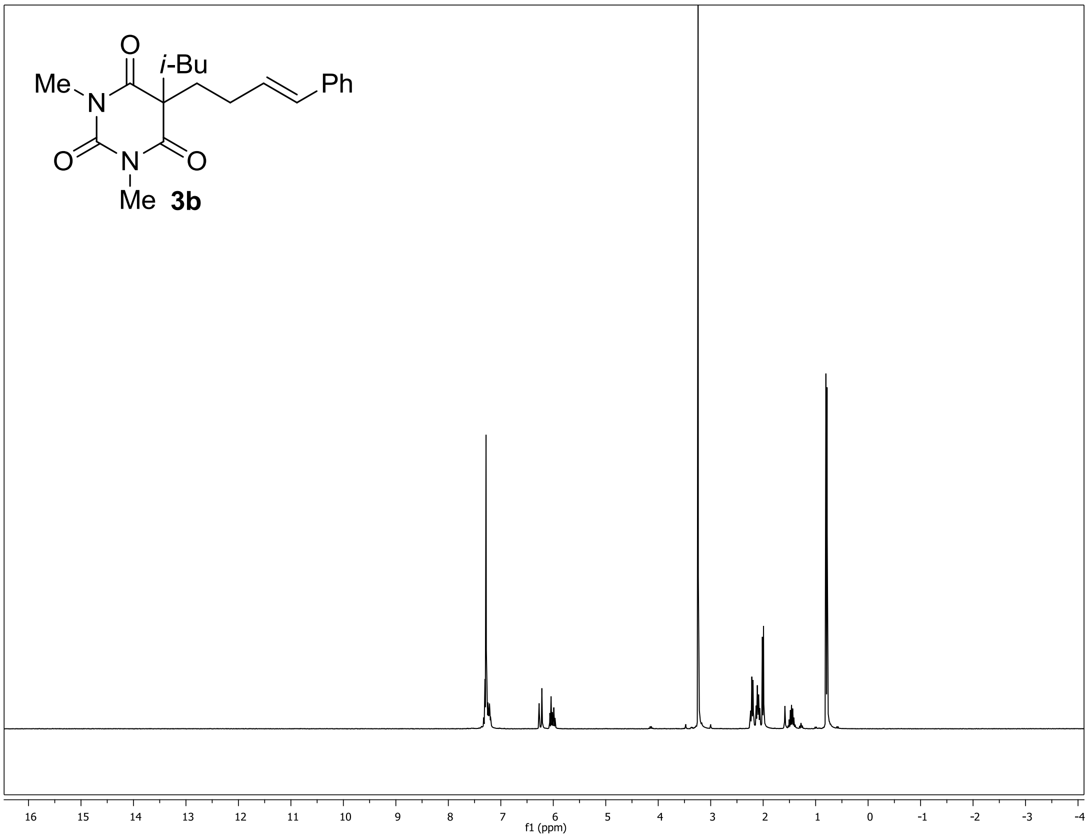

O
i-Bu
Me
N
Ph
O
N
O
Me 3b
16 15 14 13 12 11 10 9 8 7 6 5 4 3 2 1 0 -1 -2 -3 -4
f1 (ppm)

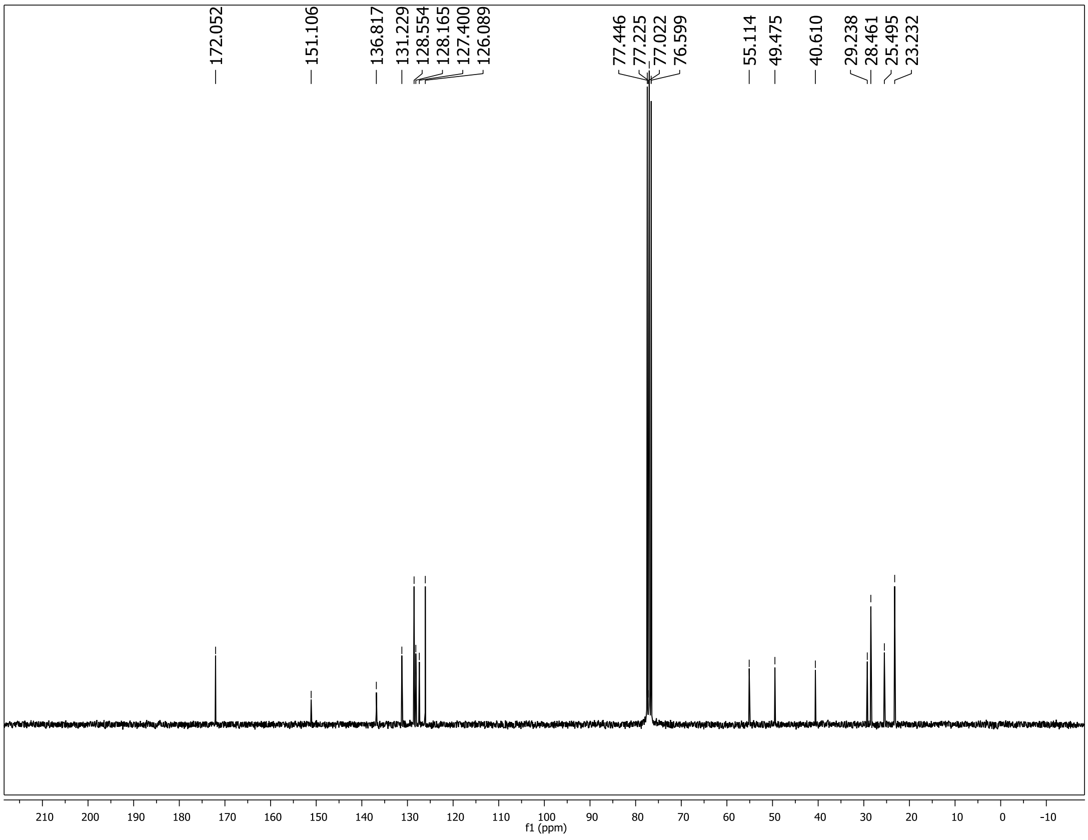

172.052
151.106
136.817
131.229
128.554
128.165
127.400
126.089
77.446
77.225
77.022
76.599
55.114
49.475
40.610
29.238
28.461
25.495
23.232
210 200 190 180 170 160 150 140 130 120 110 100 90 80 70 60 50 40 30 20 10 0 -10
f1 (ppm)

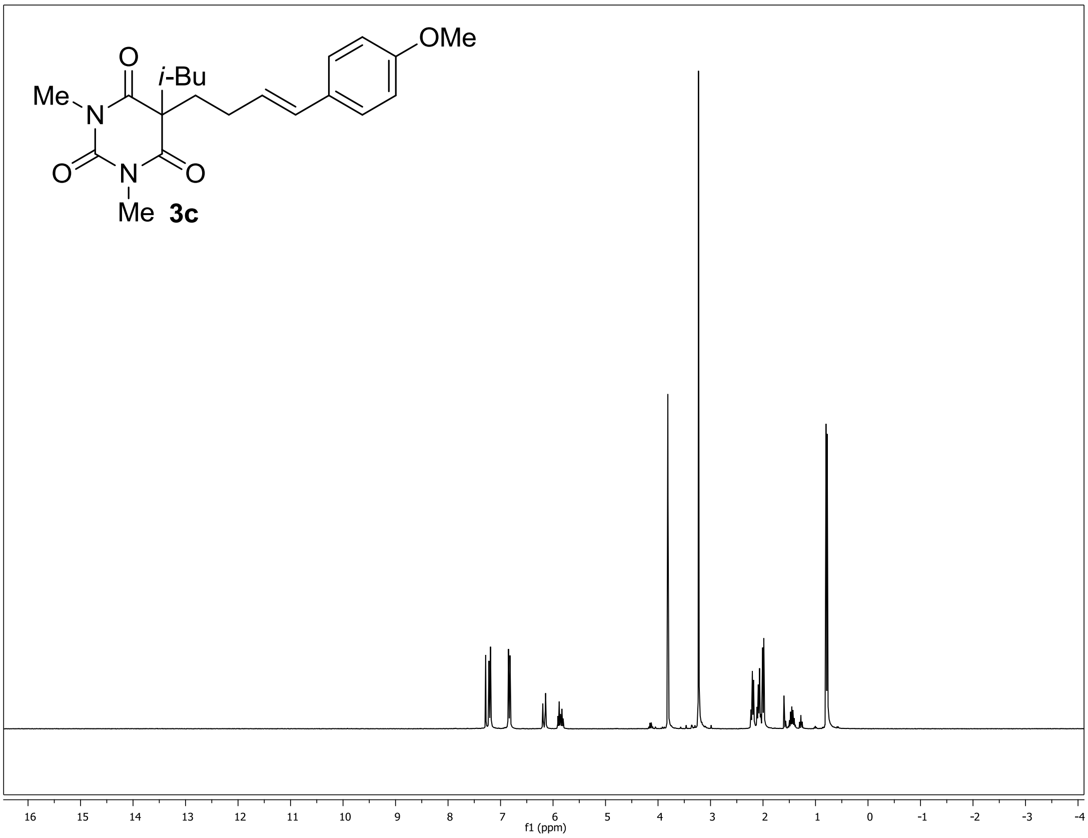
OMe
i-Bu
Me
N
O
N
O
O
Me
3c
f1 (ppm)

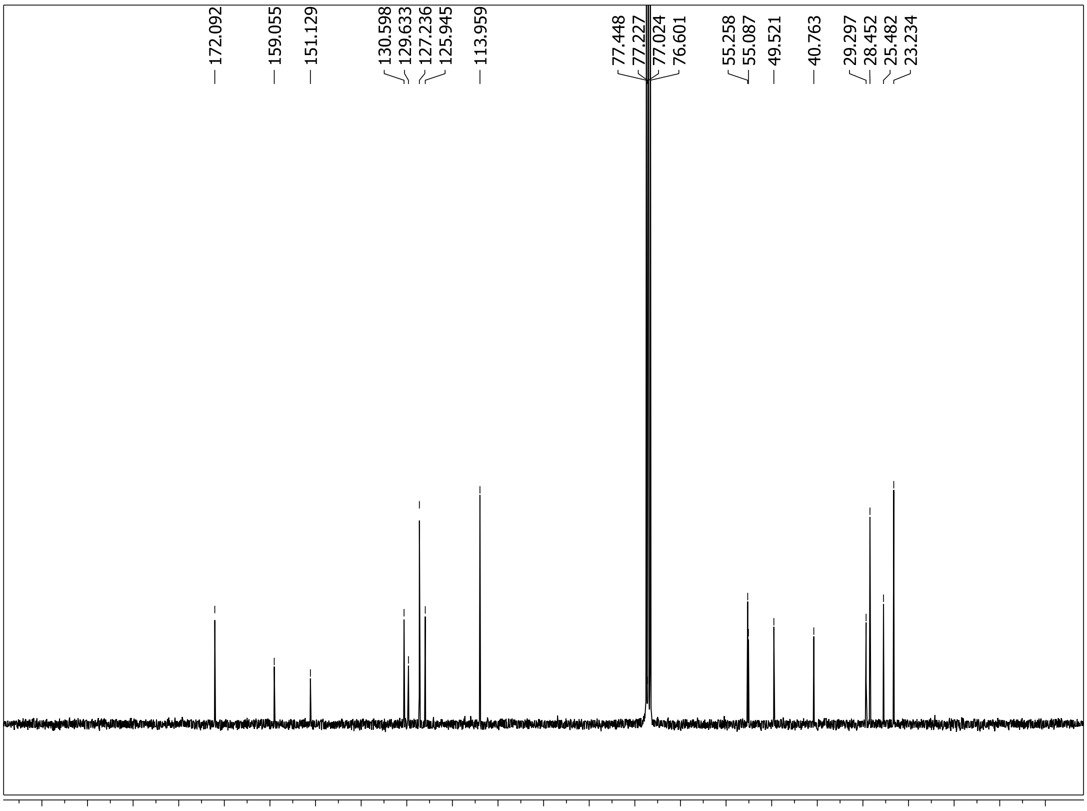
172.092
159.055
151.129
130.598
129.633
127.236
125.945
113.959
77.448
77.227
77.024
76.601
55.258
55.087
49.521
40.763
29.297
28.452
25.482
23.234
f1 (ppm)

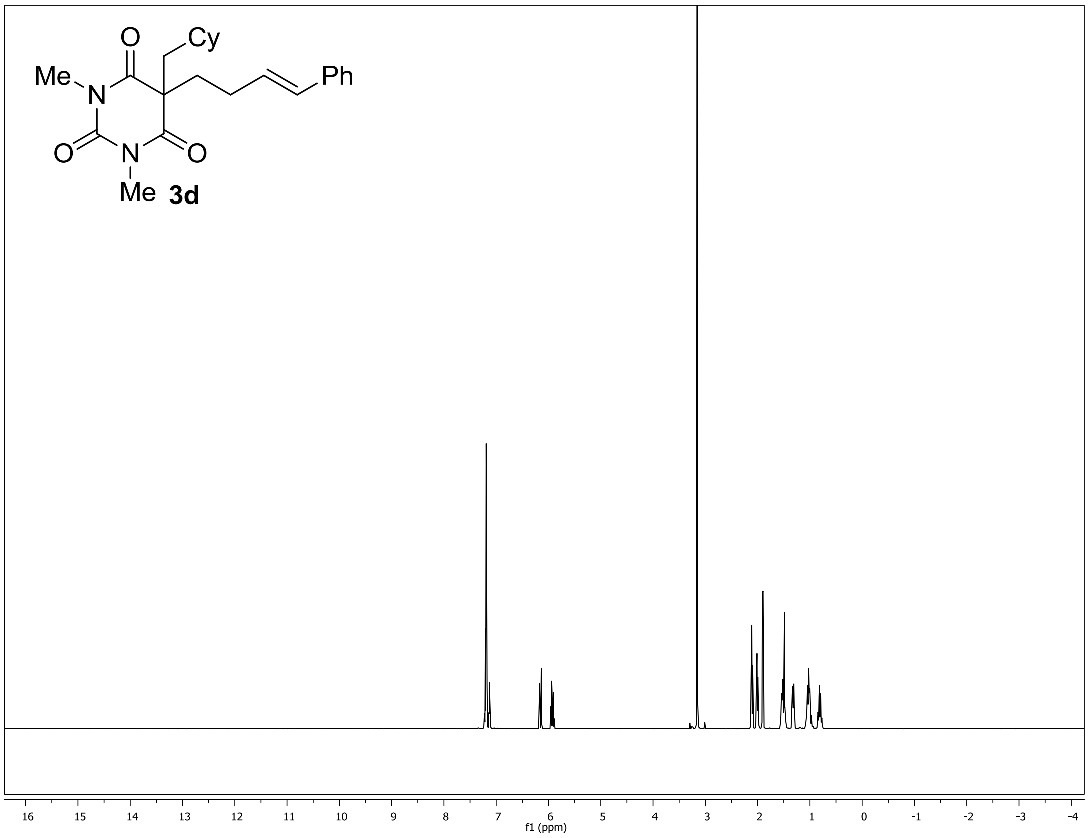
Cy
Me
N
O
Ph
Me
3d
f1 (ppm)

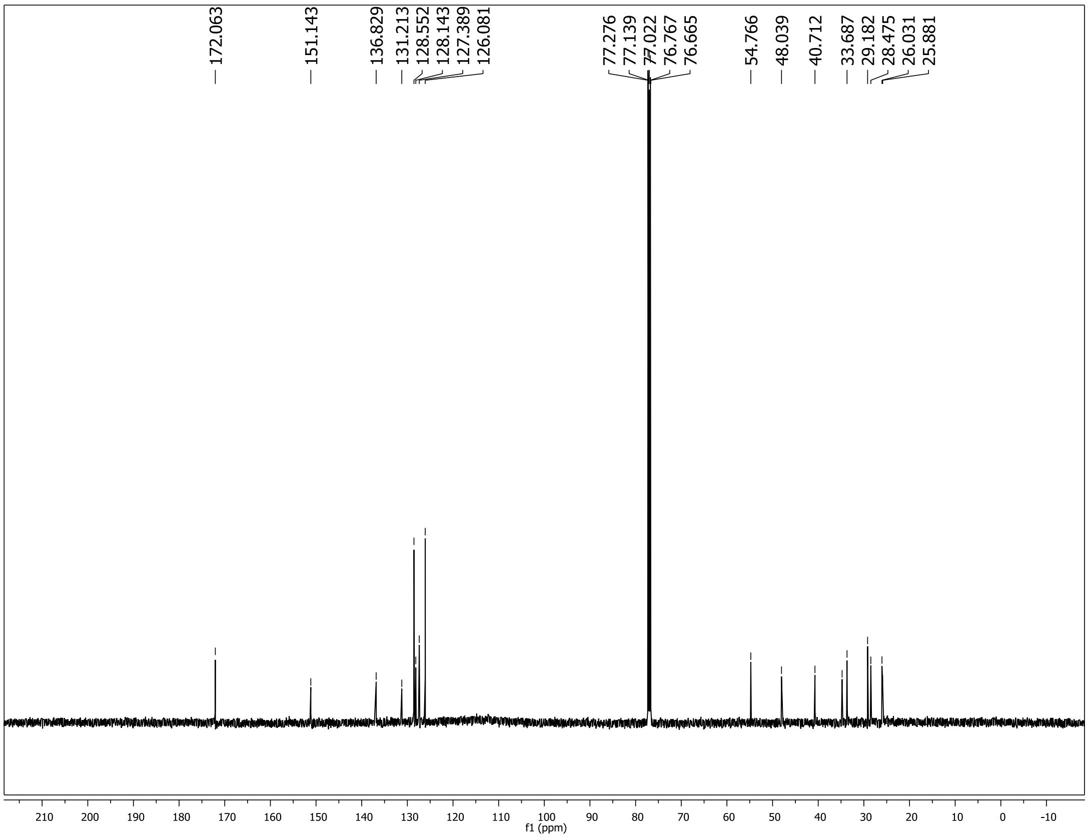
172.063
151.143
136.829
131.213
128.552
128.143
127.389
126.081
77.276
77.139
77.022
76.767
76.665
54.766
48.039
40.712
33.687
29.182
28.475
26.031
25.881
f1 (ppm)

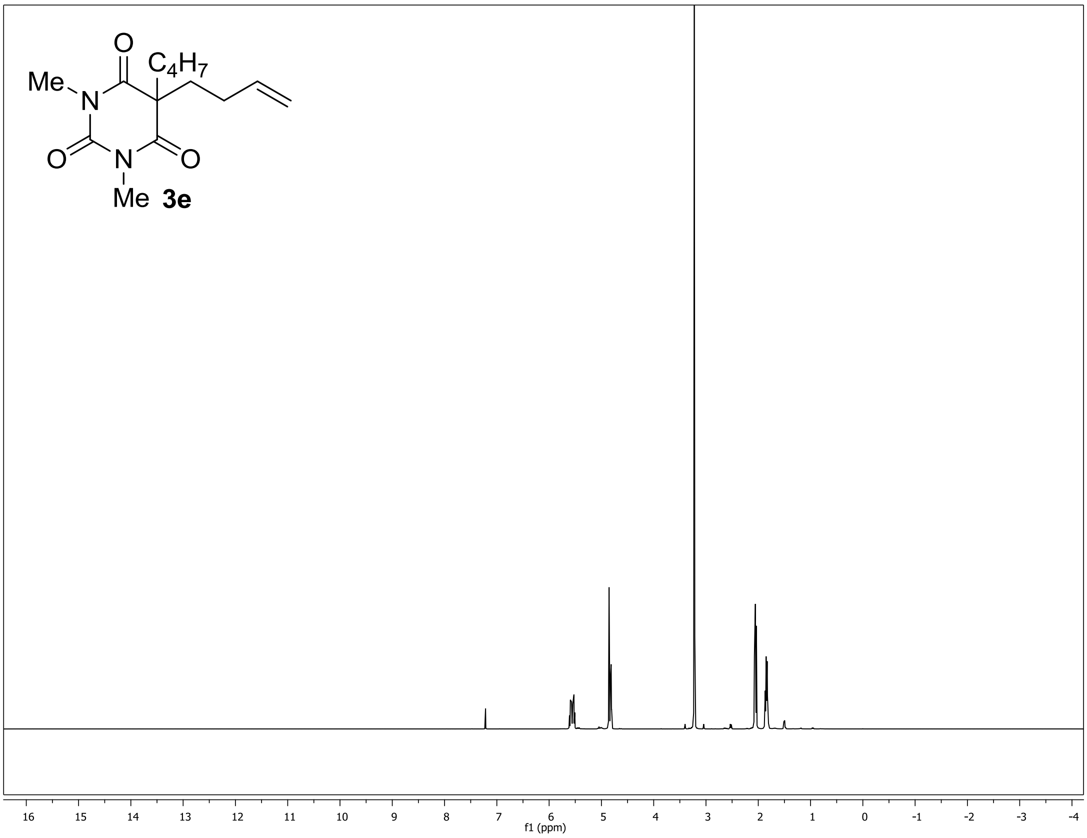
Me
N
O
C4H7
O
N
O
Me
3e
16 15 14 13 12 11 10 9 8 7 6 5 4 3 2 1 0 -1 -2 -3 -4
f1 (ppm)

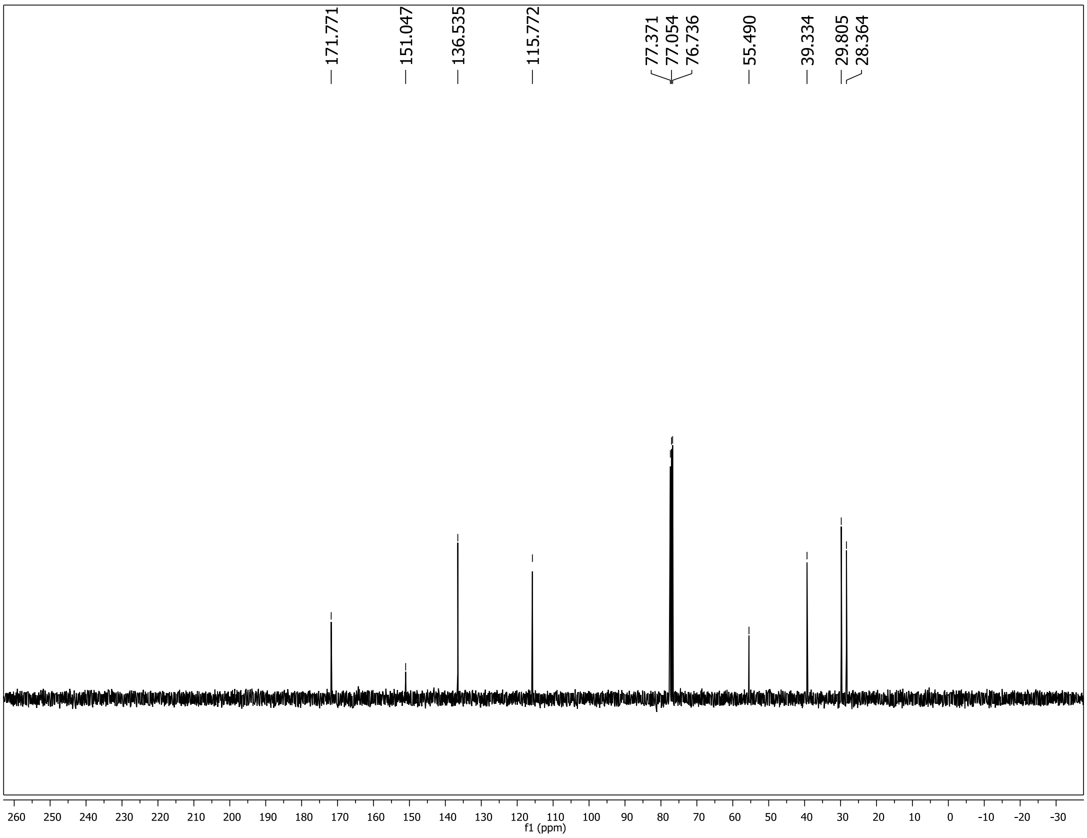
171.771
151.047
136.535
115.772
77.371
77.054
76.736
55.490
39.334
29.805
28.364
260 250 240 230 220 210 200 190 180 170 160 150 140 130 120 110 100 90 80 70 60 50 40 30 20 10 0 -10 -20 -30
f1 (ppm)

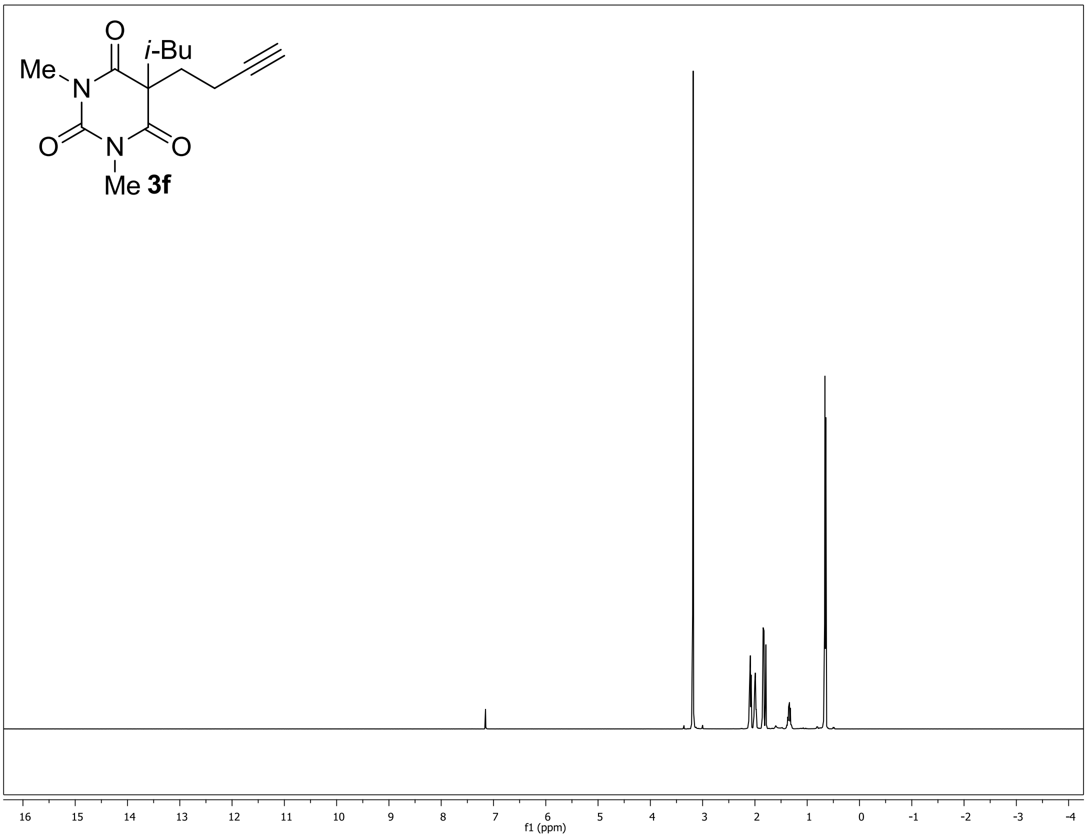

Me
N
O
i-Bu
O
N
O
Me
3f
f1 (ppm)

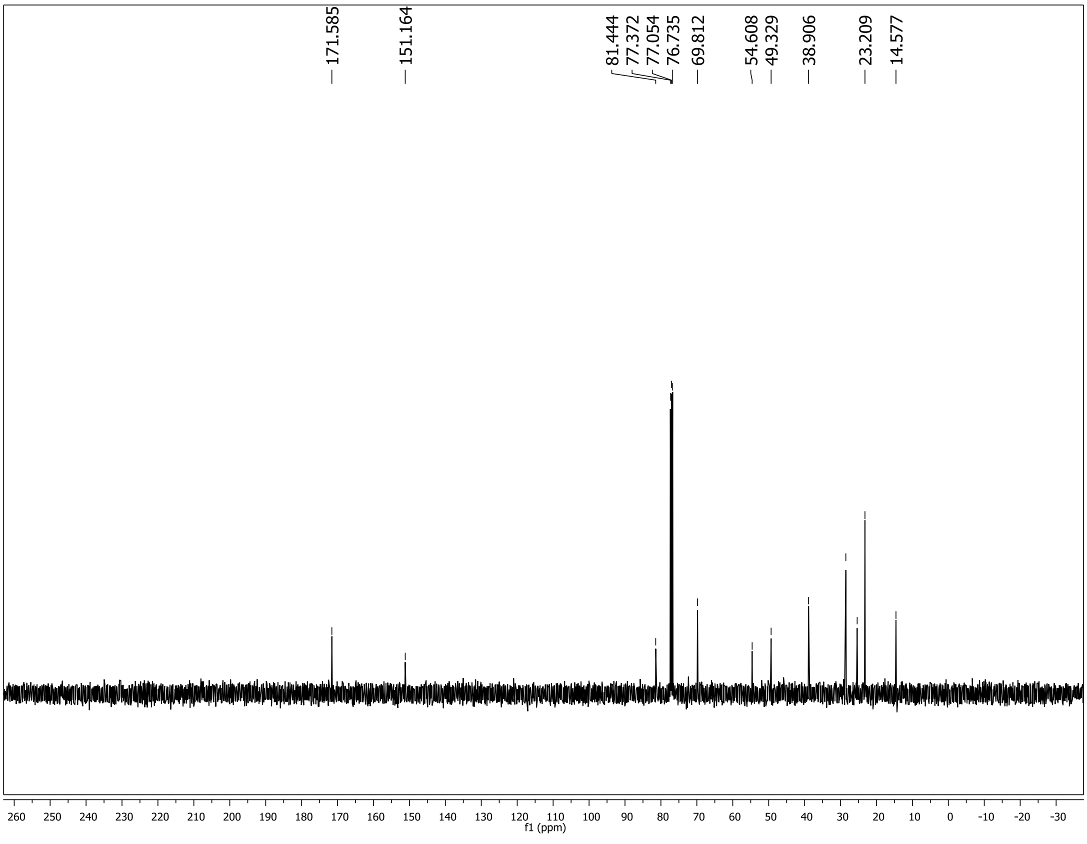

171.585
151.164
81.444
77.372
77.054
76.735
69.812
54.608
49.329
38.906
23.209
14.577
f1 (ppm)

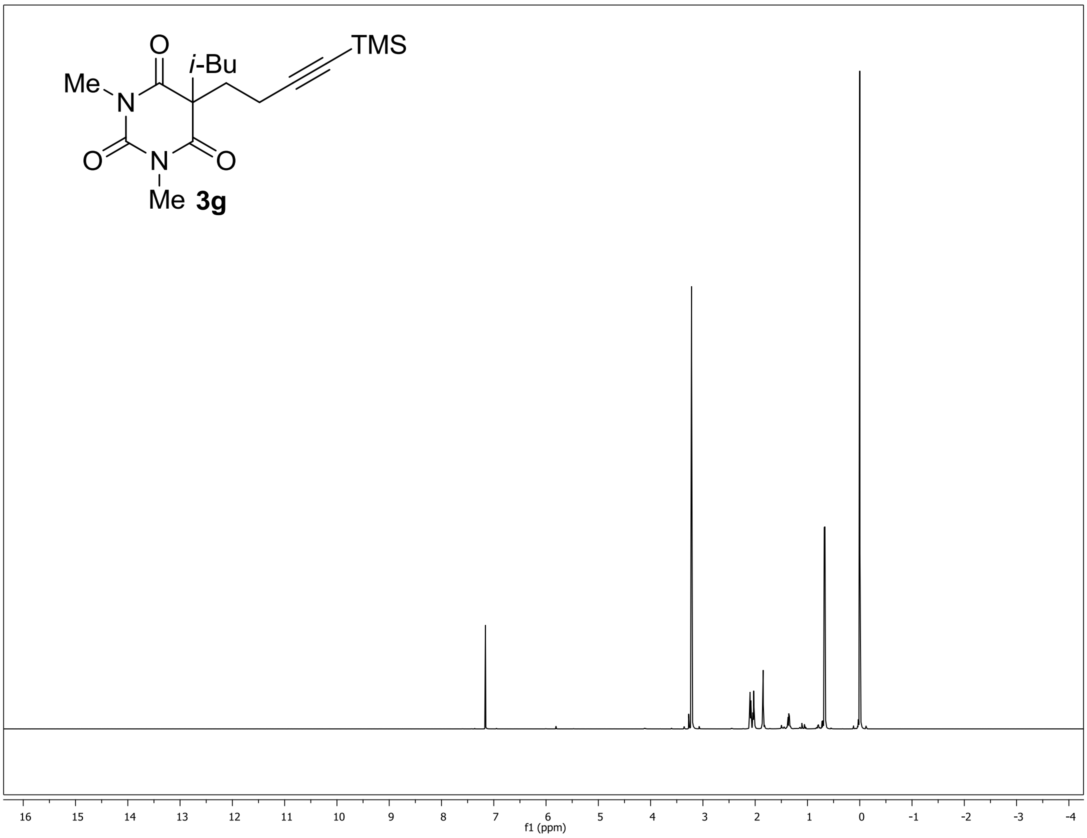
TMS
i-Bu
Me
N
O
Me 3g
16 15 14 13 12 11 10 9 8 7 6 5 4 3 2 1 0 -1 -2 -3 -4
f1 (ppm)

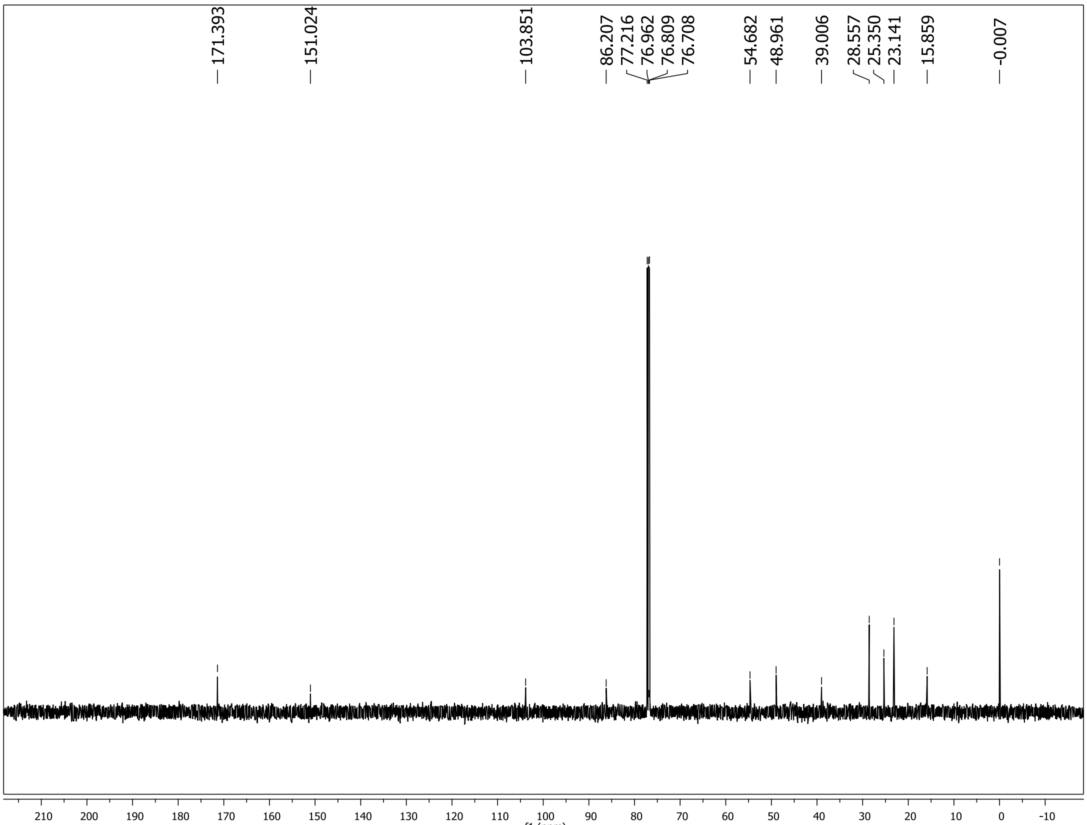
171.393
151.024
103.851
86.207
77.216
76.962
76.809
76.708
54.682
48.961
39.006
28.557
25.350
23.141
15.859
-0.007
210 200 190 180 170 160 150 140 130 120 110 100 90 80 70 60 50 40 30 20 10 0 -10
f1 (ppm)

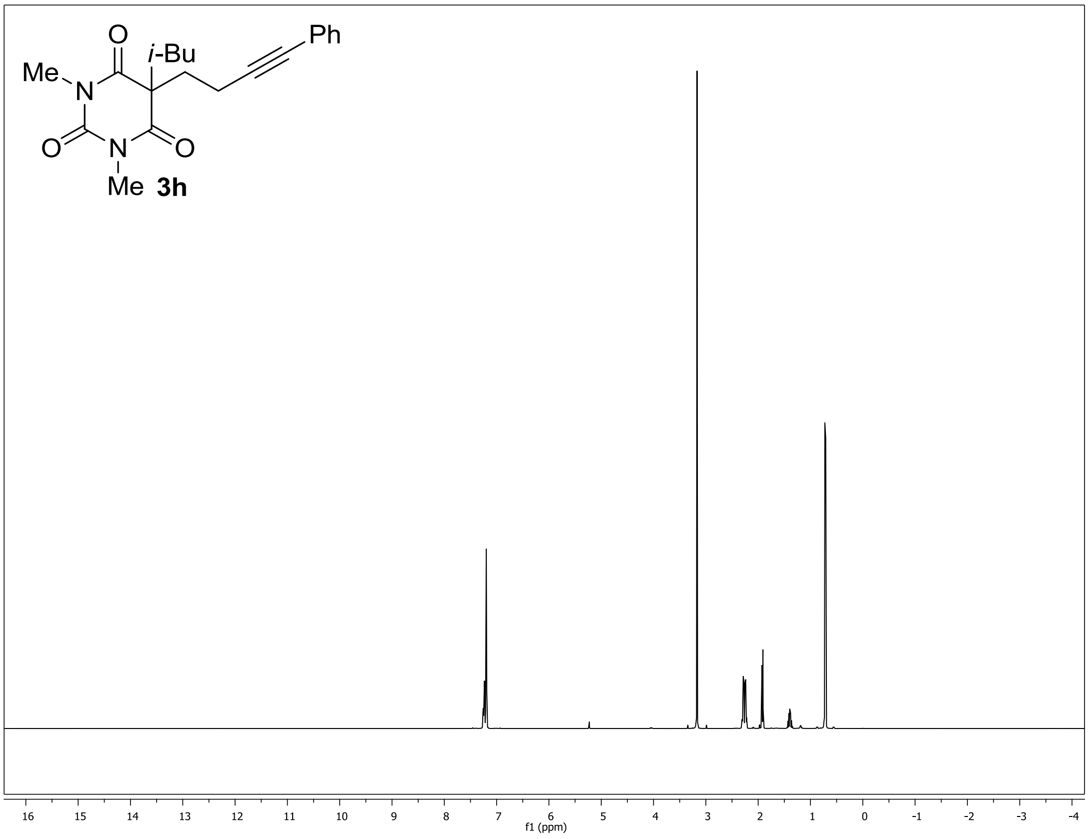
Me
N
O
i-Bu
Ph
Me
3h
f1 (ppm)

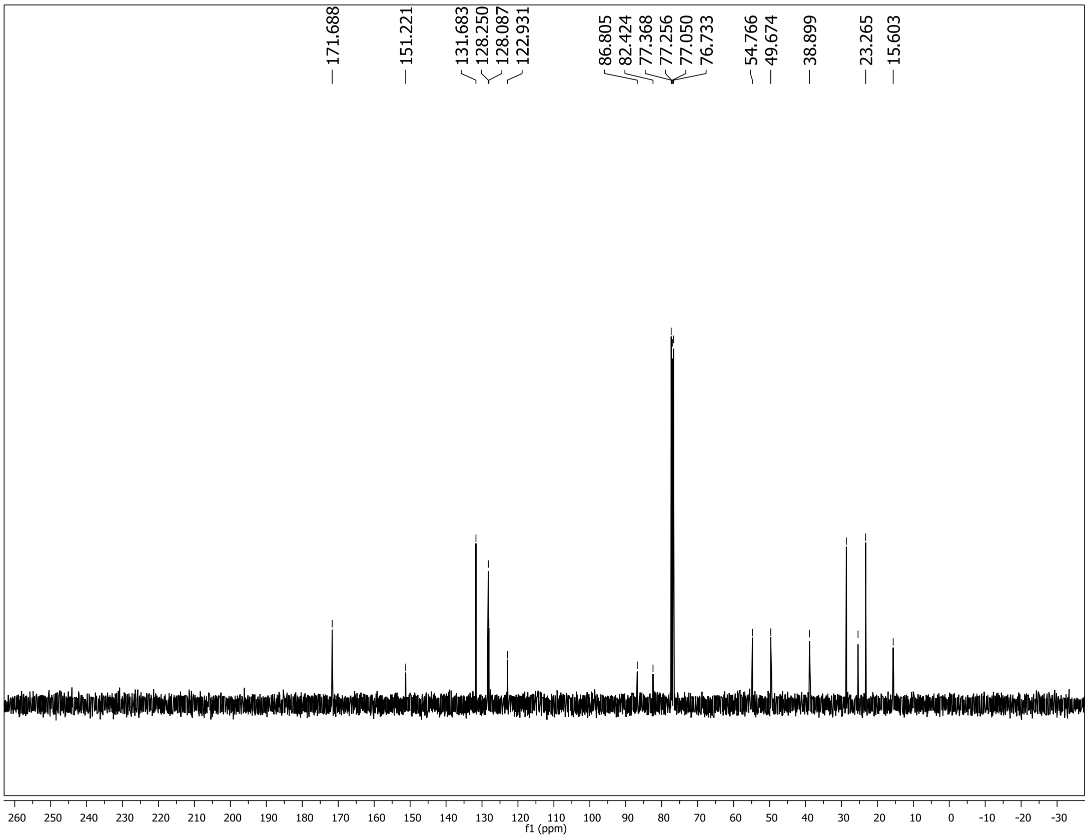
171.688
151.221
131.683
128.250
128.087
122.931
86.805
82.424
77.368
77.256
77.050
76.733
54.766
49.674
38.899
23.265
15.603
f1 (ppm)

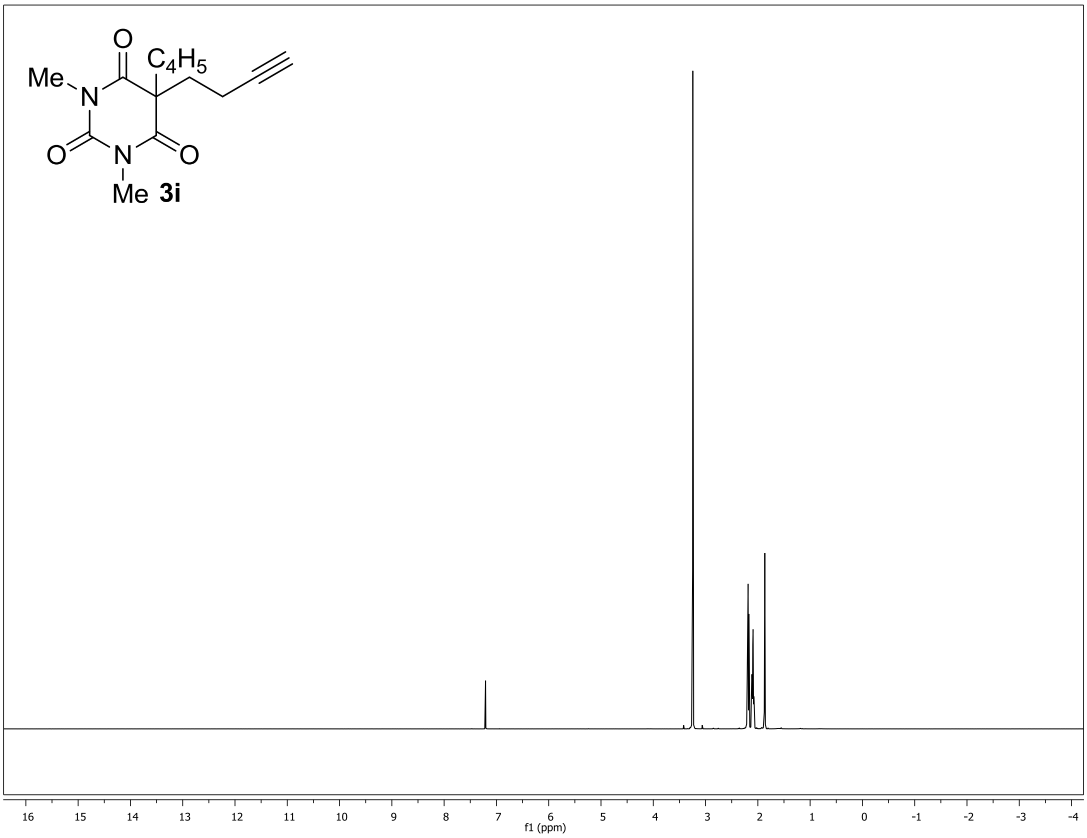
O
C_4H_5
Me
N
O
N
O
Me 3i
16 15 14 13 12 11 10 9 8 7 6 5 4 3 2 1 0 -1 -2 -3 -4
f1 (ppm)

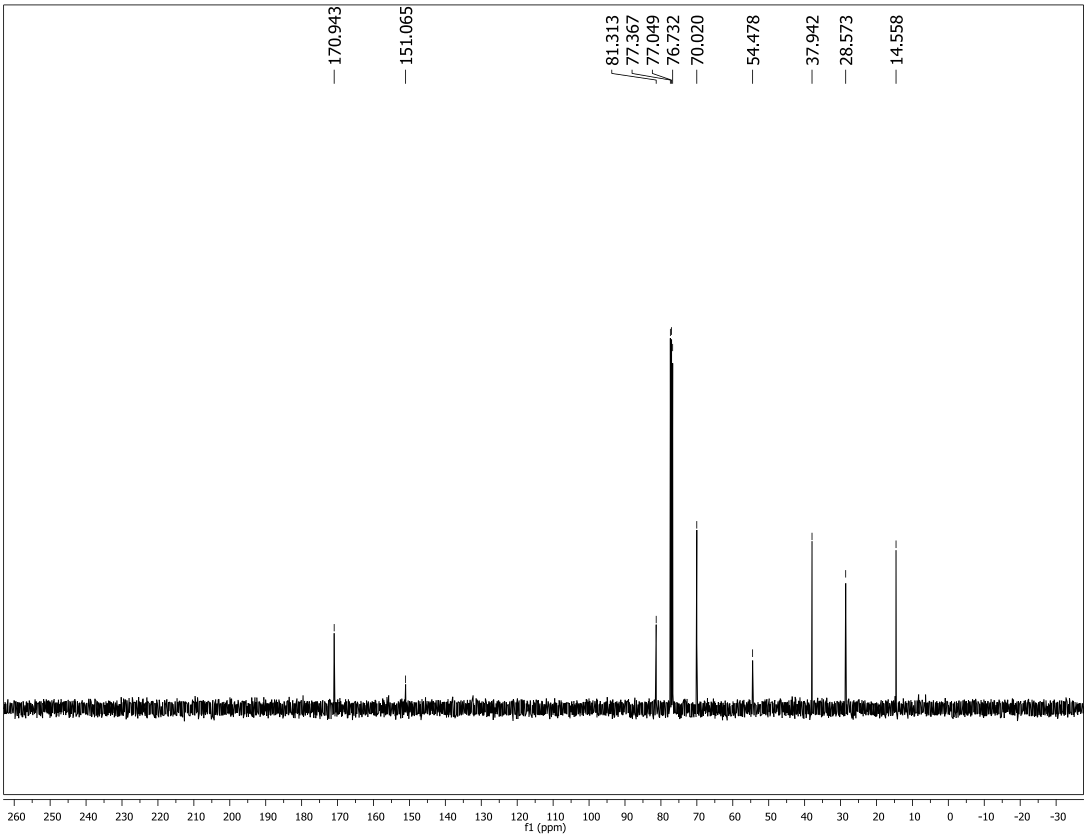
170.943
151.065
81.313
77.367
77.049
76.732
70.020
54.478
37.942
28.573
14.558
260 250 240 230 220 210 200 190 180 170 160 150 140 130 120 110 100 90 80 70 60 50 40 30 20 10 0 -10 -20 -30
f1 (ppm)

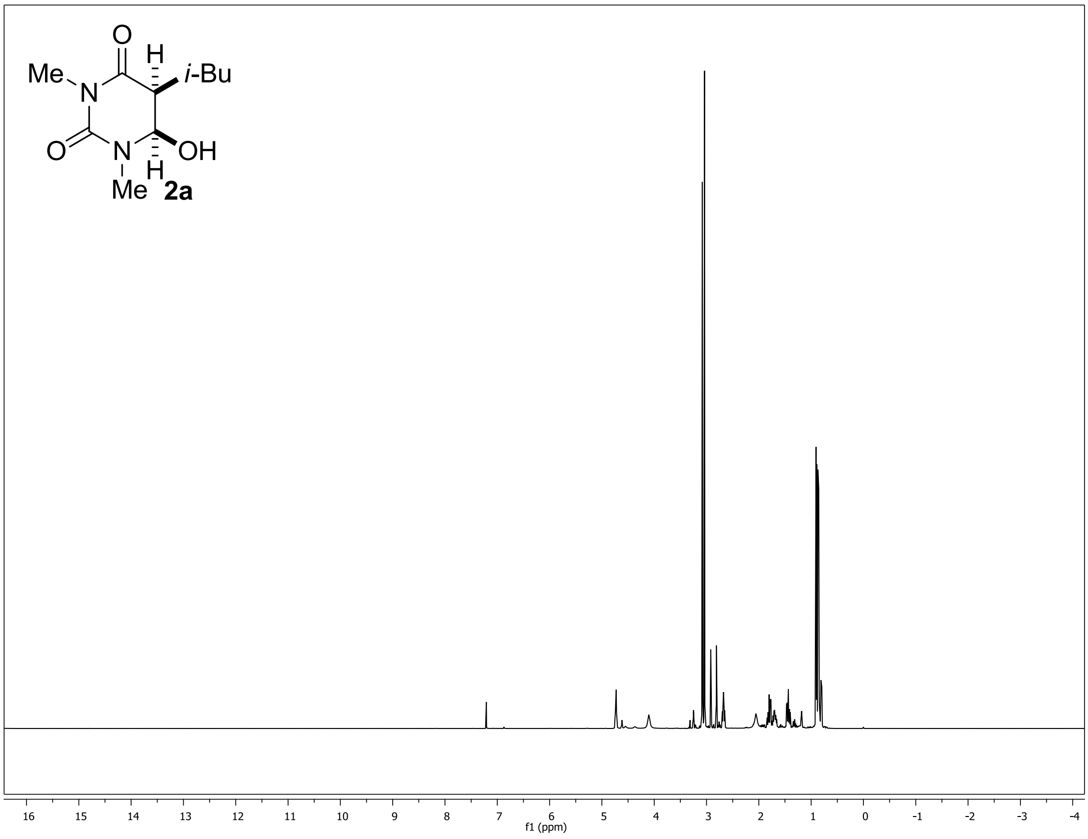
Me
N
O
H
i-Bu
O
N
OH
H
Me
2a
16 15 14 13 12 11 10 9 8 7 6 5 4 3 2 1 0 -1 -2 -3 -4
f1 (ppm)

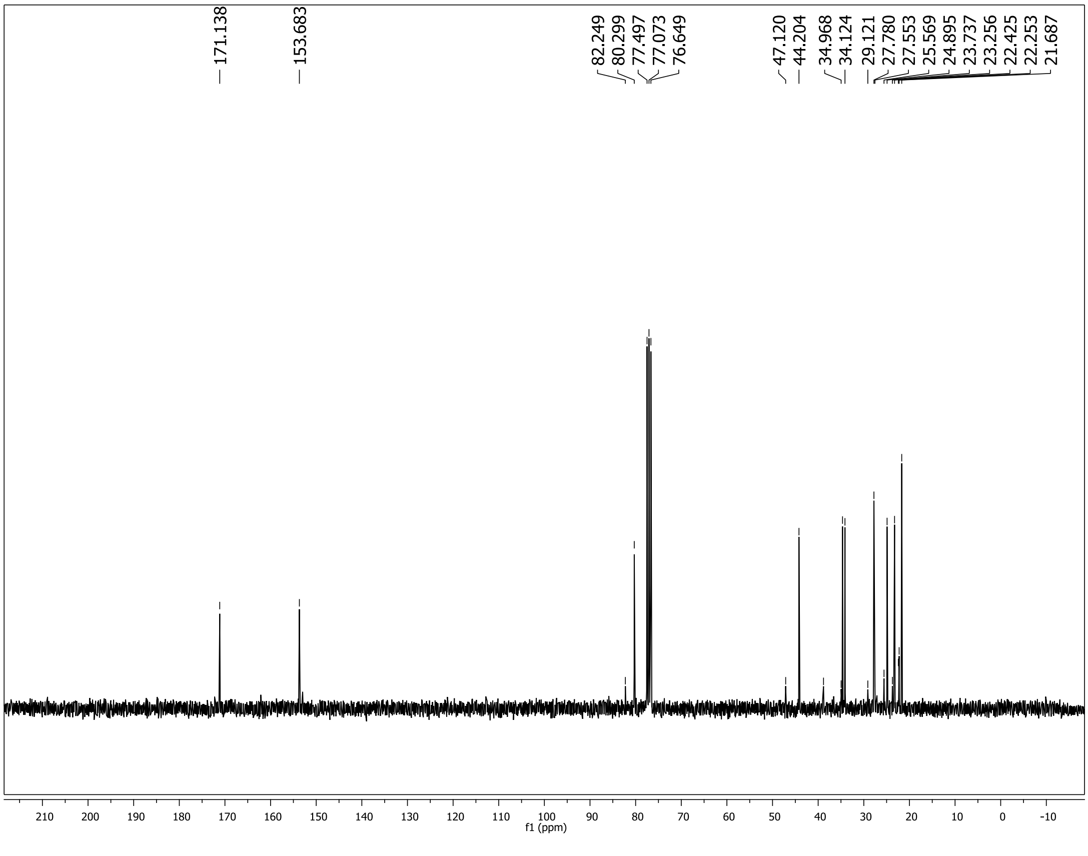
171.138
153.683
82.249
80.299
77.497
77.073
76.649
47.120
44.204
34.968
34.124
29.121
27.780
27.553
25.569
24.895
23.737
23.256
22.425
22.253
21.687
210 200 190 180 170 160 150 140 130 120 110 100 90 80 70 60 50 40 30 20 10 0 -10
f1 (ppm)

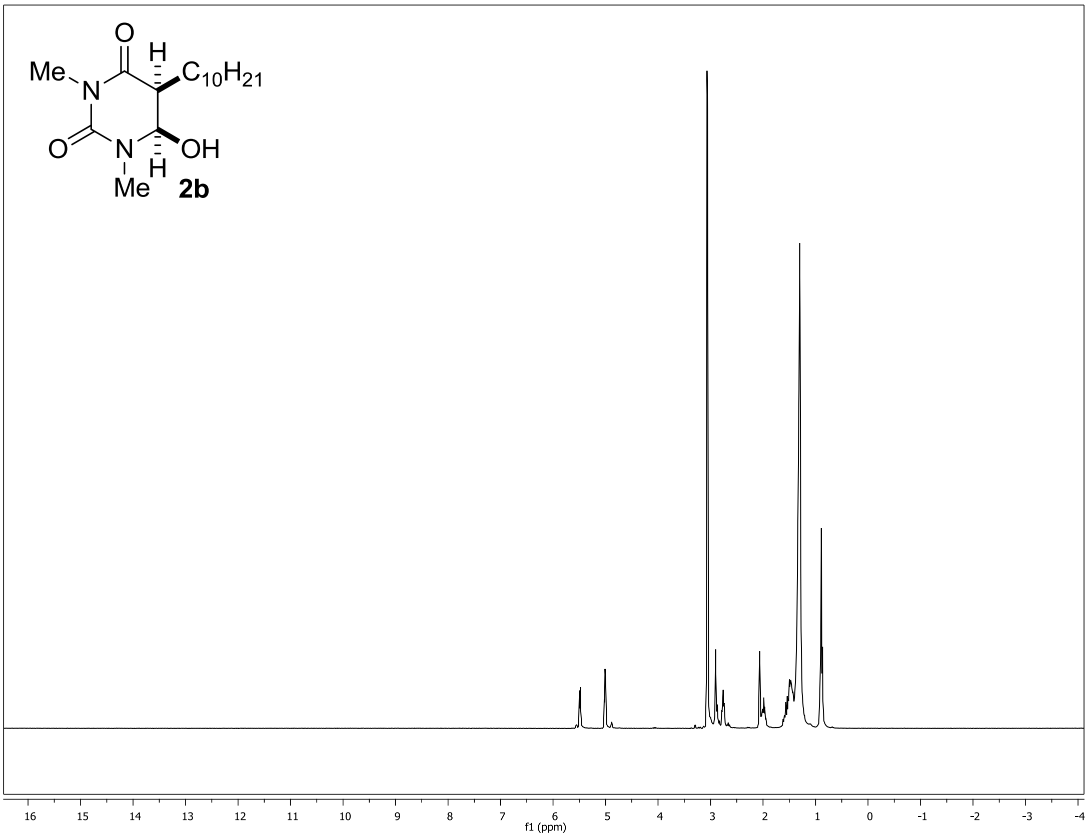

Me
N
O
H
C10H21
O
N
OH
H
Me
2b
16 15 14 13 12 11 10 9 8 7 6 5 4 3 2 1 0 -1 -2 -3 -4
f1 (ppm)

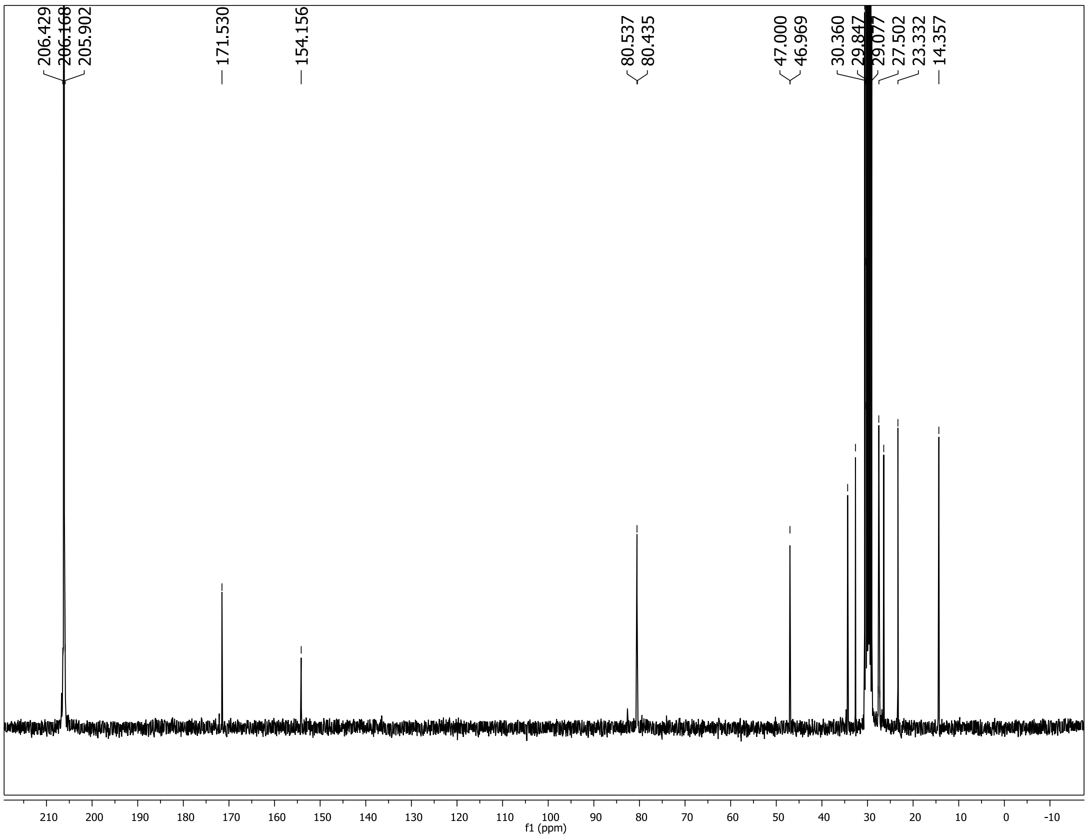

206.429
206.160
205.902
171.530
154.156
80.537
80.435
47.000
46.969
30.360
29.847
29.077
27.502
23.332
14.357
210 200 190 180 170 160 150 140 130 120 110 100 90 80 70 60 50 40 30 20 10 0 -10
f1 (ppm)

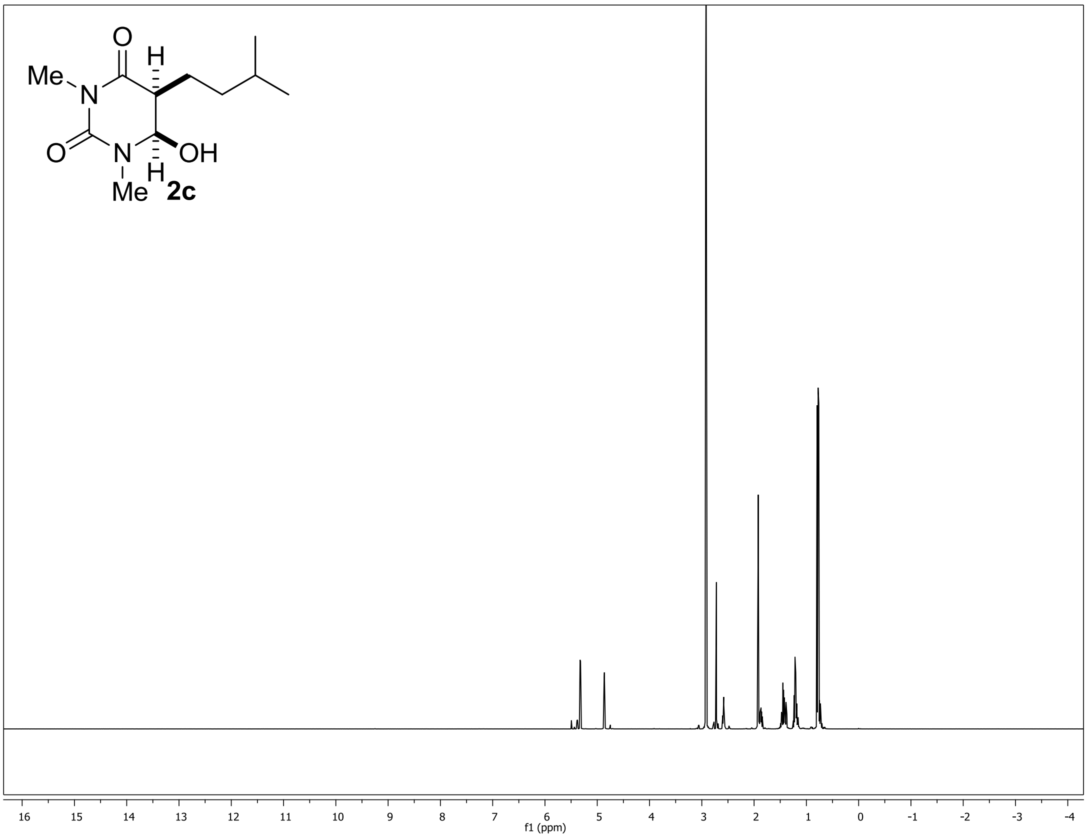
Me
N
O
H
O
N
OH
H
Me
2c
16 15 14 13 12 11 10 9 8 7 6 5 4 3 2 1 0 -1 -2 -3 -4
f1 (ppm)

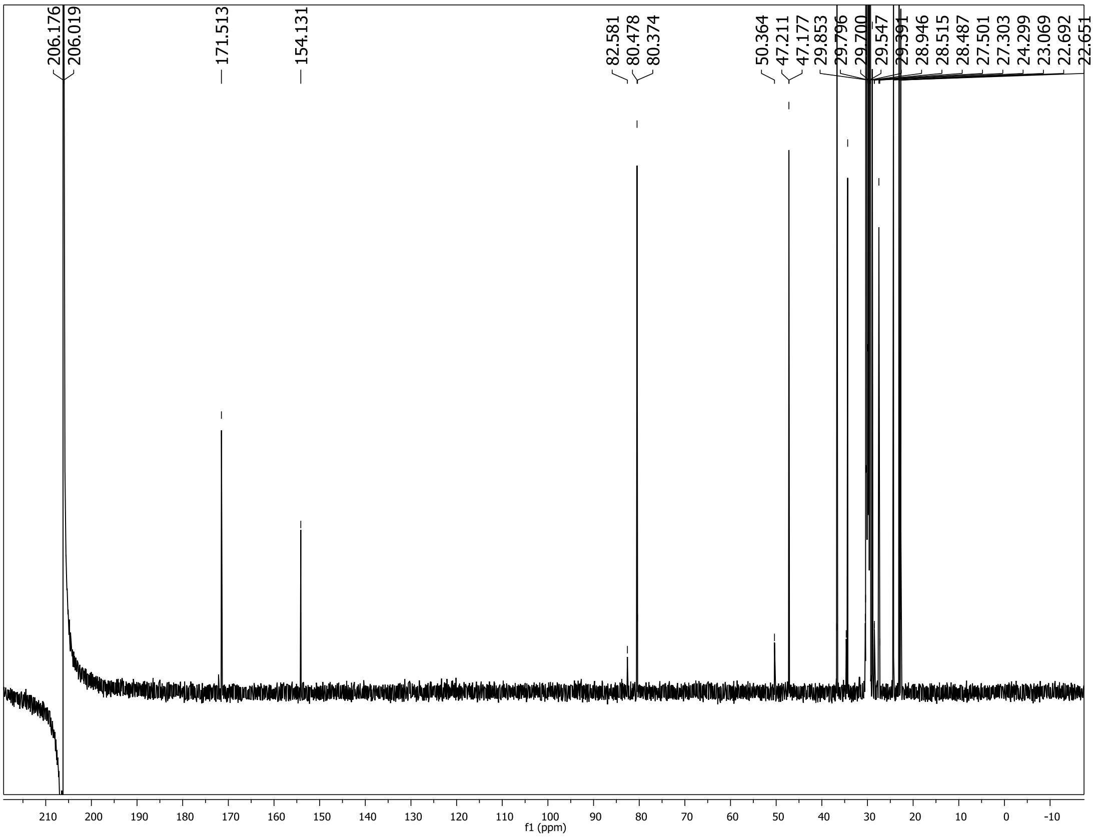
206.176
206.019
171.513
154.131
82.581
80.478
80.374
50.364
47.211
47.177
29.853
29.796
29.700
29.547
29.391
28.946
28.515
28.487
27.501
27.303
24.299
23.069
22.692
22.651
210 200 190 180 170 160 150 140 130 120 110 100 90 80 70 60 50 40 30 20 10 0 -10
f1 (ppm)

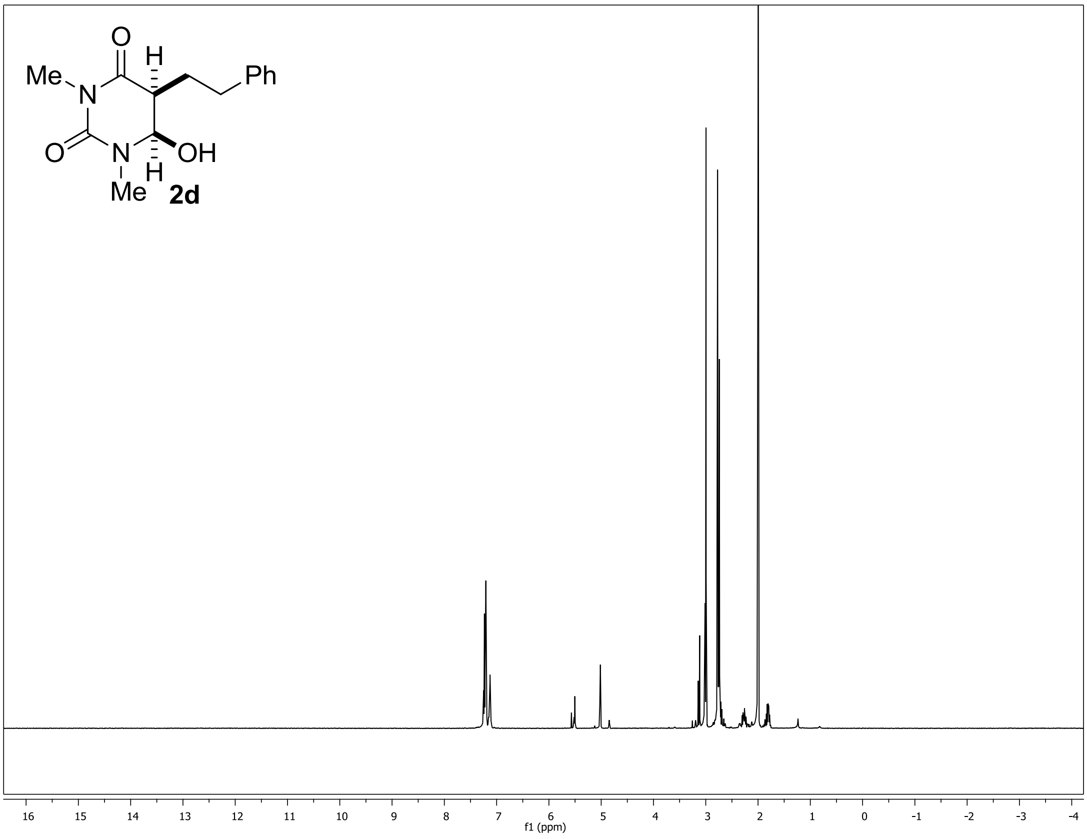
Me
N
O
H
Ph
O
N
OH
H
Me
2d
f1 (ppm)

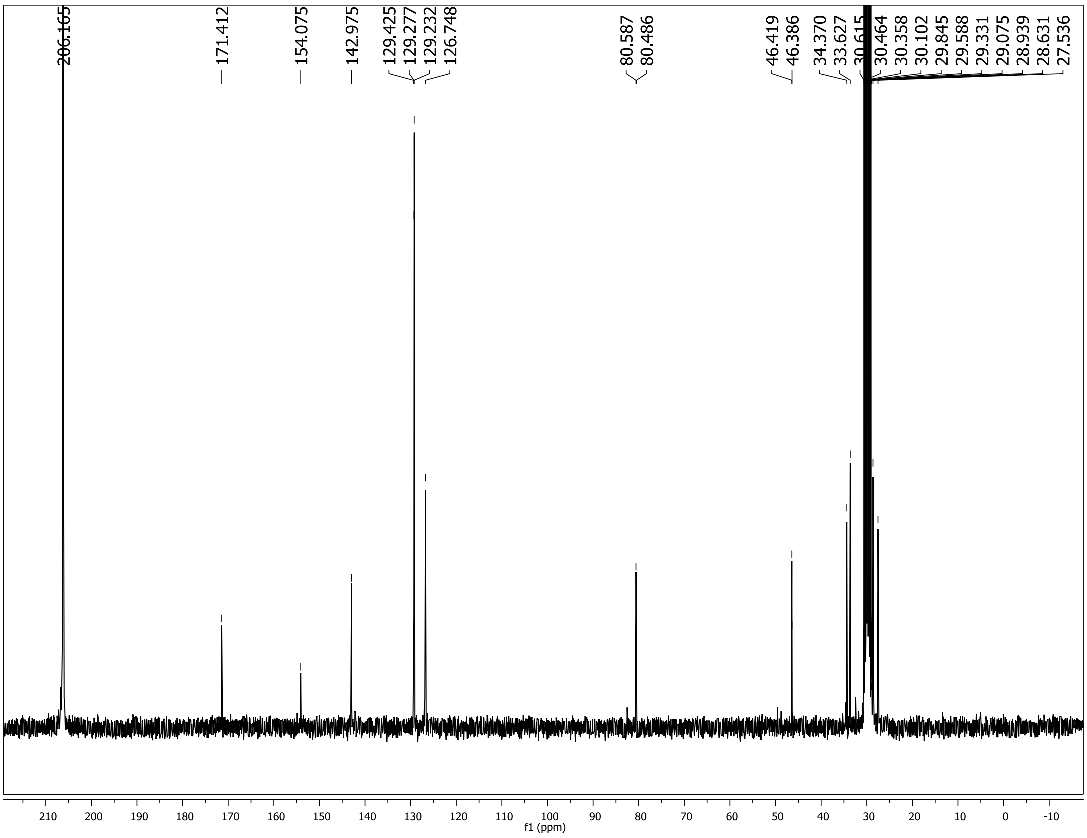
206.165
171.412
154.075
142.975
129.425
129.277
129.232
126.748
80.587
80.486
46.419
46.386
34.370
33.627
30.615
30.464
30.358
30.102
29.845
29.588
29.331
29.075
28.939
28.631
27.536
f1 (ppm)

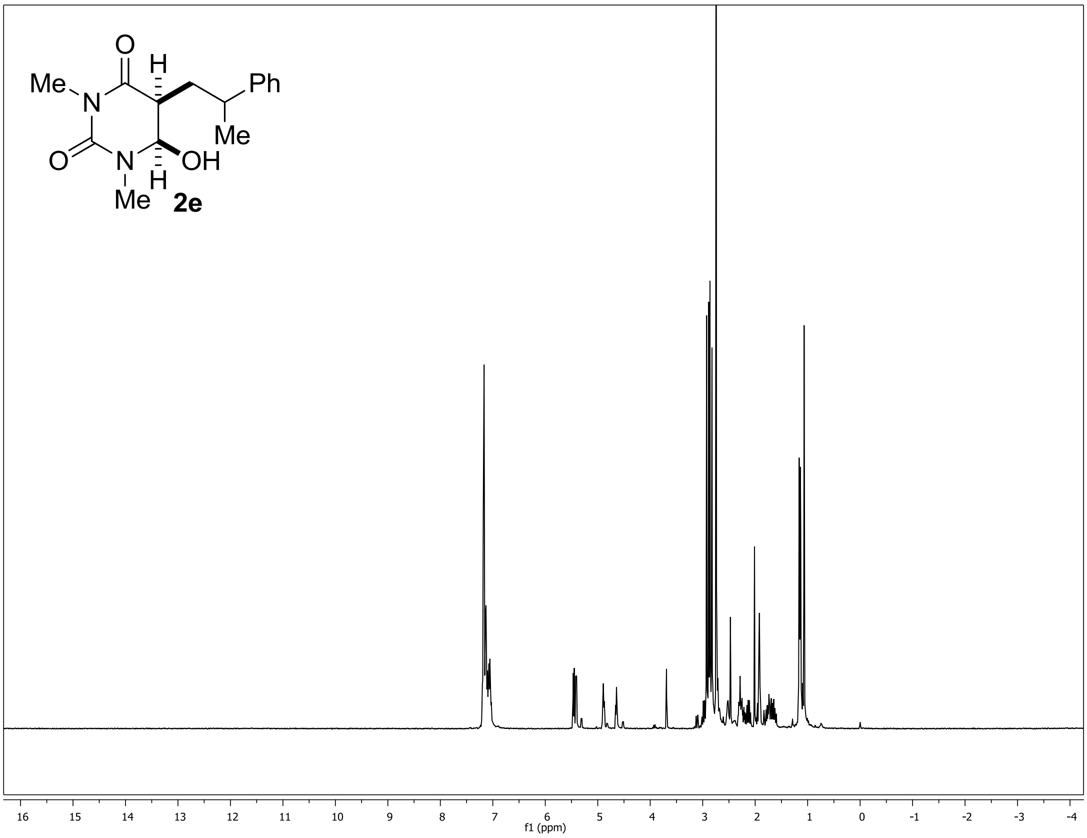
O
H
Me
N
Ph
Me
O
N
OH
H
Me
2e
16 15 14 13 12 11 10 9 8 7 6 5 4 3 2 1 0 -1 -2 -3 -4
f1 (ppm)

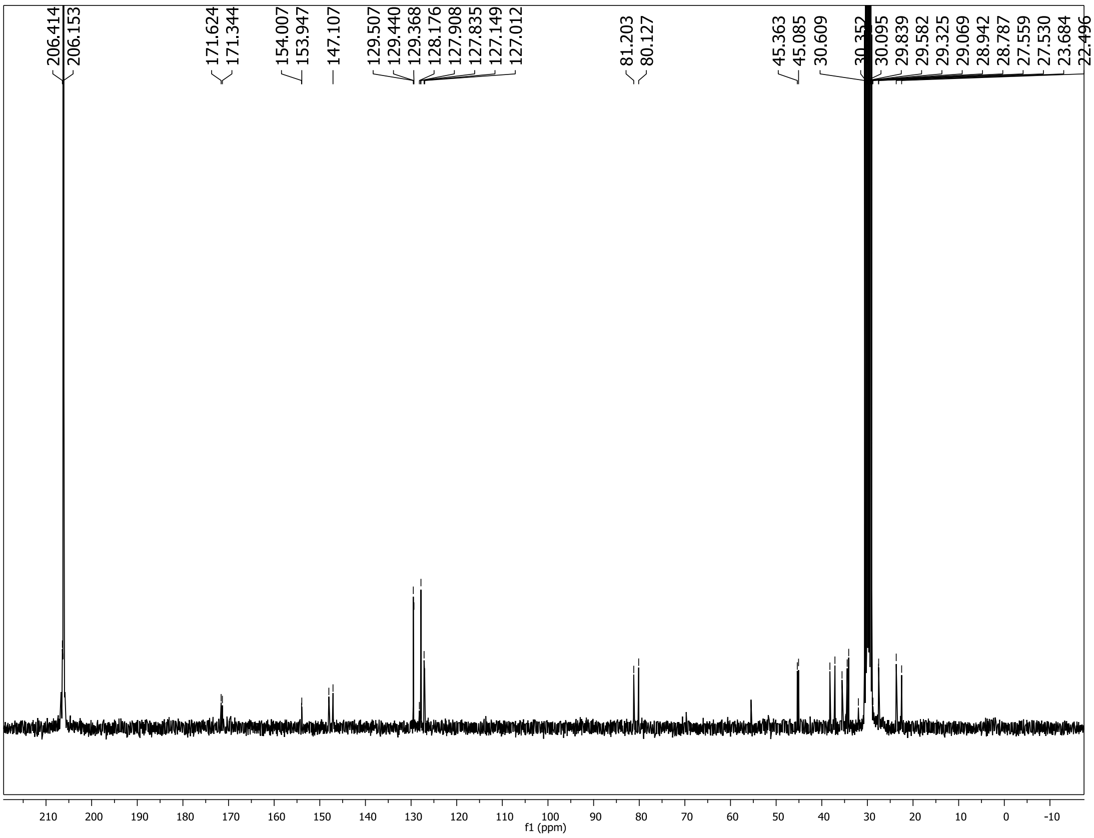
206.414
206.153
171.624
171.344
154.007
153.947
147.107
129.507
129.440
129.368
128.176
127.908
127.835
127.149
127.012
81.203
80.127
45.363
45.085
30.609
30.352
30.095
29.839
29.582
29.325
29.069
28.942
28.787
27.559
27.530
23.684
22.496
210 200 190 180 170 160 150 140 130 120 110 100 90 80 70 60 50 40 30 20 10 0 -10
f1 (ppm)

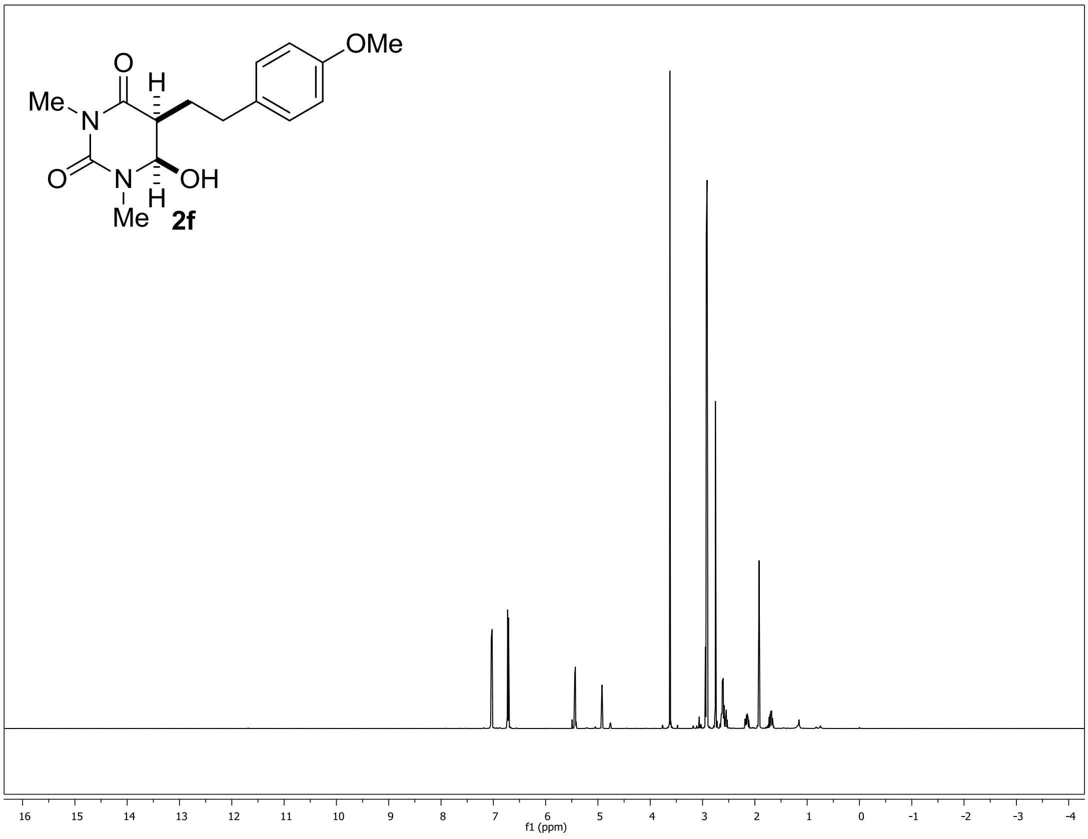
OMe
O
H
Me
N
O
N
OH
H
Me
2f
f1 (ppm)

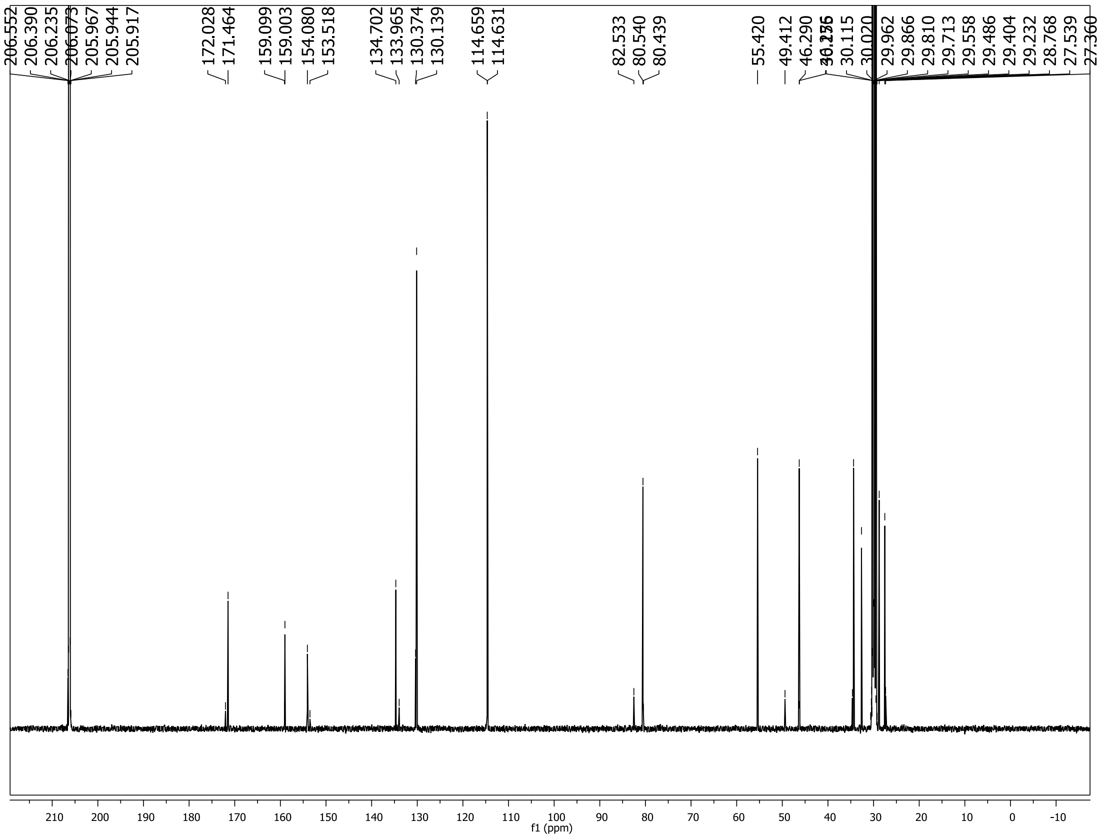
206.552
206.390
206.235
206.073
205.967
205.944
205.917
172.028
171.464
159.099
159.003
154.080
153.518
134.702
133.965
130.374
130.139
114.659
114.631
82.533
80.540
80.439
55.420
49.412
46.290
30.115
30.020
29.962
29.866
29.810
29.713
29.558
29.486
29.404
29.232
28.768
27.539
27.360
f1 (ppm)

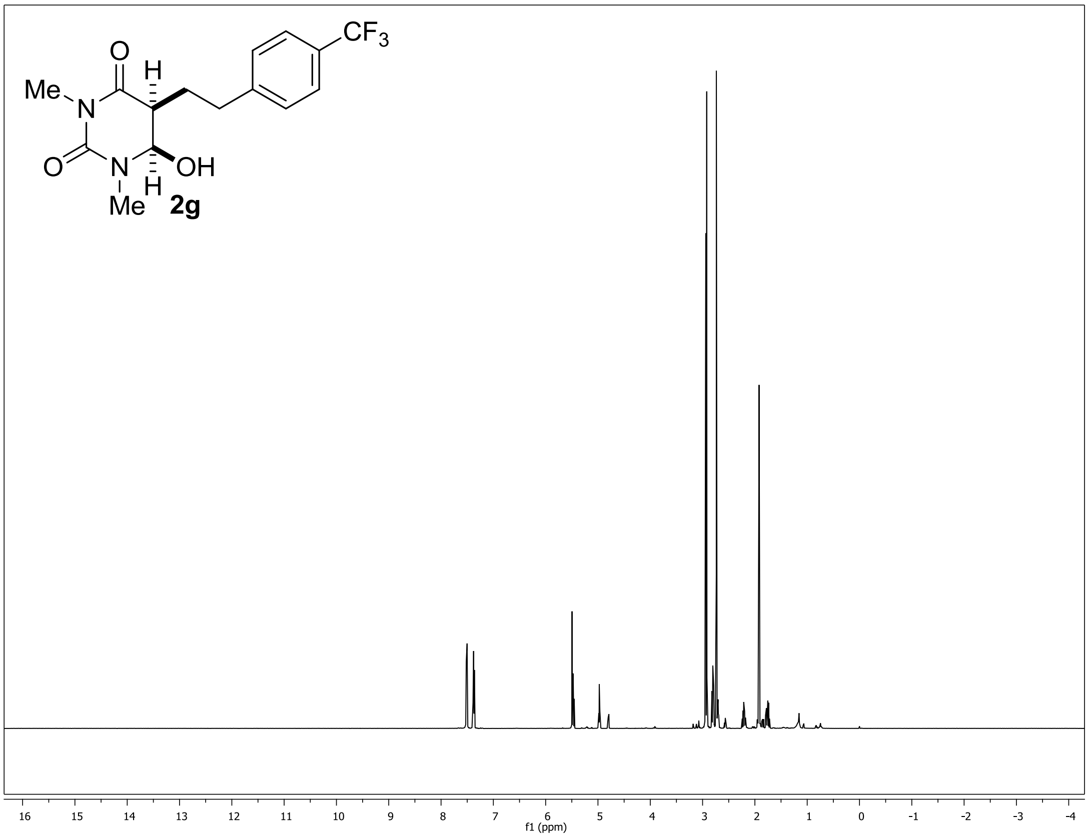
CF3
Me
N
O
H
OH
Me
2g
f1 (ppm)

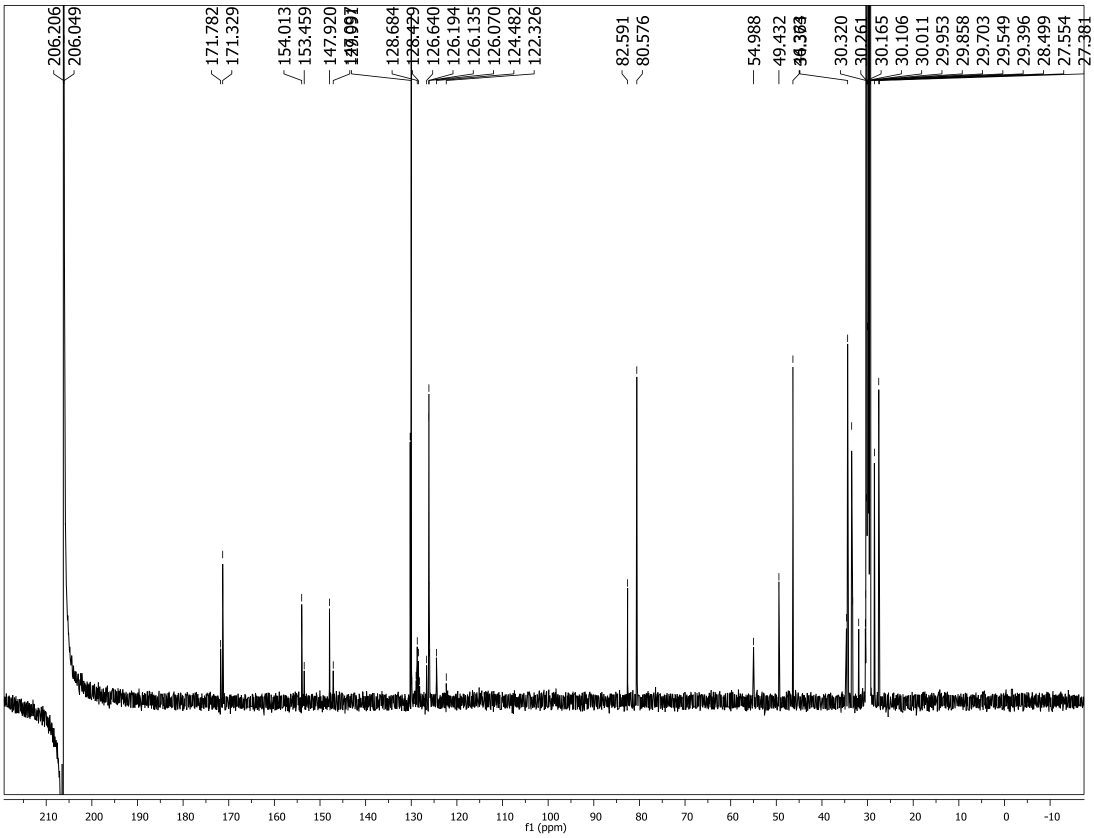
206.206
206.049
171.782
171.329
154.013
153.459
147.920
128.684
128.429
126.640
126.194
126.135
126.070
124.482
122.326
82.591
80.576
54.988
49.432
30.320
30.261
30.165
30.106
30.011
29.953
29.858
29.703
29.549
29.396
28.499
27.554
27.381
f1 (ppm)

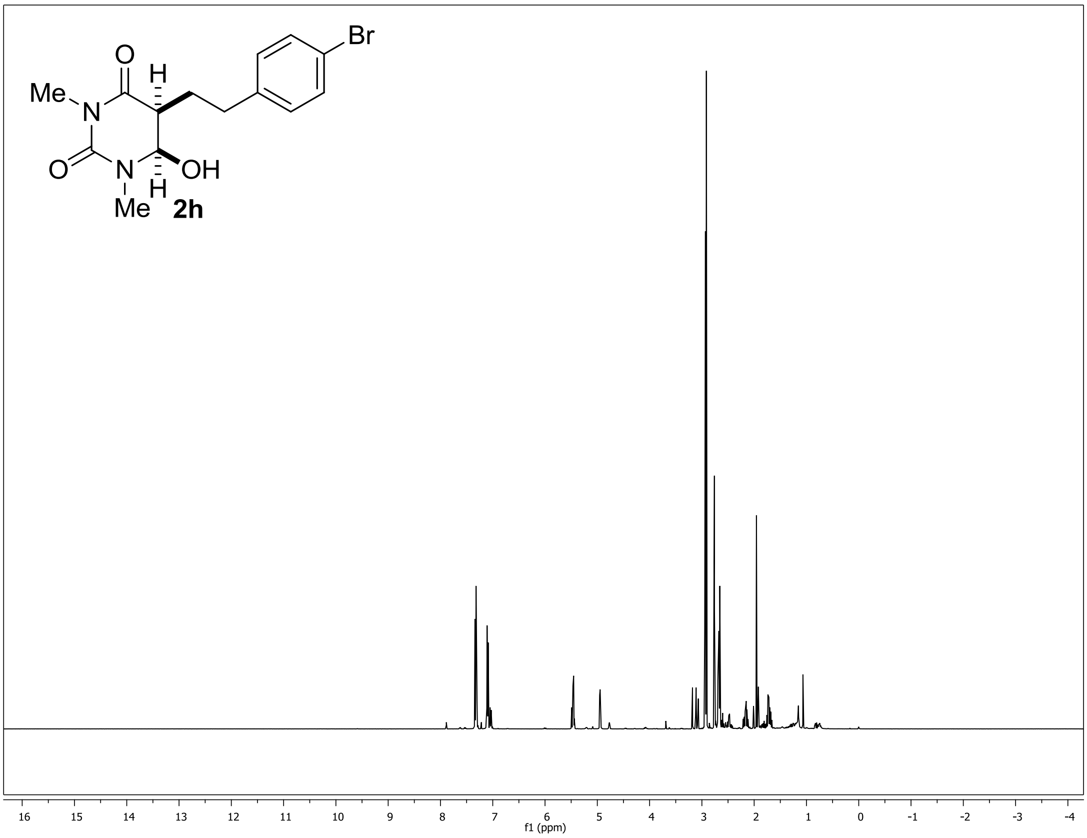
Br
O
H
Me
N
O
N
OH
H
Me
2h
f1 (ppm)

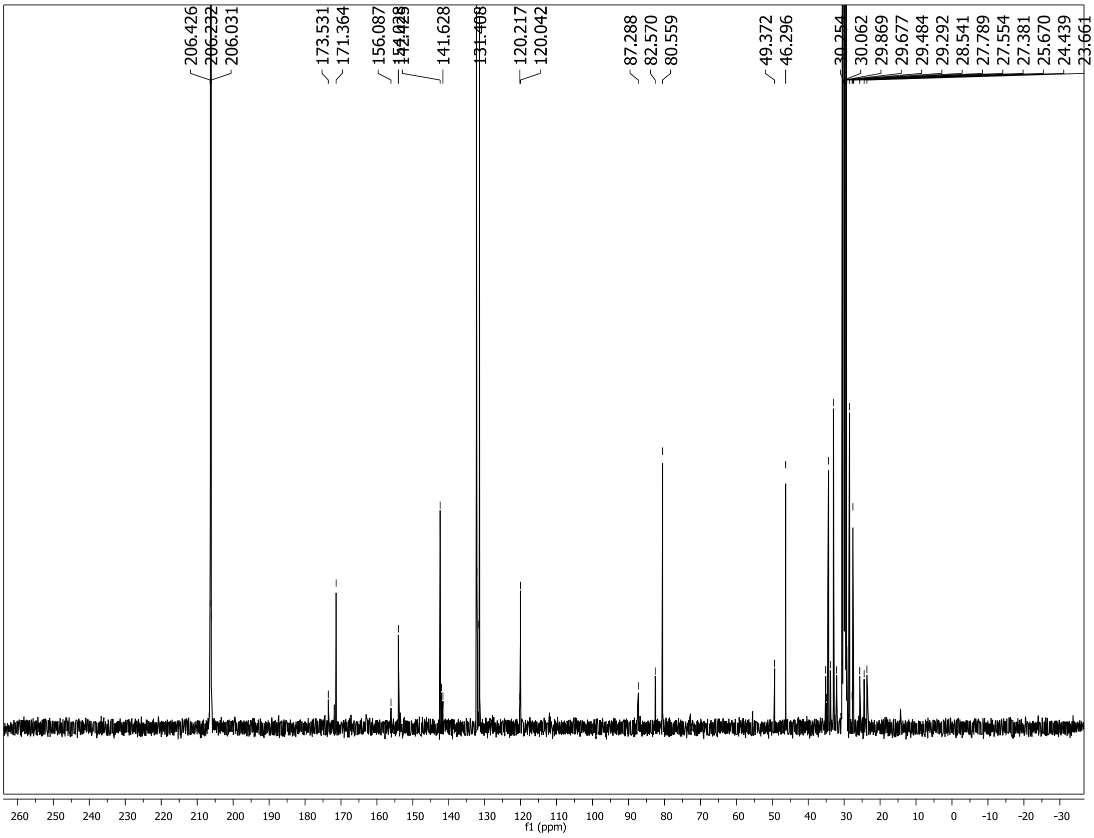
206.426
206.232
206.031
173.531
171.364
156.087
141.628
131.408
120.217
120.042
87.288
82.570
80.559
49.372
46.296
30.062
29.869
29.677
29.484
29.292
28.541
27.789
27.554
27.381
25.670
24.439
23.661
f1 (ppm)

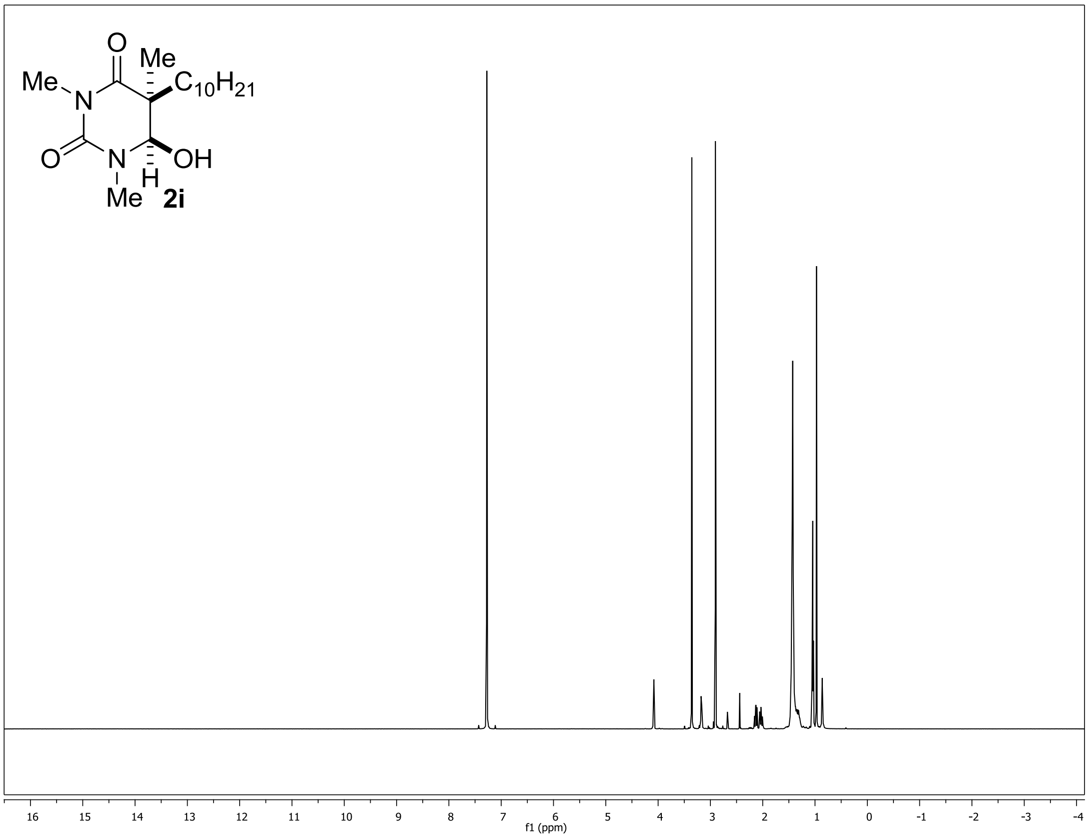
Me
Me
N
C10H21
O
N
OH
H
Me
2i
16 15 14 13 12 11 10 9 8 7 6 5 4 3 2 1 0 -1 -2 -3 -4
f1 (ppm)

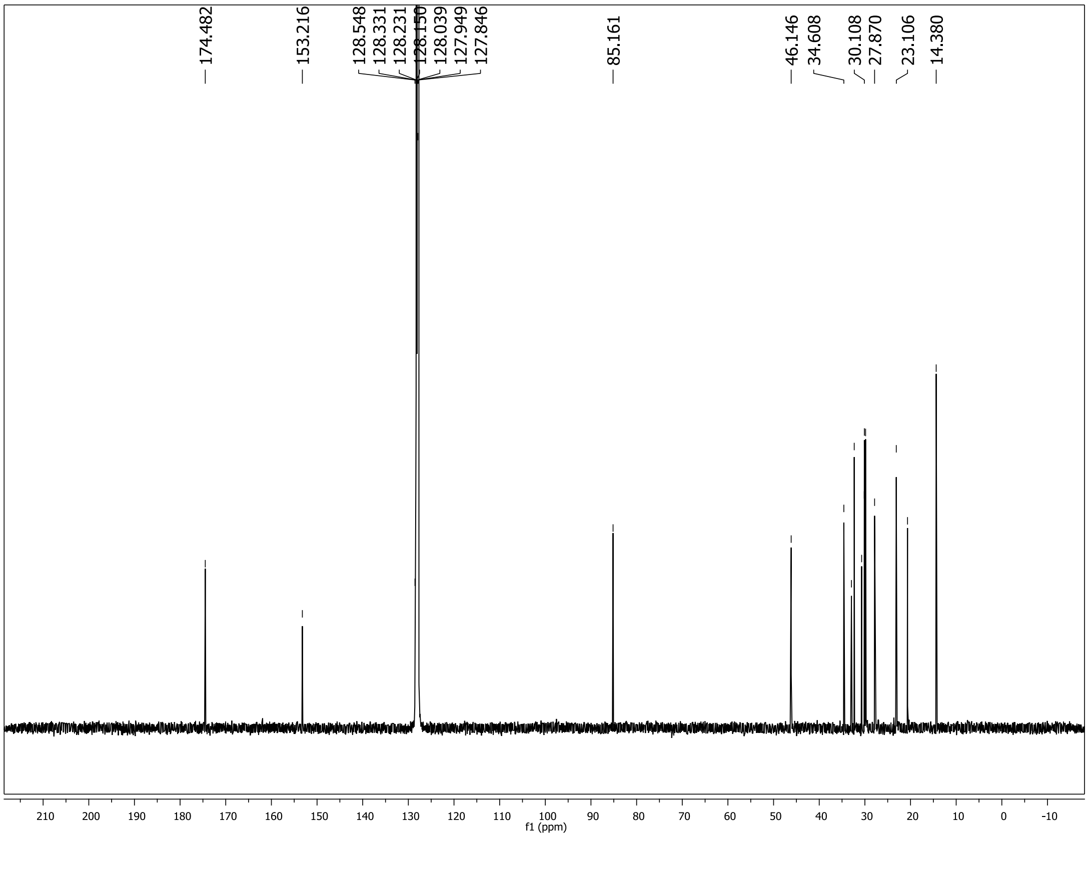
174.482
153.216
128.548
128.331
128.231
128.150
128.039
127.949
127.846
85.161
46.146
34.608
30.108
27.870
23.106
14.380
210 200 190 180 170 160 150 140 130 120 110 100 90 80 70 60 50 40 30 20 10 0 -10
f1 (ppm)

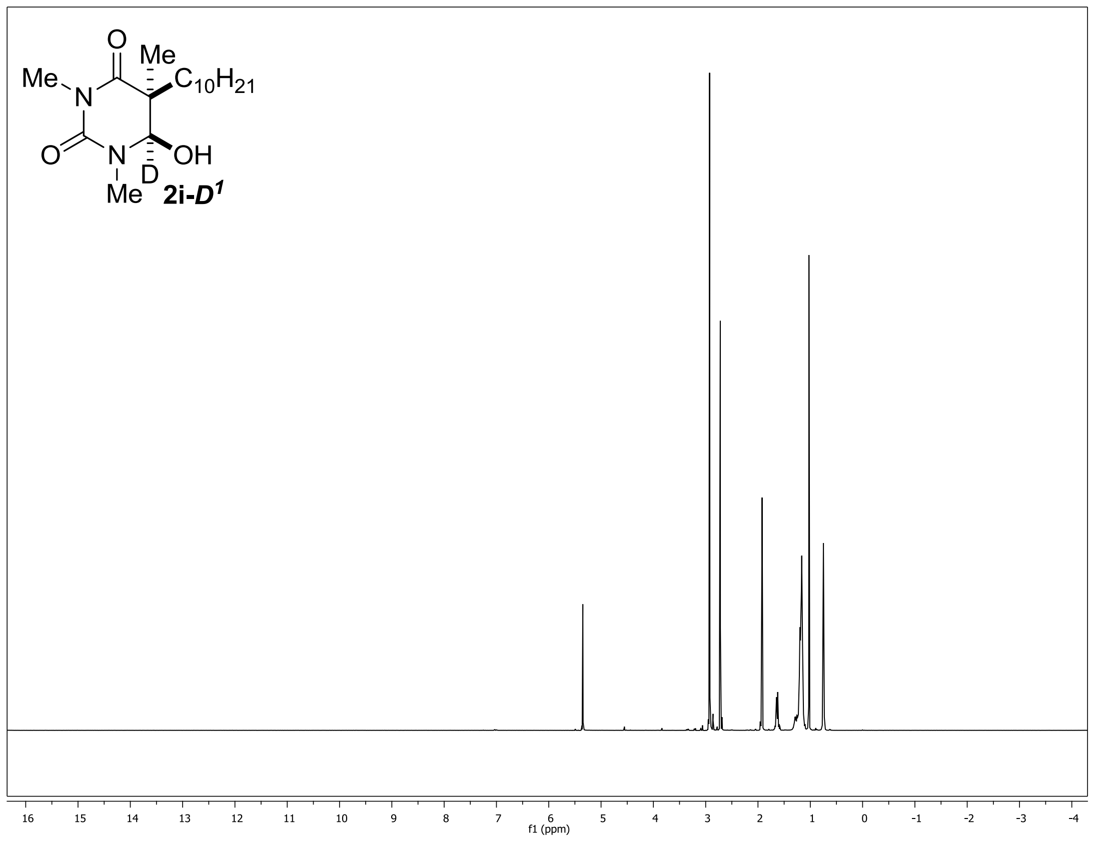
Me
C10H21
Me
N
O
O
N
D
OH
Me
2i-D1
f1 (ppm)

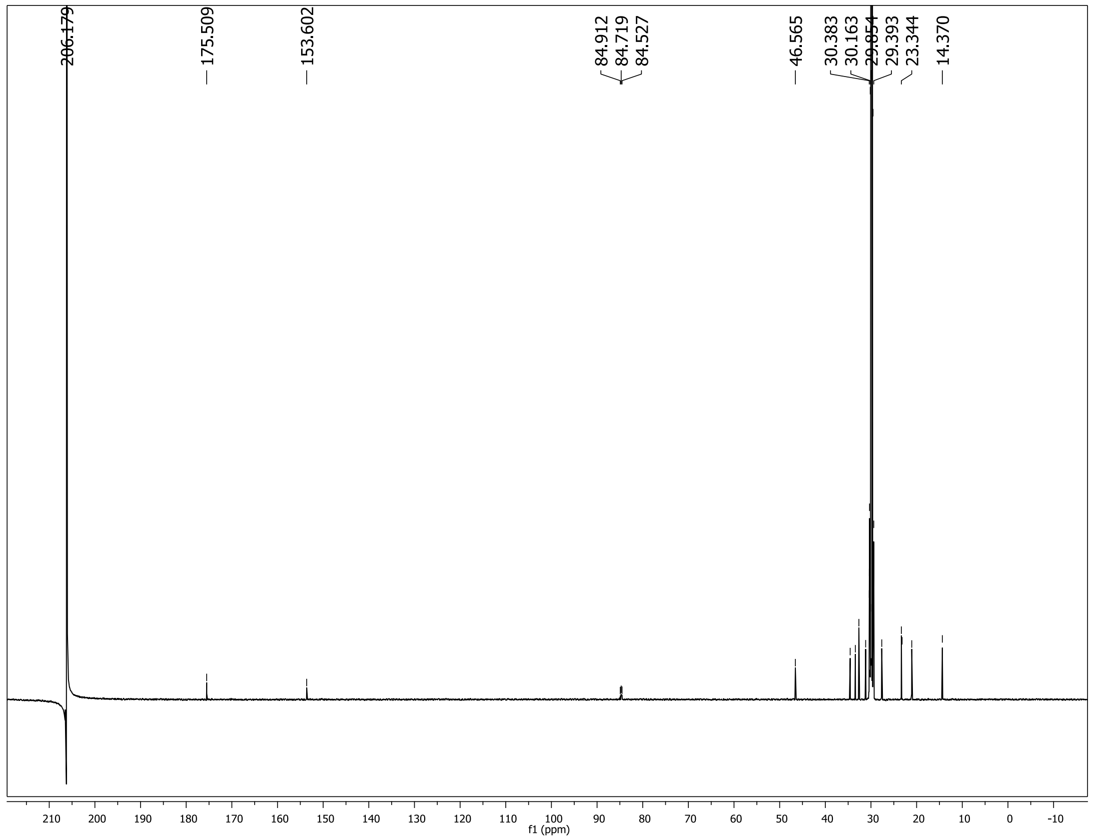
206.179
175.509
153.602
84.912
84.719
84.527
46.565
30.383
30.163
29.854
29.393
23.344
14.370
f1 (ppm)

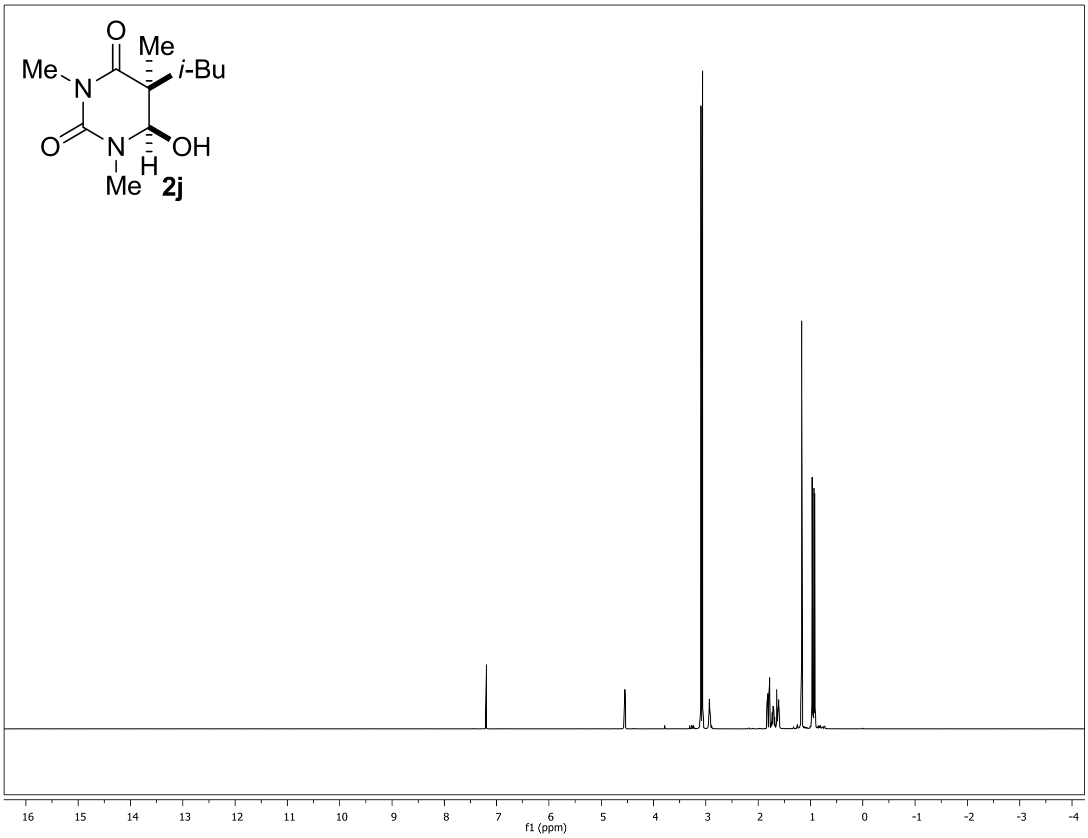
Me
Me
i-Bu
N
O
N
OH
H
Me
2j
f1 (ppm)

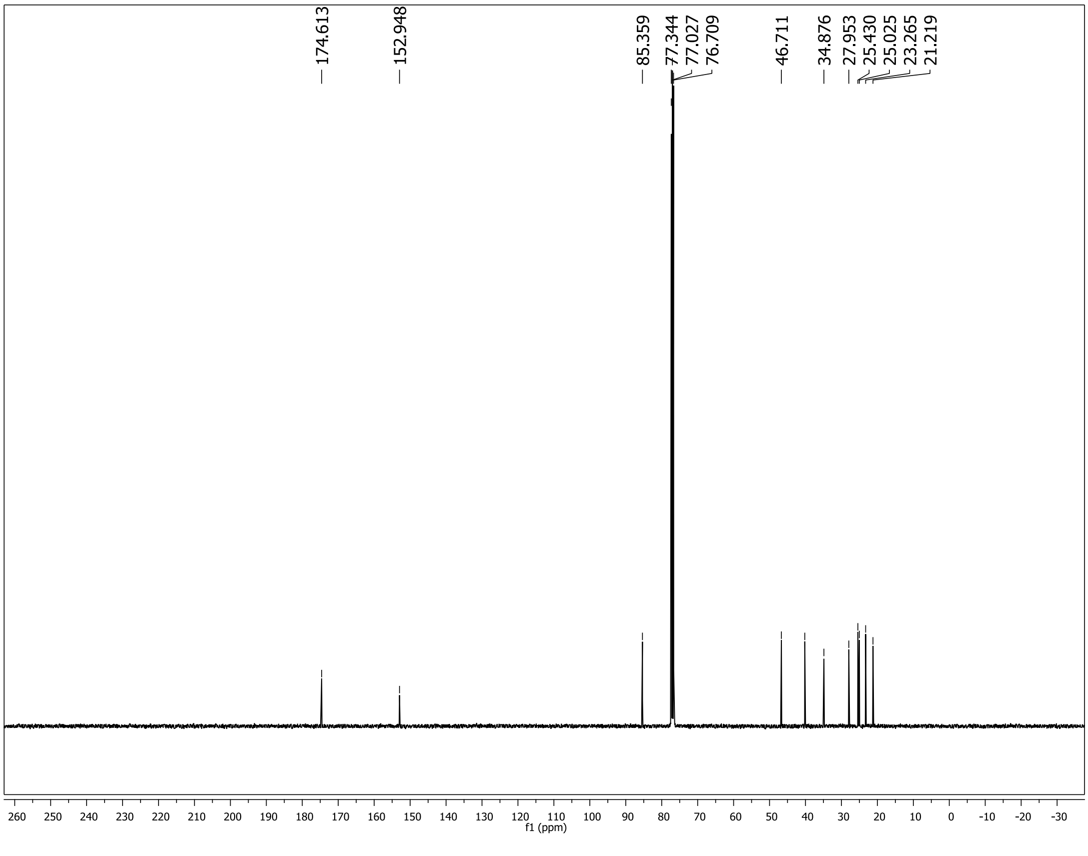
174.613
152.948
85.359
77.344
77.027
76.709
46.711
34.876
27.953
25.430
25.025
23.265
21.219
f1 (ppm)

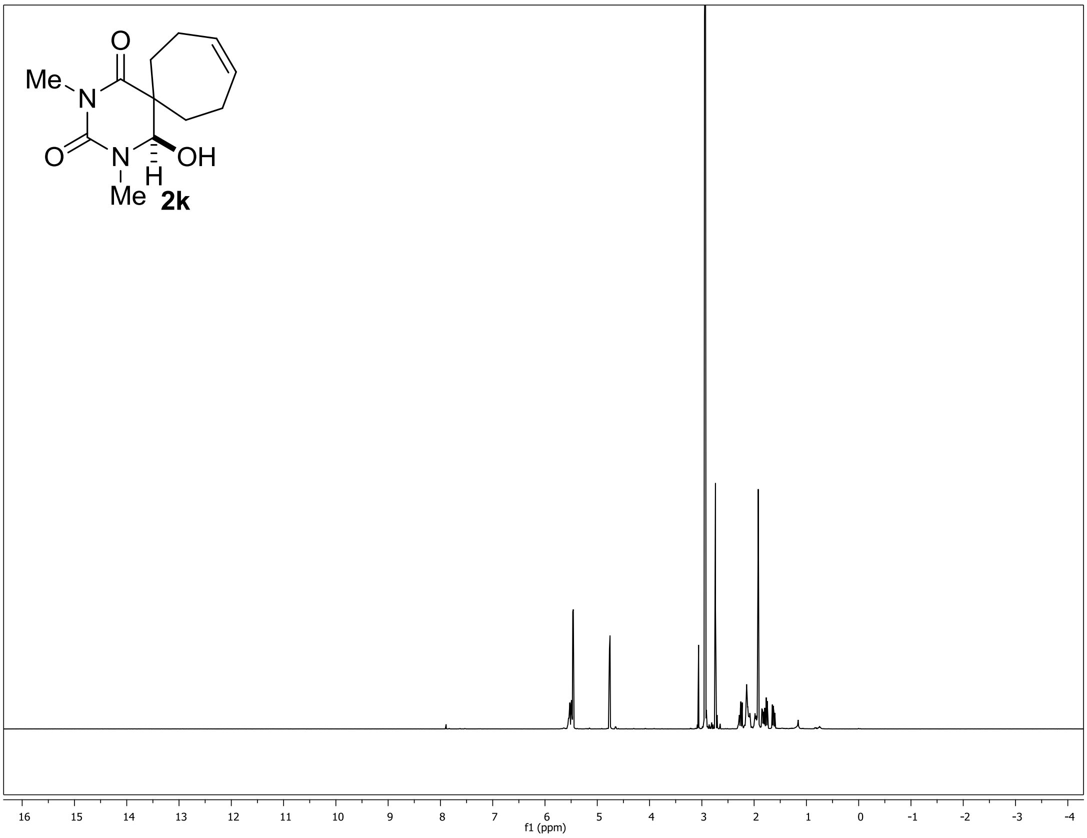
Me
N
O
O
N
OH
H
Me
2k
16 15 14 13 12 11 10 9 8 7 6 5 4 3 2 1 0 -1 -2 -3 -4
f1 (ppm)

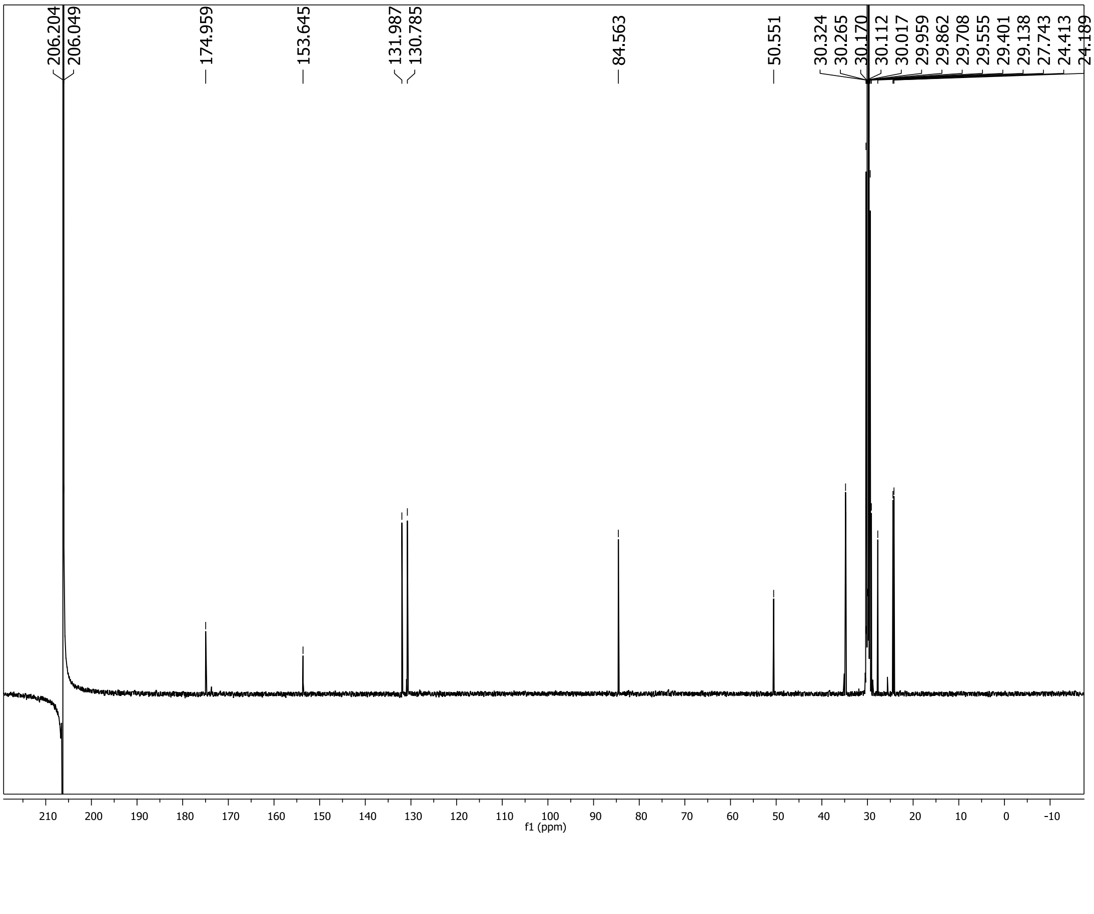
206.204
206.049
174.959
153.645
131.987
130.785
84.563
50.551
30.324
30.265
30.170
30.112
30.017
29.959
29.862
29.708
29.555
29.401
29.138
27.743
24.413
24.189
210 200 190 180 170 160 150 140 130 120 110 100 90 80 70 60 50 40 30 20 10 0 -10
f1 (ppm)

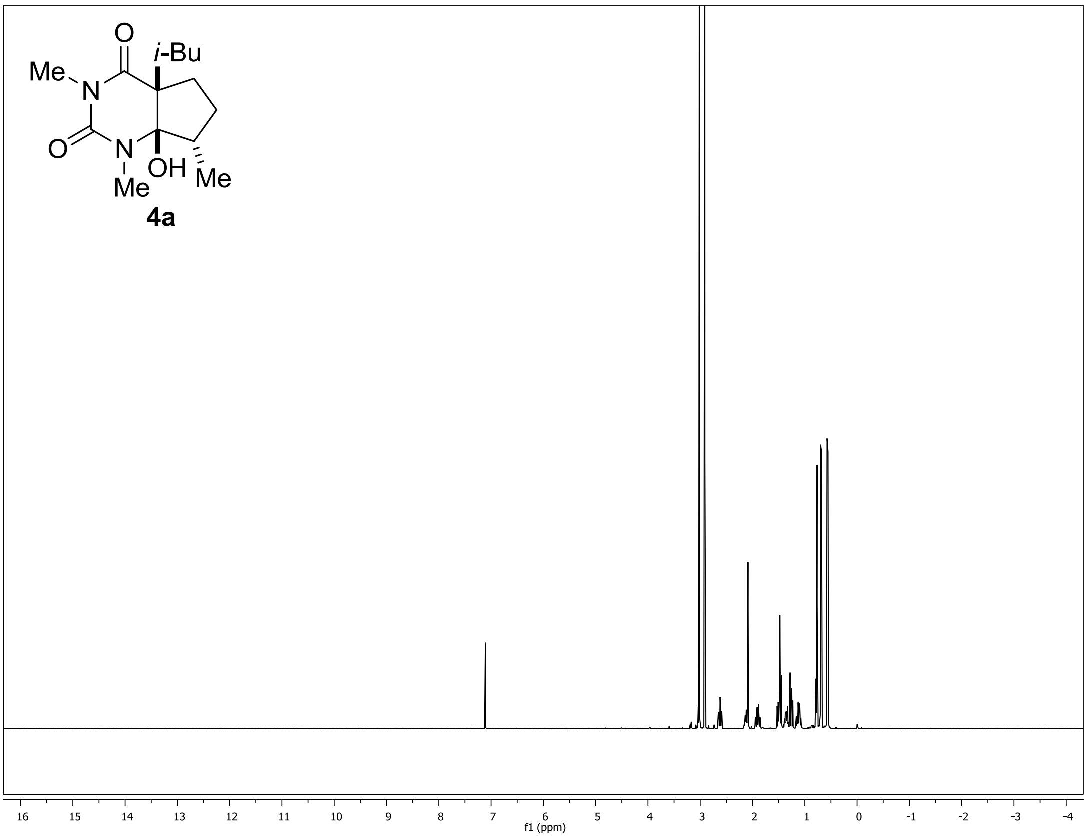
O
i-Bu
Me
N
O
N
OH
Me
Me
4a
16 15 14 13 12 11 10 9 8 7 6 5 4 3 2 1 0 -1 -2 -3 -4
f1 (ppm)

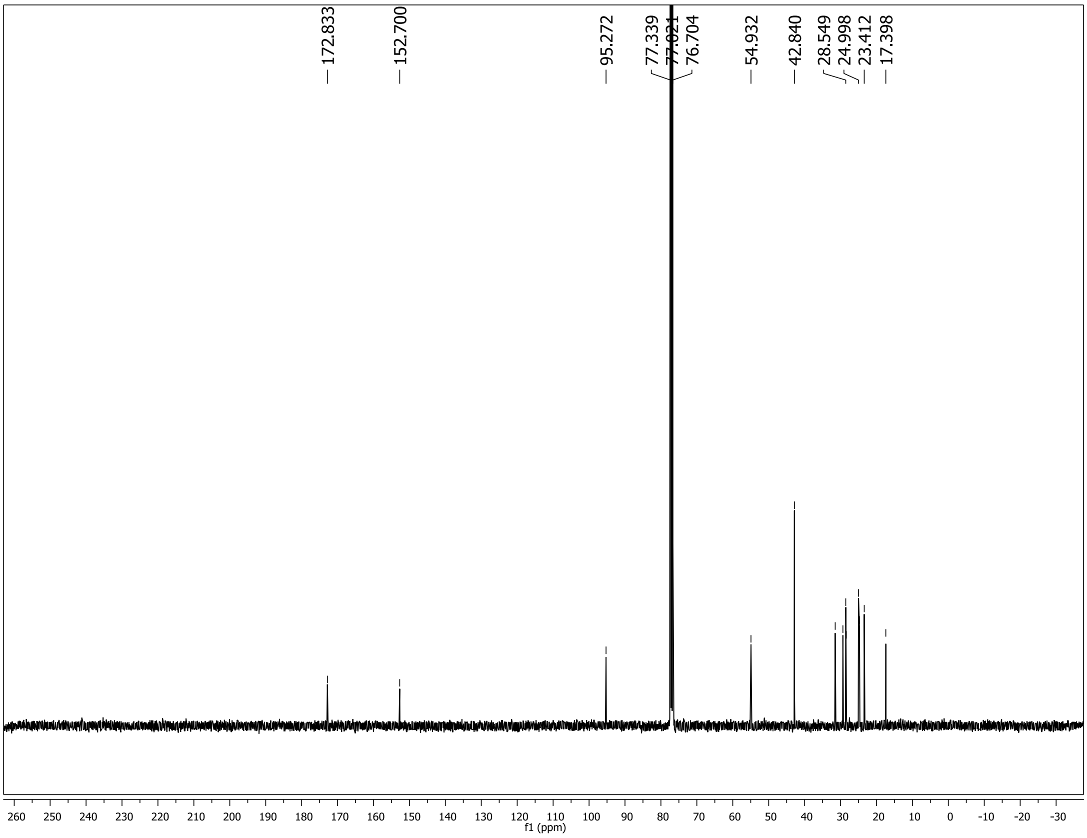
172.833
152.700
95.272
77.339
77.021
76.704
54.932
42.840
28.549
24.998
23.412
17.398
260 250 240 230 220 210 200 190 180 170 160 150 140 130 120 110 100 90 80 70 60 50 40 30 20 10 0 -10 -20 -30
f1 (ppm)

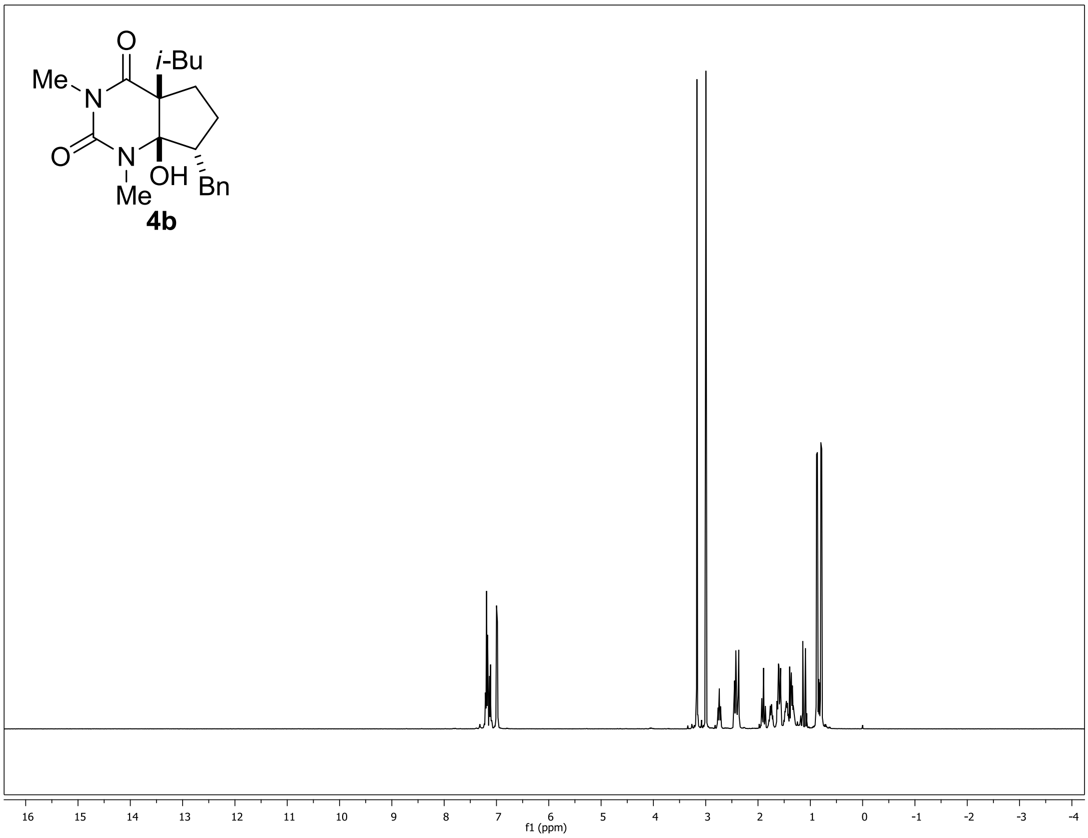

O
i-Bu
Me
N
O
N
OH
Me
Bn
4b
16 15 14 13 12 11 10 9 8 7 6 5 4 3 2 1 0 -1 -2 -3 -4
f1 (ppm)

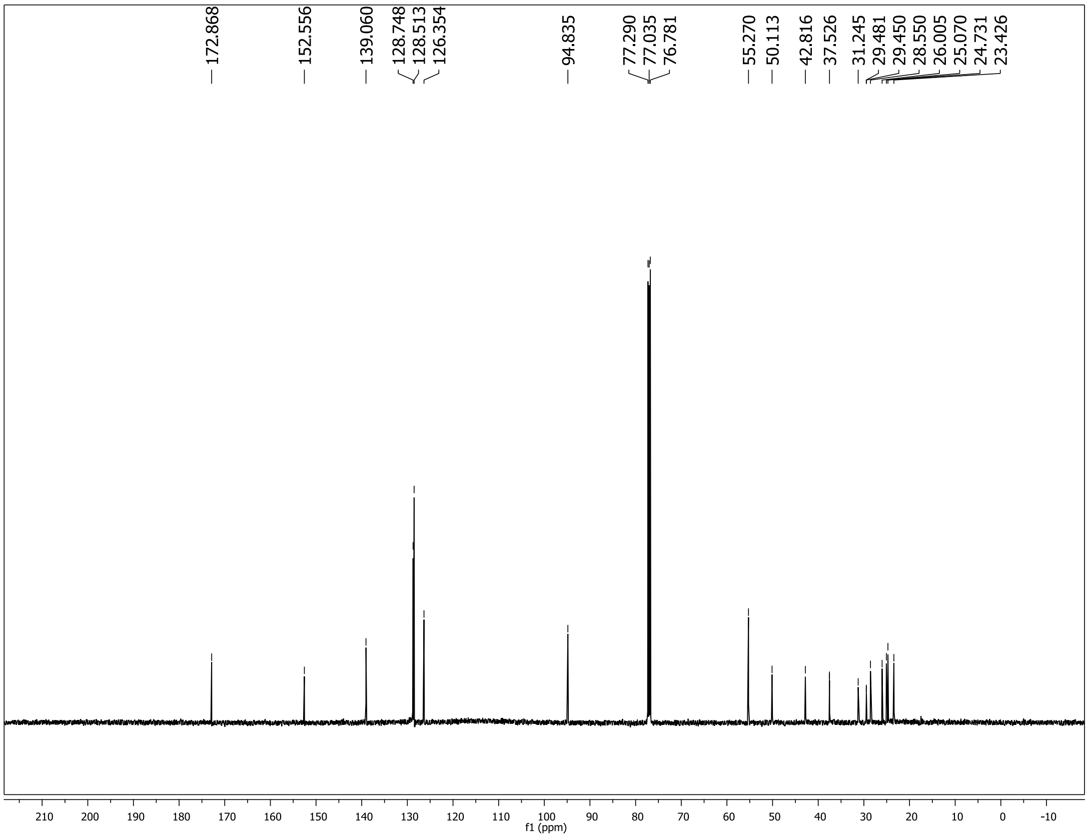

172.868
152.556
139.060
128.748
128.513
126.354
94.835
77.290
77.035
76.781
55.270
50.113
42.816
37.526
31.245
29.481
29.450
28.550
26.005
25.070
24.731
23.426
210 200 190 180 170 160 150 140 130 120 110 100 90 80 70 60 50 40 30 20 10 0 -10
f1 (ppm)

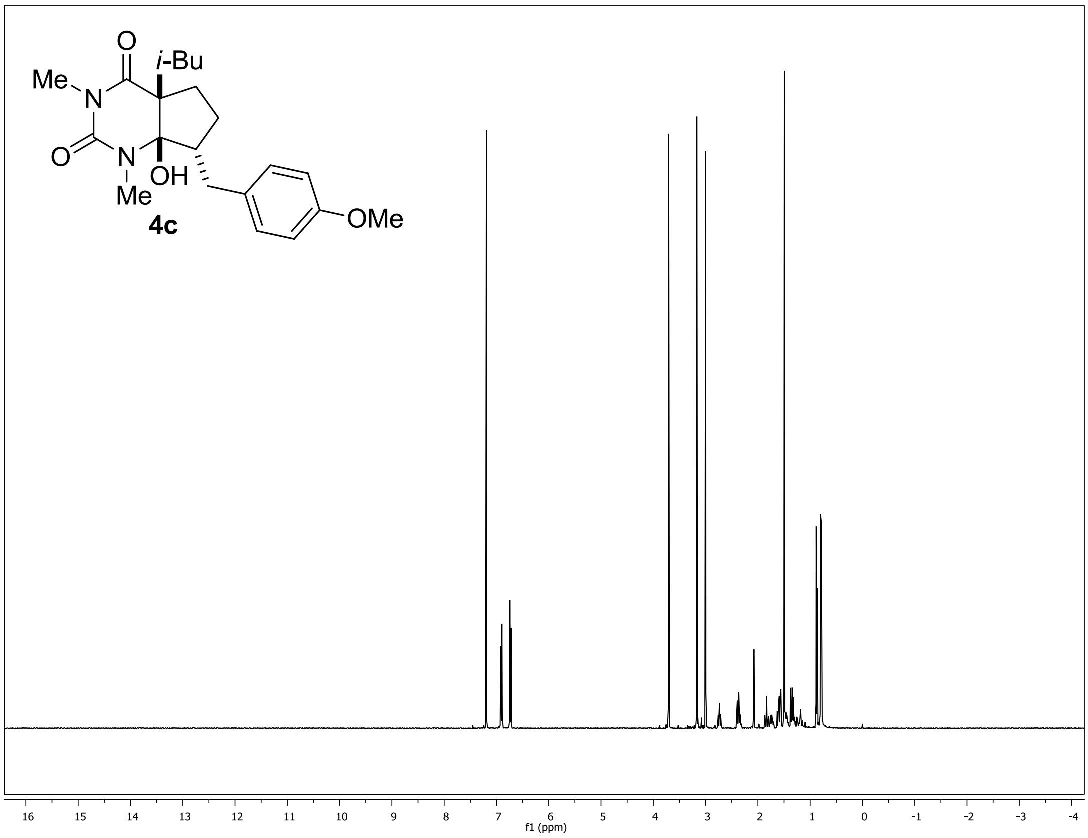
Me
N
O
O
N
Me
i-Bu
OH
OMe
4c
f1 (ppm)

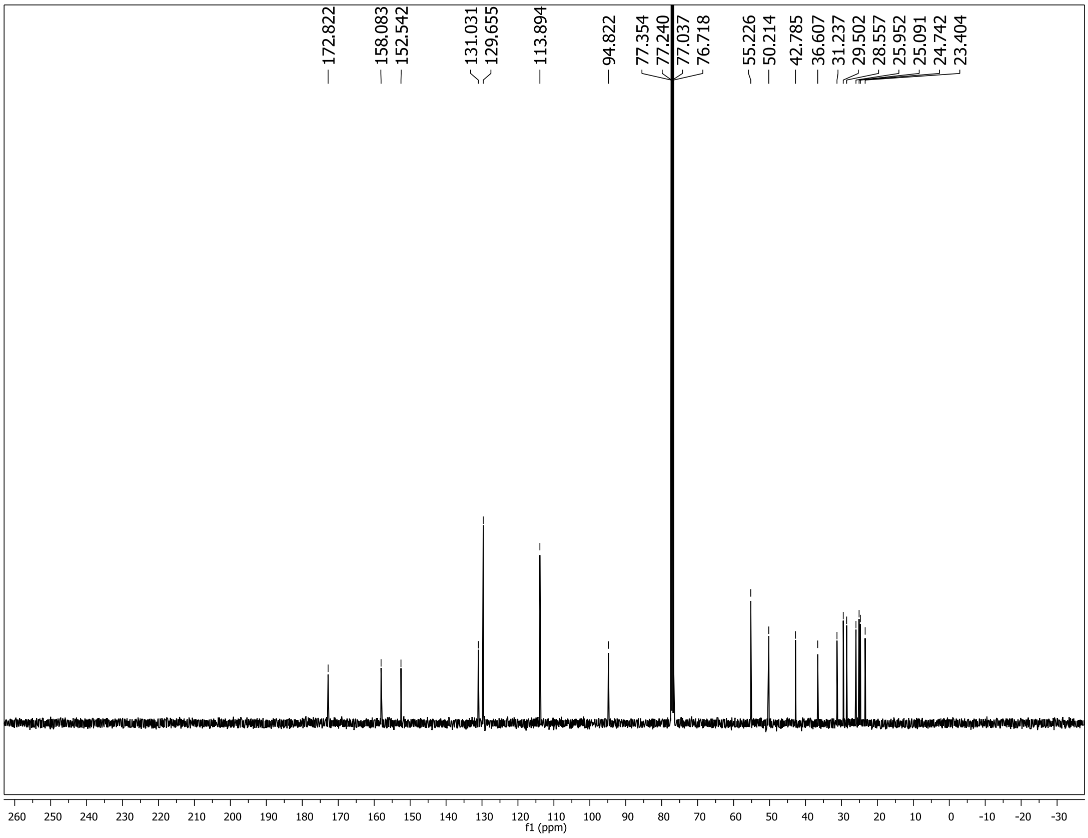
172.822
158.083
152.542
131.031
129.655
113.894
94.822
77.354
77.240
77.037
76.718
55.226
50.214
42.785
36.607
31.237
29.502
28.557
25.952
25.091
24.742
23.404
f1 (ppm)

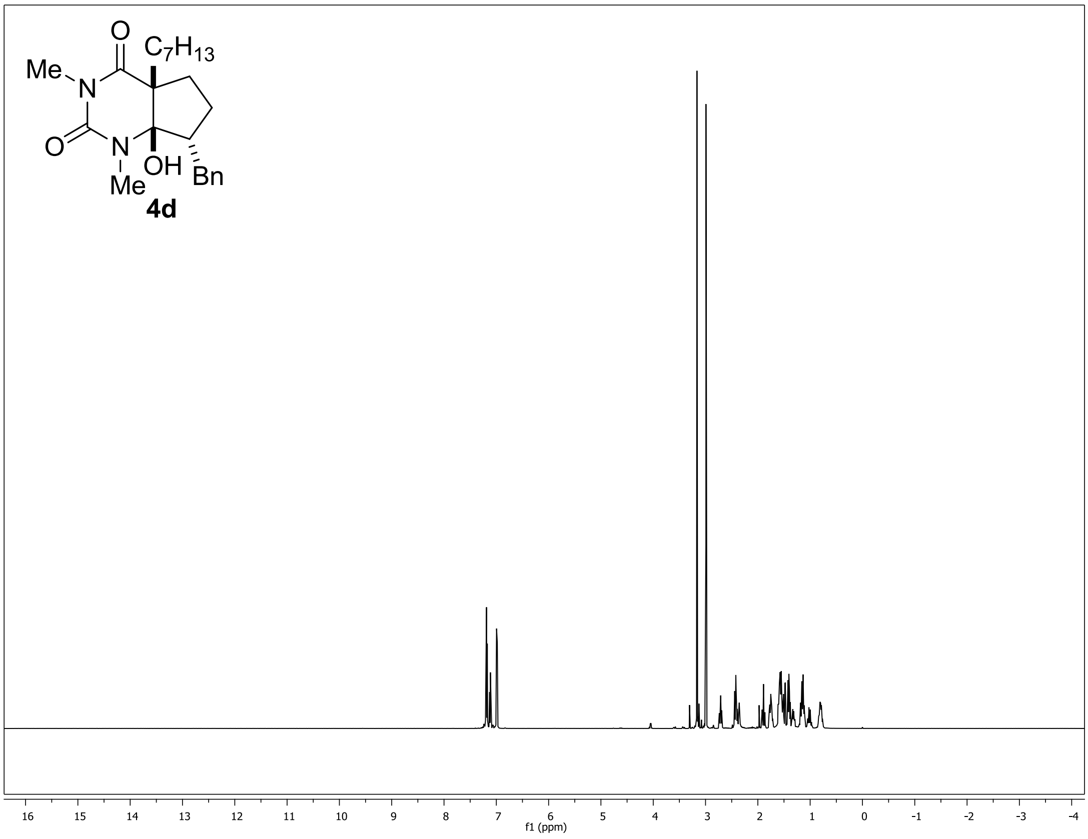
Me
N
O
C7H13
O
N
OH
Me
Bn
4d
f1 (ppm)

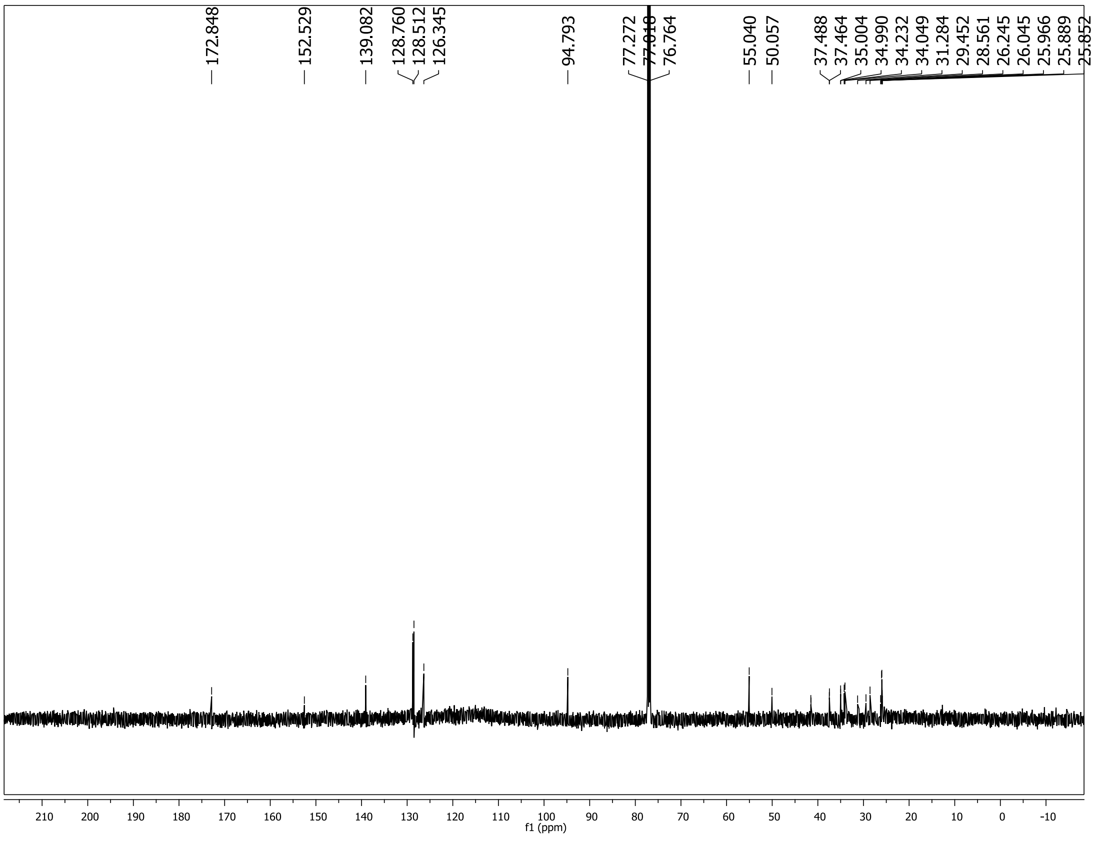
172.848
152.529
139.082
128.760
128.512
126.345
94.793
77.272
76.764
55.040
50.057
37.488
37.464
35.004
34.990
34.232
34.049
31.284
29.452
28.561
26.245
26.045
25.966
25.889
25.852
f1 (ppm)

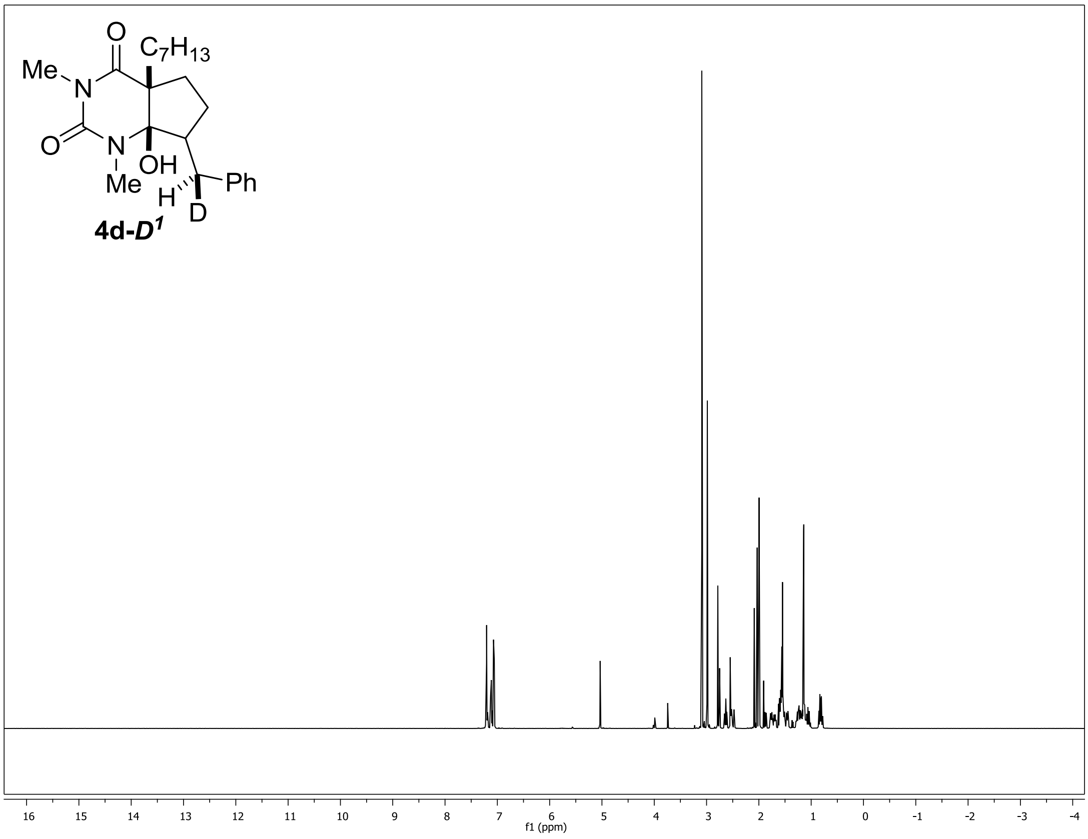

Me
N
O
O
N
Me
OH
H
D
Ph
C7H13
4d-D1
f1 (ppm)

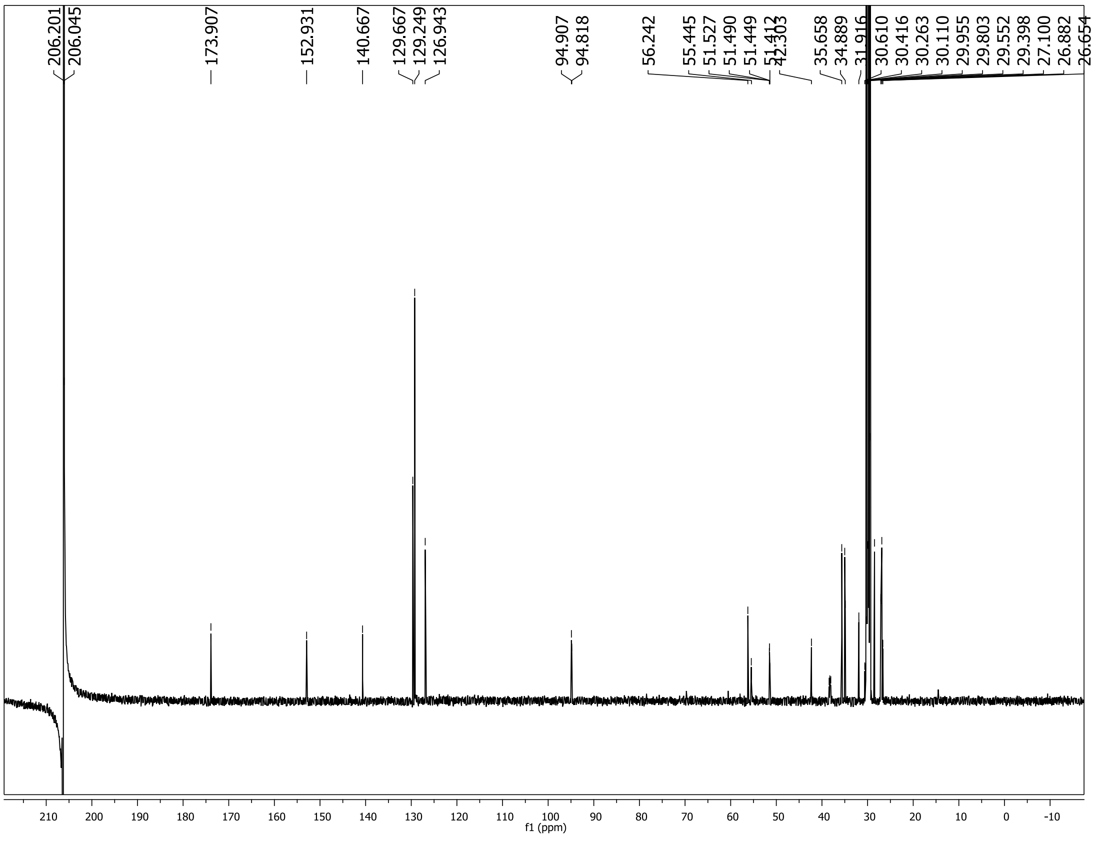

206.201
206.045
173.907
152.931
140.667
129.667
129.249
126.943
94.907
94.818
56.242
55.445
51.527
51.490
51.449
51.412
42.303
35.658
34.889
31.916
30.610
30.416
30.263
30.110
29.955
29.803
29.552
29.398
27.100
26.882
26.654
f1 (ppm)

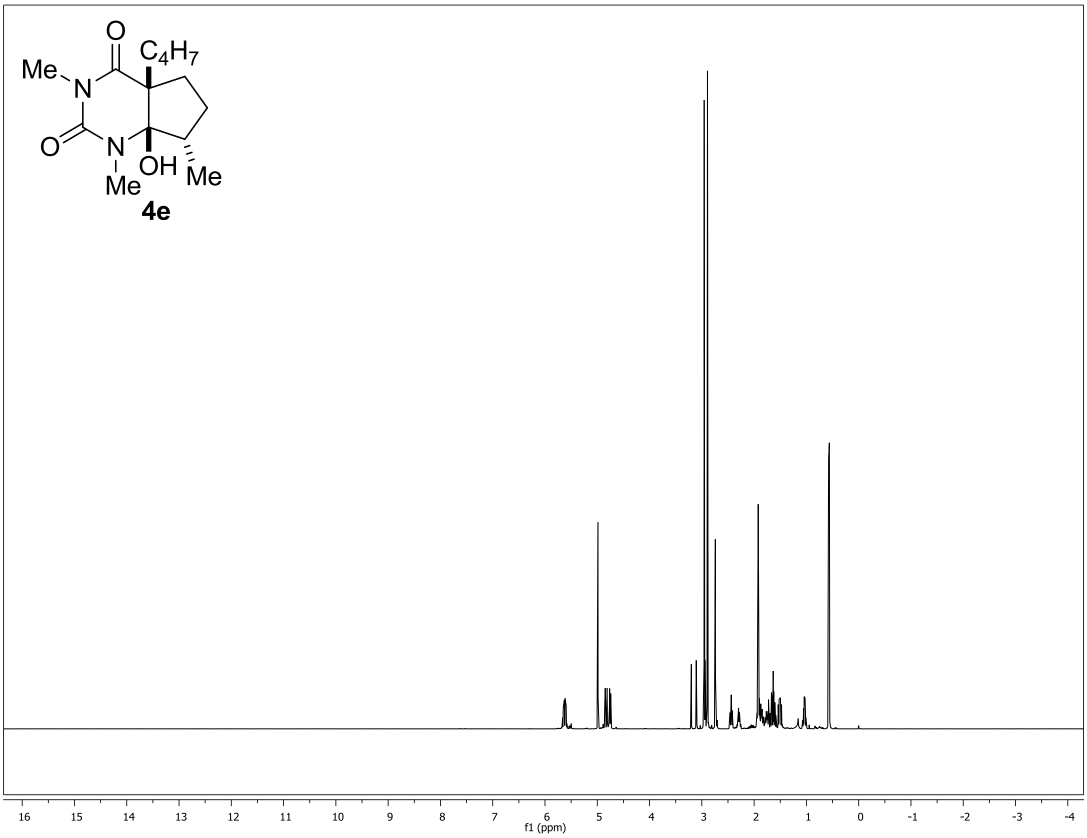
Me
N
O
C4H7
O
N
OH
Me
Me
4e
16 15 14 13 12 11 10 9 8 7 6 5 4 3 2 1 0 -1 -2 -3 -4
f1 (ppm)

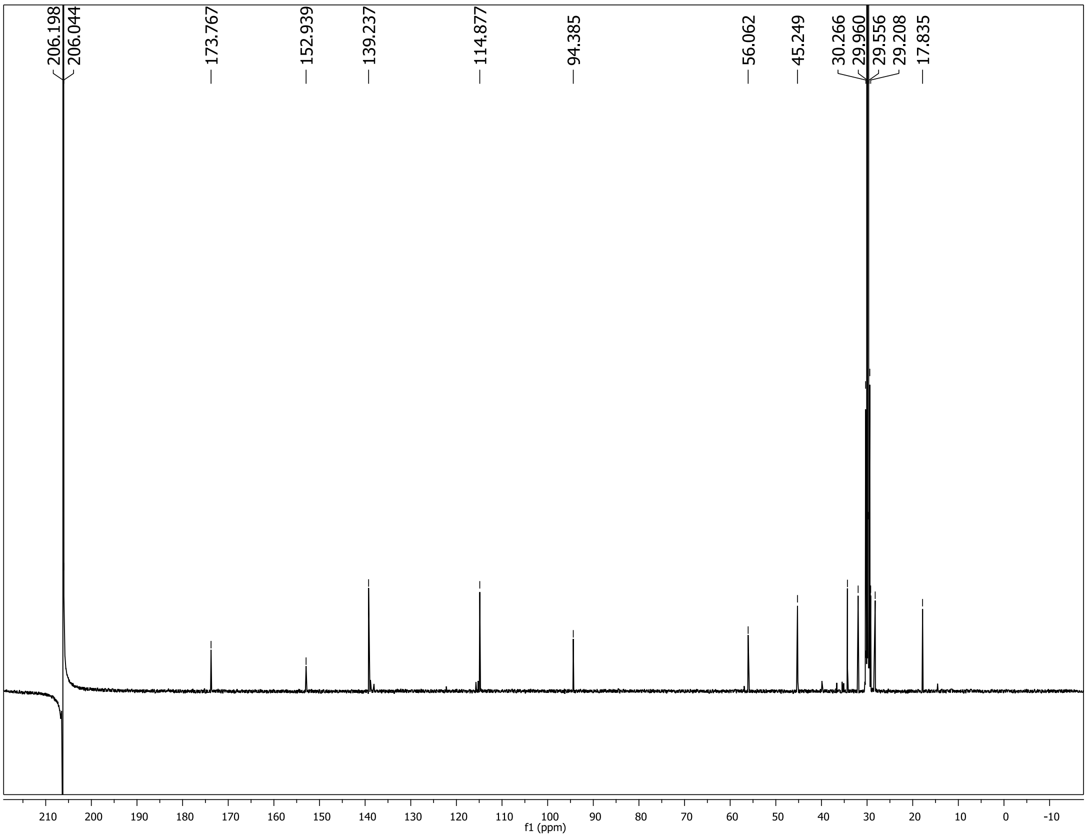
206.198
206.044
173.767
152.939
139.237
114.877
94.385
56.062
45.249
30.266
29.960
29.556
29.208
17.835
210 200 190 180 170 160 150 140 130 120 110 100 90 80 70 60 50 40 30 20 10 0 -10
f1 (ppm)

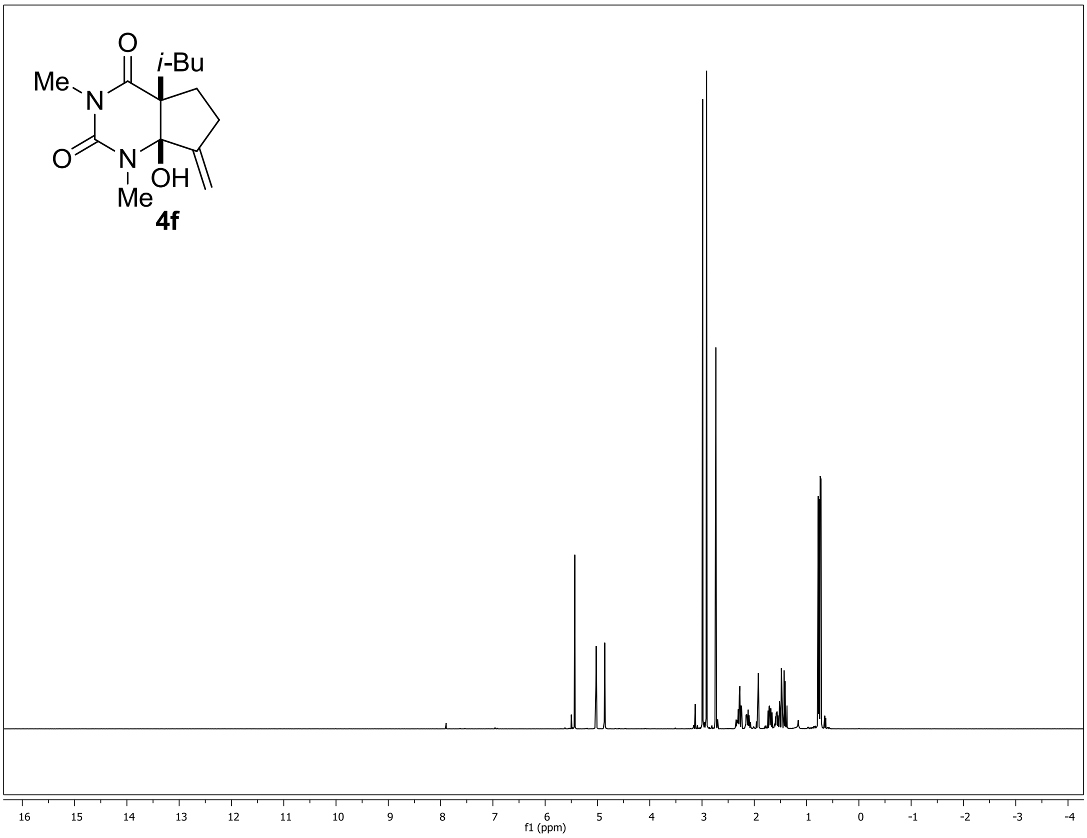
O
i-Bu
Me
N
O
N
OH
Me
4f
16 15 14 13 12 11 10 9 8 7 6 5 4 3 2 1 0 -1 -2 -3 -4
f1 (ppm)

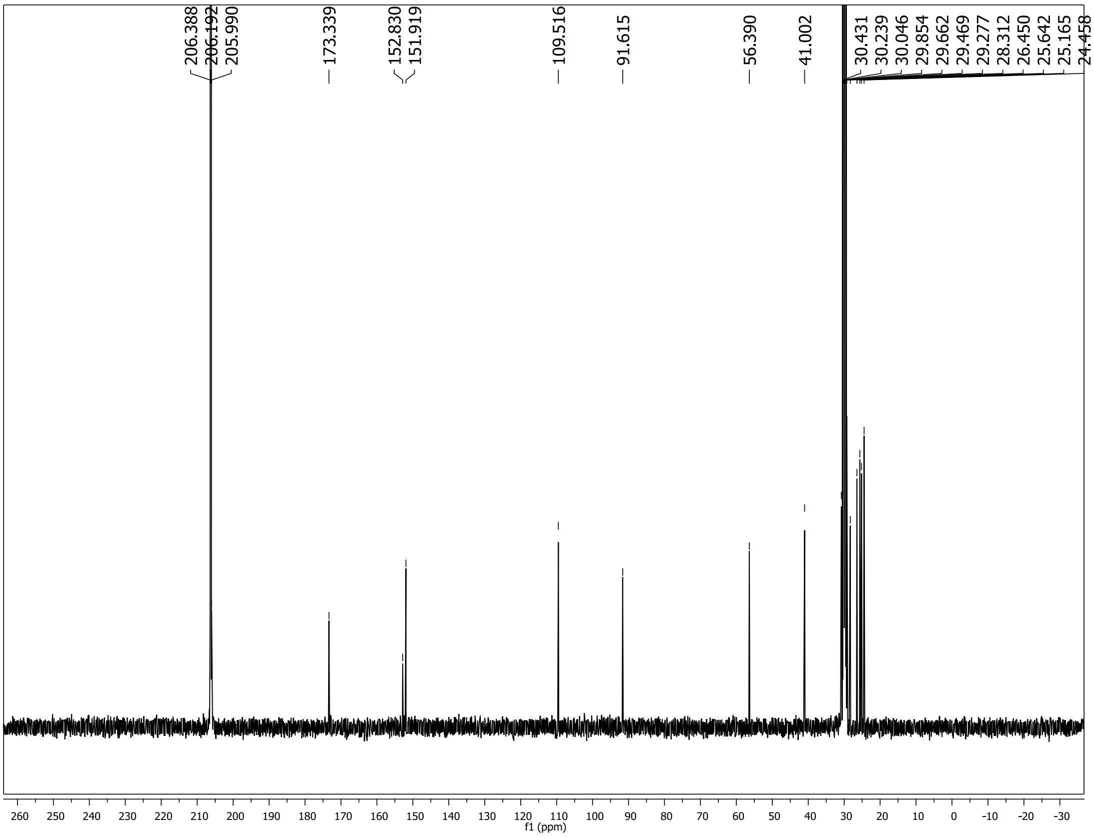
206.388
206.192
205.990
173.339
152.830
151.919
109.516
91.615
56.390
41.002
30.431
30.239
30.046
29.854
29.662
29.469
29.277
28.312
26.450
25.642
25.165
24.458
260 250 240 230 220 210 200 190 180 170 160 150 140 130 120 110 100 90 80 70 60 50 40 30 20 10 0 -10 -20 -30
f1 (ppm)

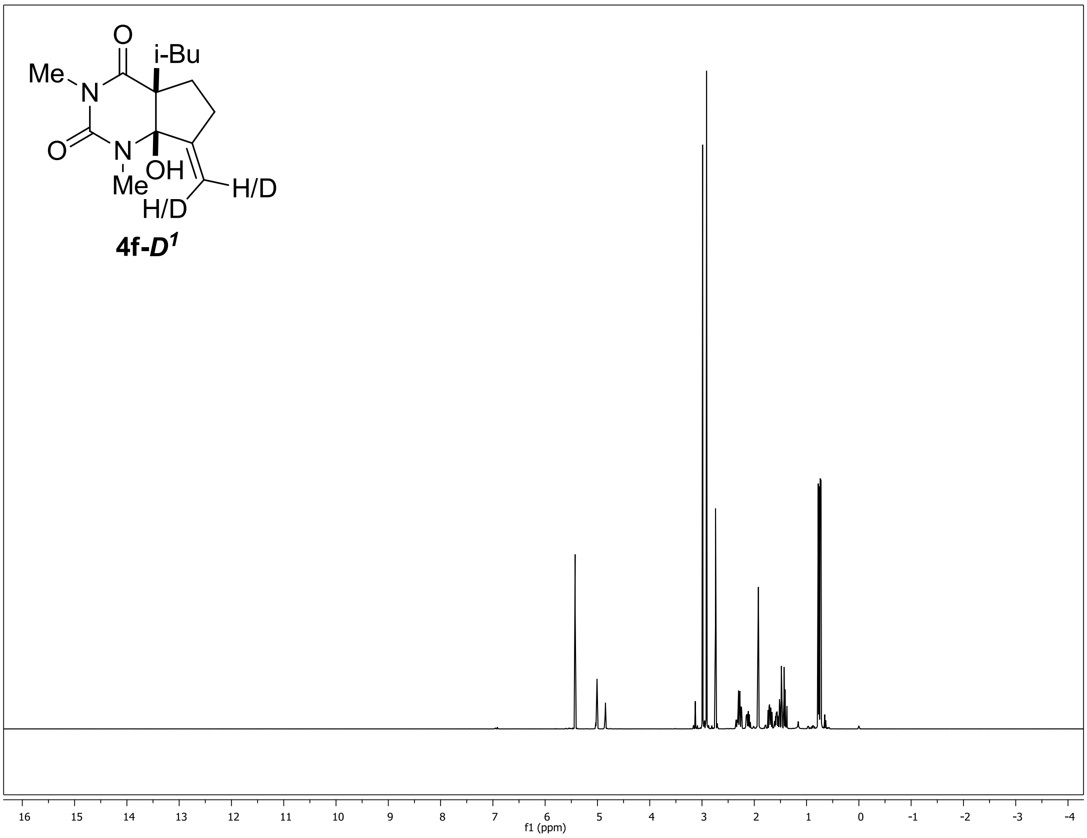

Me
N
O
i-Bu
O
N
Me
OH
H/D
H/D
4f-D1
f1 (ppm)

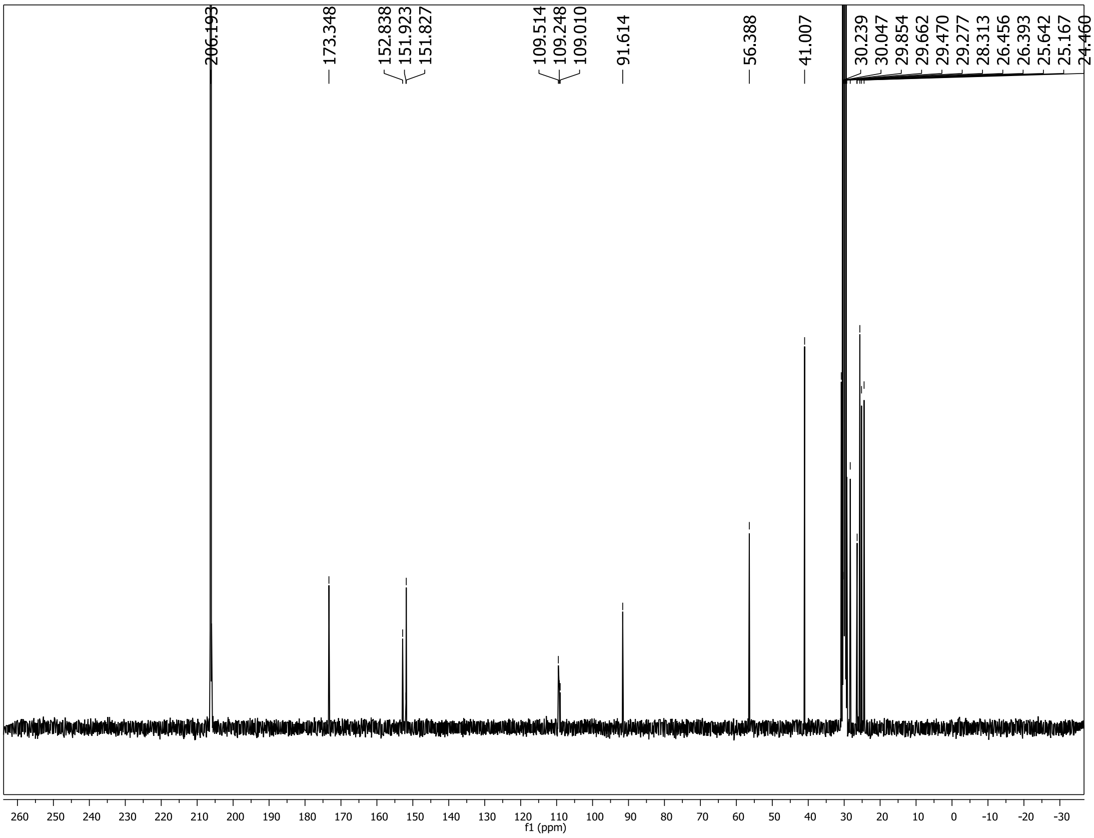

206.193
173.348
152.838
151.923
151.827
109.514
109.248
109.010
91.614
56.388
41.007
30.239
30.047
29.854
29.662
29.470
29.277
28.313
26.456
26.393
25.642
25.167
24.460
f1 (ppm)

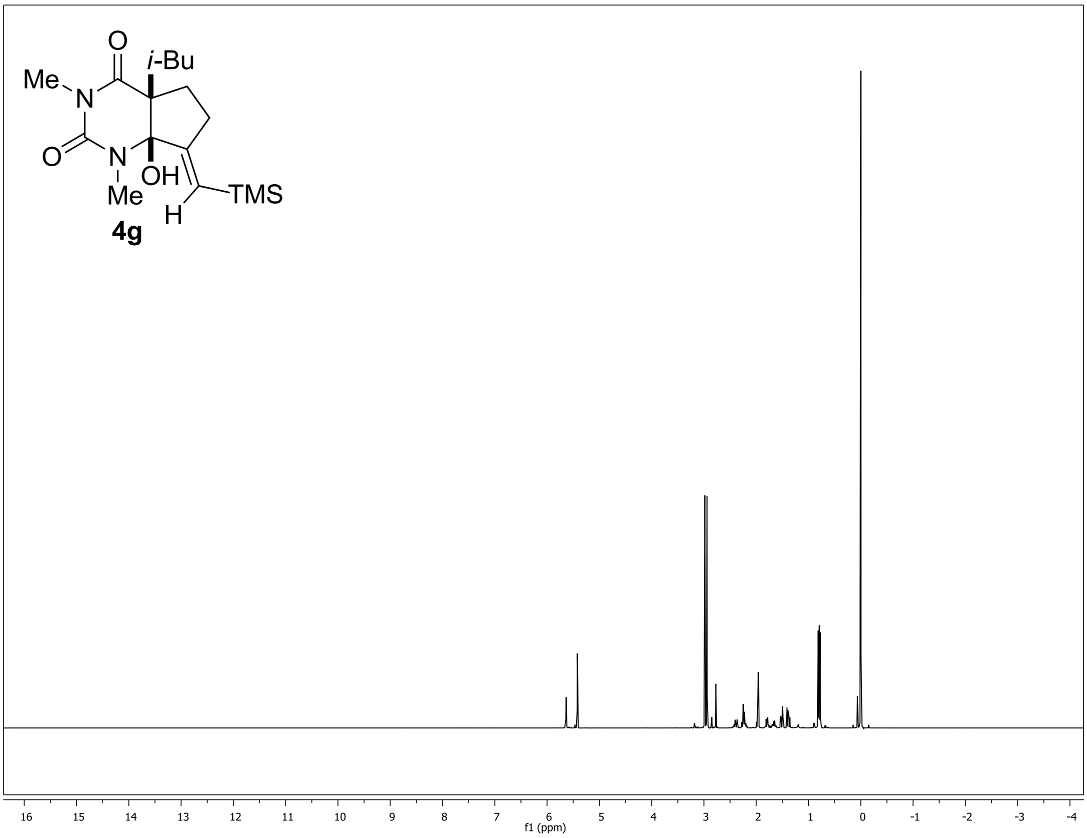
Me
N
O
i-Bu
N
OH
Me
H
TMS
4g
f1 (ppm)

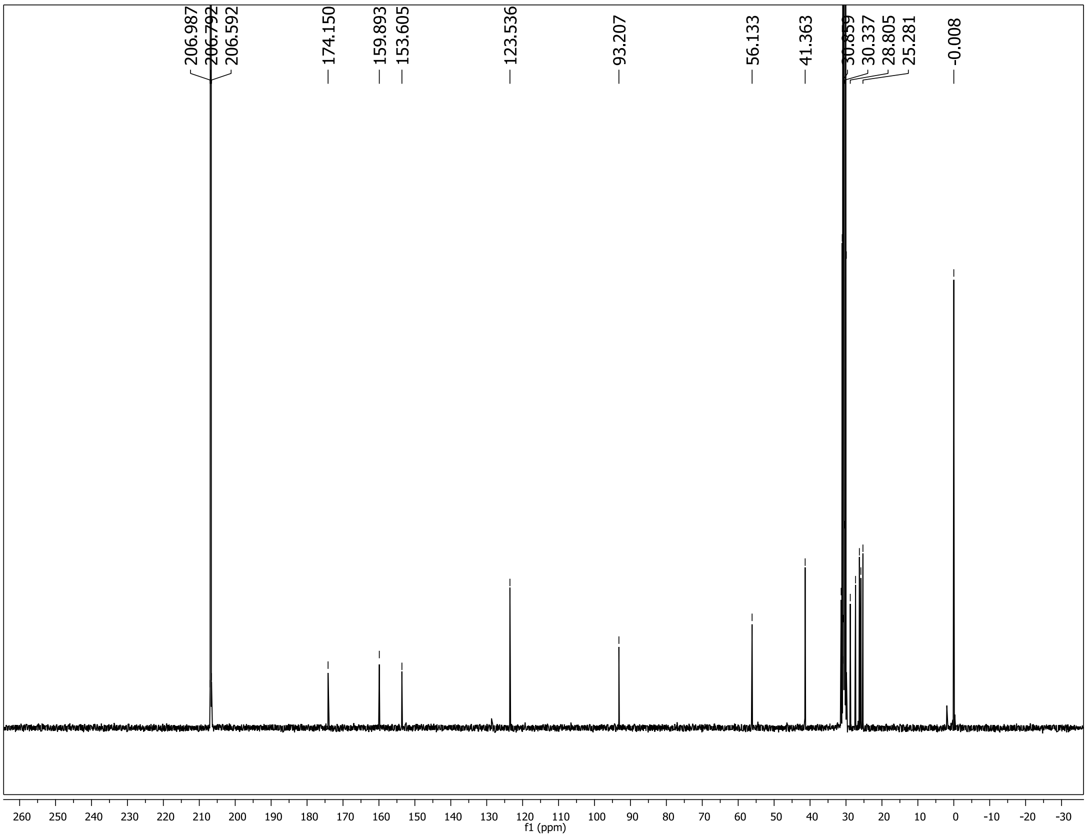
206.987
206.792
206.592
174.150
159.893
153.605
123.536
93.207
56.133
41.363
30.337
28.805
25.281
-0.008
f1 (ppm)

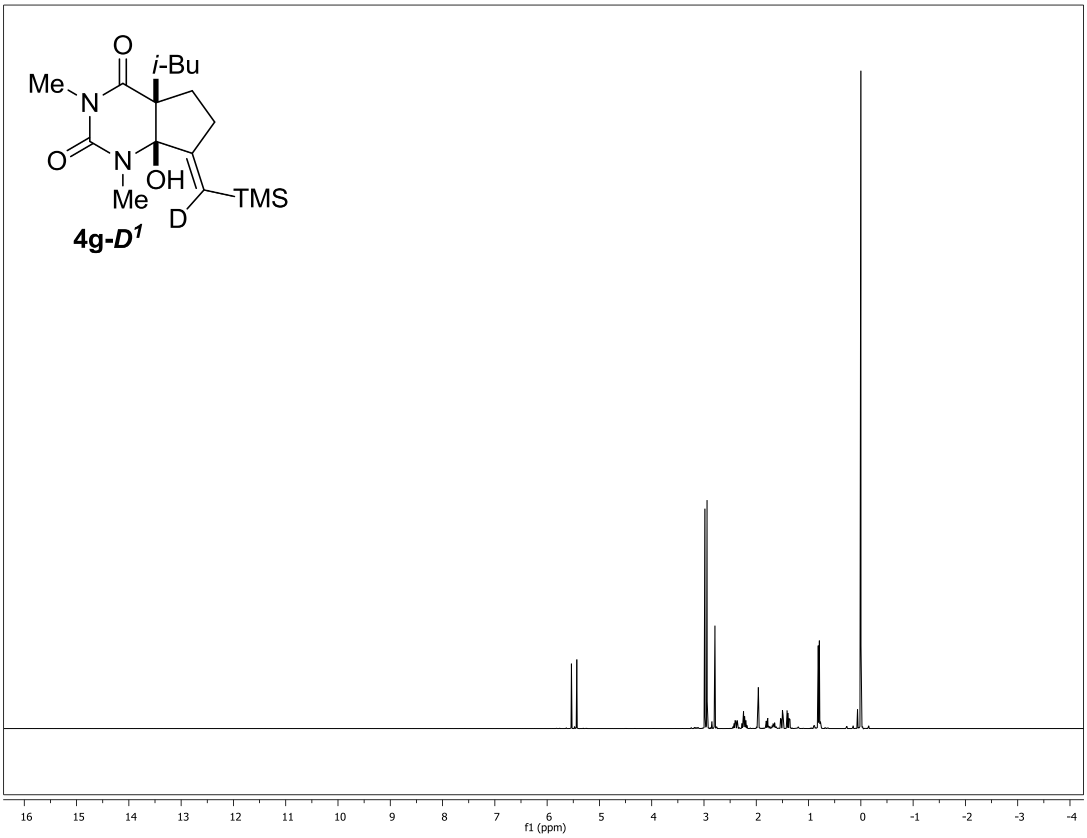
O
i-Bu
Me
N
O
N
OH
Me
TMS
D
4g-*D*[1]
f1 (ppm)

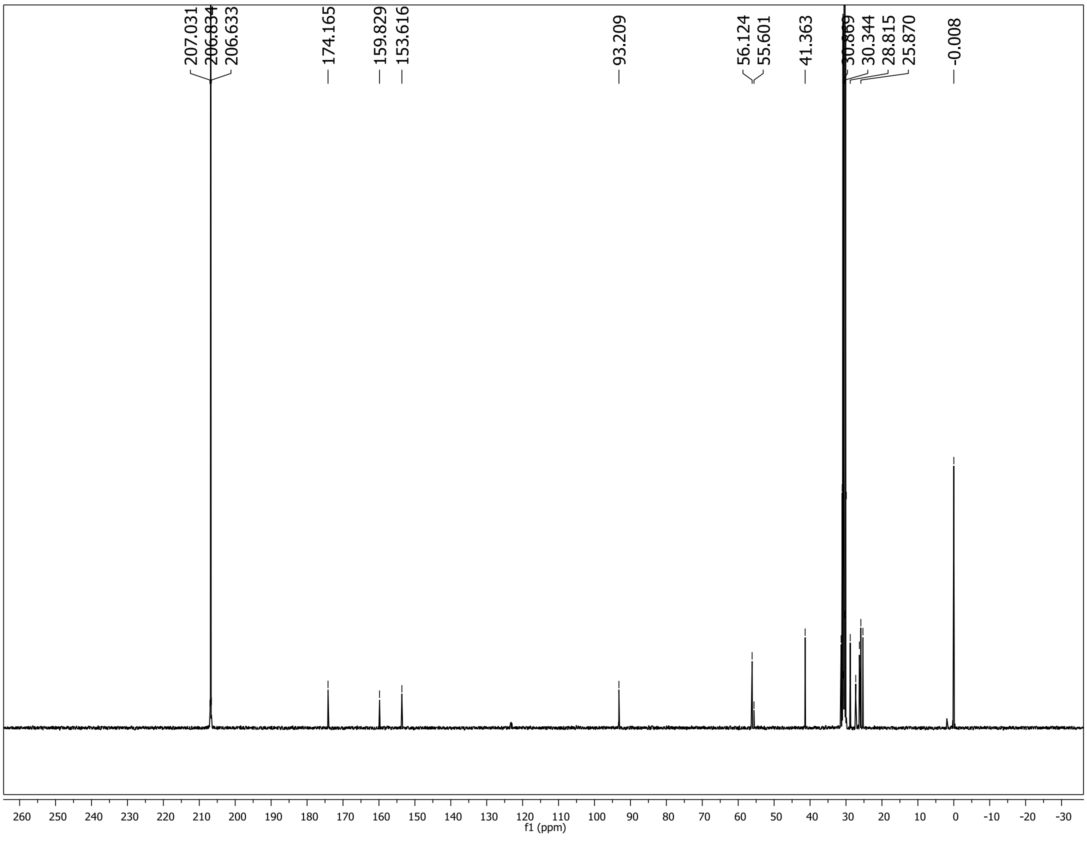
207.031
206.834
206.633
174.165
159.829
153.616
93.209
56.124
55.601
41.363
30.344
28.815
25.870
-0.008
f1 (ppm)

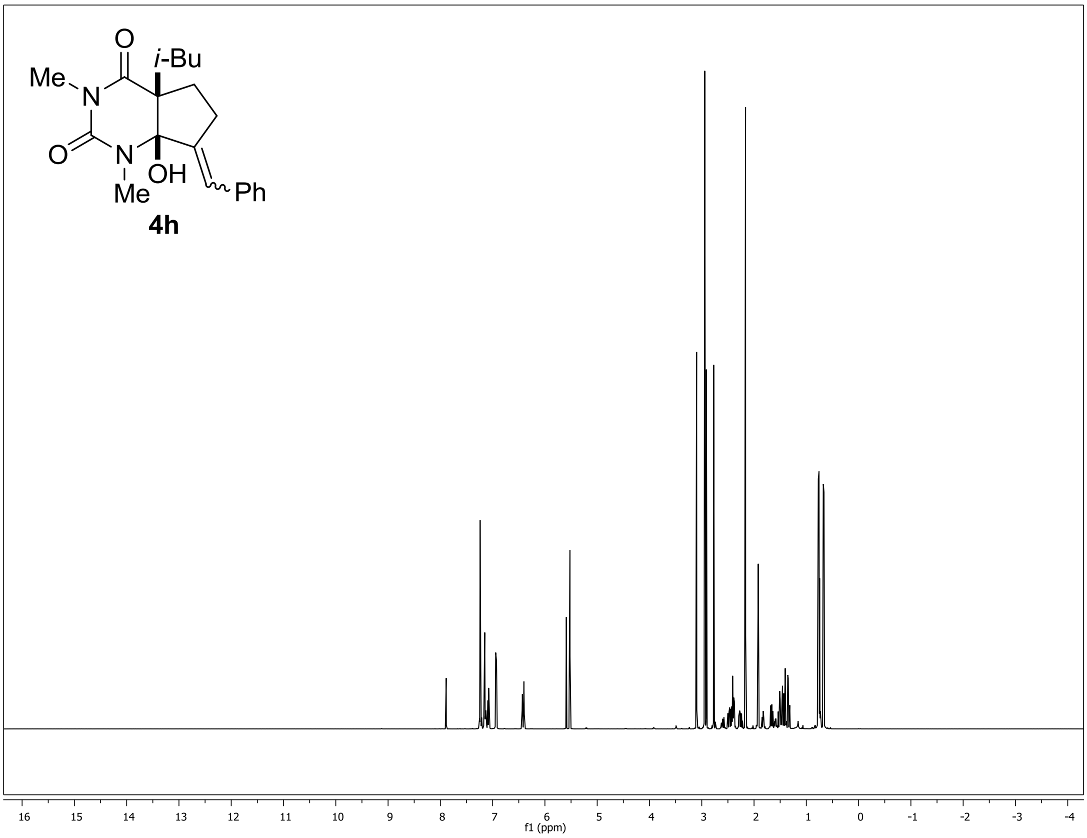
O
i-Bu
Me
N
O
N
OH
Me
Ph
4h
16 15 14 13 12 11 10 9 8 7 6 5 4 3 2 1 0 -1 -2 -3 -4
f1 (ppm)

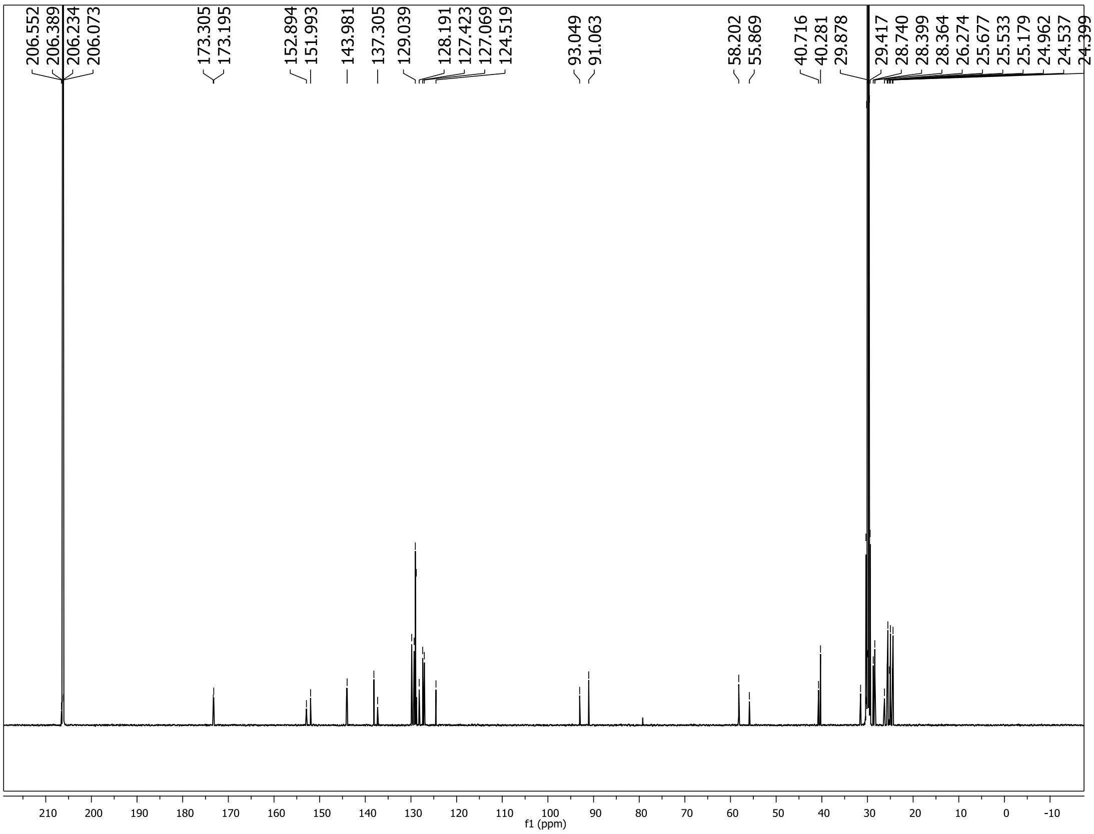
206.552
206.389
206.234
206.073
173.305
173.195
152.894
151.993
143.981
137.305
129.039
128.191
127.423
127.069
124.519
93.049
91.063
58.202
55.869
40.716
40.281
29.878
29.417
28.740
28.399
28.364
26.274
25.677
25.533
25.179
24.962
24.537
24.399
210 200 190 180 170 160 150 140 130 120 110 100 90 80 70 60 50 40 30 20 10 0 -10
f1 (ppm)

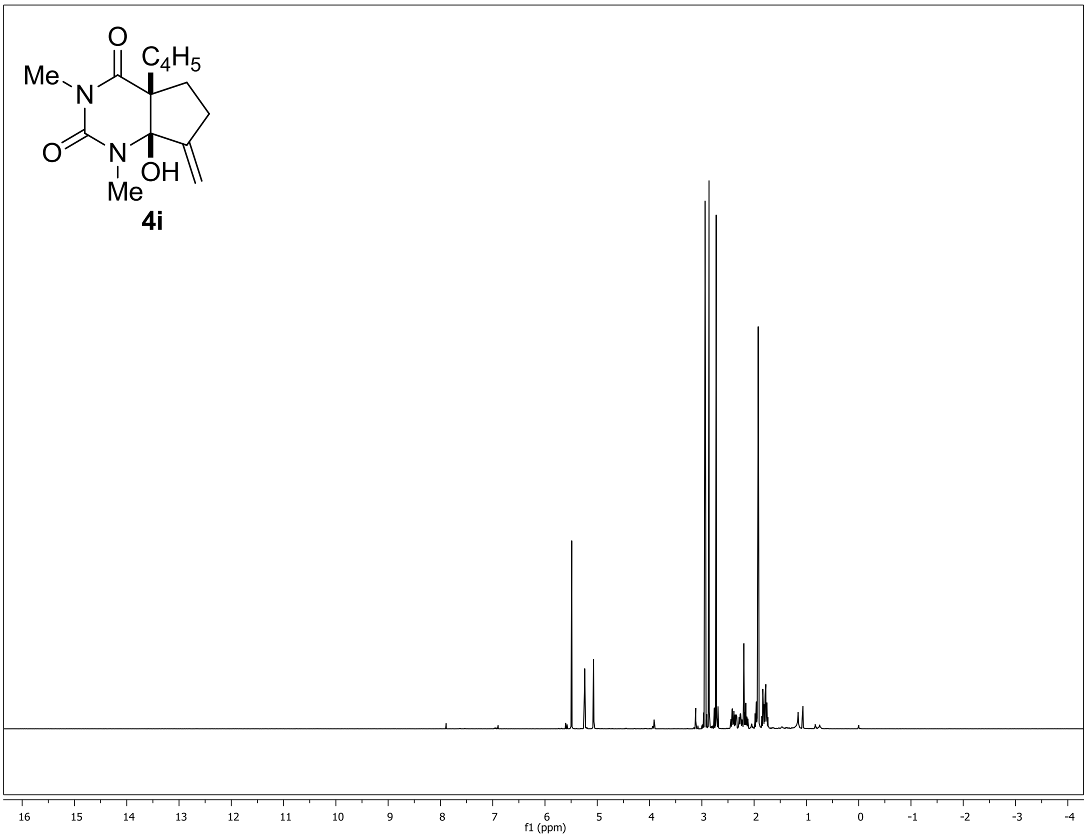
Me
N
O
C4H5
N
O
OH
Me
4i
f1 (ppm)

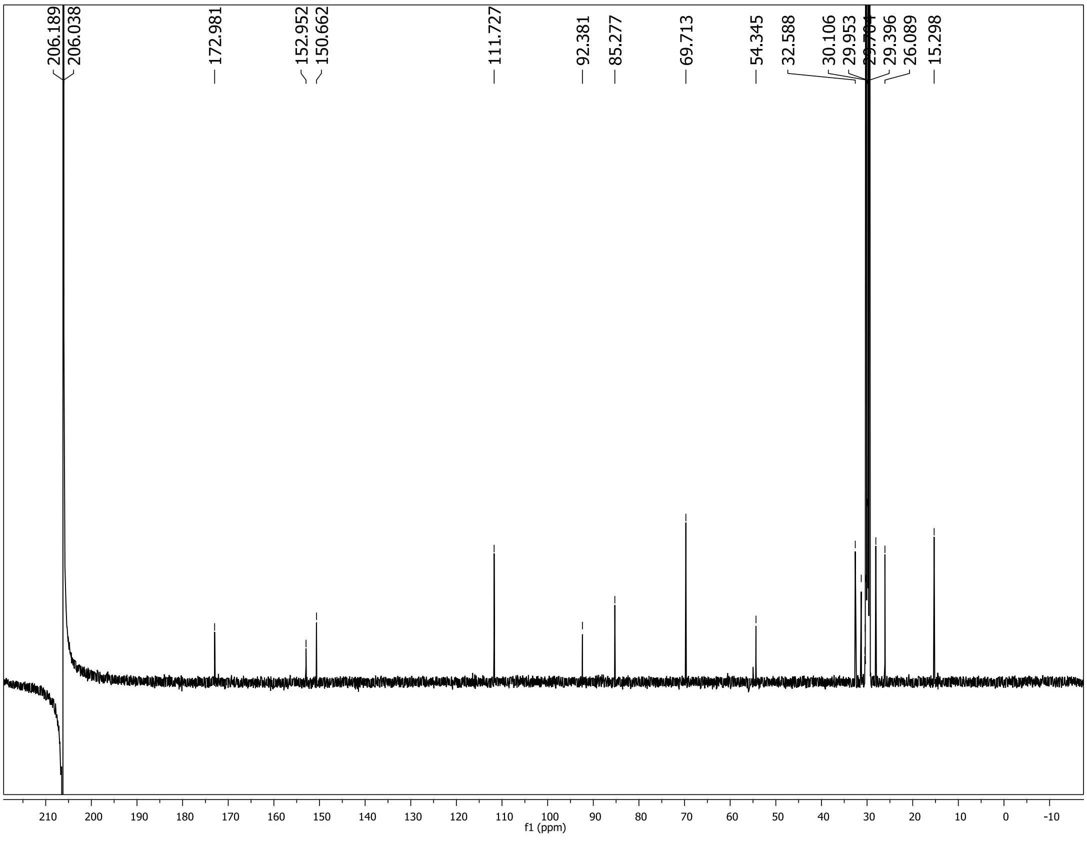
206.189
206.038
172.981
152.952
150.662
111.727
92.381
85.277
69.713
54.345
32.588
30.106
29.953
29.396
26.089
15.298
f1 (ppm)

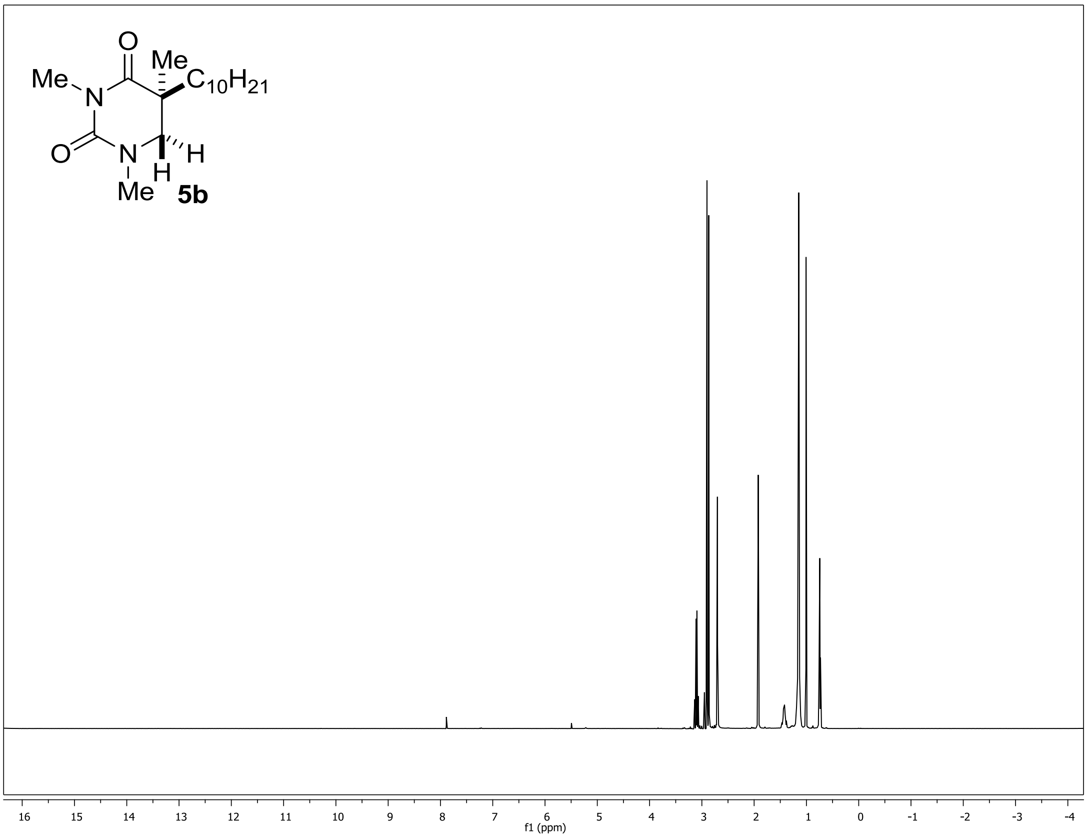
Me
O
Me
N
C10H21
O
N
H
H
Me
5b
f1 (ppm)

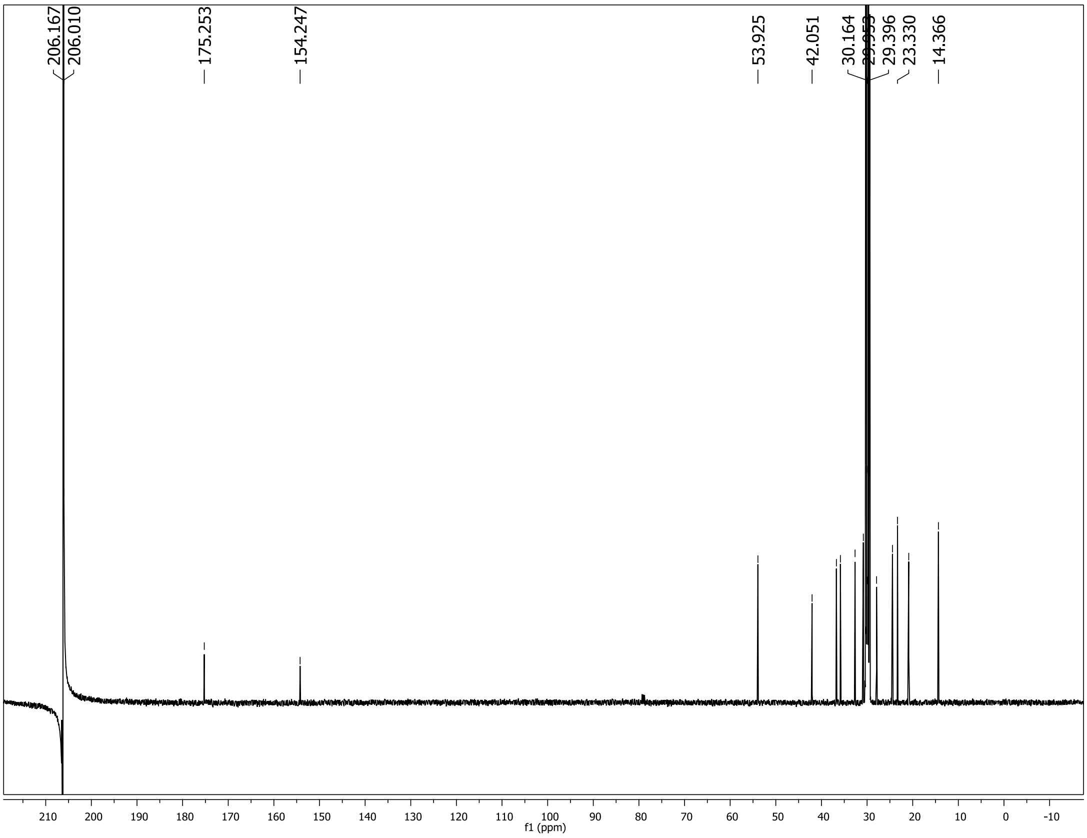
206.167
206.010
175.253
154.247
53.925
42.051
30.164
29.396
23.330
14.366
f1 (ppm)

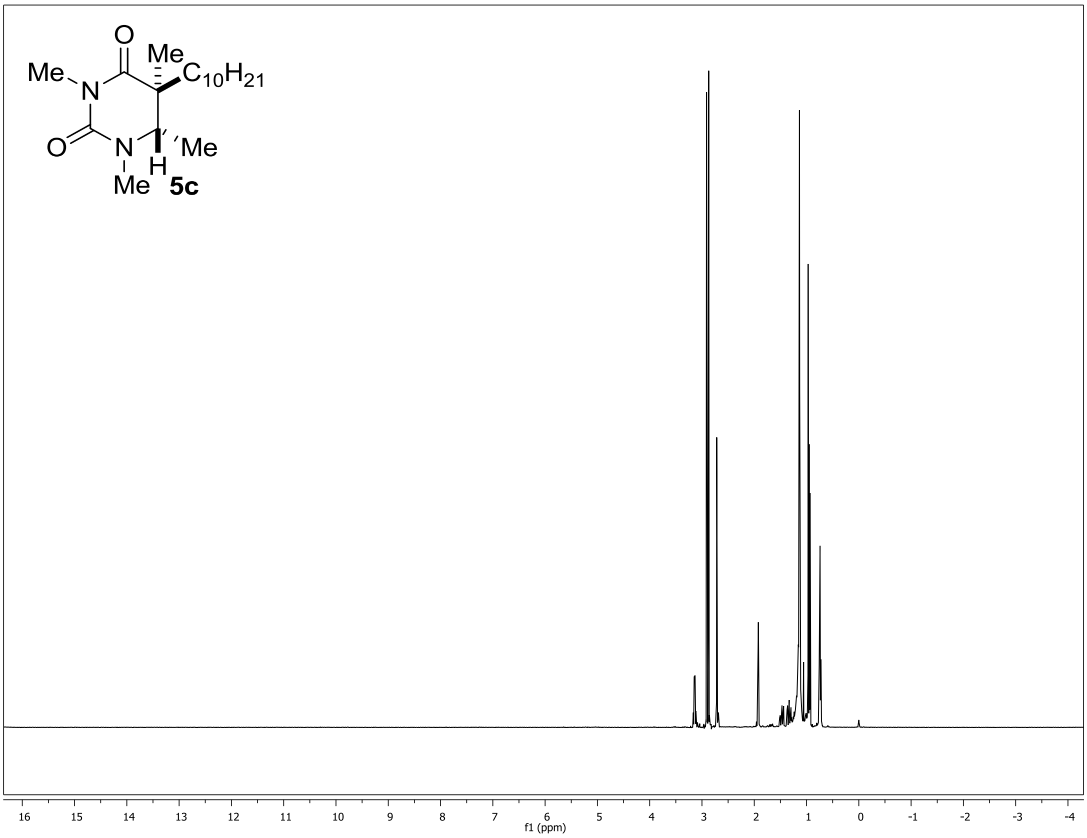
Me
C10H21
Me
5c
16 15 14 13 12 11 10 9 8 7 6 5 4 3 2 1 0 -1 -2 -3 -4
f1 (ppm)

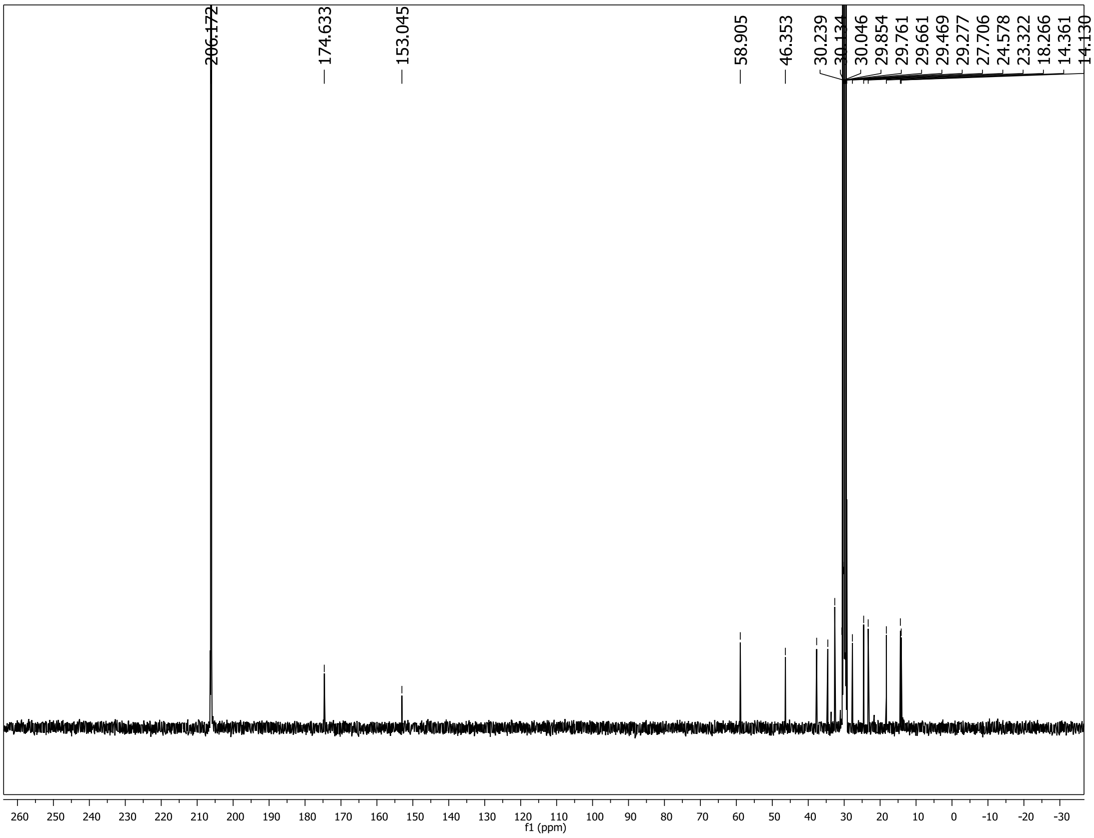
206.172
174.633
153.045
58.905
46.353
30.239
30.134
30.046
29.854
29.761
29.661
29.469
29.277
27.706
24.578
23.322
18.266
14.361
14.130
260 250 240 230 220 210 200 190 180 170 160 150 140 130 120 110 100 90 80 70 60 50 40 30 20 10 0 -10 -20 -30
f1 (ppm)

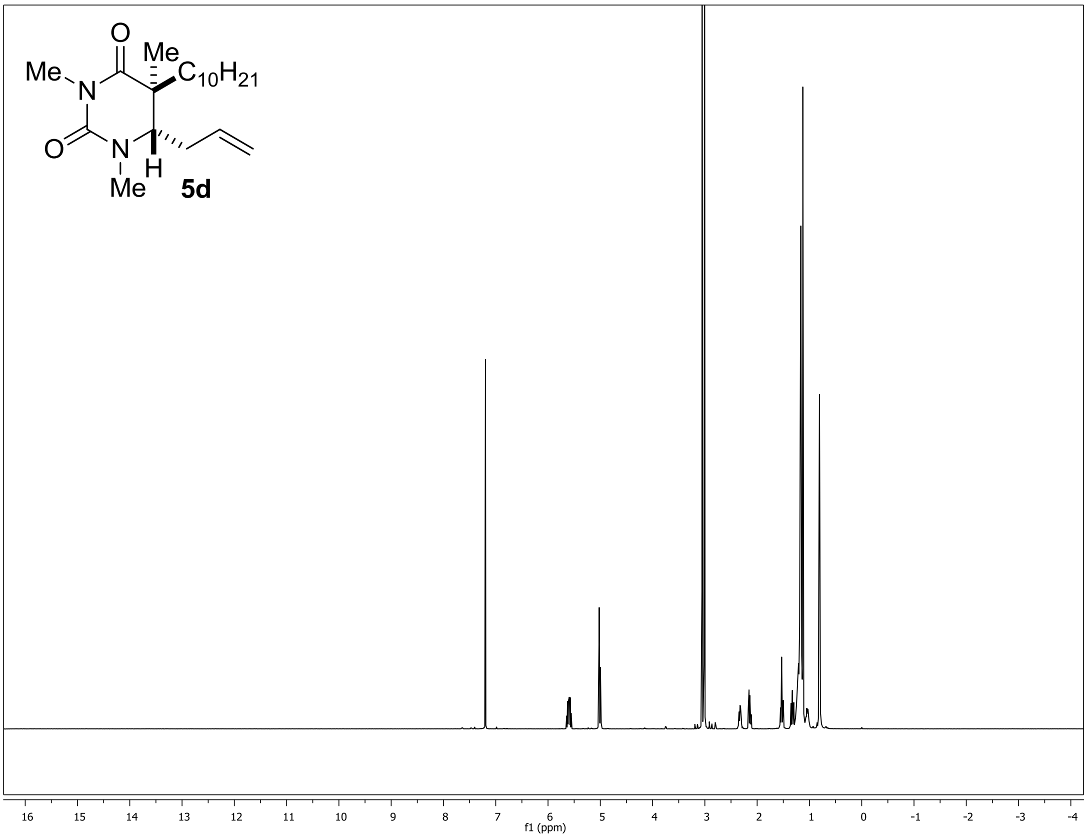
Me
N
O
Me
$C_{10}H_{21}$
O
N
H
Me
5d
16 15 14 13 12 11 10 9 8 7 6 5 4 3 2 1 0 -1 -2 -3 -4
f1 (ppm)

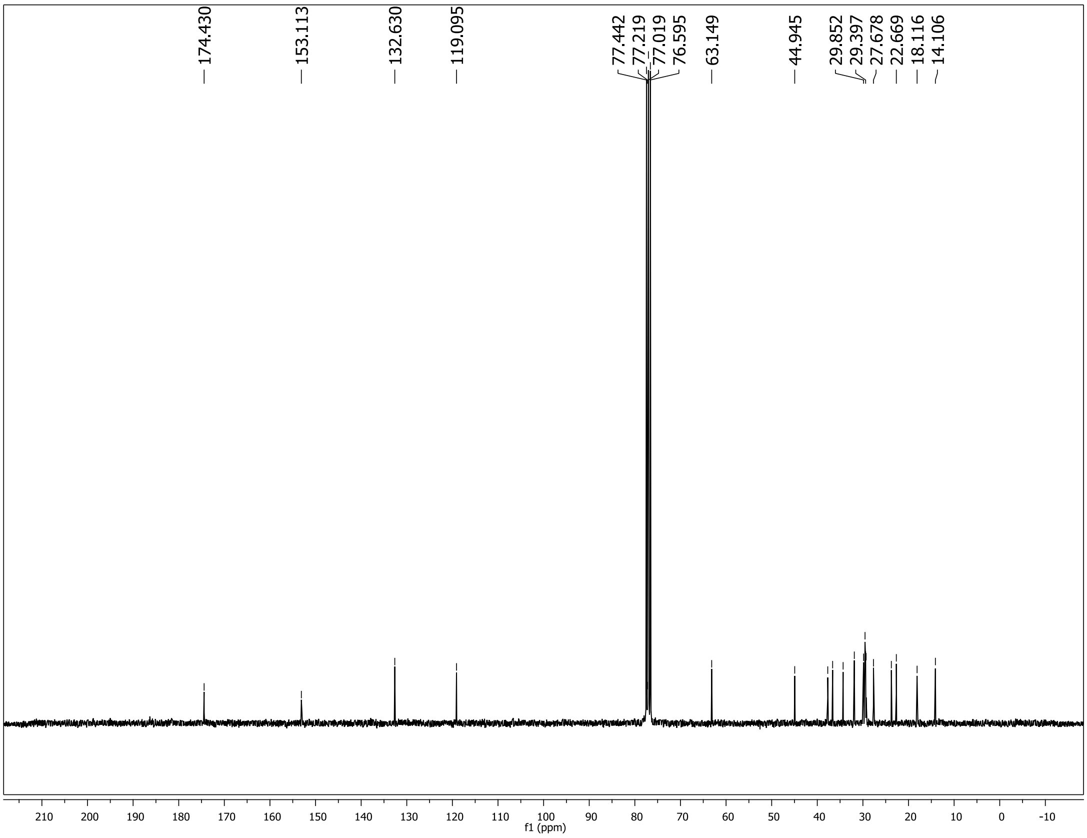
174.430
153.113
132.630
119.095
77.442
77.219
77.019
76.595
63.149
44.945
29.852
29.397
27.678
22.669
18.116
14.106
210 200 190 180 170 160 150 140 130 120 110 100 90 80 70 60 50 40 30 20 10 0 -10
f1 (ppm)

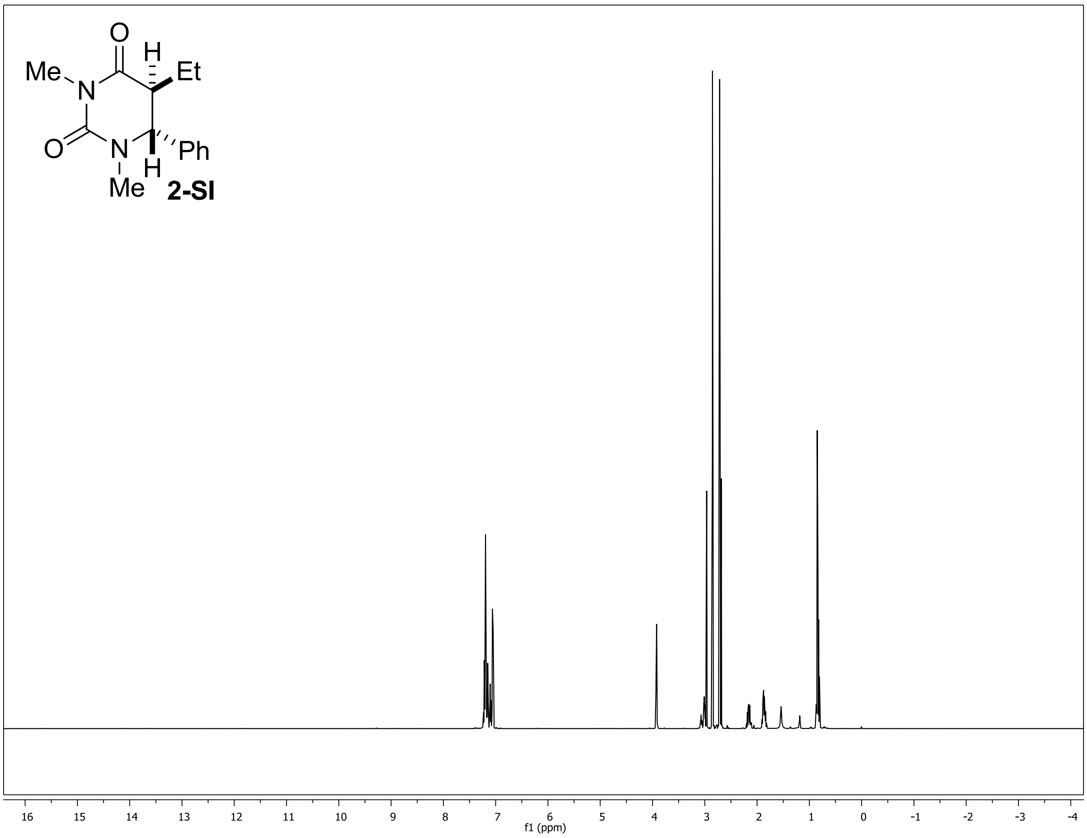
Me
N
O
H
Et
O
N
Ph
H
Me
2-SI
16 15 14 13 12 11 10 9 8 7 6 5 4 3 2 1 0 -1 -2 -3 -4
f1 (ppm)

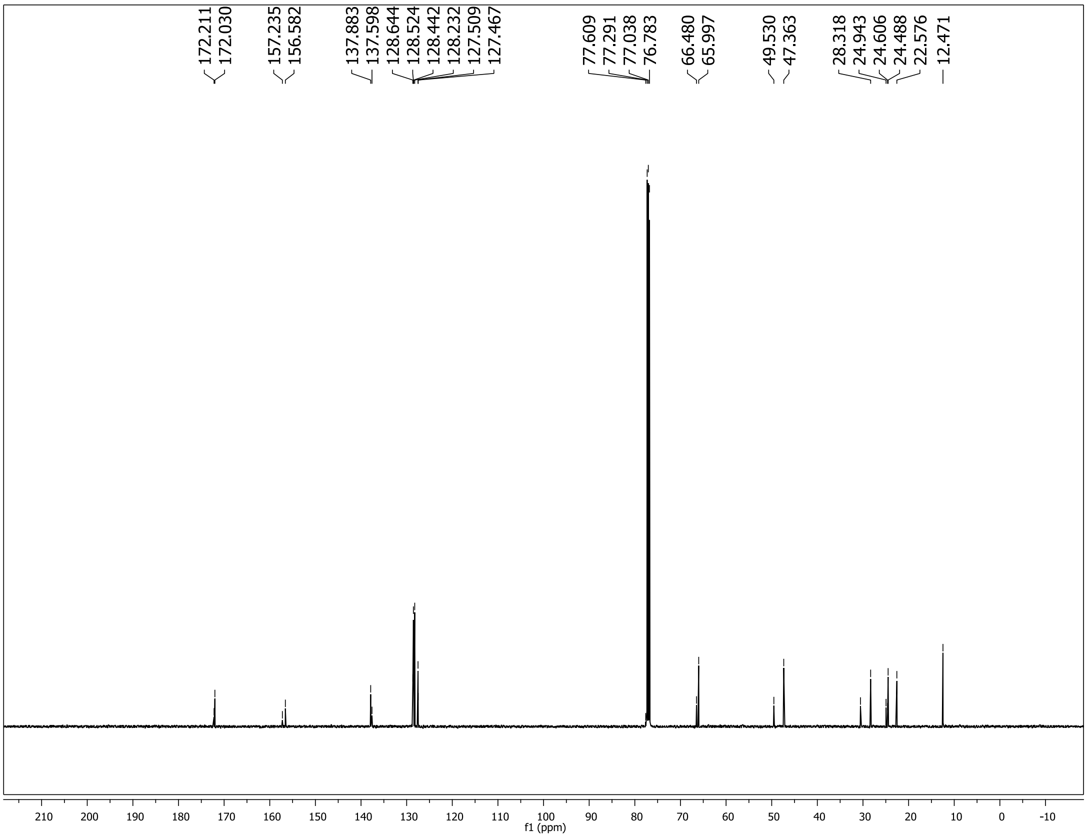
172.211
172.030
157.235
156.582
137.883
137.598
128.644
128.524
128.442
128.232
127.509
127.467
77.609
77.291
77.038
76.783
66.480
65.997
49.530
47.363
28.318
24.943
24.606
24.488
22.576
12.471
210 200 190 180 170 160 150 140 130 120 110 100 90 80 70 60 50 40 30 20 10 0 -10
f1 (ppm)

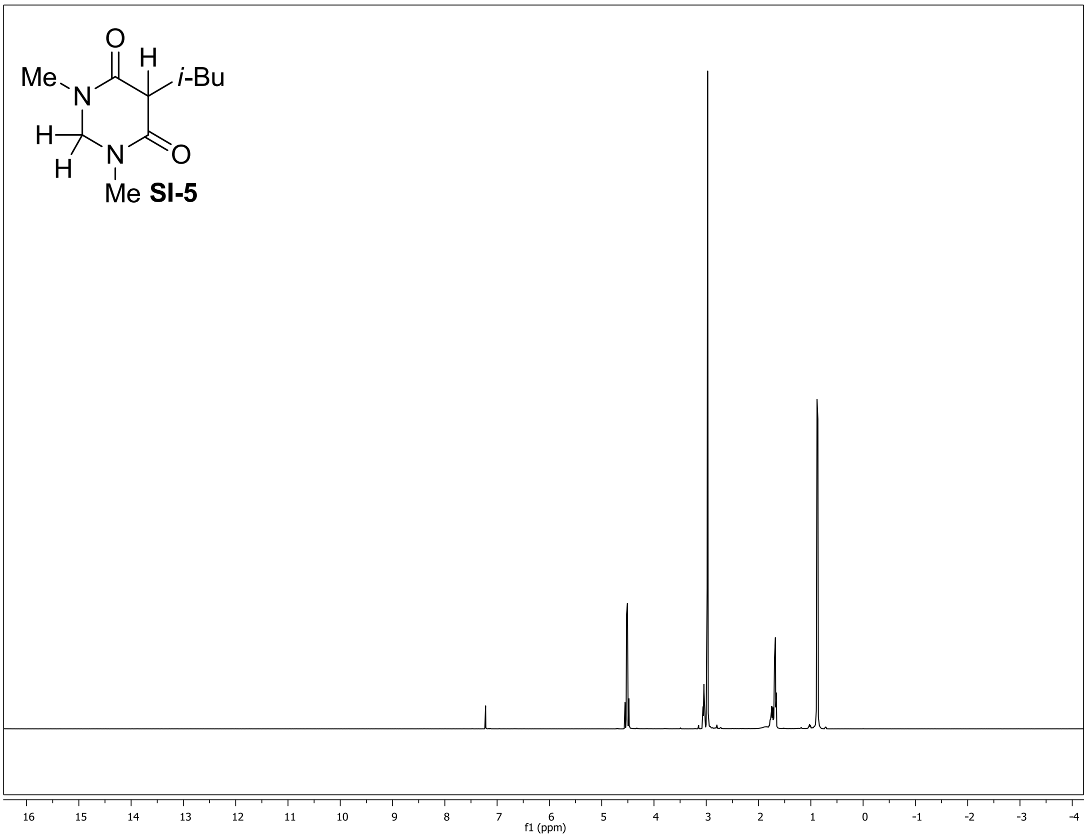
Me
N
H
i-Bu
O
H
H
N
O
Me **SI-5**
16
15
14
13
12
11
10
9
8
7
6
5
4
3
2
1
0
-1
-2
-3
-4
f1 (ppm)

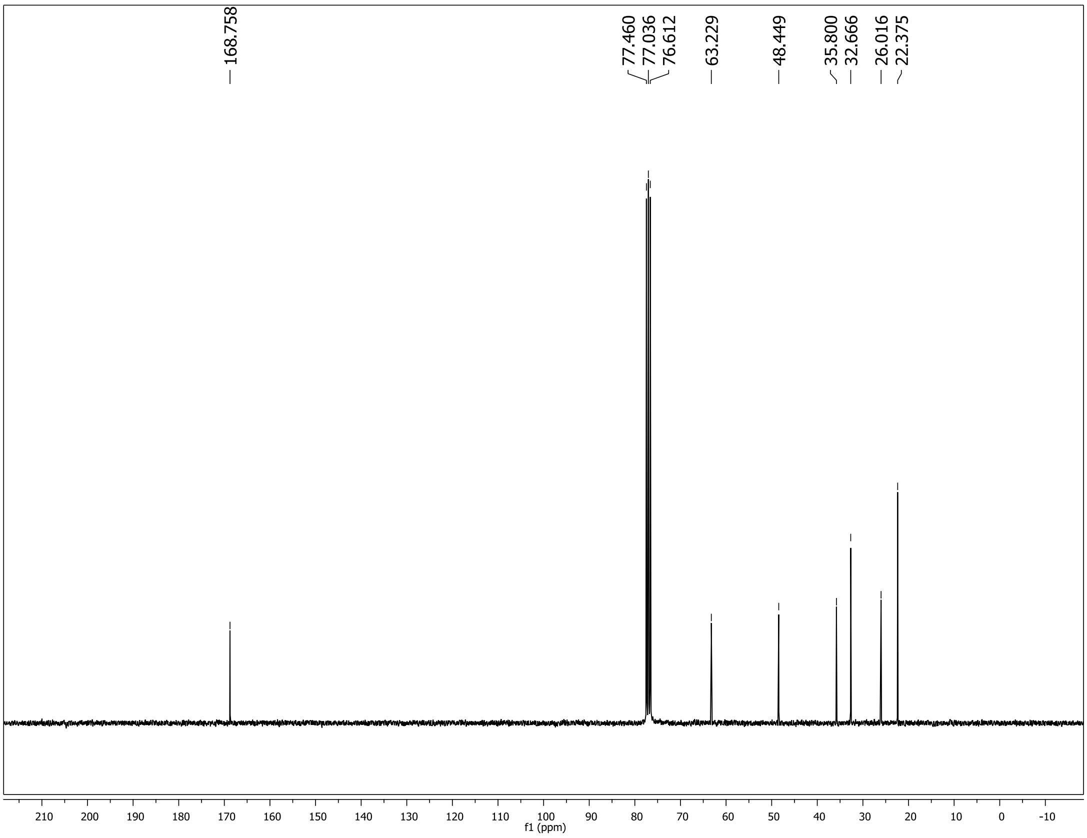
168.758
77.460
77.036
76.612
63.229
48.449
35.800
32.666
26.016
22.375
210
200
190
180
170
160
150
140
130
120
110
100
90
80
70
60
50
40
30
20
10
0
-10
f1 (ppm)
